# SynProtX: a large-scale proteomics-based deep learning model for predicting synergistic anticancer drug combinations

**DOI:** 10.1093/gigascience/giaf080

**Published:** 2025-08-12

**Authors:** Bundit Boonyarit, Matin Kositchutima, Tisorn Na Phattalung, Nattawin Yamprasert, Chanitra Thuwajit, Thanyada Rungrotmongkol, Sarana Nutanong

**Affiliations:** School of Information Science and Technology, Vidyasirimedhi Institute of Science and Technology, Rayong 21210, Thailand; Kamnoetvidya Science Academy, Rayong 21210, Thailand; Kamnoetvidya Science Academy, Rayong 21210, Thailand; School of Information, Computer, and Communication Technology, Sirindhorn International Institute of Technology, Thammasat University, Pathum Thani 12120, Thailand; Department of Immunology, Faculty of Medicine Siriraj Hospital, Mahidol University, Bangkok 10700, Thailand; Program in Bioinformatics and Computational Biology, Graduate School, Chulalongkorn University, Bangkok 10330, Thailand; Center of Excellence in Structural and Computational Biology, Department of Biochemistry, Faculty of Science, Chulalongkorn University, Bangkok 10330, Thailand; School of Information Science and Technology, Vidyasirimedhi Institute of Science and Technology, Rayong 21210, Thailand

**Keywords:** cancer drug combination, deep learning, drug discovery, graph neural networks, machine learning, multiomics, personalized medicine, proteomics, synergistic effect

## Abstract

**Motivation:**

Drug combination therapy plays a pivotal role in addressing the molecular heterogeneity of cancer, improving treatment efficacy, minimizing resistance, and reducing toxicity. Deep learning approaches have significantly advanced drug combination discovery by addressing the limitations of conventional laboratory experiments, which are time-consuming and costly. While most existing models rely on the molecular structure of drugs and gene expression data, incorporating protein-level expression provides a more accurate representation of cellular behavior and drug responses. In this study, we introduce SynProtX, an enhanced deep learning model that explicitly integrates large-scale proteomics with deep neural networks (DNNs) and the molecular structure of drugs with graph neural networks (GNNs).

**Results:**

The SynProtX-GATFP model, which combines molecular graphs and fingerprints through a graph attention network architecture, demonstrated superior predictive performance for the FRIEDMAN study dataset. We further evaluated its cell line–specific performance, which achieved accuracy across diverse tissue and study datasets. By incorporating protein expression data, the model consistently enhanced predictive performance over gene expression–only models, reflecting the functional state of cancer cells. The generalizability of SynProtX was rigorously validated using cold-start prediction, including leave-drug-combination-out, leave-drug-out, and leave-cell-line-out validation strategies, highlighting its robust performance and potential for clinical applicability. Additionally, SynProtX identified key cancer-associated proteins and molecular substructures, offering novel insights into the biological mechanisms underlying drug synergy. These findings highlight the potential of integrating large-scale proteomics and multiomics data to advance anticancer drug design and combination therapy strategies for personalized medicine.

**Availability and implementation:**  https://github.com/manbaritone/SynProtX.

## Graphical Abstract

**Graphical Abstract:**
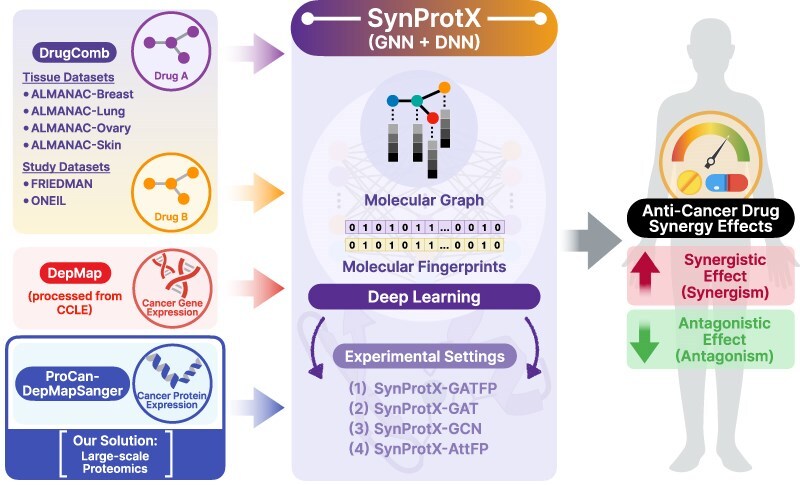
SynProtX, a deep learning model leveraging large-scale proteomics, molecular graphs, and fingerprints to enhance the prediction of the synergistic effects of anticancer drug combinations

## Background

Cancer treatment faces significant challenges, including tumor heterogeneity, which leads to resistance to monotherapy due to the different underlying genetic and molecular characteristics within each tumor. This resistance possesses the ability to bypass treatment through various mechanisms [1, 2]. Chemotherapy and targeted treatment approaches, although pivotal in treatment, come with limitations such as severe side effects and rapid development of drug resistance [3–5]. Single-agent treatments often fail to address the multiple pathways involved in cancer progression, leading to unsatisfactory therapeutic outcomes [6]. Combination therapies or multitarget approaches, which target multiple pathways, offer improved efficacy, reduced resistance, and minimized adverse effects, making them essential for optimizing patient outcomes [7]. Despite the benefits of combination therapies, prediction of effective drug combinations remains challenging. Traditional methods are labor-intensive, time-consuming, costly, and often require extensive *in vitro* and *in vivo* testing [8, 9]. Moreover, the complex interactions between drugs and their molecular targets complicate the prediction of synergistic effects, especially given the dynamic nature of cancer biology.

Computational approaches, particularly machine learning (ML) and deep learning (DL), provide promising solutions for predicting effective drug combinations by analyzing large datasets and identifying complex patterns of multiomics data, including genomics and transcriptomics [10]. By integrating multiomics data, including genomics and transcriptomics, these models provide insights into complex drug–target interactions. DL algorithms, in particular, excel in predicting drug synergy by learning from vast biological data and identifying nonlinear relationships, enhance drug discovery efficiency, and reduce experimental costs [11]. Developing ML and DL models for predicting drug synergy requires patient-specific cancer cell data. Each cancer cell possesses a unique microenvironment and multiomics information, including genomics, transcriptomics, metabolomics, epigenomics, and phosphoproteomics, which are crucial for understanding cancer behavior and treatment response [12]. Important cancer cell multiomics databases include the Cancer Cell Line Encyclopedia (CCLE), which compiles information on copy number variations, gene expression, gene mutations, and DNA methylation [13, 14]. However, the availability of multiomics data is limited, often necessitating reliance on genomic and transcriptomic data for drug response studies [15].

Currently, existing ML and DL models can predict the synergistic effects of anticancer drug combinations. Celebi et al. [16] compared multiple ML algorithms, including extreme gradient boosting (XGBoost), random forest (RF), support vector machine (SVM), Lasso, and linear regression, to classify drug combination efficacy. DeepSynergy [11], a deep neural network model, incorporates the molecular structure of drugs and gene expression data to classify and predict synergy scores for anticancer drug combinations using the Loewe additivity score. The model processes the drug structure and gene expression data separately for each drug. DeepDDS [17] extended this by combining graph convolutional networks (GCNs), graph attention networks (GATs), and multilayer perceptrons (MLPs) to jointly model molecular drug structures and gene expression profiles. AttenSyn [18] further improves predictions by employing attention mechanisms to identify the relevant molecular structure of drugs and gene expression features. Accurately predicting synergistic effects of anticancer drug combinations is critical for advancing personalized cancer therapy.

Recent advances in precision oncology emphasize the critical role of proteomic data in cancer biology. Protein expression profiling at the protein level captures functional cellular states not reflected at the genomic or transcriptomic level. Large-scale datasets, such as ProCan-DepMapSanger [19], provide protein-level expression data for 949 cancer cell lines, covering 8,498 proteins, enabling comprehensive analysis of over 8,000 proteins across diverse cancer types. Advances in high-throughput proteomics, particularly mass spectrometry, allow for comprehensive protein-level analyses, which reveal phenotypic characteristics and offer a more comprehensive understanding of cancer biology and drug responses [20]. Differential protein expression can pinpoint potential drug targets, unravel mechanisms of drug resistance, and identify protein signatures predictive of therapeutic responses, supporting personalized treatment strategies [21]. Zheng et al. [22] highlighted the superiority of proteomics in single drug response prediction tasks compared to transcriptomics using graph neural networks, emphasizing proteomics’ enhanced ability to reflect the downstream functional consequences of genomic alterations, directly influencing protein functions and therapeutic responses. Further advancing this approach, Cai et al. [23] developed DeePathNet, a transformer-based deep learning model integrating multiomic data with cancer-specific pathways, demonstrating that the incorporation of pathway-level insights and proteomic profiles based on ProCan-DepMapSanger significantly enhances predictive accuracy and interpretability in single drug response. DeePathNet explicitly leveraged proteomic data to identify novel biomarkers and therapeutic targets, marking significant progress in cancer omic integration and predictive modeling. Protein expression data thus offers crucial insights to understand cancer drug responses, as proteins can interact with drugs and various biomolecules, reflecting the disease’s pathways in living organisms [24, 25]. Despite these advancements, the explicit integration of proteomic data into ML/DL models for predicting cancer drug combinations remains relatively unexplored. This leads us to address this gap and answer an important question: Can large-scale proteomics data, combined with molecular graphs and fingerprints, improve model performance compared to traditional gene expression data alone?

Building upon these recent advances and in response to the critical gap and question, we introduce SynProtX, a computational DL framework that explicitly integrates comprehensive proteomic datasets (ProCan-DepMapSanger and CCLE) with molecular fingerprints and structural information through a graph attention network. Unlike existing computational models, SynProtX uniquely leverages proteomic profiles to advance drug combination prediction accuracy, interpretability, and clinical relevance beyond existing genomic- and transcriptomic-based models. SynProtX utilizes data from the DrugComb database [26, 27], CCLE [13, 14], and ProCan-DepMapSanger [19] to predict the synergistic effects of drug combinations. We employed GCNs [28], graph attention networks (GATs) [29], Attentive FP [30], and graph attention networks combined with molecular fingerprint (GATFP) to compare the learning processes of the molecular structure of drugs with gene and protein expression data. Our findings demonstrate that SynProtX-GATFP, which combines molecular graphs and fingerprints through a GAT coupled with DNN, provides robust predictive performance in the FRIEDMAN study [31]. Cell line–specific evaluations further show that the model achieves accuracy across various tissue and study datasets. Ablation studies reveal that incorporating protein-level expression consistently improves predictive performance compared to models based solely on gene expression. To assess generalizability, we validated SynProtX using cold-start scenarios, including leave-drug-combination-out, leave-drug-out, and leave-cell-line-out validation strategies, highlighting its robust performance and potential for clinical applicability. Additionally, SynProtX identifies cancer-associated proteins and molecular substructures of drugs, offering insights into the biological mechanisms underlying drug synergy. By leveraging proteomics, SynProtX improves the accuracy of synergy predictions and contributes to advancing anticancer drug discovery for personalized medicine.

Our contributions are summarized as follows:

- We introduced SynProtX, a DL model for predicting anticancer drug synergy utilizing new large-scale proteomics data, which aids in discovering effective anticancer drug combinations.
- We developed SynProtX, which explicitly integrates comprehensive proteomic datasets from the CCLE and ProCan-DepMapSanger databases and graph neural networks for the molecular structure of drugs, thereby enhancing the accuracy of synergy predictions.
- We designed SynProtX to visualize the importance of each molecular substructure in synergistic effects by incorporating attention mechanisms within the model architecture, allowing researchers to better understand and interpret the model’s predictions.
- We evaluated the model performance at the cell line–specific level and conducted ablation studies to assess the impact of protein- and gene-level features on predictive accuracy.
- We validated the performance of SynProtX using the cold-start prediction, including leave-drug-combination-out, leave-drug-out, and leave-cell-line-out validation strategies, to demonstrate its robustness and generalizability.

## Problem Formulation

The SynProtX includes both regression and classification models. The regression model aims to predict the synergy score of drug combinations, whereas classification model aims to classify the synergism of drug combinations. The models use the drug combinations, gene expression, and protein expression data to calculate the predicted synergy score ${y}_c$ for each drug combination. The predicted score, ${y}_c( {A,B,g,p} )\epsilon \mathbb{R}$, depends on the drug combination $\langle {A,B} \rangle $, the gene expression level *g*, and the protein expression level *p*.

For the calculation of the synergistic score ${S}_{\textit{Loewe}}$, it is obtained from drug *A* with concentration ${x}_1$, which has an efficacy value of ${y}_1$, and drug *B* with concentration ${x}_2$, which has an efficacy value of ${y}_2$. For the classification task, Loewe scores [32] that are positive (${S}_{\textit{Loewe}}$ > 0) indicate that the drug pair is considered synergistic, whereas for negative (${S}_{\textit{Loewe}}$ < 0) scores, the drug pair is considered antagonistic. Loewe scores can be calculated using the following formula [33]:


(1) \begin{eqnarray*}
{S}_{\textit{Loewe}} = {y}_c - {y}_1\left( {{x}_1 + {x}_2} \right) = {y}_c - {y}_2\left( {{x}_1 + {x}_2} \right)
\end{eqnarray*}

The dataset includes drug pairs *i* and *j* that have been modified to form molecular graphs ${M}_A$ and ${M}_B$, respectively. The gene expression level *g* and protein expression level *p* are transformed into the vectors ${v}_g$ and ${v}_p$, respectively. The drug combination (${M}_A$ and ${M}_B$), vector of gene expression (${v}_g$), and vector of protein expression (${v}_p$) data are used for synergy prediction.

## Model Architecture

**Figure 1:**
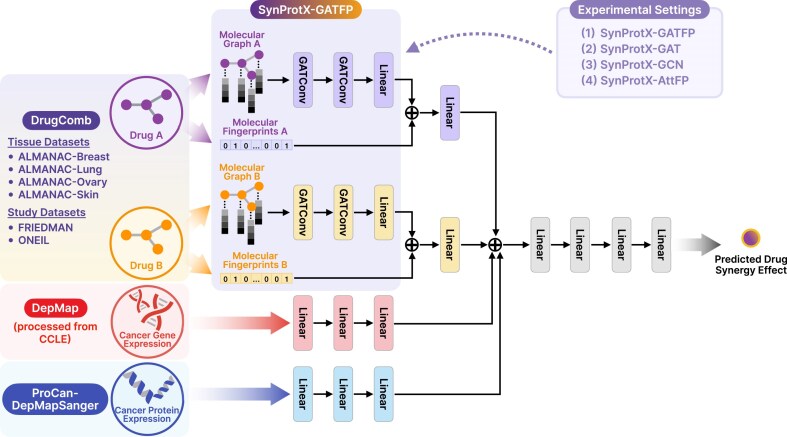
Overview of the SynProtX model architecture based on the SynProtX-GATFP experimental setting for predicting the synergistic effects of anticancer drug combinations. The model leverages data from DrugComb, including the tissue datasets: (1) ALMANAC-Breast, (2) ALMANAC-Lung, (3) ALMANAC-Ovary, and (4) ALMANAC-Skin datasets and study datasets: (1) FRIEDMAN and (2) ONEIL. The model incorporates gene and protein expressions from DepMap (processed from CCLE) and ProCan-DepMapSanger, respectively. The model achieves deep feature extraction based on different experimental settings (SynProtX-GATFP, SynProtX-GAT, SynProtX-GCN, and SynProtX-AttFP) to enhance representation learning for the molecular structure of drug combinations.

The architecture of the developed predictive model, designed to explicitly integrate large-scale proteomics data for enhancing the efficacy of combination drug therapy in cancer treatment, is illustrated in Fig. 1. Each drug combination includes 2 molecular graphs of individual drugs (drug A and drug B) that enter the first layer along with gene expression and protein expression data. Gene and protein expression data from cancer cells are learned through deep neural network layers. The resulting embedding vectors of the drug combination, gene expression, and protein expression are concatenated to form a unified feature vector. This vector is subsequently passed through additional layers of the deep neural network for prediction.

The proposed SynProtX-GATFP model integrates multiple data types, including molecular graphs, molecular fingerprints, gene expression, and protein expression data, to predict drug synergy effects. The following mathematical formulation outlines the model architecture and its components.

### Input representation

1. **Molecular Graphs**: Each drug *i* is represented by its molecular graph ${M}_i\ = \ ( {{V}_i,\ {E}_i} )$, where ${V}_i$ denotes the set of nodes (atoms) and ${E}_i$ denotes the set of edges (bonds).
2. **Molecular Fingerprints**: Each drug *i* is represented by a binary vector of molecular fingerprints ${F}_i\epsilon\ {\{ {0,\ 1} \}}^d$, where *d* is the dimensionality of the fingerprint vector.
3. **Gene Expression**: The gene expression level for cancer cells is represented $by\ {G}_{exp}\epsilon{\mathbb{R}}^n$, where *n* is the number of genes.
4. **Protein Expression**: The protein expression level for cancer cells is represented by ${P}_{exp}\epsilon{\mathbb{R}}^m,$, where *m* is the number of proteins.

### Graph neural network (GNN)

For each drug *i*, the molecular graph ${M}_i$ is processed using GAT to generate node embeddings. Let $H_i^0$ be the initial node feature matrix (generated atomic features).


(2) \begin{eqnarray*}
H_i^{\left( {l + 1} \right)}{\mathrm{\ }} = {\mathrm{\ }}\textit{GATLayer}\left( {H_i^{\left( l \right)},{\mathrm{\ }}{E}_i} \right)
\end{eqnarray*}

where $H_i^l$ represents the node features at the *l*th layer, and $GATLayer$ applies the graph attention mechanism to update the node embeddings. The whole architecture of the $GATLayer$, along with other used architectures, is shown in the Molecular Graph and Deep Neural Networks subsection of the Methods.

The final node embeddings are then pooled to produce a graph-level embedding:


(3) \begin{eqnarray*}
{z}_i{\mathrm{\ }} = {\mathrm{\ }}\textit{GlobalPooling}\left( {H_i^L} \right)
\end{eqnarray*}

### Linear transformations

The molecular fingerprint ${F}_i$, gene expression ${G}_{exp}$, and protein expression ${P}_{exp}$ data are transformed through linear layers as follows:


(4) \begin{eqnarray*}
{f}_i{\mathrm{\ }} = {\mathrm{\ }}{W}_f{\mathrm{\ }}{F}_i{\mathrm{\ }} + {\mathrm{\ }}{b}_f
\end{eqnarray*}

(5) \begin{eqnarray*}
{g}_{exp}{\mathrm{\ }} = {\mathrm{\ }}{W}_g{\mathrm{\ }}{G}_{exp}{\mathrm{\ }} + {\mathrm{\ }}{b}_g
\end{eqnarray*}

(6) \begin{eqnarray*}
{p}_{exp}{\mathrm{\ }} = {\mathrm{\ }}{W}_p{P}_{exp}{\mathrm{\ }} + {\mathrm{\ }}{b}_p
\end{eqnarray*}

where ${W}_f$, ${W}_g$, and ${W}_p$ are weight matrices and ${b}_f$, ${b}_g$, and ${b}_p$ are biased terms.

### Features fusion

For each drug combination $( {A,B} )$, the embeddings from the GNN and linear transformations are concatenated and processed through additional linear layers to integrate the information:


(7) \begin{eqnarray*}
{h}_A{\mathrm{\ }} = \textit{Concat}\left( {{z}_A,{\mathrm{\ }}{f}_A} \right)
\end{eqnarray*}

(8) \begin{eqnarray*}
{h}_B{\mathrm{\ }} = \textit{Concat}\left( {{z}_B,{\mathrm{\ }}{f}_B} \right)
\end{eqnarray*}

### Interaction modeling

The interaction between the 2 drugs is modeled by combining their embeddings:


(9) \begin{eqnarray*}
{h}_{AB}{\mathrm{\ }} = {\mathrm{\ }}\textit{Concat}\left( {{h}_A,{\mathrm{\ }}{h}_B,{g}_{exp},{p}_{exp}} \right)
\end{eqnarray*}

This combined representation is passed through a series of fully connected layers to predict the drug synergy effect.


(10) \begin{eqnarray*}
y{\mathrm{\ }} = \sigma \left( {{W}_3{\mathrm{\ }}\left( {\sigma \left( {{W}_2{\mathrm{\ }}\left( {\sigma \left( {{W}_1{\mathrm{\ }}{h}_{AB}{\mathrm{\ }} + {\mathrm{\ }}{b}_1} \right)} \right){\mathrm{\ }} + {\mathrm{\ }}{b}_2} \right)} \right){\mathrm{\ }} + {\mathrm{\ }}{b}_3} \right)
\end{eqnarray*}

(11) \begin{eqnarray*}
p{\mathrm{\ }} = \textit{sigmoid}\left( {{W}_3{\mathrm{\ }}\left( {\sigma \left( {{W}_2{\mathrm{\ }}\left( {\sigma \left( {{W}_1{\mathrm{\ }}{h}_{AB}{\mathrm{\ }} + {\mathrm{\ }}{b}_1} \right)} \right){\mathrm{\ }} + {\mathrm{\ }}{b}_2} \right)} \right){\mathrm{\ }} + {\mathrm{\ }}{b}_3} \right)
\end{eqnarray*}

where ${W}_1$, ${W}_2$, and ${W}_3$ are weight matrices; ${b}_1$, ${b}_2$, and ${b}_3$ are bias terms; and $\sigma $ denotes a nonlinear activation function (e.g., ReLU).

### Output

The final output is *y*, the scalar value representing the predicted synergy score of the drug combination $( {A,B} )$, or *p*, and the predicted probability of the drug combination $( {A,B} )$ being synergistic.

## Results and Discussion

### Protein and gene expression–assisted SynProtX development

**Figure 2:**
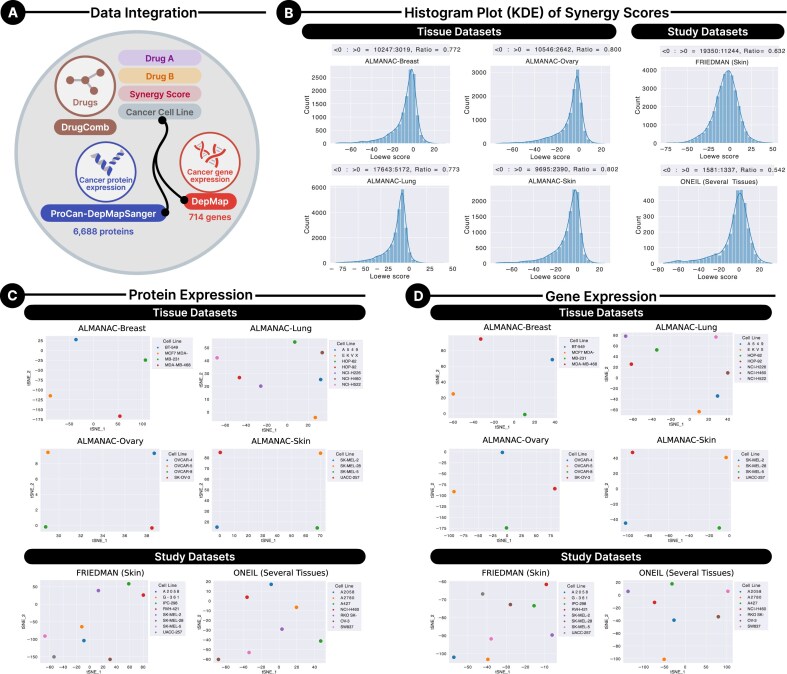
The data integration process and dataset analysis. (A) The process begins with the DrugComb database, which provides drug combination data comprising drug A, drug B, the synergy score (Loewe score), and the corresponding cancer cell line. These combinations are integrated with 6,688 protein expression levels from the ProCan-DepMapSanger dataset and 714 gene expression levels from the DepMap database (processed from the CCLE database). (B) Distribution of Loewe synergy scores following data preprocessing across the tissue datasets: (1) ALMANAC-Breast, (2) ALMANAC-Lung, (3) ALMANAC-Ovary, and (4) ALMANAC-Skin and the study datasets: (1) FRIEDMAN and (2) ONEIL. (C) The t-distributed stochastic neighbor embedding (t-SNE) visualization of cell line embeddings based on protein expression across the across the aforementioned tissue and study datasets. (D) The t-SNE visualization of cell line embeddings based on gene expression across the same datasets.

Integrating protein and gene expression data is crucial for enhancing the predictive performance of SynProtX. The process commenced with the extraction of drug combination data from the DrugComb database, encompassing datasets such as ALMANAC-Breast, ALMANAC-Lung, ALMANAC-Ovary, ALMANAC-Skin, FRIEDMAN, and ONEIL (Fig. 2A). Each entry consisted of a drug pair (drug A and drug B), a corresponding synergy score (Loewe score), and the associated cancer cell line. To further enrich these data, protein expression data from the ProCan-DepMapSanger database and gene expression data from the DepMap database, processed from the CCLE, were incorporated. After filtering and ensuring consistency, a total of 94,862 drug combination experiments were identified, as detailed in Supplementary Table S1. This integrated dataset offered a comprehensive and robust foundation for training and evaluating the model.

Notably, each drug pair was not uniformly tested across all cancer cell types, reflecting the inherent variability and context-specific nature of the data. It is also important to distinguish that while the ALMANAC-Breast, ALMANAC-Lung, ALMANAC-Ovary, ALMANAC-Skin, and FRIEDMAN datasets are derived from single tissue types, the ONEIL dataset spans multiple tissues, including skin, lung, ovary, and large intestine. This diversity in tissue types mitigates potential issues arising from cell line redundancy, which could otherwise introduce bias in model predictions. By generating sub-datasets with varying tissue types and studies, we ensure a fair evaluation of the model’s performance, as redundancy could lead to overfitting and inaccurate generalization.

The integration of multimodal data, including protein and gene expression data, enabled SynProtX to capture the complex molecular signatures underlying drug responses across diverse cancer cell lines. The synergy scores from DrugComb served as target variables, indicating the efficacy of each drug combination. Protein expression data provided insights into the proteomic landscape, while gene expression data offered a view of transcriptomic variations across cancer cell lines [34–37]. Together, these layers of biological information allowed the model to infer cellular functional states and regulatory mechanisms critical for accurate prediction of drug synergy.

The distribution of synergy scores (Loewe scores) across the ALMANAC-Breast, ALMANAC-Lung, ALMANAC-Ovary, ALMANAC-Skin, FRIEDMAN, and ONEIL datasets was analyzed after data preprocessing (Fig. 2B). The varying distributions and skewness of these Loewe scores across different datasets provide initial insights into how drug combinations interact within various cancer cell lines. This variability underscores the complexity of predicting drug synergy and highlights the importance of using advanced models to capture these effects accurately. To further explore the underlying structure of the integrated data, t-distributed stochastic neighbor embedding (t-SNE) [38] visualizations were employed. The t-SNE analysis of cell line embeddings based on protein level (Fig. 2C) and gene expression (Fig. 2D) revealed distinct clustering patterns, reflecting the unique molecular characteristics of each cancer cell line. These clusters suggest that the model can effectively distinguish between different cancer cell lines and tissue types, which is essential for generalizing across various biological settings and making accurate drug synergy predictions. Separating clusters in the t-SNE plots indicates that the model can leverage these data to identify unique expression profiles that correlate with synergistic drug effects. The integration of protein and gene expression data allows the SynProtX model to account for the cellular environment and response to external stimuli and the transcriptional landscape within the cancer cells. By incorporating these data types, SynProtX can be used to predict how different drug combinations interact within specific cellular contexts, offering a more comprehensive and accurate tool for identifying synergistic drug combinations. This multiomics approach enhances the ability of the model to capture the intricate interplay between proteomic and transcriptomic factors that influence drug response [39, 40], ultimately leading to more reliable predictions and insights into the mechanisms of drug synergy.

### SynProtX elevates the model’s predictive performance on regression and classification tasks

**Figure 3:**
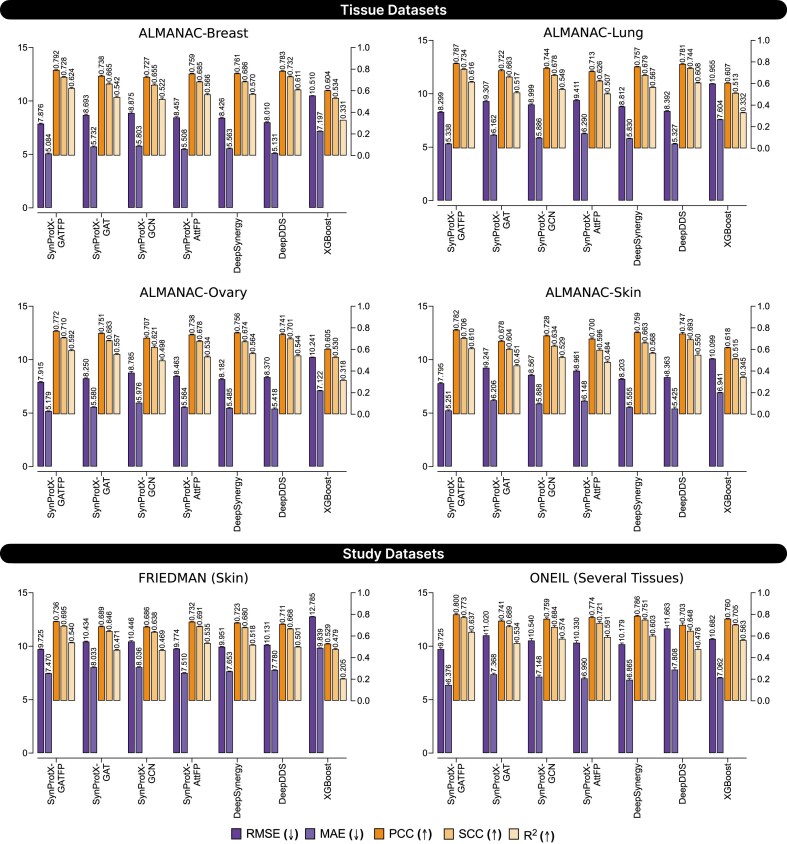
The predictive performance on regression tasks across the tissue datasets: (1) ALMANAC-Breast, (2) ALMANAC-Lung, (3) ALMANAC-Ovary, and (4) ALMANAC-Skin and the study datasets: (1) FRIEDMAN and (2) ONEIL with different training models.

The SynProtX-GATFP model consistently demonstrated improvements in predictive performance across both regression and classification tasks for tissue and study datasets. In the regression task, SynProtX-GATFP achieved the lowest Root Mean Square Error (RMSE) and mean absolute error (MAE), along with the highest Pearson correlation coefficient (PCC) and *R*² scores across all ALMANAC tissue datasets (Fig. 3 and Supplementary Table S2 and Fig. S1). Specifically, it achieved RMSE values of 7.876, 8.299, 7.915, and 7.795 for the Breast, Lung, Ovary, and Skin datasets, respectively, along with corresponding PCC values ranging from 0.772 to 0.792. These results underscore the model’s capability to accurately capture synergy patterns in tissue-specific contexts. Compared to other SynProtX variants (GAT, GCN, AttFP) and traditional baselines such as DeepSynergy, DeepDDS, and XGBoost, SynProtX-GATFP consistently ranked highest across nearly all regression metrics. This trend continued in the study datasets, with SynProtX-GATFP achieving the best regression performance in both FRIEDMAN (RMSE: 9.725 and PCC: 0.736) and ONEIL (RMSE: 9.725 and PCC: 0.800) datasets.

**Figure 4:**
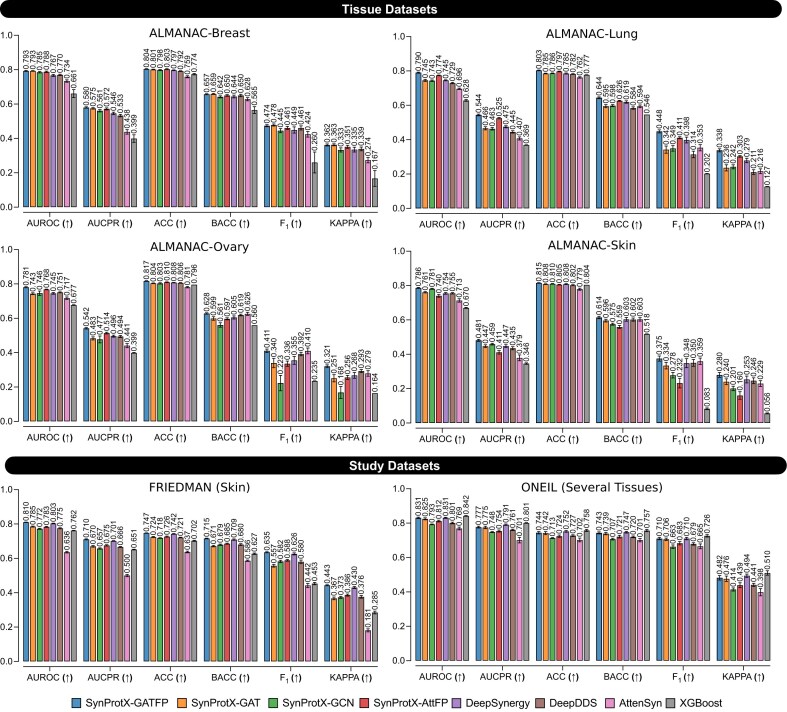
The predictive performance on classification tasks across the tissue datasets: (1) ALMANAC-Breast, (2) ALMANAC-Lung, (3) ALMANAC-Ovary, and (4) ALMANAC-Skin and the study datasets: (1) FRIEDMAN and (2) ONEIL with different training models.

In classification tasks, SynProtX-GATFP also outperformed most models across all individual tissue-specific datasets (Fig. 4 and Supplementary Table S3 and Supplementary Figs. S2 and S3). It attained the highest area under the receiver operating characteristic curve (AUROC) in ALMANAC-Breast (0.793), Lung (0.790), Ovary (0.781), and Skin (0.786), along with top area under the precision-recall curve (AUCPR), F_1_, and kappa scores, highlighting its effectiveness in both balanced and imbalanced settings. In the FRIEDMAN dataset, it again led across all metrics (AUROC: 0.810, AUCPR: 0.710, F_1_: 0.635, kappa: 0.443), confirming its consistent classification capability. However, in the ONEIL dataset, although SynProtX-GATFP achieved strong classification performance (AUROC: 0.831, AUCPR: 0.777, F_1_: 0.710, kappa: 0.482), it was marginally outperformed by XGBoost, which recorded the highest AUROC (0.842) and AUCPR (0.801). This exception suggests that while SynProtX-GATFP is highly generalizable, classical ensemble-based methods like XGBoost may still retain an edge in certain classification scenarios with smaller, multitissue datasets.

**Figure 5:**
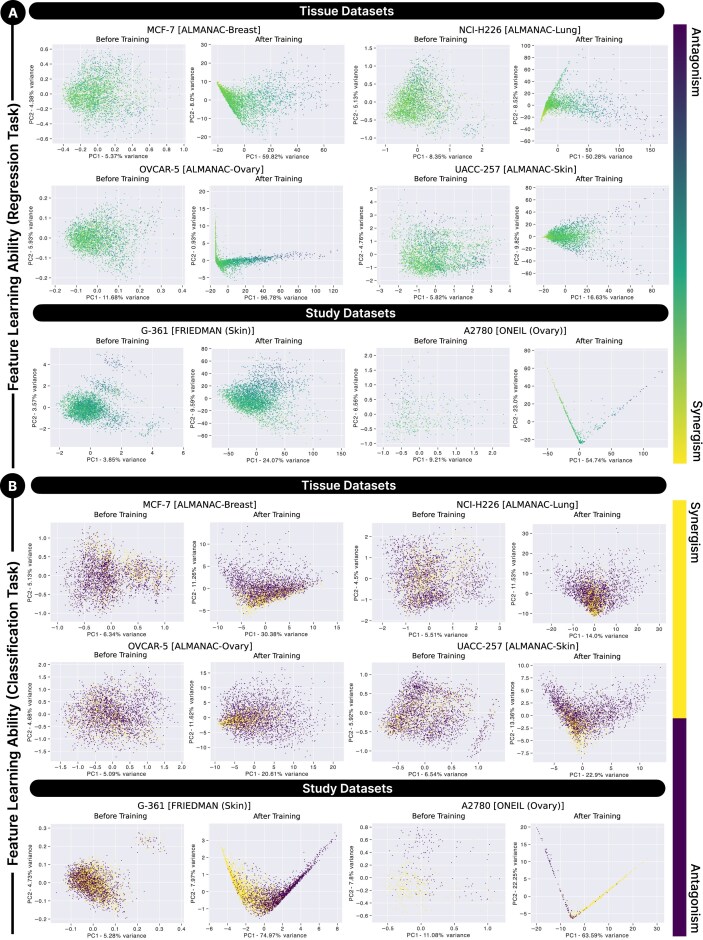
PCA visualization of the feature space distribution of drug combinations on specific cell line for the SynProtX-GATFP model in (A) the regression task and (B) the classification task, shown before and after the training process.

The principal component analysis (PCA) analysis provided further insights into the predictive performance of SynProtX by visualizing the feature space distribution of drug combinations for each cell line before and after training (Fig. 5). The PCA plots for the regression task highlighted the capacity of SynProtX to closely align the predicted synergy scores with the ground truth. The plots show improved feature space alignment after training, indicating that the model successfully learned the complex relationships between the input features and synergy scores (Fig. 5A). For the classification task, PCA visualizations showed that the SynProtX-GATFP model effectively separated different classes, demonstrating its ability to accurately capture the underlying structure of the data. This effective separation suggests that SynProtX can identify and utilize the most relevant features to distinguish between synergistic and nonsynergistic drug combinations (Fig. 5B). This alignment is crucial to contribute reliable predictions across diverse biological settings and underscores capability of SynProtX to generalize effectively across different datasets. In summary, SynProtX significantly improves the predictive performance of classification and regression tasks across various datasets, consistently outperforming other state-of-the-art models. These improvements are particularly evident in the enhanced accuracy and robustness of predictions supported by PCA analysis, which highlights the ability of the model to effectively capture and utilize complex biological data.

### Molecular fingerprints enhance feature representation and model generalization

The incorporation of molecular fingerprints into the SynProtX model significantly enhances its feature representation and generalization capabilities, thereby improving the predictive performance across various datasets. Integrating molecular fingerprints into SynProtX-GATFP resulted in marked improvements in the predictive performance over SynProtX-GAT across all datasets. In regression tasks, SynProtX-GATFP also demonstrated superior performance, achieving lower RMSE and MAE values while obtaining higher PCC, SCC, and *R*^2^ scores than SynProtX-GAT. These improvements were statistically significant, with PCC increasing by up to 10.4% and *R*^2^ increasing by up to 15.9% (Fig. 3 and Supplementary Table S2). In the classification tasks, SynProtX-GATFP consistently outperformed SynProtX-GAT, achieving higher AUROC, AUCPR, accuracy, balanced accuracy, F_1_, and Cohen’s kappa values. For example, in the FRIEDMAN dataset, SynProtX-GATFP showed a 2.50% improvement in AUROC and a 2.30% enhancement in accuracy compared to SynProtX-GAT (Fig. 4 and Supplementary Table S3). Similar trends were observed across the ALMANAC-Breast, ALMANAC-Lung, ALMANAC-Ovary, and ALMANAC-Skin datasets, in which SynProtX-GATFP exhibited improvements across various metrics. These results underscore the significant impact of molecular fingerprints on the ability of the model to accurately predict drug synergy effects, reflecting the added value of this feature in capturing the chemical characteristics of drug combinations. Moreover, these improvements highlight the model’s enhanced ability to predict synergy scores with greater accuracy and reliability, thereby validating the effectiveness of molecular fingerprints in improving the regression task performance.

The comparison of the distribution of the ground truth and predicted synergy scores further supports the enhanced feature representation and generalization achieved by SynProtX-GATFP. The violin plots in Supplementary Fig. S4 illustrate how SynProtX-GATFP’s predictions align more closely with the ground-truth distributions across all datasets than SynProtX-GAT. In the ALMANAC-Breast dataset, for example, SynProtX-GATFP displayed narrower and more centered distributions around the ground truth, indicating higher consistency and reduced prediction variance. This alignment is crucial to ensure that the model generalizes well across different datasets and accurately captures the underlying biological mechanisms that drive drug synergy. These results suggest that the molecular fingerprints allowed the model to better capture the underlying relationships between the chemical structures of drug and their synergistic effects, leading to more precise predictions across different cancer types. The consistent performance improvements across both tasks highlight the importance of molecular fingerprints in enhancing the feature representation within the SynProtX-GATFP model, thereby improving its ability to generalize across various datasets. This enhanced generalization is critical for drug discovery, in which accurate predictions across different biological contexts and chemical spaces are essential. Overall, the inclusion of molecular fingerprints significantly strengthened the capabilities of SynProtX-GATFP, making it a more robust and reliable tool for predicting drug synergy in diverse cancer datasets.

### SynProtX-GATFP affirms the performance on cell-specific responses

**Figure 6:**
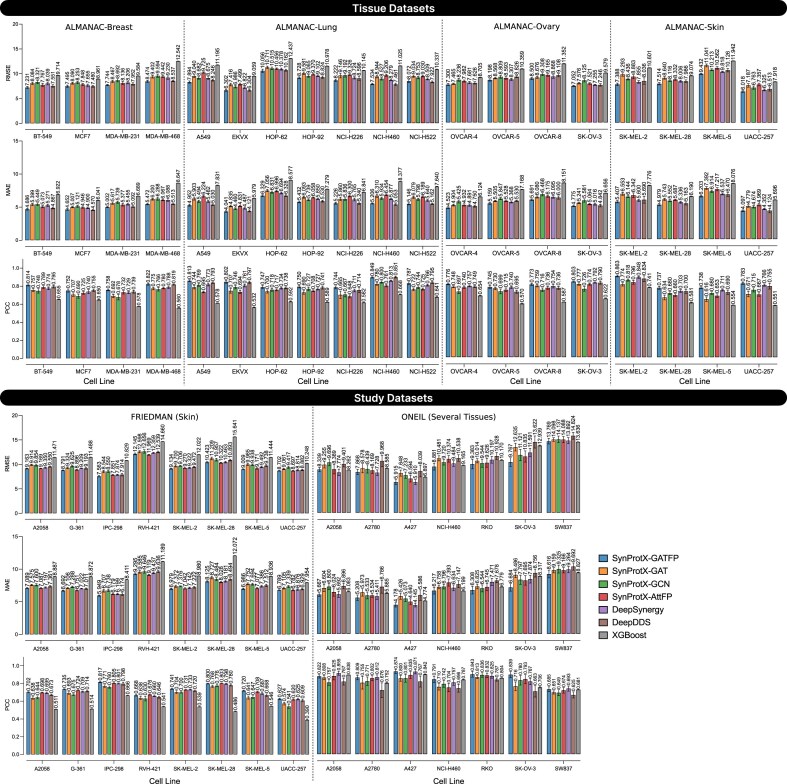
The predictive cell line–specific performance on regression tasks across the tissue datasets: (1) ALMANAC-Breast, (2) ALMANAC-Lung, (3) ALMANAC-Ovary, and (4) ALMANAC-Skin and the study datasets: (1) FRIEDMAN and (2) ONEIL with different training models.

The SynProtX-GATFP model demonstrated strong performance across both regression and classification tasks at the individual cancer cell line level, reinforcing its robustness in capturing fine-grained multiomics and drug-related features. For regression tasks, SynProtX-GATFP achieved the lowest RMSE and MAE, along with the highest PCC, across most of the cell lines in both tissue and study datasets (Fig. 6 and Supplementary Tables S4–S9). In the ALMANAC-Breast dataset, SynProtX-GATFP outperformed competing models across all 3 cell lines: BT-549, MDA-MB-231, and MDA-MB-468, with RMSEs ranging from 7.231 to 8.474 and PCCs from 0.758 to 0.822, notably higher than those of DeepSynergy, DeepDDS, and XGBoost. Similar trends were observed in ALMANAC-Lung, where SynProtX-GATFP led in 5 of 7 cell lines, achieving the highest PCC and lowest RMSE, including A549, EKVX, HOP-62, HOP-92, and NCI-H226. The model also delivered consistently accurate predictions across all cell lines in the ALMANAC-Ovary and ALMANAC-Skin datasets. In the FRIEDMAN dataset, which includes 8 skin cancer cell lines, SynProtX-GATFP led across nearly all regression metrics. For instance, in the IPC-298 cell line, it achieved a PCC of 0.817, outperforming DeepSynergy (0.801) and XGBoost (0.666), while also attaining the lowest RMSE (7.563) and MAE (5.949). Even in the heterogeneous ONEIL dataset, which spans cell lines from multiple tissue origins such as lung, ovary, skin, and colon, SynProtX-GATFP maintained top-tier performance in most cases, including A427 (PCC: 0.874) and SK-OV-3 (PCC: 0.839), further demonstrating its cross-tissue generalizability.

**Figure 7:**
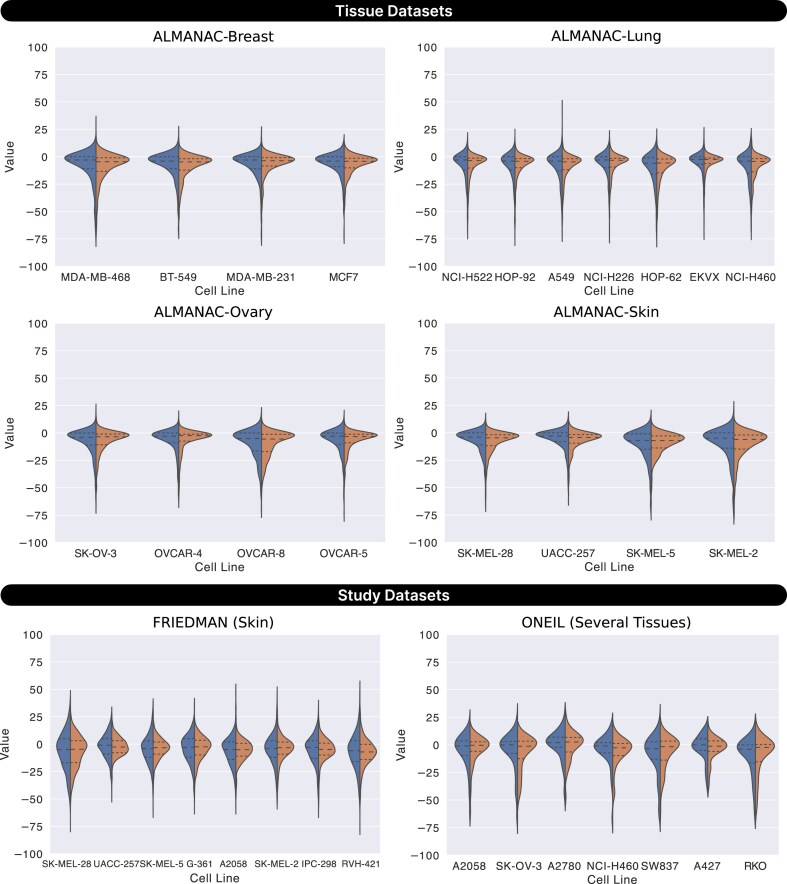
Comparison of distribution of ground truth and prediction synergy scores on a test set across cell lines based on SynProtX-GATFP for the tissue datasets: (1) ALMANAC-Breast, (2) ALMANAC-Lung, (3) ALMANAC-Ovary, and (4) ALMANAC-Skin and the study datasets: (1) FRIEDMAN and (2) ONEIL.

Figure 7 presents violin plots of predicted synergy scores across individual cell lines in both tissue and study datasets. SynProtX-GATFP showed tight prediction distributions with centered medians across nearly all cell lines, especially in ALMANAC-Breast and ALMANAC-Lung, indicating a strong alignment between predicted and true synergy values. Similar consistency was observed in ALMANAC-Ovary, ALMANAC-Skin, and FRIEDMAN datasets, reflecting the model’s ability to capture synergy patterns in diverse biological settings. Even within the more complex ONEIL dataset, the model preserved stable predictive behavior across multiple tissue types. The density and shape of the violin plots further suggest that SynProtX-GATFP effectively learned nuanced, cell-specific patterns rather than overfitting to global trends. These results collectively support the model’s capacity to generalize across both homogeneous and heterogeneous cellular contexts.

**Figure 8:**
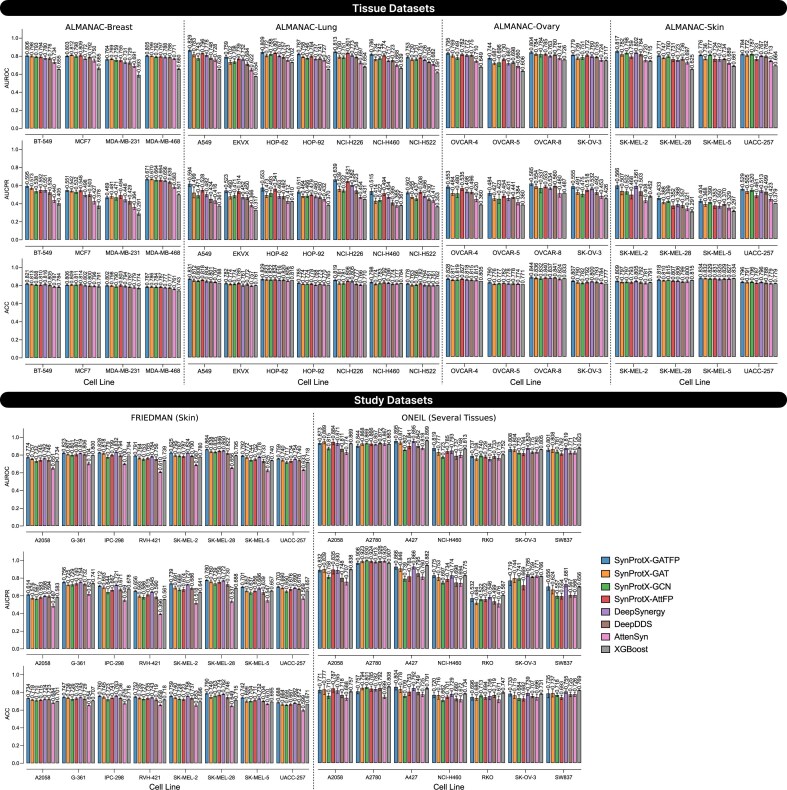
The predictive cell line–specific performance on classification tasks across the tissue datasets: (1) ALMANAC-Breast, (2) ALMANAC-Lung, (3) ALMANAC-Ovary, and (4) ALMANAC-Skin and the study datasets: (1) FRIEDMAN and (2) ONEIL with different training models.

In the classification task, SynProtX-GATFP also maintained high performance across key evaluation metrics, including AUROC, AUCPR, F_1_, precision, and recall, across most cell lines (Fig. 8 and Supplementary Tables S10–S15). In the ALMANAC-Breast dataset, it achieved AUROC values exceeding 0.8 for BT-549 and MDA-MB-468, outperforming classical machine learning baselines such as XGBoost, as well as several state-of-the-art (SOTA) deep learning models. The model also recorded top AUROC values in lung, ovary, and skin cell lines, including challenging examples such as SK-MEL-5. Within the more heterogeneous FRIEDMAN and ONEIL datasets, SynProtX-GATFP remained highly competitive, though it was marginally outperformed by XGBoost in select cases, suggesting the potential benefit of ensemble-based approaches. Overall, these findings underscore that the integration of proteomic and transcriptomic data enhances the model’s ability to capture functional heterogeneity in drug responses at the cellular level. The cell-specific insights derived from SynProtX-GATFP reinforce its promise for personalized drug combination therapy and precision oncology applications.

Despite its strong overall performance, SynProtX-GATFP exhibited variability across certain cell lines, particularly within the diverse ONEIL dataset. For instance, while it performed well in A427 and SK-OV-3, the model’s predictions were less centered and more dispersed in challenging cases such as RKO and SW837 (Fig. 7), indicating potential difficulties in capturing synergy behavior in cell lines with limited training data or greater biological complexity. These observations highlight areas for future improvement, such as enhancing data diversity, incorporating dynamic proteomic responses, and refining strategies for underrepresented cell types.

### Protein level improves model predictive performance for SynProtX-GATFP

To quantify the contribution of protein-level expression data to predictive modeling, we conducted ablation studies comparing SynProtX-GATFP variants in which either protein-level or gene expression features were excluded. The results, summarized in Table 1 for regression task and Table 2 for classification task, demonstrate that the inclusion of protein-level features substantially enhances model performance across both tissue and study datasets. In regression tasks, the exclusion of gene expression features consistently led to lower RMSE and higher correlation metrics (PCC and SCC) compared to models without protein-level data. For example, in the ALMANAC-Breast dataset, excluding gene expression improved RMSE from 8.064 to 7.960 and increased PCC from 0.781 to 0.786, indicating that protein features alone capture functionally relevant variance. Similar trends were observed in ALMANAC-Lung, Ovary, and Skin datasets, suggesting that protein-level data contribute robustly to prediction accuracy. This effect was even more pronounced in the FRIEDMAN and ONEIL datasets. In ONEIL, the model with only protein-level data achieved a PCC of 0.801 compared to 0.728 without protein data, as well as a 10.8% increase in *R*², underscoring the generalization strength of protein features in heterogeneous, multitissue contexts. In classification tasks, models that retained protein-level features consistently outperformed those lacking them across all evaluation metrics, including AUROC, AUCPR, F_1_ score, and Cohen’s kappa. For instance, in ALMANAC-Lung, removing gene expression improved AUROC to 0.794 compared to 0.768 when protein data were excluded, a 2.6% gain. Similar improvements were seen in the FRIEDMAN and ONEIL datasets, confirming that protein-level features significantly enhance the model’s ability to distinguish synergistic versus nonsynergistic drug combinations.

**Table 1:** The predictive performance on the regression task of ablation studies between protein level and gene expression across the tissue datasets: (1) ALMANAC-Breast, (2) ALMANAC-Lung, (3) ALMANAC-Ovary, and (4) ALMANAC-Skin and the study datasets: (1) FRIEDMAN and (2) ONEIL

		Regression metrics				
Dataset	Ablation study	RMSE (↓)	MAE (↓)	PCC (↑)	SCC (↑)	R^2^ (↑)
**Tissue Datasets**						
ALMANAC-Breast	w/o Prot Level	8.064 ± 0.068	5.182 ± 0.035	0.781 ± 0.004	0.719 ± 0.003	0.606 ± 0.007
	w/o Gene Exp	7.960 ± 0.083	5.140 ± 0.067	0.786 ± 0.005	0.720 ± 0.006	0.616 ± 0.008
ALMANAC-Lung	w/o Prot Level	8.735 ± 0.076	5.624 ± 0.045	0.761 ± 0.005	0.709 ± 0.004	0.575 ± 0.007
	w/o Gene Exp	8.428 ± 0.055	5.444 ± 0.032	0.781 ± 0.003	0.726 ± 0.005	0.604 ± 0.005
ALMANAC-Ovary	w/o Prot Level	8.023 ± 0.042	5.203 ± 0.026	0.766 ± 0.003	0.709 ± 0.003	0.581 ± 0.004
	w/o Gene Exp	7.820 ± 0.082	5.096 ± 0.059	0.778 ± 0.005	0.719 ± 0.006	0.602 ± 0.008
ALMANAC-Skin	w/o Prot Level	8.095 ± 0.100	5.333 ± 0.083	0.763 ± 0.007	0.694 ± 0.008	0.579 ± 0.010
	w/o Gene Exp	7.740 ± 0.072	5.236 ± 0.038	0.785 ± 0.005	0.707 ± 0.004	0.615 ± 0.007
**Study Datasets**						
FRIEDMAN (skin)	w/o Prot Level	10.239 ± 0.082	7.888 ± 0.065	0.701 ± 0.006	0.656 ± 0.006	0.490 ± 0.008
	w/o Gene Exp	9.717 ± 0.020	7.438 ± 0.016	0.737 ± 0.001	0.695 ± 0.002	0.541 ± 0.002
ONEIL (several tissues)	w/o Prot Level	11.084 ± 0.114	7.468 ± 0.074	0.728 ± 0.007	0.672 ± 0.007	0.529 ± 0.010
	w/o Gene Exp	9.734 ± 0.138	6.429 ± 0.098	0.801 ± 0.006	0.769 ± 0.008	0.637 ± 0.010

*Note:* w/o Prot Level denotes exclusion of the protein-level dat,a and w/o Gene Exp denotes exclusion of the gene expression data.

**Table 2:** The predictive performance on the classification task of ablation studies between protein level and gene expression across the tissue datasets: (1) ALMANAC-Breast, (2) ALMANAC-Lung, (3) ALMANAC-Ovary, and (4) ALMANAC-Skin and the study datasets: (1) FRIEDMAN and (2) ONEIL

		Classification metrics					
Dataset	Ablation study	AUROC (↑)	AUCPR (↑)	ACC (↑)	BACC (↑)	F_1_ (↑)	KAPPA (↑)
**Tissue Datasets**							
ALMANAC-Breast	w/o Prot Level	0.779 ± 0.003	0.525 ± 0.007	0.791 ± 0.001	0.614 ± 0.003	0.385 ± 0.007	0.279 ± 0.006
	w/o Gene Exp	0.791 ± 0.003	0.573 ± 0.004	0.801 ± 0.002	0.658 ± 0.005	0.476 ± 0.010	0.361 ± 0.009
ALMANAC-Lung	w/o Prot Level	0.768 ± 0.004	0.509 ± 0.008	0.792 ± 0.002	0.631 ± 0.009	0.422 ± 0.019	0.306 ± 0.016
	w/o Gene Exp	0.794 ± 0.002	0.555 ± 0.004	0.805 ± 0.002	0.650 ± 0.004	0.460 ± 0.008	0.351 ± 0.007
ALMANAC-Ovary	w/o Prot Level	0.777 ± 0.002	0.529 ± 0.003	0.814 ± 0.001	0.623 ± 0.006	0.398 ± 0.014	0.307 ± 0.012
	w/o Gene Exp	0.779 ± 0.003	0.536 ± 0.007	0.816 ± 0.002	0.624 ± 0.005	0.400 ± 0.012	0.311 ± 0.011
ALMANAC-Skin	w/o Prot Level	0.764 ± 0.004	0.438 ± 0.007	0.805 ± 0.002	0.579 ± 0.008	0.292 ± 0.023	0.204 ± 0.017
	w/o Gene Exp	0.787 ± 0.003	0.498 ± 0.006	0.816 ± 0.002	0.613 ± 0.005	0.374 ± 0.011	0.282 ± 0.010
**Study Datasets**							
FRIEDMAN (skin)	w/o Prot Level	0.776 ± 0.007	0.672 ± 0.009	0.725 ± 0.004	0.675 ± 0.006	0.565 ± 0.010	0.373 ± 0.012
	w/o Gene Exp	0.810 ± 0.001	0.710 ± 0.002	0.745 ± 0.002	0.712 ± 0.003	0.629 ± 0.006	0.437 ± 0.006
ONEIL (several tissues)	w/o Prot Level	0.801 ± 0.003	0.751 ± 0.005	0.689 ± 0.008	0.445 ± 0.011	0.727 ± 0.005	0.724 ± 0.006
	w/o Gene Exp	0.817 ± 0.009	0.770 ± 0.010	0.693 ± 0.010	0.469 ± 0.016	0.741 ± 0.008	0.734 ± 0.008

*Note:* w/o Prot Level denotes exclusion of the protein-level data, and w/o Gene Exp denotes exclusion of the gene expression data.

**Figure 9:**
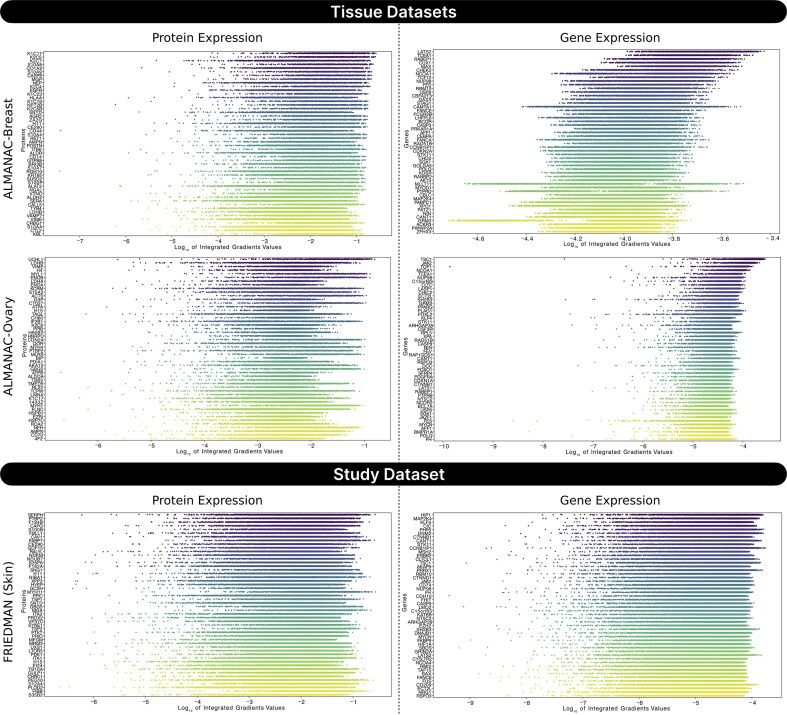
IG attribution scores for the top 50 protein and gene features identified by the SynProtX-GATFP regression model across tissue datasets (ALMANAC-Breast and ALMANAC-Ovary) and a study dataset (FRIEDMAN). Features are ranked based on the log_10_-transformed mean IG attribution scores, highlighting those most influential in the predictions. IG scores of exactly zero in gene features across ALMANAC-Lung, ALMANAC-Skin, and ONEIL (several tissues) potentially reflect ReLU-induced gradient sparsity.

**Figure 10:**
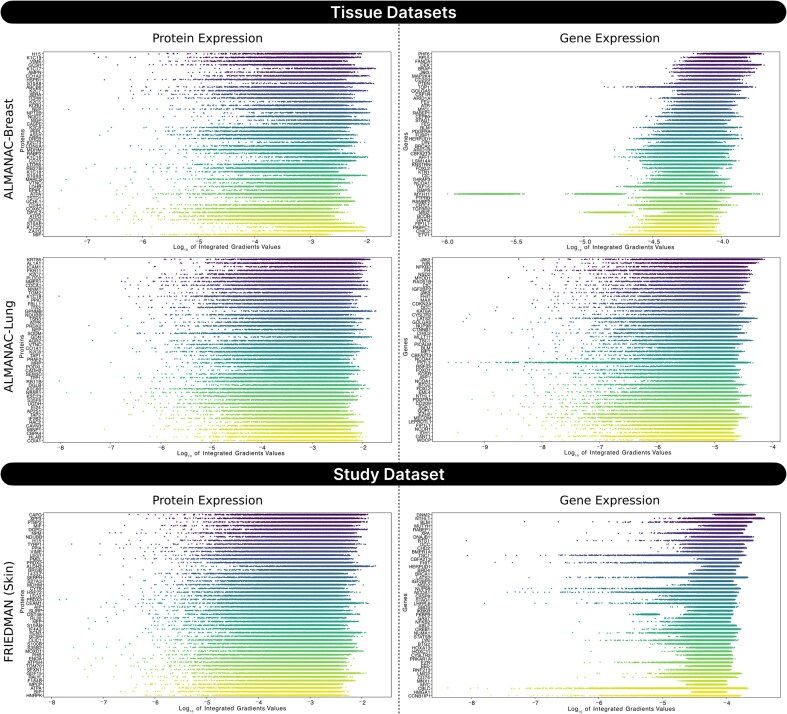
IG attribution scores for the top 50 protein and gene features identified by the SynProtX-GATFP classification model across tissue datasets (ALMANAC-Breast and ALMANAC-Lung) and a study dataset (FRIEDMAN). Features are ranked based on the log_10_-transformed mean IG attribution scores, highlighting those most influential in the predictions. IG scores of exactly zero in gene features across ALMANAC-Ovary potentially reflect ReLU-induced gradient sparsity.

To interpret the contribution of specific features, we employed integrated gradients (IG), a widely used attribution method designed to assess the importance of input features in deep learning models. This method satisfies key axioms such as sensitivity and implementation invariance, making it suitable for analyzing complex biological models. IG enables identification of which proteins or genes drive the model’s predictions, offering transparency and mechanistic insight into the black-box architecture of SynProtX-GATFP. Using IG, we identified the top 50 most influential protein and gene features across regression (Fig. 9) and classification (Fig. 10) tasks. In all examined datasets, especially ALMANAC-Breast, ALMANAC-Lung, and FRIEDMAN, protein features consistently showed higher log_10_-transformed IG scores than gene features.

To explore the biological relevance of these top protein features, we analyzed the top 50 proteins ranked by IG scores in the ALMANAC-Breast regression model. These features were selected for their significant contribution to model predictions and provide insight into the underlying mechanisms of drug response. Several of these proteins are well-established players in breast cancer pathogenesis, particularly in aggressive subtypes. Keratins, including KRT5 and KRT17, are structural proteins typically expressed in basal epithelial cells and have been identified as markers for basal-like and triple-negative breast cancer (TNBC), subtypes that lack hormone receptors and HER2 expression. Their elevated expression correlates with higher tumor grade, chemoresistance, and poor prognosis [41]. Similarly, KRT7 and KRT16 have been implicated in metastatic progression. KRT7 stabilizes epithelial identity and contributes to cell migration, while KRT16 expression is associated with increased tumor cell proliferation and shorter relapse-free survival in breast cancer cohorts [42, 43]. The S100 protein family plays multifunctional roles in inflammation, immune regulation, and metastasis. Notably, S100A4, S100A8, S100A9, and S100P have been found to promote epithelial–mesenchymal transition (EMT), enhance tumor invasiveness, and facilitate immune evasion mechanisms in breast cancer. Elevated expression of these proteins is commonly linked to poor survival and recurrence outcomes [44, 45]. EGFR (epidermal growth factor receptor), a transmembrane tyrosine kinase, is frequently overexpressed in TNBC and drives cellular proliferation, survival, and angiogenesis. It remains a clinically relevant therapeutic target, particularly in basal-like tumors [46]. The extracellular matrix protein COL1A2, encoding collagen type I alpha 2 chain, is upregulated in breast tumors and contributes to desmoplasia, tumor stiffness, and metastasis. Its increased expression is associated with poor recurrence-free survival, likely due to its role in creating a pro-tumorigenic microenvironment [47]. Additional important contributors identified include CASP8, a key initiator of extrinsic apoptosis, with germline mutations associated with familial breast cancer predisposition and tumor immune evasion [48]. MGP (matrix gla protein) functions in tissue remodeling and is upregulated in TNBC; it enhances migration and proliferation through NF-κB signaling and is emerging as a potential therapeutic target [49, 50]. NEP (CD10), a membrane metallopeptidase, is typically downregulated in aggressive breast tumors, and its loss has been associated with increased invasiveness and poor prognosis [51]. Finally, HLA-A, a major histocompatibility complex class I molecule, is involved in antigen presentation and immune recognition. Aberrant expression or allelic variation of HLA-A can impair immune surveillance and promote tumor escape from cytotoxic T lymphocytes [52, 53].

Similarly, in the FRIEDMAN (skin) classification model, IG analysis highlighted several high-attribution proteins with well-documented roles in melanoma biology. TYRP1 (tyrosinase-related protein 1) is a melanosomal enzyme involved in melanin synthesis and is overexpressed in metastatic melanoma, making it a viable target for Chimeric Antigen Receptor T (CAR-T) cell therapy due to its melanocyte specificity [54]. S100B, a calcium-binding protein, is a clinically validated biomarker in melanoma; its serum levels are used for monitoring disease progression and response to treatment, and elevated levels strongly correlate with worse survival [55, 56]. Vimentin (VIME), an intermediate filament protein, is a classical EMT marker that facilitates cytoskeletal reorganization, invasion, and metastatic spread. Its expression is a hallmark of aggressive melanoma phenotypes [57]. The antioxidant enzyme PRDX2 (peroxiredoxin-2) has been shown to inhibit EMT and reduce invasiveness in melanoma, likely through modulation of redox-sensitive transcription factors [58]. CEACAM1 (carcinoembryonic antigen-related cell adhesion molecule 1) is overexpressed in melanoma and functions as both an adhesion molecule and immune checkpoint modulator. It promotes tumor cell proliferation and facilitates immune escape by inhibiting natural killer (NK)– and T cell–mediated cytotoxicity [59]. Lastly, GDF15 (growth differentiation factor 15), a stress-induced cytokine, has emerged as a biomarker of poor prognosis and is implicated in resistance to immune checkpoint inhibitors and reduced antitumor immunity in patients with melanoma [60].

### SynProtX-GATFP demonstrates generalization capability on cold-start scenarios

To assess the generalizability of SynProtX-GATFP in realistic drug discovery settings, we conducted 3 cold-start validation experiments on classification tasks: (i) leave-drug-combination-out, (ii) leave-drug-out, and (iii) leave-cell-line-out. These scenarios simulate the prediction of synergy for unseen drug pairs, novel drugs, and uncharacterized cell lines, respectively. By systematically excluding specific drugs, combinations, or cell lines during training and testing performance on these unseen cases, we can better evaluate the model’s ability to generalize and practical applicability in real-world drug development pipelines [11, 61].

**Figure 11:**
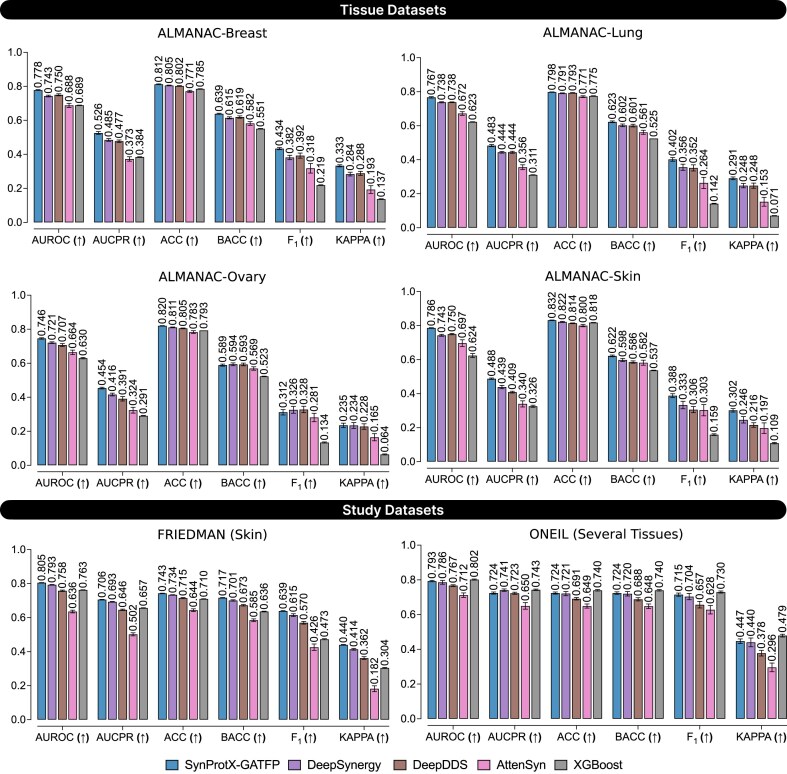
Model performance comparison on the classification task using the leave-drug-combination-out cross-validation technique for the tissue datasets: (1) ALMANAC-Breast, (2) ALMANAC-Lung, (3) ALMANAC-Ovary, and (4) ALMANAC-Skin and the study datasets: (1) FRIEDMAN and (2) ONEIL with different training models.

**Figure 12:**
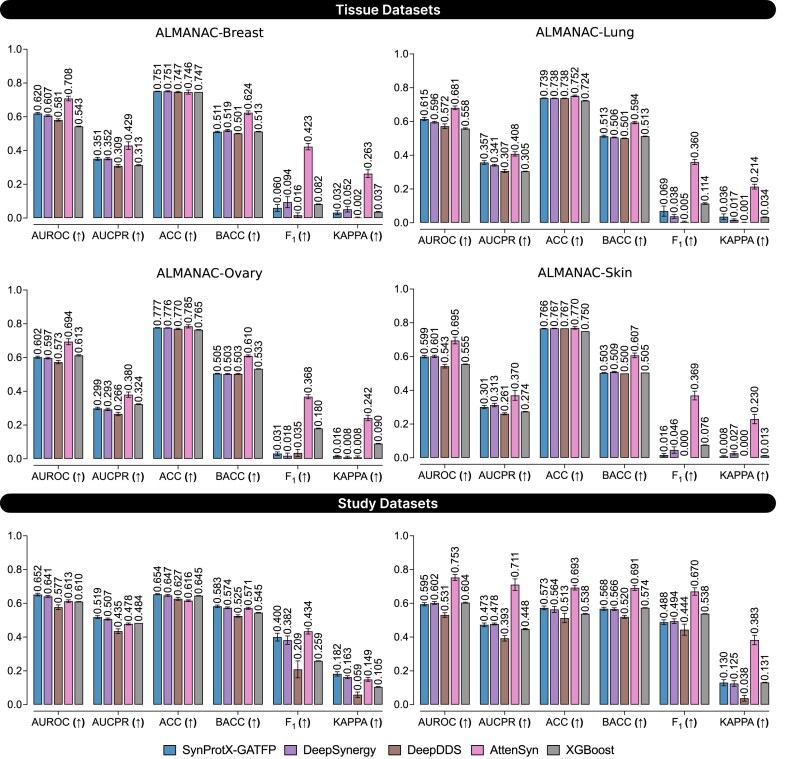
Model performance comparison on the classification task using the leave-drug-out cross-validation technique for the tissue datasets: (1) ALMANAC-Breast, (2) ALMANAC-Lung, (3) ALMANAC-Ovary, and (4) ALMANAC-Skin and the study datasets: (1) FRIEDMAN and (2) ONEIL with different training models.

**Figure 13:**
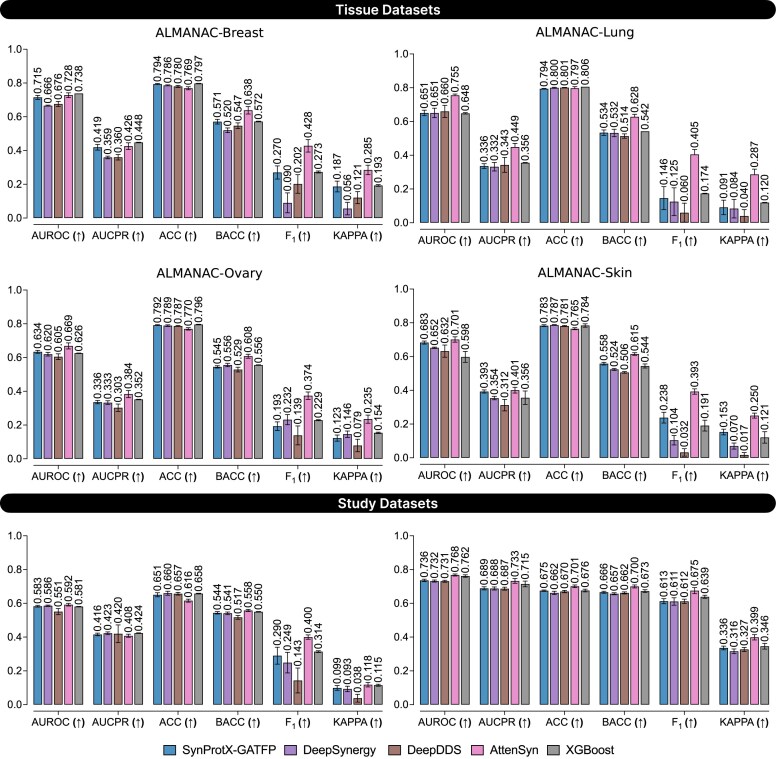
Model performance comparison on the classification task using the leave-cell-lines-out cross-validation technique for the tissue datasets: (1) ALMANAC-Breast, (2) ALMANAC-Lung, (3) ALMANAC-Ovary, and (4) ALMANAC-Skin and the study datasets: (1) FRIEDMAN and (2) ONEIL with different training models.

As shown in Fig. 11, under the leave-drug-combination-out validation, SynProtX-GATFP consistently achieved superior performance across all metrics and datasets. Its AUROC and F_1_ were notably higher in datasets like ALMANAC-Ovary, ALMANAC-Skin, and ONEIL, highlighting its strength in learning drug–pair interactions even when combinations were unseen during training. Under the more stringent leave-drug-out validation (Fig. 12), where entire drug compounds were held out, performance decreased substantially across all models, including SynProtX-GATFP. While it still achieved high performance compared to the baselines in AUROC and balanced accuracy (BACC), its F_1_ and kappa scores were considerably lower—sometimes approaching near-zero levels in datasets like FRIEDMAN and ALMANAC-Skin. These results underscore a known limitation of deep learning models in generalizing to unseen chemical space, particularly when the structural or functional characteristics of the novel compounds differ significantly from those in the training set [62, 63]. The leave-cell-lines-out validation (Fig. 13), simulating predictions in previously unseen biological systems, presented the most difficult setting. While SynProtX-GATFP continued to outperform competing methods in AUROC and BACC, it exhibited noticeable reductions in AUCPR, F_1_, and kappa metrics, especially in datasets such as ALMANAC-Lung and ALMANAC-Breast. These results suggest a potential overfitting to cell line–specific signatures and limited capacity to adapt across different biological contexts, an issue previously reported in drug response prediction models that fail to incorporate comprehensive biological context or pan-cancer features [64, 65].

### Rediscovery of clinically validated synergistic and antagonistic drug combinations

The SynProtX-GATFP model was validated by its ability to rediscover the known synergistic and antagonistic effects of US Food and Drug Administration (FDA)–approved drug combinations, as shown in Table 3. This validation is crucial as it demonstrates not only the predictive accuracy of the model but also its relevance in real-world clinical scenarios. The model accurately predicted the synergistic effects of several drug combinations across different tissues and cell lines, which is consistent with the findings of existing research. For instance, the combination of gefitinib and vorinostat was predicted to have a high probability of synergy in the lung tissue, with a score of 0.9928. This prediction is supported by studies demonstrating the feasibility and efficacy of this combination in activating apoptosis and reducing the levels of key survival proteins such as EGFR, MET, and AKT [66, 67]. Another example is the docetaxel–lapatinib combination in breast tissue, with a predicted synergy score of 0.8477. This finding is corroborated by clinical studies that have confirmed the effectiveness of this combination as a neoadjuvant treatment for HER2^+^ breast cancer [68]. Lastly, the gefitinib–dasatinib combination was predicted to be synergistic in ovarian tissue, with a score of 0.9374, consistent with research showing its antitumor properties through the suppression of crucial signaling pathways [69].

**Table 3:** Rediscovering synergistic and antagonistic effects of FDA-approved drug combinations on the test set through the SynProtX-GATFP classification model

Drug A	Drug B	Tissue	Cell line	Predicted score (probability)	Publication (PMCID)
**Synergistic Effect**					
Gefitinib	Vorinostat	Lung	NCI-H226	0.9928	25552401 [66], 30365122 [67]
Docetaxel	Lapatinib	Breast	MDA-MB-468	0.8477	22999386 [68]
Gefitinib	Dasatinib	Ovary	OVCAR-5	0.9374	28446239 [69]
**Antagonistic Effect**					
Mitoxantrone	Ifosfamide	Lung	HOP-62	1.2089E-06	3735704 [70]
Chlorambucil	6-Mercaptopurine	Breast	MDA-MB-468	3.8256E-05	31813938 [71]
Temozolomide	Romidepsin	Ovary	OVCAR-8	9.6136E-05	15196862 [72]

In addition to identifying synergistic effects, SynProtX-GATFP also successfully predicted antagonistic interactions. For example, the mitoxantrone–ifosfamide combination was expected to have a negligible probability of synergy in lung tissue, with a score of 1.2089E-06. This prediction is validated by existing clinical data indicating the limited efficacy of this combination in treating non–small cell lung cancer (NSCLC) due to its low antitumor activity [70]. Similarly, the combination of chlorambucil and 6-mercaptopurine in breast tissue was predicted to have an antagonistic interaction, supported by clinical trials showing limited effectiveness and significant side effects in patients with breast cancer [71]. Lastly, temozolomide and romidepsin were predicted to exhibit antagonism in the ovarian tissue, which aligns with findings indicating a lack of clinical response in treated patients [72].

### SynProtX-GATFP supports model explainability via protein networks and molecular substructures for drug combinations

**Figure 14:**
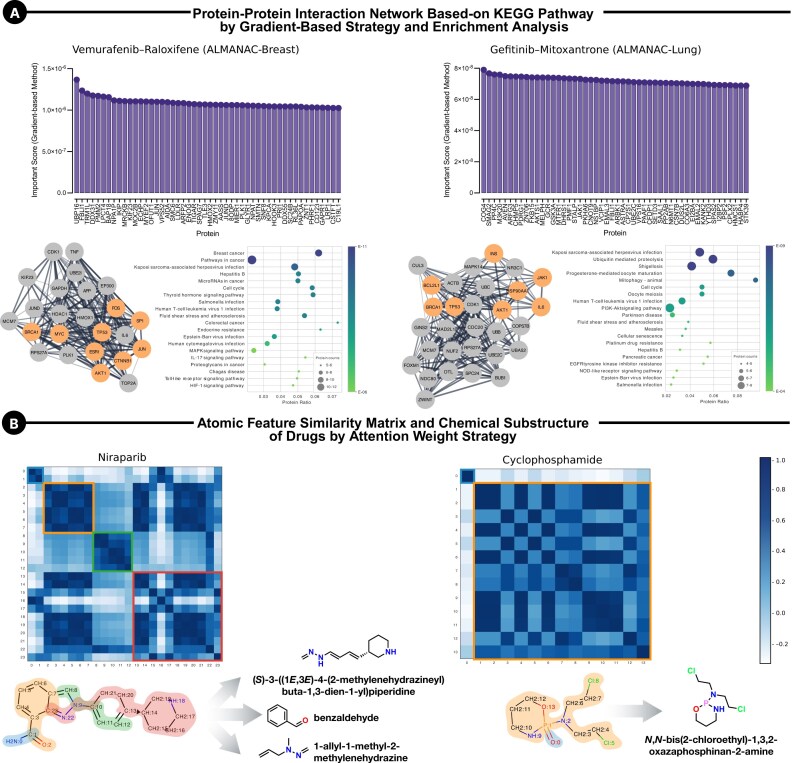
Model interpretability of SynProtX-GATFP. (A) Protein–protein interaction networks based on KEGG pathways using a gradient-based strategy and enrichment analysis for vemurafenib–raloxifene in the ALMANAC-Breast dataset and gefitinib–mitoxantrone in the ALMANAC-Lung dataset. Orange nodes indicate proteins associated with cancer pathways. (B) Atomic feature similarity matrix and chemical substructure analysis of drugs using an attention-weight strategy for niraparib and cyclophosphamide.

The SynProtX-GATFP model not only demonstrates high predictive accuracy but also offers significant explainability, which is crucial for understanding the underlying biological mechanisms driving its predictions. By leveraging gradient-based and attention-weight strategies, SynProtX-GATFP provides insights into relevant cancer-associated proteins and important chemical substructures that contribute to the model’s predictions, thus supporting its interpretability. The gradient-based method from SynProtX-GATFP revealed the key cancer-associated proteins involved in the predicted synergistic effects of the drug combinations, as depicted in Fig. 14A and Supplementary Table S16. For instance, the combination of vemurafenib and raloxifene in breast tissue revealed a protein–protein interaction (PPI) network with several proteins associated with breast cancer pathways, including PLK1, JUN, and MYEF2. These proteins are critical for cell cycle regulation and apoptosis, which are essential for cancer progression [73–75]. Similarly, the combination of gefitinib and mitoxantrone in lung tissue highlighted a PPI network involving proteins linked to the PI3K-Akt signaling pathway, such as AKTS1 and GSK3A, which are known to play a role in cancer cell survival and proliferation [76, 77]. The identification of these proteins and their associated pathways underscores the ability of the model to predict drug synergy and elucidate the molecular basis of these interactions. In addition to protein interactions, SynProtX-GATFP provides valuable insights into the chemical substructures driving drug synergy or antagonism through an attention-weight strategy. As shown in Fig. 14B and Supplementary Table S16, the atomic feature similarity matrix for niraparib and cyclophosphamide revealed critical substructures, such as the methylenhydrazine and benzaldehyde groups in niraparib and the oxazaphosphorine derivative in cyclophosphamide, which are pivotal in their anticancer activity [78]. Piperidine, another compound highlighted in the analysis, has demonstrated significant anticancer potential in various cancer types, including breast, prostate, and colon cancers. The mechanism of action of piperidine involves the regulation of key signaling pathways such as NF-κB, PI3K/Aκt, and STAT-3, which are crucial for cancer cell survival and proliferation. Studies have shown that piperidine can induce apoptosis, inhibit cell migration, and arrest the cell cycle, making it a potent component of anticancer drug design [79]. By highlighting these specific chemical features, the model offers a deeper understanding of how molecular structure influence drug interactions, which is vital for rational drug design and development of more effective combination therapies. By providing insights into the biological and chemical features that drive its predictions, the model offers interpretable insights that help researchers better understand the factors contributing to its predictions, moving toward more transparent and explainable outputs. This level of interpretability is crucial for validating the predictions of the model in a clinical context and gaining trust in its applications for drug development.

### IG method of SynProtX-GATFP explains block effects

To investigate the observed block effects, where certain drugs consistently perform better across multiple cell lines, we conducted a mechanistic interpretability analysis using the IG method on the SynProtX-GATFP model. We selected a top proportion drug found in the top 50 from the ALMANAC-Breast and ALMANAC-Lung test sets and extracted the 50 most influential proteins for each drug–cell line pair based on IG attribution scores. To elucidate the molecular mechanisms underlying these effects, we performed gene set enrichment analysis (GSEA) using the MSigDB Oncogenic Signature library, which includes curated gene sets associated with oncogenic signaling pathways and cancer driver genes. Significance was determined using false discovery rate (FDR)–adjusted *P* values. Furthermore, we conducted molecular fragment analysis to identify key structural features associated with the observed pathway activations. Our analyses focused on 2 representative combinations: vismodegib–mitotane in breast cancer cell lines (MDA-MB-468 and MCF-7) and vandetanib–gefitinib in lung cancer cell lines (NCI-H226 and A549).

**Figure 15:**
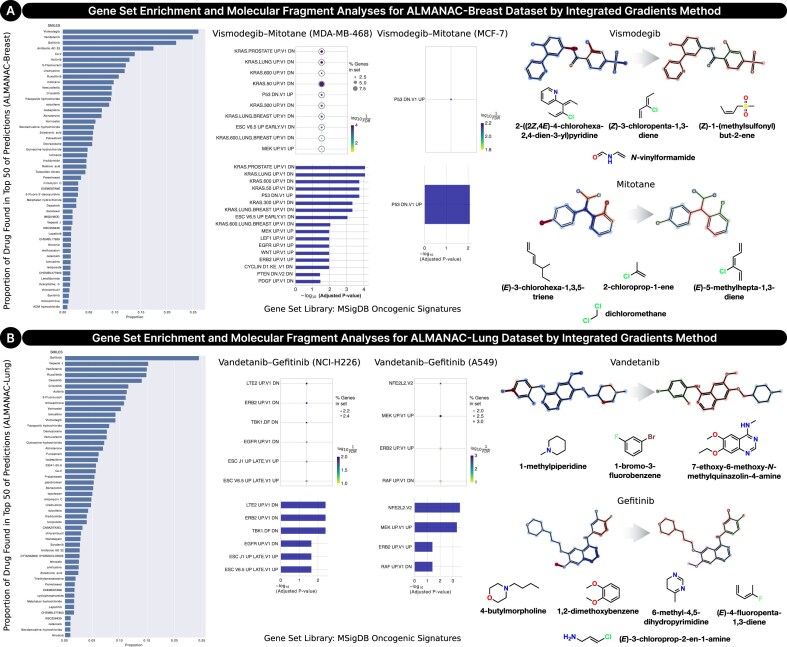
Block effect analysis of SynProtX-GATFP based on the top-ranked 50 proteins by IG method. (A) Gene set enrichment and molecular fragment analyses of vismodegib–mitotane via MDA-MB-468 and MCF-7 cell lines on the ALMANAC-Breast dataset. (B) Gene set enrichment and molecular fragment analyses of vandetanib–gefitinib via NCI-H226 and A549 cell lines on the ALMANAC-Lung dataset. The gene set enrichment analysis is based on a gene set library of MSigDB Oncogenic Signature.

In the MDA-MB-468 cell line (TNBC), GSEA revealed significant enrichment of downregulated KRAS-associated pathways (e.g., *KRAS.LUNG.BREAST UP.V1 DN, KRAS.600 UP.V1 DN*) and upregulation of *P53 DN.V1 UP*, indicating suppression of KRAS signaling and activation of the p53 stress response (Fig. 15A and Supplementary Table S17). This aligns with prior studies showing that vismodegib, a Hedgehog pathway inhibitor, targets hypoxic and stress-associated regions in TNBC tumors [80–82]. Mitotane, known for its broad cytotoxicity, likely enhances synergy in this context, although direct evidence in TNBC remains limited. In contrast, the MCF-7 cell line (estrogen receptor positive) showed more selective pathway enrichment, with only *P53 DN.V1 UP* significantly activated. This suggests a p53-driven mechanism specific to this hormone-responsive subtype. Prior studies have shown mitotane’s ability to suppress proliferation and alter mitochondrial metabolism in MCF-7 cells, supporting its pathway-specific activity [83, 84]. Fragment analysis further corroborates these findings. Vismodegib contains a 2-arylpyridine core and halogenated aromatic rings, facilitating its interaction with the Smoothened (SMO) receptor, an essential component of Hedgehog signaling. These features are particularly relevant in TNBC, where Hedgehog pathway components are frequently overexpressed [85]. Mitotane, structurally derived from DDT, carries chlorinated aromatic groups that localize to the mitochondria and endoplasmic reticulum, triggering ATF4/ATF3-mediated endoplasmic reticulum (ER) stress and apoptosis, mechanisms consistent with p53 pathway activation in MCF-7 cells [86].

In the NCI-H226 cell line (squamous cell lung carcinoma), GSEA identified downregulation of multiple receptor tyrosine kinase pathways, including *EGFR UP.V1 DN, ERBB2 UP.V1 DN*, and *RAF UP.V1 DN* (Fig. 15B and Supplementary Table S18). These findings are supported by preclinical studies showing that vandetanib inhibits tumor growth in NCI-H226-derived mesothelioma via EGFR and VEGFR2 inhibition [87]. Gefitinib has also been shown to enhance antitumor efficacy when combined with agents targeting EGFR and hypoxia-related pathways [88]. In contrast, A549 cells (EGFR wild-type adenocarcinoma) exhibited enrichment in *MEK UP.V1 UP, ERBB2 UP.V1 UP*, and *NFE2L2.V2* pathways, indicating activation of MAPK and oxidative stress signaling. These pathways likely reflect adaptive resistance mechanisms. Consistent with this, vandetanib suppresses p-ERK and p-JNK signaling in A549 cells [87], while gefitinib modulates the PI3K/AKT/mTOR axis and induces autophagy [89]. Molecular fragment analysis provides additional insight into these mechanisms. Both vandetanib and gefitinib share a quinazoline core, a hallmark scaffold in tyrosine kinase inhibitors, enabling selective binding to ATP-binding pockets of kinases like EGFR and VEGFR. Halogenated aromatic substitutions of vandetanib enhance its binding affinity across multiple targets, including RET, VEGFR, and EGFR, aligning with the downregulated signaling observed in NCI-H226 [90–92]. Activity of gefitinib in A549 cells is linked to its ability to modulate redox and autophagic pathways, consistent with NFE2L2 and MEK pathway enrichment [89, 93].

Together, these findings demonstrate that the observed block effects are not merely statistical artifacts but are biologically grounded in specific pathway activations, structural drug features, and cell line contexts. The integration of IG-based feature attribution with GSEA and fragment analysis enables a biologically interpretable framework for error analysis in deep learning models. This approach enhances the mechanistic understanding of drug synergy and cell-specific responses in combination therapy prediction.

### Limitations of the study

SynProtX demonstrates strong performance in predicting the synergistic effects of anticancer drug combinations by integrating large-scale proteomic data, gene expression profiles, and molecular structures. By leveraging graph neural networks, attention mechanisms, and deep learning, the model captures complex biological interactions and provides interpretable predictions that are critical for advancing precision oncology. Despite these strengths, several limitations remain that present opportunities for further development. One key limitation lies in the model’s ability to generalize to unseen data, particularly in cold-start scenarios. While SynProtX-GATFP demonstrates strong performance under traditional validation settings and leave-drug-combination-out, its predictive accuracy diminishes in more challenging scenarios, such as leave-drug-out and leave-cell-line-out evaluations. This reflects a fundamental difficulty in generalizing to novel drugs or unfamiliar cellular contexts. To address this, self-supervised pretraining on molecular graphs or few-shot learning techniques could enhance extrapolation. For example, Meta-MGNN applies molecular graph neural networks to learn molecular representations and utilizes a meta-learning framework to optimize model performance, particularly in scenarios with limited data [94]. Additionally, the model shows signs of overfitting to specific cell line signatures. While SynProtX performs well within homogeneous datasets, its consistency decreases in heterogeneous datasets, such as the ONEIL dataset, where the number of datapoints is limited and cell lines span multiple tissue types. This suggests that SynProtX may not fully capture the cross-tissue biological variation. To improve generalizability, incorporating pathway-informed features or pan-cancer biological representations could help. Pathway activation models, such as DIPx, have shown improvements in personalized drug synergy predictions by focusing on tumor- and drug-specific pathway activation scores [61, 95, 96]. Another limitation of SynProtX is its reliance on static multiomics inputs, which may not adequately reflect the dynamic nature of cellular responses to drug treatment. Cancer cells often undergo rapid molecular reprogramming in response to therapeutic agents. Integrating time-resolved omics data, such as phosphoproteomics or perturbation-induced transcriptomic profiles (e.g., LINCS L1000), could provide a more accurate representation of these adaptive processes and improve the prediction of synergistic effects under real-world conditions [97, 98]. Furthermore, data imbalance across drug–cell line pairs poses a challenge, as some cell lines or tissue types are underrepresented in the training data, leading to inconsistencies in model performance. Future work could address this by utilizing generative modeling techniques, such as generative adversarial networks (GANs) or variational autoencoders (VAEs), to create synthetic omics profiles or by adopting transfer learning approaches to enrich training for rare or sparsely sampled conditions [99].

## Potential Implications

While the primary focus of SynProtX is on predicting synergistic effects of anticancer drug combinations, the methodology and data presented in this work open up several potential avenues for broader applications that extend beyond cancer research. The integration of large-scale proteomic data, gene expression profiles, and molecular structures, paired with advanced deep learning models such as graph neural networks, can provide valuable insights for a wide range of scientific fields and industries.

### Drug discovery for personalized medicine to other diseases

SynProtX’s integration of proteomic, gene expression, and molecular structure data can be adapted for drug discovery in diseases beyond cancer, such as cardiovascular, diabetes, or neurological disorders. By using patient-specific omics data, the model can predict drug interactions tailored to individual molecular profiles, improving the effectiveness and safety of treatments. This approach can help design personalized drug regimens for conditions like Alzheimer’s or Parkinson’s disease, optimizing therapy based on specific disease mechanisms and reducing adverse effects.

### Pharmacoproteomics for drug toxicity prediction

SynProtX’s integration of proteomic data can enhance pharmacoproteomics by predicting drug toxicity. By analyzing protein expression profiles, the model can identify molecular mechanisms of drug-induced toxicity, such as liver or heart toxicity. Incorporating time-resolved proteomics and perturbation data could improve predictions of adverse effects over time, helping identify toxic compounds early in the drug development process. This could lead to safer drugs and more efficient screening methods, reducing the risk of late-stage failures.

### Data-driven biomarker discovery

SynProtX’s data-driven approach to drug synergy prediction can be applied to biomarker discovery for various diseases. By analyzing protein expression and drug interactions, the model can identify biomarkers linked to disease progression or treatment response. This could accelerate the identification of biomarkers for conditions such as autoimmune diseases, chronic inflammation, or infections, improving diagnostic accuracy and enabling personalized treatment strategies.

## Methods

### Data collection and preparation

The dataset for developing the deep learning models was sourced from the DrugComb database version 1.5 [26, 27]. This database contains drug combination data from various diseases obtained through high-throughput laboratory screenings, including cancer, malaria, Ebola, and SARS-CoV-2. Specifically, for anticancer drug combination data, studies such as ALMANAC [100], FRIEDMAN [31], and ONEIL (O’Neil) [101] were used to compile the dataset. The records in the dataset represent drug combinations tested against cell lines, with the respective Loewe scores documented. To preprocess the data, we eliminated all invalid entries, such as blank cells. Next, records with Loewe scores higher than 50 or lower than –75 were discarded to remove any potential outliers. Additionally, only drugs available in the ChEMBL database [102–104] were included, and cell lines were limited to those with overlapping between gene expression and protein expression data. Duplicate triplets of a cell line and the corresponding standard drug names were removed. As a result, a drug combination dataset with target values of 94,862 entries was obtained, including the tissue datasets (1) ALMANAC-Breast, (2) ALMANAC-Lung, (3) ALMANAC-Ovary, and (4) ALMANAC-Skin and the study datasets: (1) FRIEDMAN and (2) ONEIL (Supplementary Table S1).

To prepare gene expression data from human cancer cells, we slightly modified the method described by Liu et al. [105]. Initially, data were collected from The Cancer Dependency Map (DepMap) version 22Q2, a database that processes omics data of cancer cells from the CCLE [13, 14]. Since the gene expression data from DepMap include genes present in each cell, regardless of their relevance to cancer development, it was necessary to filter for genes significant to cancer development. Gene names were sourced from the Catalogue of Somatic Mutations in Cancer (COSMIC) version 96 [106], a database that compares gene data from human cancer cells to the human genome reference (GRCh38). Finally, the identified 714 genes associated with cancer development were used for model development.

For protein expression data of cancer cells, we utilized the ProCan-DepMapSanger database [19], a comprehensive resource covering protein data for 8,498 proteins from 949 types of cancer cells and over 40 cancer types. These protein data were acquired through laboratory experiments using data-independent acquisition mass spectrometry (DIA-MS), which is noted for its high reproducibility and speed [107]. Ultimately, the identified 6,688 proteins were employed for model development.

### Chemical representations

#### Molecular fingerprints

To represent the structural information of the molecules, we transformed it from a molecular graph into a molecular fingerprint. This fingerprint consists of Boolean molecular descriptors indicating the chemical and physical properties of a molecule. The Extended Connectivity Fingerprint 6 (ECFP6), a 2,048-bit fingerprint generated by processing the Morgan fingerprint with a radius of 3, was used [108, 109]. ECFP6 has been widely used in traditional machine learning models, such as RF and XGBoost, due to its strong performance across various applications. The molecular fingerprints were utilized to develop the SynProtX-GATFP model and were subsequently compared with state-of-the-art models.

#### Molecular graph

To transform the molecular structure data into a molecular graph format, the Simplified Molecular Input Line Entry System (SMILES) notation was used for the drug names present in the dataset. The SMILES notation was obtained from version 31 of the ChEMBL database [102, 103] and was subsequently converted into a molecular graph using the RDKit package. The molecular graph of each drug is defined as $M = ( {V,E} )$, where $V\ $ is a set of nodes and $E\ $ is a set of edges displayed in an adjacency matrix *A*. Each node ${v}_{u} \epsilon V $ represents an atom $u\ $ in the molecule, and each edge ${e}_{uw}\epsilon E $ represents the bond ${e}_{uw}$ between atoms $u\ $ and *w*.

The graph neural network (GNN) utilizes this non-Euclidean and noninvariant molecular graph to extract compound features for predicting bioactivity. For each node, the atomic and bond features ${x}_i\epsilon{v}_u$ are calculated to represent the identity of the node. These features are binary vectors that include implicit values (one-hot and integer). During the GNN learning process, these features are employed through a message-passing phase between each node and its neighboring nodes. In this work, we utilized *ConvMolFeaturizer* for atomic features by DeepChem [110].

### Molecular graph and deep neural networks

#### Graph convolutional network (GCN) [28]

GCN is a graph algorithm that is widely used in image processing, natural language processing (NLP), chemical property prediction of molecules, and systems biology, among others. The propagation process of GCN is defined by the following equation:


(12) \begin{eqnarray*}
H_u^{\left( {l + 1} \right)} = \sigma \left( {{{\hat{D}}}^{ - \frac{1}{2}}\hat{A}{{\hat{D}}}^{ - \frac{1}{2}}{W}^{\left( l \right)}h_u^{\left( l \right)}} \right)
\end{eqnarray*}

Let $\hat{A} = \ A + I$, where *I* is the identity matrix and $\hat{D}$ is the degree matrix of the diagonal nodes obtained from ${\hat{D}}_{uw} = \mathop \sum \limits_w {\hat{A}}_{uw}$. Here, D and A are the degree and adjacency matrices, respectively, according to the order of ${W}^{( l )}$ and $h_u^{( l )}$, which are the weights and learnable parameters from node *u* of layer *l*, respectively. $\sigma $ is the nonlinear activation function. ${\hat{D}}^{{\mathrm{-}}\frac{1}{2}}\hat{A}{\hat{D}}^{{\mathrm{-}}\frac{1}{2}}$ adds self-connections to the nodes and maintains feature vector size. The message-passing phase consists of 2 steps: (i) aggregation of the neighboring node vectors ${h}_w$ to generate the node vector ${\hat{h}}_u$ and (ii) linear projection of ${\hat{h}}_u$ with a nonlinear activation function that can be calculated from the projection function, resulting in the following equation.


(13) \begin{eqnarray*}
{h}_u = \sigma \left( {{W}_u{{\hat{h}}}_u} \right)
\end{eqnarray*}

#### Graph attention network (GAT) [29]

GAT is an algorithm that is an extension of the GCN algorithm. The difference between GAT and GCN is that GAT assigns weights to nodes differently using an attention mechanism to facilitate the process of data aggregation. In GCN, the weights of neighboring atoms are calculated by normalizing the sum to be the same. The propagation process of GAT can be represented with the following equation.


(14) \begin{eqnarray*}
H_u^{\left( {l + 1} \right)} = \sigma \left( {\mathop \sum \limits_{v \epsilon N\left( u \right)} \alpha _{uw}^{\left( l \right)}{W}^{\left( l \right)}h_u^{\left( l \right)}} \right)
\end{eqnarray*}

In this context, the notations *W*, $N( u )$, and $\sigma $ denote the trainable weight matrix, the set of neighboring nodes of node *u*, and nonlinear activation function, respectively. Moreover, the symbol $\alpha _{uw}^{( l )}$ represents the normalized attention score between node *u* and its neighbor *w* in the ${l}_{th}$ graph convolutional layer, which is computed by the softmax function:


(15) \begin{eqnarray*}
{\alpha }_{uw} = \textit{softmax}_w\left( {{e}_{uw}} \right) = \frac{{exp\left( {{e}_{uw}} \right)}}{{\mathop \sum \nolimits_{w \epsilon N\left( u \right)} exp\left( {{e}_{uw}} \right)}}
\end{eqnarray*}

The variable ${e}_{uw}$ denotes the output of the initial layer, which is a feed-forward neural network using the nonlinear activation function of LeakyReLU and without projection (i.e., a bias vector). This can be formulated as follows:


(16) \begin{eqnarray*}
{e}_{uw} = \textit{LeakyReLU}\left( {W \cdot \left[ {{e}_u,{e}_w} \right]} \right)
\end{eqnarray*}

The attention mechanism leverages the ability to selectively attend to different aspects of information to improve performance compared to traditional GCNs.

#### Attentive FP [30]

Attentive FP is a graph neural network architecture that employs a recurrent neural network (RNN) to aggregate information from neighboring and distant atoms to the focal atom of interest. Attentive FP is a state-of-the-art algorithm that can efficiently predict the chemical properties of benchmark molecular datasets with high accuracy on many sub-datasets. One of the reasons why attentive FP can achieve high performance is due to the fact that during message passing, M, the attention mechanism gathers information from neighboring nodes via gated recurrent units (GRUs), which helps filter unnecessary data and select relevant data for learning. The output stage uses a summation function as the output function to convert the graph vector representation ${h}_G$, before the next prediction step, as shown in the following equation.

Message-passing step:


(17) \begin{eqnarray*}
m_u^{\left( {k + 1} \right)} = \mathop \sum \limits_{v\epsilon N\left( u \right)} {M}^{\left( k \right)}\left( {h_u^{\left( k \right)},h_w^{\left( k \right)}} \right)
\end{eqnarray*}

(18) \begin{eqnarray*}
h_u^{\left( {k + 1} \right)} = GR{U}^{\left( k \right)}\left( {m_u^{\left( {k + 1} \right)},h_u^{\left( k \right)}} \right)
\end{eqnarray*}

Output step:


(19) \begin{eqnarray*}
{h}_G = Sum\left( {h_u^{\left( K \right)}{\mathrm{|}}u\epsilon G} \right)
\end{eqnarray*}

#### Deep neural network (DNN)

Deep neural networks (DNNs) are composed of multiple interconnected neurons that gather information from other neurons. The collected data are subsequently processed through nonlinear activation functions to compute the probability. The learning process of deep neural networks involves intricate and rapidly changing nonlinear functions that enable them to extract hierarchical features from the input data. The activation vector *h* of the first hidden unit and each hidden unit at $l + 1$ can be computed using the following equation.


(20) \begin{eqnarray*}
h_i^{\left( 1 \right)} = {\sigma }^{\left( 1 \right)}\left( {\mathop \sum \limits_j W_{ij}^{\left( 1 \right)}{x}_j + b_i^{\left( 1 \right)}} \right)
\end{eqnarray*}

(21) \begin{eqnarray*}
h_i^{\left( {l + 1} \right)} = {\sigma }^{\left( {l + 1} \right)}\left( {\mathop \sum \limits_j W_{ij}^{\left( {l + 1} \right)}h_j^{\left( l \right)} + b_i^{\left( {l + 1} \right)}} \right)
\end{eqnarray*}

where ${x}_j\ $ is the input data vector used to calculate the activation vector of the first layer $h_i^{( 1 )}$ and $h_j^{( l )}$ is the activation vector of layer *l*, ${W}_{ij}$ is the weight matrix that can be learned, *b* is the bias vector, and $\sigma \ $ is a nonlinear activation function

In the classification task, the sigmoid activation function was added to calculate *p*, which is the probability of the input being a synergistic drug combination, as shown in the following equation.


(22) \begin{eqnarray*}
p = \textit{sigmoid}\left( {\mathop \sum \limits_j W_j^{out}h_j^l + {b}^{out}} \right)
\end{eqnarray*}

### Model development

The SynProtX model was developed to predict anticancer drug synergy in a particular cell line using proteomics data. This approach involves conducting both classification and regression tasks across datasets categorized by study datasets (FRIEDMAN and ONEIL) and cancer tissue datasets (ALMANAC-Breast, ALMANAC-Lung, ALMANAC-Ovary, and ALMANAC-Skin). We utilized the PyTorch [111], PyTorch Geometric (PyG) [112], and DeepChem [110] packages as their primary toolkits for developing the model.

For molecular graph learning of compounds, we conducted experimental settings through the following approaches to compare the learning of drug molecule structures:

1. **SynProtX-GATFP:** employs a GAT for learning molecular structures and DNNs for molecular fingerprints
2. **SynProtX-GAT:** employs only a GAT for learning molecular structures
3. **SynProtX-GCN:** employs only a GCN for learning molecular structures
4. **SynProtX-AttFP:** employs only an attentive FP (AttFP) for learning molecular structures

In the comparative analysis of methodologies in drug discovery, our approach incorporated the use of advanced models such as DeepSynergy, DeepDDS, and AttenSyn. In addition to these advanced models, we compared our models with classical algorithms such as XGBoost to establish a baseline. XGBoost is recognized for its state-of-the-art performance across various machine learning challenges and is widely adopted for its robustness and efficiency in handling sparse data. This comprehensive comparison allowed us to thoroughly evaluate the efficacy of our model in the context of both contemporary and classical methodologies.

### SOTA models

The study employed 3 advanced SOTA deep learning models, including DeepSynergy, DeepDDS, and AttenSyn, to predict the synergistic effects of anticancer drug combinations.

#### DeepSynergy [11]

DeepSynergy integrates chemical descriptors and gene expression data into a fully connected neural network, utilizing features from both domains to enhance the predictive accuracy. It is particularly effective in assessing the potential of drug pairs to work synergistically by using established metrics such as AUROC and AUPR for evaluation.

#### DeepDDS [17]

DeepDDS leverages graph neural networks, specifically GCNs and GATs, to learn drug-embedding vectors from molecular graphs, integrating cell line–specific data to enhance prediction accuracy. The attention mechanism of the model captures the complex interactions between drugs and cell lines, enabling precise synergy prediction.

#### AttenSyn [18]

AttenSyn extends this approach by incorporating an attention-based pooling module that identifies and focuses on key substructures within drug molecules. This attention mechanism not only improves the model’s performance but also enhances its interpretability, making it possible to discern the specific molecular features contributing to the predicted outcomes. These models collectively provide a robust framework for predicting drug synergy, supporting drug discovery, and potentially improving combination therapy strategies in oncology.

To ensure a fair comparison, all competitive SOTA models were evaluated using the same criteria as for SynProtX. This included training and testing on identical datasets using the same performance metrics and applying the same cross-validation techniques. By adhering to these consistent evaluation standards, this study provides a robust comparison of model performance, ensuring that the reported results are directly comparable and reflective of each model’s true predictive capabilities.

### Model evaluation

During model training, we randomly partitioned the dataset into training and test sets using an 80:20 ratio with the random splitting method in DeepChem [110]. To ensure robustness, we performed 5-fold cross-validation [113]. The dataset was split into 5 parts, with the model trained on 4 parts and validated on the remaining part. This process was repeated 5 times, each time with a different validation part. We employed Bayesian optimization with 30 trials using Optuna [114] to find the optimal set of hyperparameters, chosen for its efficiency and reduced time consumption with flexible neural network architectures. To further improve the training process and prevent overfitting, we implemented an early stopping mechanism. This early stopping mechanism monitors the validation loss and stops the training process if the performance does not improve for a specified number of epochs, thereby preventing overfitting and saving computational resources. Specifically, training stops if the validation loss does not improve for 30 consecutive epochs or if it increases for 10 consecutive epochs.

To ensure reliable model evaluation, we generated models with different seed numbers independently using the test set. This process was repeated 15 times. The final model in each cross-validation iteration was employed to assess the predictive performance of the model on the test set using selected metrics to compare the ground truth with the predicted drug synergies. We then calculated the mean and confidence interval (α = 0.05) of these results. For performance evaluation, we used distinct metrics for the classification and regression tasks. For the classification, we used the AUROC, AUCPR, accuracy (ACC), BACC, F_1_ score (F_1_), and Cohen’s kappa coefficient (kappa). For the regression, we used the RMSE, MAE, PCC, SCC, and coefficient of determination (*R*^2^). The classification and regression metrics are presented in the Metrics for Model Evaluation subsection of Supplementary S1.

### Model interpretability

#### IG-guided top-ranked features and block effect analysis

To enhance the interpretability of the SynProtX model and identify the key contributors to predicted drug synergy, we applied IG. IG computes the integral of gradients along the straight-line path from a baseline to the actual input. This method was employed to attribute scores to both protein and gene features and to analyze block effects in drug combinations, with a particular focus on protein-level contributions to the model’s predictions.

The first application of IG involved calculating attribution scores for protein and gene features that contribute to the model’s prediction of synergy between drug combinations. The IG method assigns a contribution score to each feature by integrating the gradients of the model’s output with respect to the input, relative to a baseline, typically set to zero. The attribution score for each feature *i* is computed as:


(23) \begin{eqnarray*}
{\mathrm{AttributionScor}}{{\mathrm{e}}}_i\left( x \right) = \left( {{x}_i - x_i^0} \right) \cdot \mathop \smallint \limits_0^1 \nabla F\left( {x^{\prime} + \alpha \left( {x - {x}^0} \right)} \right)d\alpha
\end{eqnarray*}

where ${x}_i$ represents the feature of interest (either protein or gene expression), $x_i^0$ is the baseline feature (set to zero for this analysis), $\nabla F( x )$ represents the gradient of the model’s output with respect to the input *x*, and $\alpha $ is the scaling factor that moves from the baseline to the actual value of the feature.

In addition to feature attribution, we investigated block effects in drug combinations. Block effects occur when one drug in a combination dominates the synergistic effect, causing the synergy to be primarily driven by this drug across multiple cell lines. To identify these block effects, we applied IG to assess the contribution of individual protein features, focusing on the most influential features identified through ablation studies, to the drug combination synergy prediction. By analyzing the attribution scores for each drug combination, we identified the dominant protein features within specific combinations, revealing which proteins had a more substantial impact on synergy. This analysis focused on combinations of high effectiveness and determined the proportion of drugs found in the top 50 over all occurrences of the respective drug in both ALMANAC-Breast and ALMANAC-Lung datasets.

#### Attention-weight strategy elaborates key chemical substructure

To improve the explainability of the SynProtX-GATFP model, we employed an attention-weight strategy that leverages the atomic feature similarity matrix. This method highlights the most relevant molecular substructures by assigning attention weights to atoms in the drug molecules. The attention mechanism is particularly effective in identifying the key interactions between molecular features and predicting the synergistic effects of drug combinations.

The first step in the attention-weight strategy is to construct an atomic feature similarity matrix, which represents the similarity between atoms based on their chemical properties. For each drug in the combination, we used a set of atomic features, such as atomic number, bond type, aromaticity, and electron density, among others. These atomic features are then compared across all atoms within the molecular graph of a drug to calculate a similarity score between each pair of atoms. The atomic feature similarity matrix *S* is formulated as follows:


(24) \begin{eqnarray*}
{S}_{uw} = {\mathrm{sim}}\left( {{f}_u{\mathrm{,}}{f}_w} \right)
\end{eqnarray*}

where ${S}_{uw}$ is the similarity score between atoms *u* and *w*, ${f}_u$ and ${f}_w$ are the feature vectors of atoms *u* and *w*, respectively, and ${\mathrm{sim}}( {{f}_u{\mathrm{,}}{f}_w} )\ $ is the similarity function, which typically uses cosine similarity or Euclidean distance between the feature vectors of atoms.

Next, we computed the attention weights for each atom in the molecule based on the GAT of the model. In the context of SynProtX-GATFP, the attention mechanism is used to assign varying importance to different atoms in the molecular graph based on their relevance to the synergy prediction. The attention weight for each atom is computed by using a learnable attention coefficient, which is updated during training to optimize the model’s predictive performance. The attention weight for the edge connecting atoms is computed as Equation 15. Once the attention weights are computed, they are integrated with the atomic feature similarity matrix to highlight the most significant atomic interactions for drug synergy. The weighted atomic features are then passed through the subsequent layers of the model to make predictions. The integration step can be represented as Equation 14.

## Availability of Source Code and Requirements

- Project Name: SynProtX
- Project homepage: https://github.com/manbaritone/SynProtX
- Operating system(s): Platform independent
- Programming language: Python
- Other requirements: python 3.7.9, deepchem 2.5.0, dgl 0.9.0, dgllife 0.3.2, numpy 1.21.5, openbabel 2.4.1, optuna 3.0.5, pandas 1.3.5, pyg 2.2.0, pytorch 1.12.0, pytorch-cluster 1.6.0, pytorch-scatter 2.1.0, pytorch-sparse 0.6.16, rdkit 2019.03.2, scikit-learn 1.0.2, scipy 1.7.3, torchaudio 0.12.0, torchmetrics 0.11.0, torchvision 0.13.0
- License: MIT
- RRID: SynProtX, RRID:SCR_027034
- Software Heritage PID [115]: swh:1:snp:750d09d4ed20b1628cef1 f20cf0d2b2e518c4a3b;origin=https://github.com/manbaritone/SynProtX
- WorkflowHub [116]: https://workflowhub.eu/workflows/1726?version=3

## Supplementary Material

giaf080_Supplemental_File

giaf080_Authors_Response_To_Reviewer_Comments_Original_Submission

giaf080_Authors_Response_To_Reviewer_Comments_Revision_1

giaf080_GIGA-D-24-00586_Original_Submission

giaf080_GIGA-D-24-00586_Revision_1

giaf080_GIGA-D-24-00586_Revision_2

giaf080_Reviewer_1_Report_Original_Submission Fuhai Li -- 2/10/2025

giaf080_Reviewer_1_Report_Revision_1 Fuhai Li -- 5/31/2025

giaf080_Reviewer_2_Report_Original_Submission Ling Wang -- 2/14/2025

giaf080_Reviewer_2_Report_Revision_1 Ling Wang -- 5/20/2025

## Data Availability

The source code for SynProtX is publicly available at https://github.com/manbaritone/SynProtX [117]. The datasets, hyperparameters, and model checkpoints can be retrieved from Zenodo [118]. The original drug combination datasets, including ALMANAC [100], FRIEDMAN [31], and ONEIL (O’Neil) [101], can be retrieved from the DrugComb Portal (version 1.5) [119]. Protein expression data for cancer cell lines are from ProCan-DepMapSanger [120]. Gene expression data for cancer cell lines from the Cancer Cell Line Encyclopedia (CCLE) [13, 14] are accessible through the Cancer Dependency Map (DepMap) version 22Q2 [121, 122]. A snapshot of the GitHub repository has been archived via Software Heritage [115]. DOME-ML annotations for the SynProtX model and experiment are available through the DOME registry under accession ID 7hk5upi8vx [123]. The SynProtX workflow is also accessible via WorkflowHub [116].

## Additional Files

**Supplementary Method S1**. Metrics for model evaluation.

**Supplementary Fig. S1**. Comparison of scatterplot between ground truth and prediction synergy scores.

**Supplementary Fig. S2**. Comparison of AUROC curve.

**Supplementary Fig. S3**. Comparison of AUCPR curve.

**Supplementary Fig. S4**. Comparison of distribution between ground truth and prediction synergy scores.

**Supplementary Fig. S5**. Comparison of regression loss curve.

**Supplementary Fig. S6**. Comparison of classification loss curve.

**Supplementary Table S1**. Statistic of processed data of anticancer drug combinations.

**Supplementary Table S2**. Predictive performance on regression task across tissue and study datasets.

**Supplementary Table S3**. Predictive performance on classification task across tissue and study datasets.

**Supplementary Table S4**. Predictive performance on regression task across cell lines of ALMANAC-Breast tissue dataset.

**Supplementary Table S5**. Predictive performance on regression task across cell lines of ALMANAC-Lung tissue dataset.

**Supplementary Table S6**. Predictive performance on regression task across cell lines of ALMANAC-Ovary tissue dataset.

**Supplementary Table S7**. Predictive performance on regression task across cell lines of ALMANAC-Skin tissue dataset.

**Supplementary Table S8**. Predictive performance on regression task across cell lines of FRIEDMAN study dataset.

**Supplementary Table S9**. Predictive performance on regression task across cell lines of ONEIL study dataset.

**Supplementary Table S10**. Predictive performance on classification task across cell lines of ALMANAC-Breast tissue dataset.

**Supplementary Table S11**. Predictive performance on classification task across cell lines of ALMANAC-Lung tissue dataset.

**Supplementary Table S12**. Predictive performance on classification task across cell lines of ALMANAC-Ovary tissue dataset.

**Supplementary Table S13**. Predictive performance on classification task across cell lines of ALMANAC-Skin tissue dataset.

**Supplementary Table S14**. Predictive performance on classification task across cell lines of FRIEDMAN study dataset.

**Supplementary Table S15**. Predictive performance on classification task across cell lines of ONEIL study dataset.

**Supplementary Table S16**. Relevant cancer-associated proteins of cancer drug combinations from gradient-based method.

**Supplementary Table S17**. Top-ranked 50 proteins by integrated gradients method across SynProtX-GATFP on the ALMANAC-Breast tissue dataset for vismodegib–mitotane.

**Supplementary Table S18**. Top-ranked 50 proteins by integrated gradients method across SynProtX-GATFP on the ALMANAC-Lung dataset for vandetanib–gefitinib.

**Supplementary Table S19**. Hyperparameter settings of SynProtX.

**Supplementary Table S20**. Best hyperparameter settings of SynProtX-GATFP on classification task.

**Supplementary Table S21**. Best hyperparameter settings of SynProtX-GATFP on regression task.

## Abbreviations

ACC: accuracy; AttFP: attentive FP; AUCPR: area under the precision-recall curve; AUROC: area under the receiver operating characteristic curve; BACC: balanced accuracy; CAR-T: chimeric antigen receptor T; CCLE: Cancer Cell Line Encyclopedia; DL: deep learning; DNN: deep neural network; ECFP: extended connectivity fingerprint; F_1_: F_1_ score; FDR: false discovery rate; GANs: generative adversarial networks; GAT: graph attention network; GATFP: graph attention network and molecular fingerprint; GCN: graph convolutional network; GNN: graph neural network; GRU: gated recurrent unit; GSEA: gene set enrichment analysis; IG: integrated gradients; KAPPA: Cohen’s kappa coefficient; MAE: mean absolute error; ML: machine learning; MLP: multilayer perceptron; PCA: principal component analysis; PCC: Pearson correlation coefficient; PPI: protein–protein interaction; *R*^2^: coefficient of determination; ReLU: rectified linear unit; RF: random forest; RMSE: root mean square error; RNN: recurrent neural network; SCC: Spearman’s rank correlation coefficient; SMILE: simplified molecular-input line-entry system; SOTA: state-of-the-art; SVM: support vector machine; t-SNE: t-distributed stochastic neighbor embedding; VAEs: variational autoencoders; XGBoost: extreme gradient boosting.
