# Supplementary material for: SynProtX: a large-scale proteomics-based deep learning model for predicting synergistic anticancer drug combinations
Source: Gigascience. 2025 Aug 12;14:giaf080. doi: 10.1093/gigascience/giaf080 (PMC12343095; doi:10.1093/gigascience/giaf080)
Supplement: giaf080_Supplemental_File [file giaf080_supplemental_file.pdf]

Supplementary material

SynProtX: A Large-Scale Proteomics-Based Deep Learning Model for Predicting Synergistic Anticancer Drug Combinations

Bundit Boonyarit[1], Matin Kositchutima[2], Tisorn Na Phattalung[2], Nattawin Yamprasert[3], Chanitra Thuwajit[4], Thanyada Rungrotmongkol[5,6,*], Sarana Nutanong[1,*]

[1]School of Information Science and Technology, Vidyasirimedhi Institute of Science and Technology, Rayong, 21210, Thailand

[2]Kamnoetvidya Science Academy, Rayong, 21210, Thailand

[3]School of Information, Computer, and Communication Technology, Sirindhorn International Institute of Technology, Thammasat University, Pathum Thani, 12120, Thailand

[4]Department of Immunology, Faculty of Medicine Siriraj Hospital, Mahidol University, Bangkok, 10700, Thailand

[5]Program in Bioinformatics and Computational Biology, Graduate School, Chulalongkorn University, Bangkok, 10330, Thailand

[6]Center of Excellence in Structural and Computational Biology, Department of Biochemistry, Faculty of Science, Chulalongkorn University, Bangkok, 10330, Thailand

* *Corresponding authors*

Table of Contents

SUPPLEMTANRY METHOD **4**
S1 Metrics for Model Evaluation 4
SUPPLEMENTARY FIGURES **7**
S1 Comparison of Scatter Plot between Ground Truth and Prediction Synergy Scores 7
S2 Comparison of AUROC Curve 8
S3 Comparison of AUCPR Curve 9
S4 Comparison of Distribution between Ground Truth and Prediction Synergy Scores 10
S5 Comparison of Regression Loss Curve 11
S6 Comparison of Classification Loss Curve 12
SUPPLEMENTARY TABLES **13**
S1 Statistic of Processed Data of Anticancer Drug Combinations 13
S2 Predictive Performance on Regression Task Across Tissue and Study Datasets 14
S3 Predictive Performance on Classification Task Across Tissue and Study Datasets 17
S4 Predictive Performance on Regression Task Across Cell Lines of ALMANAC-Breast Tissue Dataset 20
S5 Predictive Performance on Regression Task Across Cell Lines of ALMANAC-Lung Tissue Dataset 21
S6 Predictive Performance on Regression Task Across Cell Lines of ALMANAC-Ovary Tissue Dataset 23
S7 Predictive Performance on Regression Task Across Cell Lines of ALMANAC-Skin Tissue Dataset 24
S8 Predictive Performance on Regression Task Across Cell Lines of FRIEDMAN Study Dataset 25
S9 Predictive Performance on Regression Task Across Cell Lines of ONEIL Study Dataset 27
S10 Predictive Performance on Classification Task Across Cell Lines of ALMANAC-Breast Tissue Dataset 29
S11 Predictive Performance on Classification Task Across Cell Lines of ALMANAC-Lung Tissue Dataset 31

S12 Predictive Performance on Classification Task Across Cell Lines of ALMANAC-Ovary Tissue Dataset 33

S13 Predictive Performance on Classification Task Across Cell Lines of ALMANAC-Skin Tissue Dataset 35

S14 Predictive Performance on Classification Task Across Cell Lines of FRIEDMAN Study Dataset 37

S15 Predictive Performance on Classification Task Across Cell Lines of ONEIL Study Dataset 39

S16 Relevant Cancer-Associated Proteins of Cancer Drug Combinations from Gradient-Based Method 41

S17 Top-ranked 50 Proteins by Integrated Gradients Method Across SynProtX-GATFP on the ALMANAC-Breast dataset for Vismodegib–Mitotane 42

S18 Top-ranked 50 Proteins by Integrated Gradients Method Across SynProtX-GATFP on the ALMANAC-Lung dataset for Vandetanib–Gefitinib 44

S19 Hyperparameter Settings of SynProtX 46

S20 Best Hyperparameter Settings of SynProtX-GATFP on Regression Task 47

S21 Best Hyperparameter Settings of SynProtX-GATFP on Classification Task 50

REFERENCES 53

SUPPLEMTANRY METHOD

S1 Metrics for Model Evaluation

The regression metrics are calculated by the following formula:

$$RMSE = \sqrt{\frac{\sum_{i=1}^{n}\left(y_{observed} - y_{predicted}\right)^2}{N}} \tag{1}$$

RMSE represents the square root of the average squared differences between predicted and observed values, highlighting large errors.

$$MAE = \frac{1}{n}\sum_{i=1}^{n}\left|y_{observed} - y_{predicted}\right| \tag{2}$$

MAE is the average of the absolute differences between predicted and observed values, providing a straightforward measure of prediction accuracy.

$$PCC = \frac{\sum_{i=1}^{n}\left(y_{observed} - y_{mean(observed)}\right)\left(y_{predicted} - y_{mean(predicted)}\right)}{\sqrt{\sum_{i=1}^{n}\left(y_{observed} - y_{mean(observed)}\right)^2\left(y_{predicted} - y_{mean(predicted)}\right)^2}} \tag{3}$$

PCC quantifies the linear relationship between two variables, indicating both the strength and direction of correlation.

$$SCC = 1 - \frac{6\sum d_i^2}{n(n^2 - 1)} \tag{4}$$

SCC measures the strength and direction of the monotonic relationship between two ranked variables, where d is the pairwise distances of the ranks of the $y_{observed}$ and $y_{predicted}$.

$$R^2 = 1 - \frac{\sum\left(y_{observed} - y_{predicted}\right)^2}{\sum\left(y_{observed} - y_{mean(observed)}\right)^2} \tag{5}$$

R^2 explains the proportion of variance in the dependent variable that is predictable from the independent variable(s).

The classification metrics are calculated by the following formula:

$$AUROC = \int_{0}^{1} TPR(FPR)\, d(FPR) \tag{6}$$

AUROC measures the area under the curve of the true positive rate (TPR) against the false positive rate (FPR) or 1-specificity at various threshold settings. It indicates the model's ability to distinguish between classes.

$$AUCPR = \int_{0}^{1} Precision(Recall) d(Recall) \tag{7}$$

AUCPR evaluates the area under the curve plotting precision against recall. It is particularly useful for imbalanced datasets, where it highlights the trade-off between precision and recall.

$$ACC = \frac{TP + TN}{TP + TN + FP + FN} \tag{8}$$

ACC is the ratio of correctly predicted instances to the total number of instances, where TP is true positive, TN is true negative, FP is false positive, and FN is false negative.

$$BACC = \frac{TPR + TNR}{2} \tag{9}$$

BACC accounts for class imbalance by averaging the sensitivity (true positive rate, TPR) and specificity (true negative rate, TNR).

$$F_1 = 2 \cdot \frac{Precision \cdot Recall}{Precision + Recall} \tag{10}$$

The F_1 score is the harmonic mean of precision and recall, balancing both concerns in imbalanced datasets

$$KAPPA = \frac{p_o - p_e}{1 - p_e} \tag{11}$$

KAPPA measures the agreement between predicted (p_e) and observed (p_o) classifications, adjusted for chance agreement.

SUPPLEMENTARY FIGURES

S1 Comparison of Scatter Plot between Ground Truth and Prediction Synergy Scores

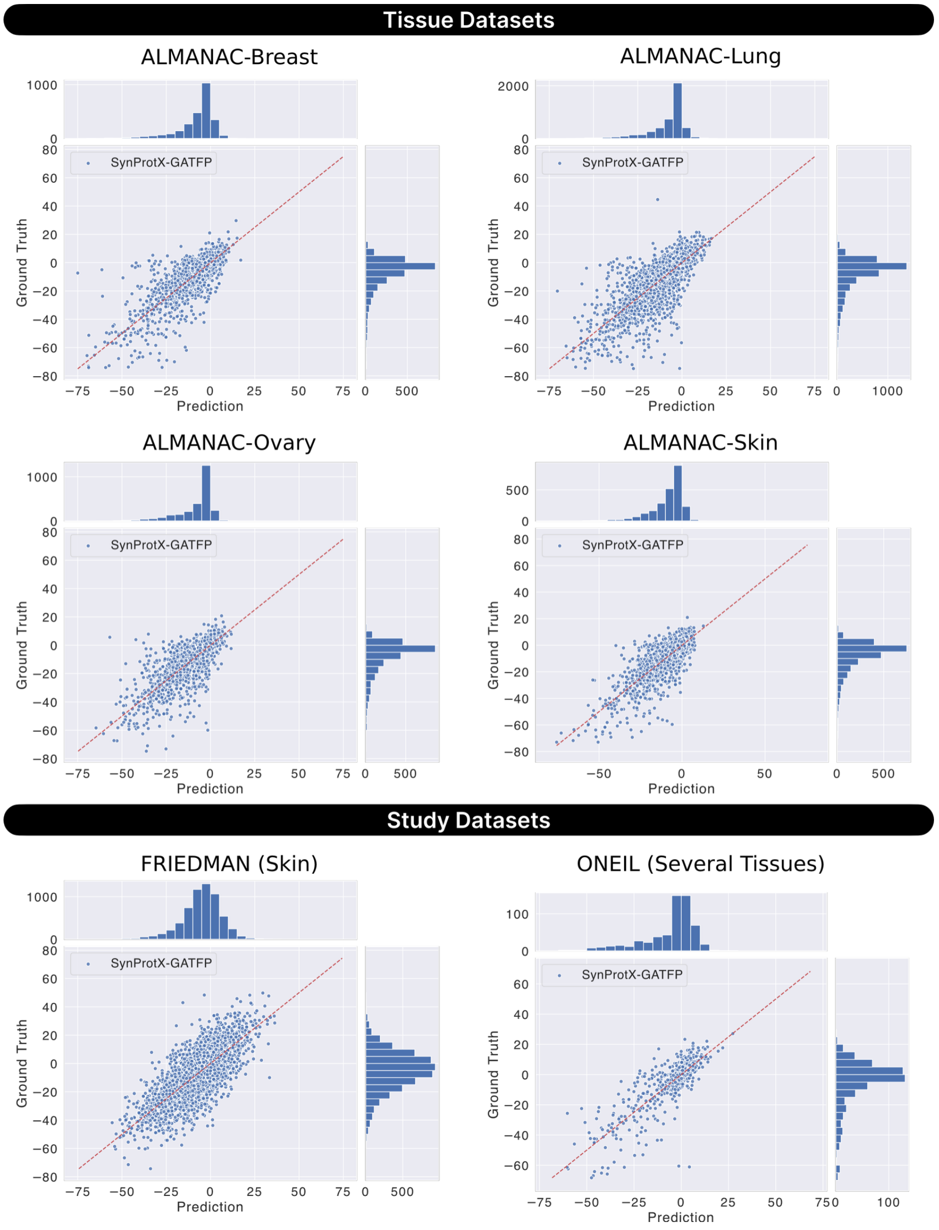

Figure S1. Comparison of scatter plot between ground truth and prediction synergy scores on test set across SynProtX-GATFP for the Tissue Datasets: (1) ALMANAC-Breast, (2) ALMANAC-Lung, (3) ALMANAC-Ovary, and (4) ALMANAC-Skin; and the Study Datasets: (1) FRIEDMAN and (2) ONEIL. The plots were conducted under the best experimental setting and best epoch for each model.

S2 Comparison of AUROC Curve

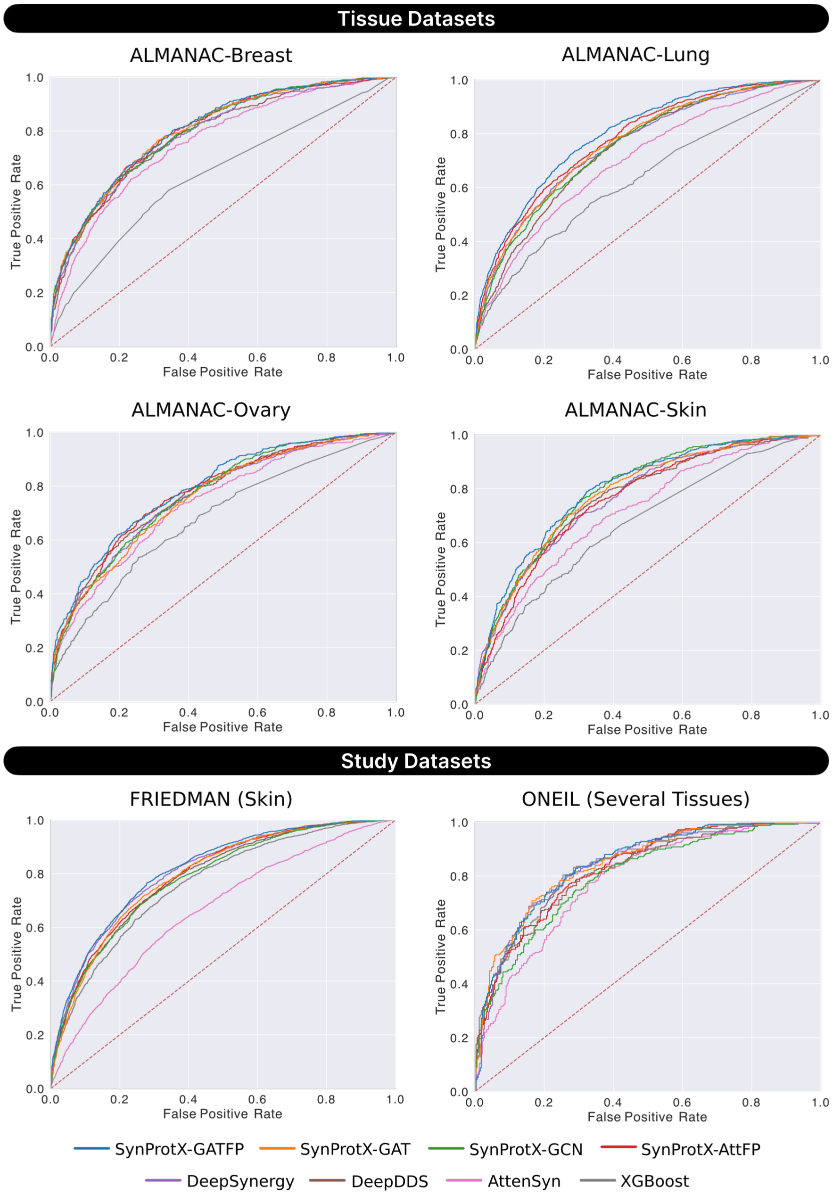

Figure S2. Comparison of receiver operating characteristic curve across SynProtX-GATFP (blue), SynProtX-GAT (orange), SynProtX-GCN (green), SynProtX-AttFP (red), DeepDDS (violet), DeepSynergy (brown), AttenSyn (pink), and XGBoost (gray) for the Tissue Datasets: (1) ALMANAC-Breast, (2) ALMANAC-Lung, (3) ALMANAC-Ovary, and (4) ALMANAC-Skin; and the Study Datasets: (1) FRIEDMAN and (2) ONEIL. The curves were conducted under the best experimental setting and best epoch for each model.

S3 Comparison of AUCPR Curve

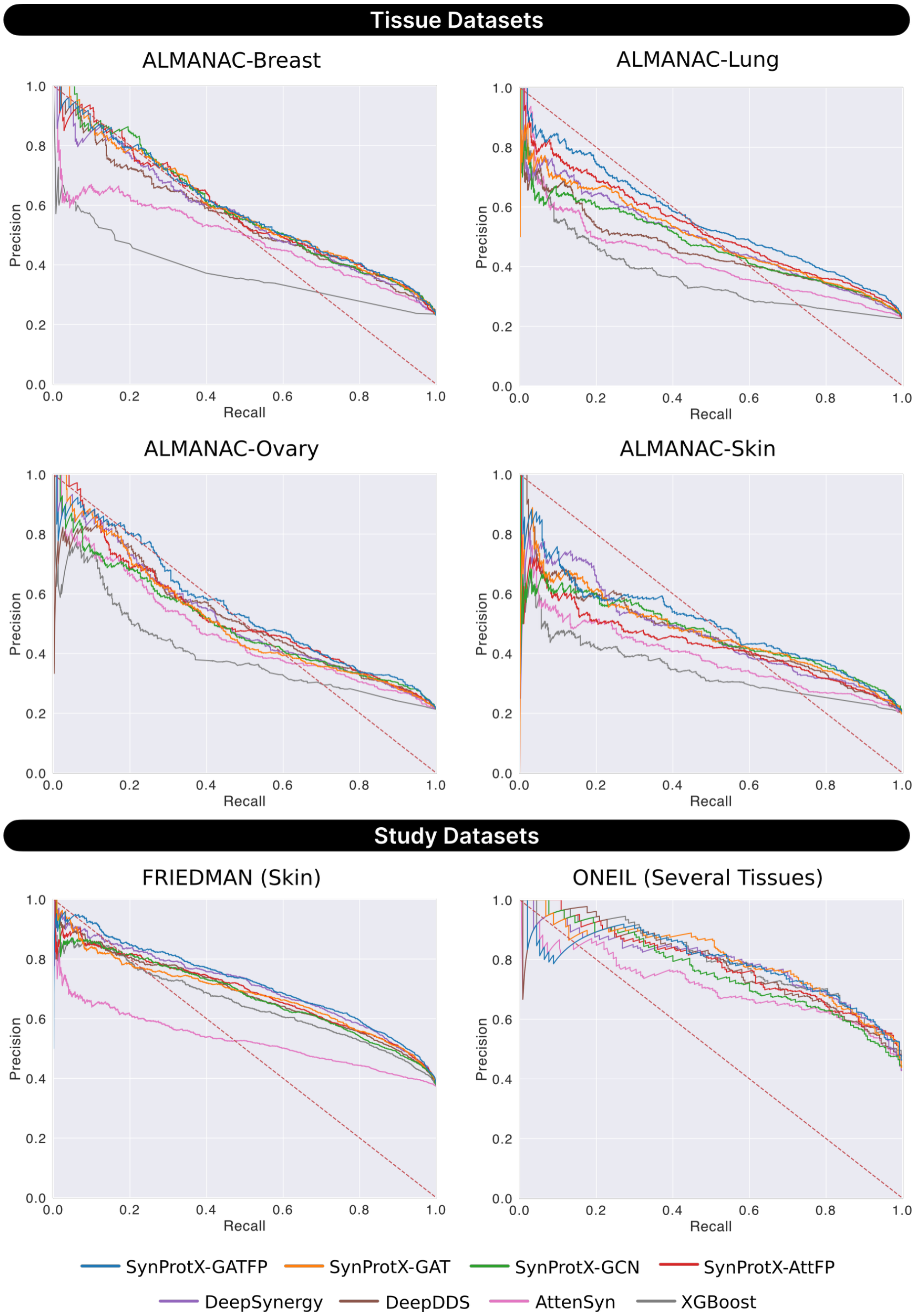

Figure S3. Comparison of precision-recall curve across SynProtX-GATFP (blue), SynProtX-GAT (orange), SynProtX-GCN (green), SynProtX-AttFP (red), DeepDDS (violet), DeepSynergy (brown), AttenSyn (pink), and XGBoost (gray) for the Tissue Datasets: (1) ALMANAC-Breast, (2) ALMANAC-Lung, (3) ALMANAC-Ovary, and (4) ALMANAC-Skin; and the Study Datasets: (1) FRIEDMAN and (2) ONEIL. The curves were conducted under the best experimental setting and best epoch for each model.

S4 Comparison of Distribution between Ground Truth and Prediction Synergy Scores

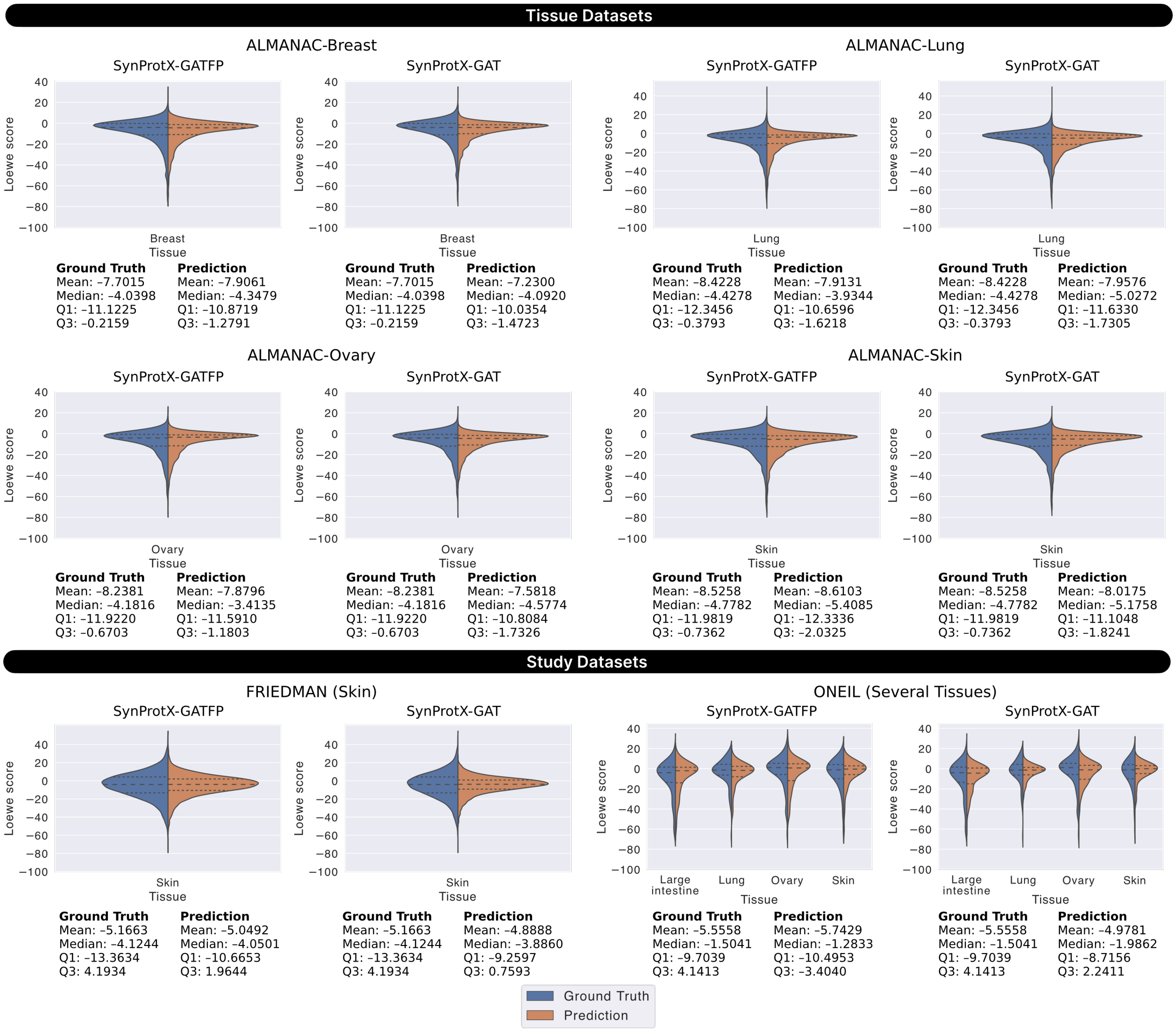

Figure S4. Comparison of distribution of ground truth and prediction synergy scores on test set across SynProtX-GATFP and SynProtX-GAT for the Tissue Datasets: (1) ALMANAC-Breast, (2) ALMANAC-Lung, (3) ALMANAC-Ovary, and (4) ALMANAC-Skin; and the Study Datasets: (1) FRIEDMAN and (2) ONEIL. The violin plots were conducted under the best experimental setting and best epoch for each model.

S5 Comparison of Regression Loss Curve

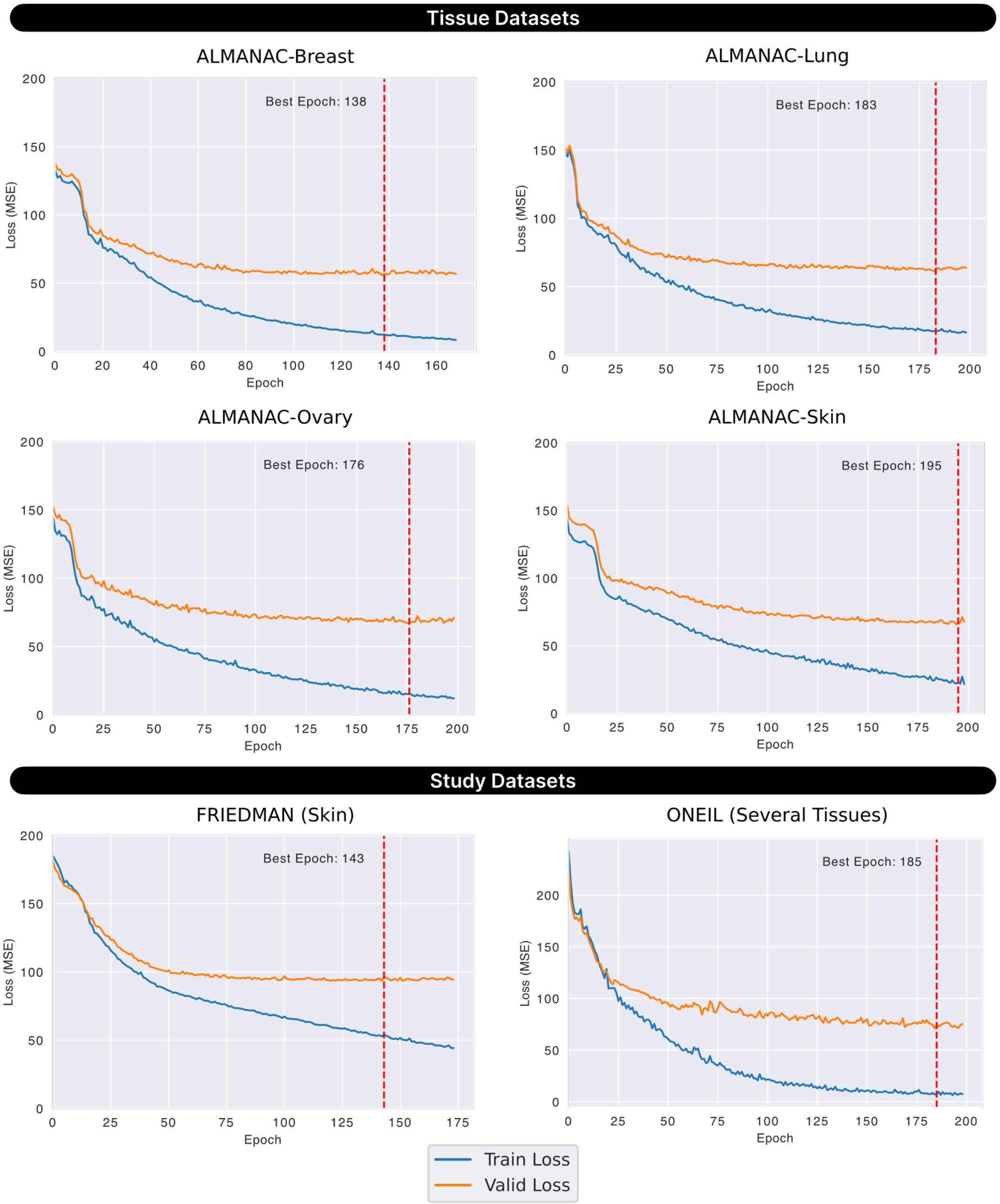

Figure S5. Comparison of regression loss curve between training set (blue) and validation set (orange) across SynProtX-GATFP for the Tissue Datasets: (1) ALMANAC-Breast, (2) ALMANAC-Lung, (3) ALMANAC-Ovary, and (4) ALMANAC-Skin; and the Study Datasets: (1) FRIEDMAN and (2) ONEIL. The loss curves were conducted under the best experimental setting for each model. The selection of the epoch for evaluation on the test set was based on achieving the lowest mean squared error (MSE) loss on the validation set (Best Epoch) monitored by early stopping mechanism.

S6 Comparison of Classification Loss Curve

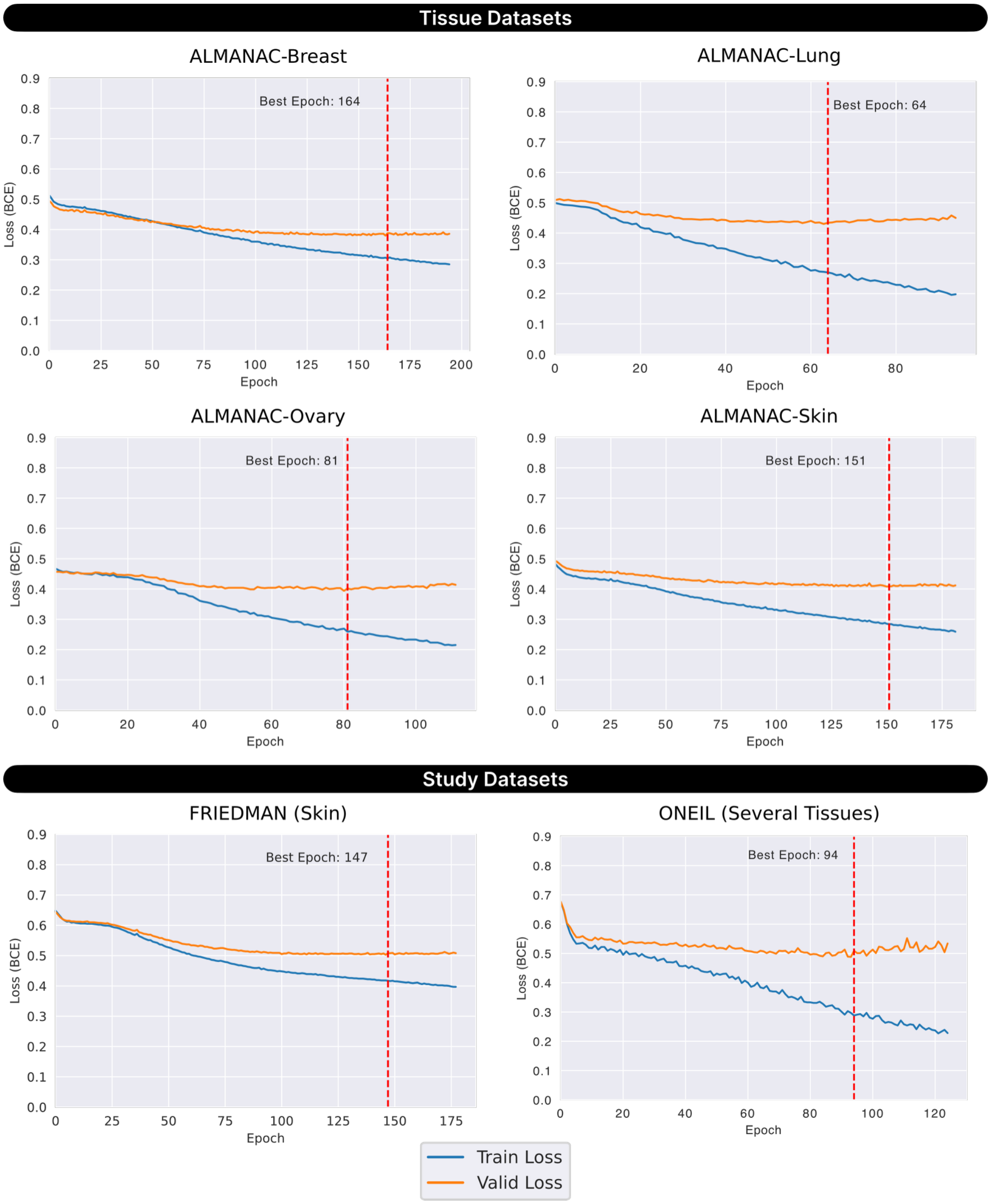

Figure S6. Comparison of classification loss curve between training set (blue) and validation set (orange) across SynProtX-GATFP for the Tissue Datasets: (1) ALMANAC-Breast, (2) ALMANAC-Lung, (3) ALMANAC-Ovary, and (4) ALMANAC-Skin; and the Study Datasets: (1) FRIEDMAN and (2) ONEIL. The loss curves were conducted under the best experimental setting for each model. The selection of the epoch for evaluation on the test set was based on achieving the lowest binary cross-entropy (BCE) loss on the validation set (Best Epoch) monitored by early stopping mechanism.

SUPPLEMENTARY TABLES

S1 Statistic of Processed Data of Anticancer Drug Combinations

Table S1 The statistic of processed data of anticancer drug combinations from DrugComb including the Tissue Datasets: (1) ALMANAC-Breast, (2) ALMANAC-Lung, (3) ALMANAC-Ovary, and (4) ALMANAC-Skin; and the Study Datasets: (1) FRIEDMAN and (2) ONEIL.

Dataset	No. drug combinations	No. unique drugs	No. cancer cell lines	Cancer cell lines	Full dose-response matrix size
**Tissue Datasets**					
ALMANAC-Breast	13,266	81	4	BT-549, MCF7, MDA-MB-231, MDA-MB-468	
ALMANAC-Lung	22,812	81	7	A549, EKVX, HOP-62, HOP-92, NCI-H226, NCI-H460, NCI-H522	4 x 4, 4 x 6
ALMANAC-Ovary	13,188	81	4	OVCAR-4, OVCAR-5, OVCAR-8, SK-OV-3	
ALMANAC-Skin	12,084	81	4	SK-MEL-2, SK-MEL-5, SK-MEL-28, UACC-257	
**Study Datasets**					
FRIEDMAN (Skin)	30,594	92	8	A2058, G-361, IPC-298, RVH-421, SK-MEL-2, SK-MEL-5, SK-MEL-28, UACC-257	3 x 3
ONEIL (Several Tissues)	2,918	32	7	A2058 (skin), NCI-H460 (lung), SK-OV-3 (ovary), A2780 (ovary), A427 (lung), RKO (large intestine), SW837 (large intestine)	5 x 5

S2 Predictive Performance on Regression Task Across Tissue and Study Datasets

Table S2 The predictive performance on regression task across the Tissue Datasets: (1) ALMANAC-Breast, (2) ALMANAC-Lung, (3) ALMANAC-Ovary, and (4) ALMANAC-Skin; and the Study Datasets: (1) FRIEDMAN and (2) ONEIL, with difference training models.

Dataset	Model	Regression Metrics				
		**RMSE (↓)**	**MAE (↓)**	**PCC (↑)**	**SCC (↑)**	**R^2 (↑)**
**Tissue Datasets**						
ALMANAC-Breast	SynProtX-GATFP	**7.876±0.050**	**5.084±0.047**	**0.792±0.003**	0.728±0.004	**0.624±0.005**
	SynProtX-GAT	8.693±0.080	5.732±0.054	0.738±0.006	0.665±0.007	0.542±0.009
	SynProtX-GCN	8.875±0.106	5.803±0.071	0.727±0.008	0.655±0.008	0.522±0.012
	SynProtX-AttFP	8.457±0.066	5.508±0.048	0.759±0.003	0.685±0.004	0.566±0.007
	DeepSynergy	8.426±0.060	5.563±0.044	0.761±0.003	0.686±0.004	0.570±0.006
	DeepDDS	8.010±0.051	5.131±0.034	0.783±0.003	**0.732±0.004**	0.611±0.005
	AttenSyn	–	–	–	–	–
	XGBoost	10.510±0.002	7.197±0.005	0.604±0.001	0.534±0.001	0.331±0.000
ALMANAC-Lung	SynProtX-GATFP	**8.299±0.047**	5.338±0.036	**0.787±0.003**	0.734±0.004	**0.616±0.004**
	SynProtX-GAT	9.307±0.099	6.162±0.065	0.722±0.007	0.663±0.007	0.517±0.010
	SynProtX-GCN	8.999±0.059	5.886±0.039	0.744±0.004	0.678±0.006	0.549±0.006
	SynProtX-AttFP	9.411±0.053	6.290±0.040	0.713±0.004	0.626±0.005	0.507±0.006
	DeepSynergy	8.812±0.034	5.830±0.022	0.757±0.002	0.679±0.003	0.567±0.003
	DeepDDS	8.392±0.043	**5.327±0.034**	0.781±0.003	**0.744±0.003**	0.608±0.004
	AttenSyn	–	–	–	–	–
	XGBoost	10.955±0.009	7.604±0.010	0.607±0.001	0.513±0.001	0.332±0.001
ALMANAC-Ovary	SynProtX-GATFP	**7.915±0.054**	**5.179±0.035**	**0.772±0.003**	**0.710±0.003**	**0.592±0.006**
	SynProtX-GAT	8.250±0.050	5.580±0.027	0.751±0.003	0.683±0.002	0.557±0.005
	SynProtX-GCN	8.785±0.118	5.976±0.091	0.707±0.010	0.621±0.012	0.498±0.014

Dataset	Model	Regression Metrics				
		RMSE (↓)	MAE (↓)	PCC (↑)	SCC (↑)	R^2 (↑)
	SynProtX-AttFP	8.463±0.052	5.564±0.034	0.738±0.004	0.678±0.005	0.534±0.006
	DeepSynergy	8.182±0.054	5.485±0.045	0.756±0.004	0.674±0.006	0.564±0.006
	DeepDDS	8.370±0.076	5.418±0.071	0.741±0.005	0.701±0.007	0.544±0.008
	AttenSyn	–	–	–	–	–
	XGBoost	10.241±0.009	7.122±0.008	0.605±0.001	0.530±0.002	0.318±0.001
ALMANAC-Skin	SynProtX-GATFP	**7.795±0.070**	**5.251±0.056**	**0.782±0.004**	**0.706±0.004**	**0.610±0.007**
	SynProtX-GAT	9.247±0.093	6.206±0.063	0.678±0.008	0.604±0.009	0.451±0.011
	SynProtX-GCN	8.567±0.071	5.888±0.057	0.728±0.005	0.634±0.007	0.529±0.008
	SynProtX-AttFP	8.961±0.081	6.148±0.079	0.700±0.007	0.596±0.011	0.484±0.009
	DeepSynergy	8.203±0.048	5.555±0.042	0.759±0.004	0.663±0.006	0.568±0.005
	DeepDDS	8.363±0.150	5.425±0.145	0.747±0.011	0.693±0.014	0.550±0.017
	AttenSyn	–	–	–	–	–
	XGBoost	10.099±0.013	6.941±0.008	0.618±0.002	0.515±0.001	0.345±0.002
**Study Datasets**						
FRIEDMAN (Skin)	SynProtX-GATFP	**9.725±0.016**	**7.470±0.011**	**0.736±0.001**	**0.695±0.001**	**0.540±0.002**
	SynProtX-GAT	10.434±0.050	8.033±0.040	0.689±0.004	0.646±0.004	0.471±0.005
	SynProtX-GCN	10.446±0.069	8.036±0.053	0.686±0.005	0.638±0.006	0.469±0.007
	SynProtX-AttFP	9.774±0.021	7.510±0.021	0.732±0.001	0.691±0.001	0.535±0.002
	DeepSynergy	9.951±0.025	7.653±0.023	0.723±0.002	0.680±0.002	0.518±0.002
	DeepDDS	10.131±0.024	7.780±0.023	0.711±0.002	0.668±0.002	0.501±0.002
	AttenSyn	–	–	–	–	–
	XGBoost	12.785±0.010	9.839±0.010	0.529±0.002	0.479±0.004	0.205±0.001
ONEIL (Several Tissues)	SynProtX-GATFP	**9.725±0.138**	**6.376±0.109**	**0.800±0.006**	**0.773±0.009**	**0.637±0.010**
	SynProtX-GAT	11.020±0.150	7.368±0.112	0.741±0.008	0.689±0.010	0.534±0.013
	SynProtX-GCN	10.540±0.183	7.148±0.151	0.759±0.009	0.684±0.012	0.574±0.015

Dataset	Model	Regression Metrics				
		RMSE (↓)	MAE (↓)	PCC (↑)	SCC (↑)	R^2 (↑)
	SynProtX-AttFP	10.330±0.188	6.990±0.117	0.774±0.009	0.721±0.012	0.591±0.015
	DeepSynergy	10.179±0.128	6.865±0.103	0.786±0.006	0.751±0.009	0.603±0.010
	DeepDDS	11.663±0.265	7.808±0.190	0.703±0.018	0.648±0.017	0.478±0.024
	AttenSyn	–	–	–	–	–
	XGBoost	10.682±0.041	7.062±0.021	0.760±0.003	0.705±0.003	0.563±0.003

Note: Values shown in bold indicate the best performance when compared to each dataset in each model. Due to AttenSyn did not report the predictive performance for the regression task, nor did it provide details on the hyperparameter settings, we have omitted including a report on the regression task.

S3 Predictive Performance on Classification Task Across Tissue and Study Datasets

Table S3 The predictive performance on classification task across the Tissue Datasets: (1) ALMANAC-Breast, (2) ALMANAC-Lung, (3) ALMANAC-Ovary, and (4) ALMANAC-Skin; and the Study Datasets: (1) FRIEDMAN and (2) ONEIL, with difference training models.

**Dataset**	**Model**	**Classification Metrics**					
		**AUROC (↑)**	**AUCPR (↑)**	**ACC (↑)**	**BACC (↑)**	**F_1 (↑)**	**KAPPA (↑)**
**Tissue Datasets**							
ALMANAC-Breast	SynProtX-GATFP	**0.793±0.003**	**0.580±0.004**	**0.804±0.002**	0.657±0.003	0.474±0.006	0.362±0.006
	SynProtX-GAT	**0.793±0.002**	0.575±0.004	0.801±0.002	**0.659±0.003**	**0.478±0.006**	**0.363±0.006**
	SynProtX-GCN	0.785±0.004	0.561±0.007	0.798±0.003	0.642±0.006	0.445±0.012	0.333±0.012
	SynProtX-AttFP	0.788±0.004	0.572±0.006	0.803±0.003	0.650±0.005	0.461±0.009	0.351±0.009
	DeepSynergy	0.767±0.006	0.546±0.007	0.797±0.002	0.644±0.008	0.449±0.018	0.335±0.014
	DeepDDS	0.770±0.004	0.533±0.007	0.792±0.002	0.650±0.007	0.461±0.012	0.339±0.011
	AttenSyn	0.734±0.007	0.438±0.013	0.759±0.006	0.628±0.009	0.424±0.016	0.274±0.016
	XGBoost	0.661±0.023	0.399±0.022	0.774±0.004	0.565±0.020	0.260±0.061	0.167±0.046
ALMANAC-Lung	SynProtX-GATFP	**0.790±0.003**	**0.544±0.005**	**0.803±0.002**	**0.644±0.005**	**0.448±0.009**	**0.338±0.009**
	SynProtX-GAT	0.745±0.006	0.466±0.009	0.785±0.002	0.595±0.009	0.342±0.021	0.236±0.018
	SynProtX-GCN	0.743±0.004	0.463±0.007	0.786±0.002	0.598±0.006	0.349±0.016	0.242±0.012
	SynProtX-AttFP	0.774±0.002	0.525±0.004	0.797±0.002	0.626±0.003	0.411±0.006	0.303±0.005
	DeepSynergy	0.745±0.003	0.475±0.006	0.785±0.003	0.619±0.007	0.398±0.017	0.279±0.013
	DeepDDS	0.729±0.004	0.445±0.007	0.782±0.002	0.584±0.007	0.314±0.019	0.211±0.014
	AttenSyn	0.696±0.004	0.407±0.009	0.762±0.004	0.594±0.008	0.353±0.018	0.216±0.014
	XGBoost	0.628±0.005	0.369±0.001	0.777±0.001	0.546±0.000	0.202±0.001	0.127±0.001
ALMANAC-Ovary	SynProtX-GATFP	**0.781±0.003**	**0.542±0.004**	**0.817±0.002**	**0.628±0.004**	**0.411±0.010**	**0.321±0.008**
	SynProtX-GAT	0.743±0.005	0.483±0.009	0.804±0.002	0.599±0.010	0.340±0.027	0.251±0.021
	SynProtX-GCN	0.746±0.013	0.477±0.020	0.803±0.004	0.561±0.013	0.223±0.045	0.168±0.035

Dataset	Model	Classification Metrics					
		**AUROC (↑)**	**AUCPR (↑)**	**ACC (↑)**	**BACC (↑)**	**F_1 (↑)**	**KAPPA (↑)**
	SynProtX-AttFP	0.768±0.002	0.514±0.005	0.810±0.002	0.597±0.006	0.336±0.015	0.256±0.012
	DeepSynergy	0.745±0.005	0.496±0.006	0.808±0.001	0.605±0.009	0.355±0.023	0.268±0.017
	DeepDDS	0.751±0.003	0.494±0.006	0.806±0.002	0.619±0.005	0.392±0.010	0.293±0.009
	AttenSyn	0.717±0.007	0.441±0.012	0.781±0.004	0.626±0.009	0.410±0.017	0.279±0.016
	XGBoost	0.677±0.002	0.399±0.002	0.796±0.000	0.560±0.000	0.235±0.001	0.164±0.000
ALMANAC-Skin	SynProtX-GATFP	**0.786±0.003**	**0.481±0.006**	**0.815±0.002**	**0.614±0.007**	**0.375±0.015**	**0.280±0.014**
	SynProtX-GAT	0.761±0.004	0.447±0.008	0.808±0.002	0.596±0.008	0.334±0.019	0.240±0.017
	SynProtX-GCN	0.781±0.003	0.459±0.005	0.810±0.002	0.575±0.006	0.278±0.016	0.201±0.013
	SynProtX-AttFP	0.740±0.008	0.411±0.010	0.805±0.002	0.559±0.009	0.232±0.028	0.160±0.022
	DeepSynergy	0.754±0.005	0.447±0.008	0.808±0.003	0.603±0.011	0.348±0.026	0.253±0.020
	DeepDDS	0.755±0.004	0.435±0.009	0.802±0.003	0.602±0.009	0.350±0.020	0.246±0.017
	AttenSyn	0.713±0.010	0.379±0.017	0.779±0.006	0.603±0.010	0.359±0.019	0.229±0.017
	XGBoost	0.670±0.003	0.346±0.004	0.804±0.000	0.518±0.001	0.083±0.005	0.056±0.003
**Study Datasets**							
FRIEDMAN (Skin)	SynProtX-GATFP	**0.810±0.001**	**0.710±0.003**	**0.747±0.001**	**0.715±0.002**	**0.635±0.003**	**0.443±0.003**
	SynProtX-GAT	0.785±0.003	0.670±0.005	0.724±0.003	0.671±0.004	0.557±0.008	0.367±0.008
	SynProtX-GCN	0.772±0.003	0.657±0.004	0.718±0.002	0.679±0.004	0.582±0.007	0.373±0.006
	SynProtX-AttFP	0.783±0.003	0.675±0.004	0.726±0.003	0.685±0.003	0.588±0.005	0.386±0.006
	DeepSynergy	0.803±0.002	0.701±0.002	0.742±0.002	0.709±0.004	0.626±0.006	0.430±0.006
	DeepDDS	0.775±0.003	0.666±0.003	0.721±0.002	0.680±0.005	0.580±0.010	0.376±0.008
	AttenSyn	0.636±0.005	0.500±0.007	0.637±0.005	0.586±0.004	0.442±0.012	0.181±0.009
	XGBoost	0.762±0.001	0.651±0.002	0.702±0.003	0.627±0.004	0.453±0.007	0.285±0.008
ONEIL (Several Tissues)	SynProtX-GATFP	0.831±0.004	0.777±0.005	0.744±0.007	0.743±0.006	0.710±0.007	0.482±0.013
	SynProtX-GAT	0.825±0.006	0.775±0.007	0.742±0.009	0.739±0.007	0.706±0.007	0.476±0.016
	SynProtX-GCN	0.793±0.002	0.748±0.003	0.713±0.003	0.707±0.005	0.663±0.010	0.414±0.009
	SynProtX-AttFP	0.812±0.005	0.754±0.006	0.724±0.007	0.721±0.008	0.683±0.010	0.439±0.014

Dataset	Model	Classification Metrics					
		**AUROC (↑)**	**AUCPR (↑)**	**ACC (↑)**	**BACC (↑)**	**F_1 (↑)**	**KAPPA (↑)**
	DeepSynergy	0.831±0.003	0.791±0.004	0.752±0.005	0.747±0.005	0.710±0.007	0.494±0.010
	DeepDDS	0.801±0.005	0.761±0.004	0.727±0.005	0.720±0.005	0.679±0.007	0.441±0.009
	AttenSyn	0.769±0.008	0.701±0.016	0.702±0.011	0.701±0.010	0.665±0.014	0.398±0.021
	XGBoost	**0.842±0.002**	**0.801±0.002**	**0.758±0.006**	**0.757±0.007**	**0.726±0.007**	**0.510±0.013**

Note: Values shown in bold indicate the best performance when compared to each dataset in each model.

S4 Predictive Performance on Regression Task Across Cell Lines of ALMANAC-Breast Tissue Dataset

Table S4 The predictive performance on regression task across cell lines of ALMANAC-Breast tissue dataset, with difference training models.

Cell Line	SynProtX-GATFP	SynProtX-GAT	SynProtX-GCN	SynProtX-AttFP	DeepSynergy	DeepDDS	AttenSyn	XGBoost
**RMSE**								
BT-549	**7.231±0.195**	8.084±0.235	8.321±0.334	7.767±0.312	8.039±0.286	7.551±0.245	–	9.714±0.020
MCF7	7.495±0.225	8.090±0.192	8.253±0.242	7.848±0.195	7.655±0.265	**7.480±0.194**	–	8.961±0.032
MDA-MB-231	**7.744±0.137**	8.467±0.236	8.692±0.321	8.136±0.327	8.206±0.237	7.952±0.231	–	9.584±0.011
MDA-MB-468	**8.474±0.219**	9.402±0.266	9.594±0.339	9.442±0.208	9.230±0.338	8.537±0.144	–	12.542±0.025
**MAE**								
BT-549	**4.686±0.127**	5.399±0.168	5.449±0.200	5.073±0.176	5.271±0.180	4.867±0.130	–	6.922±0.010
MCF7	4.652±0.135	5.007±0.112	5.121±0.151	4.946±0.107	4.900±0.116	**4.570±0.087**	–	6.041±0.021
MDA-MB-231	**5.002±0.094**	5.617±0.178	5.739±0.178	5.377±0.208	5.455±0.150	5.092±0.126	–	6.689±0.007
MDA-MB-468	**5.472±0.120**	6.200±0.132	6.288±0.238	6.067±0.098	6.088±0.216	5.513±0.133	–	8.647±0.009
**PCC**								
BT-549	**0.814±0.011**	0.757±0.017	0.748±0.024	0.789±0.016	0.774±0.014	0.795±0.014	–	0.655±0.003
MCF7	0.752±0.018	0.707±0.019	0.690±0.023	0.725±0.016	0.740±0.023	**0.755±0.014**	–	0.650±0.005
MDA-MB-231	**0.758±0.009**	0.692±0.019	0.678±0.030	0.732±0.019	0.729±0.012	0.739±0.016	–	0.578±0.002
MDA-MB-468	**0.822±0.010**	0.775±0.015	0.766±0.020	0.780±0.010	0.788±0.014	0.819±0.006	–	0.560±0.004

Note: Values shown in bold indicate the best performance when compared to each metric in each model. Due to AttenSyn did not report the predictive performance for the regression task, nor did it provide details on the hyperparameter settings, we have omitted including a report on the regression task.

S5 Predictive Performance on Regression Task Across Cell Lines of ALMANAC-Lung Tissue Dataset

Table S5 The predictive performance on regression task across cell lines of ALMANAC-Lung tissue dataset, with difference training models.

Cell Line	SynProtX-GATFP	SynProtX-GAT	SynProtX-GCN	SynProtX-AttFP	DeepSynergy	DeepDDS	AttenSyn	XGBoost
**RMSE**								
A549	**7.894±0.213**	9.040±0.276	8.682±0.248	9.725±0.135	8.675±0.245	8.246±0.161	–	11.195±0.016
EKVX	**6.302±0.183**	7.416±0.258	6.986±0.232	7.499±0.187	6.822±0.166	6.304±0.174	–	9.059±0.013
HOP-62	**10.056±0.309**	10.711±0.240	10.515±0.264	10.456±0.150	10.378±0.187	10.162±0.189	–	12.437±0.017
HOP-92	**8.728±0.181**	9.581±0.238	9.065±0.254	9.370±0.128	9.102±0.162	8.824±0.221	–	10.978±0.017
NCI-H226	**8.222±0.228**	9.146±0.445	9.192±0.329	9.326±0.168	8.724±0.231	8.596±0.361	–	10.145±0.009
NCI-H460	7.534±0.148	8.944±0.249	8.527±0.239	9.206±0.193	8.279±0.116	**7.461±0.219**	–	11.025±0.020
NCI-H522	8.072±0.258	9.034±0.224	8.753±0.242	9.030±0.155	8.509±0.193	**7.922±0.183**	–	10.337±0.045
**MAE**								
A549	**4.952±0.144**	5.903±0.227	5.494±0.167	6.224±0.119	5.482±0.147	5.030±0.121	–	7.831±0.017
EKVX	**4.041±0.106**	4.835±0.121	4.460±0.114	4.831±0.088	4.487±0.104	4.121±0.137	–	5.979±0.005
HOP-62	6.329±0.136	7.056±0.170	6.831±0.165	6.996±0.131	6.694±0.127	**6.328±0.140**	–	8.577±0.012
HOP-92	**5.432±0.130**	6.093±0.133	5.739±0.137	6.058±0.083	5.850±0.077	5.530±0.134	–	7.279±0.008
NCI-H226	**5.226±0.136**	5.860±0.232	5.836±0.178	6.300±0.145	5.765±0.200	5.240±0.175	–	6.841±0.014
NCI-H460	5.206±0.079	6.310±0.236	5.934±0.157	6.454±0.121	5.877±0.107	**5.053±0.124**	–	8.377±0.003
NCI-H522	**5.146±0.158**	6.079±0.146	5.798±0.153	6.189±0.114	5.640±0.129	5.162±0.102	–	7.640±0.031
**PCC**								
A549	**0.813±0.011**	0.744±0.017	0.769±0.015	0.695±0.009	0.773±0.015	0.793±0.008	–	0.578±0.002
EKVX	**0.802±0.014**	0.707±0.025	0.746±0.018	0.694±0.019	0.761±0.014	0.797±0.013	–	0.532±0.004
HOP-62	**0.747±0.016**	0.700±0.016	0.718±0.015	0.717±0.011	0.734±0.008	0.738±0.011	–	0.592±0.003
HOP-92	**0.750±0.012**	0.690±0.019	0.727±0.018	0.708±0.010	0.727±0.011	0.741±0.016	–	0.589±0.003
NCI-H226	**0.744±0.015**	0.665±0.040	0.667±0.025	0.648±0.016	0.711±0.015	0.714±0.026	–	0.582±0.002

Cell Line	SynProtX-GATFP	SynProtX-GAT	SynProtX-GCN	SynProtX-AttFP	DeepSynergy	DeepDDS	AttenSyn	XGBoost
NCI-H460	0.849±0.007	0.780±0.014	0.800±0.013	0.761±0.012	0.813±0.006	**0.851±0.010**	–	0.668±0.001
NCI-H522	0.787±0.015	0.722±0.015	0.744±0.015	0.725±0.010	0.766±0.010	**0.795±0.010**	–	0.641±0.005

Note: Values shown in bold indicate the best performance when compared to each metric in each model. Due to AttenSyn did not report the predictive performance for the regression task, nor did it provide details on the hyperparameter settings, we have omitted including a report on the regression task.

S6 Predictive Performance on Regression Task Across Cell Lines of ALMANAC-Ovary Tissue Dataset

Table S6 The predictive performance on regression task across cell lines of ALMANAC-Ovary tissue dataset, with difference training models.

Cell Line	SynProtX-GATFP	SynProtX-GAT	SynProtX-GCN	SynProtX-AttFP	DeepSynergy	DeepDDS	AttenSyn	XGBoost
**RMSE**								
OVCAR-4	**7.300±0.220**	7.465±0.128	8.236±0.281	7.982±0.203	7.681±0.233	7.626±0.221	–	8.705±0.027
OVCAR-5	**8.198±0.231**	8.568±0.161	8.809±0.301	8.593±0.259	8.307±0.213	8.826±0.199	–	10.359±0.026
OVCAR-8	**8.500±0.223**	8.676±0.203	9.308±0.287	9.165±0.251	8.868±0.334	9.108±0.303	–	11.352±0.016
SK-OV-3	**7.052±0.115**	7.576±0.157	8.125±0.315	7.521±0.145	7.255±0.123	7.246±0.234	–	9.579±0.004
**MAE**								
OVCAR-4	**4.523±0.146**	4.904±0.132	5.425±0.216	4.932±0.155	4.891±0.167	4.750±0.150	–	6.124±0.012
OVCAR-5	**5.159±0.134**	5.505±0.110	5.847±0.166	5.528±0.125	5.368±0.151	5.530±0.096	–	7.168±0.016
OVCAR-8	**5.691±0.118**	6.080±0.150	6.468±0.234	6.175±0.146	6.095±0.190	6.000±0.227	–	8.151±0.006
SK-OV-3	**4.775±0.071**	5.241±0.115	5.581±0.242	5.064±0.113	5.016±0.126	4.842±0.194	–	6.656±0.010
**PCC**								
OVCAR-4	**0.776±0.008**	0.748±0.010	0.697±0.023	0.740±0.011	0.757±0.011	0.749±0.011	–	0.654±0.004
OVCAR-5	**0.745±0.017**	0.730±0.012	0.699±0.026	0.715±0.019	0.740±0.011	0.695±0.017	–	0.570±0.005
OVCAR-8	**0.773±0.011**	0.759±0.013	0.716±0.019	0.736±0.016	0.754±0.019	0.736±0.017	–	0.587±0.001
SK-OV-3	**0.803±0.007**	0.777±0.013	0.726±0.026	0.774±0.010	0.792±0.006	0.790±0.016	–	0.622±0.002

Note: Values shown in bold indicate the best performance when compared to each metric in each model. Due to AttenSyn did not report the predictive performance for the regression task, nor did it provide details on the hyperparameter settings, we have omitted including a report on the regression task.

S7 Predictive Performance on Regression Task Across Cell Lines of ALMANAC-Skin Tissue Dataset

Table S7 The predictive performance on regression task across cell lines of ALMANAC-Skin tissue dataset, with difference training models.

Cell Line	SynProtX-GATFP	SynProtX-GAT	SynProtX-GCN	SynProtX-AttFP	DeepSynergy	DeepDDS	AttenSyn	XGBoost
**RMSE**								
SK-MEL-2	**7.388±0.248**	9.293±0.330	8.436±0.198	8.883±0.394	7.855±0.264	8.036±0.616	–	10.601±0.014
SK-MEL-28	**7.514±0.218**	8.640±0.258	8.116±0.150	8.332±0.142	8.006±0.191	7.986±0.274	–	9.074±0.033
SK-MEL-5	**9.432±0.132**	11.041±0.275	10.213±0.187	10.562±0.249	9.818±0.123	10.126±0.324	–	11.942±0.001
UACC-257	**6.018±0.161**	7.187±0.340	6.763±0.312	7.357±0.264	6.325±0.186	6.513±0.406	–	7.918±0.010
**MAE**								
SK-MEL-2	**5.407±0.174**	6.553±0.221	6.144±0.151	6.342±0.339	5.600±0.151	5.690±0.494	–	7.776±0.022
SK-MEL-28	**4.979±0.165**	5.743±0.169	5.552±0.163	5.687±0.094	5.336±0.140	5.163±0.225	–	6.190±0.015
SK-MEL-5	**6.203±0.117**	7.392±0.203	6.914±0.172	7.217±0.221	6.537±0.144	6.470±0.282	–	8.076±0.004
UACC-257	**4.097±0.114**	4.779±0.183	4.674±0.179	4.969±0.173	4.352±0.102	4.124±0.319	–	5.696±0.007
**PCC**								
SK-MEL-2	**0.863±0.010**	0.774±0.018	0.818±0.009	0.796±0.020	0.848±0.009	0.834±0.030	–	0.741±0.002
SK-MEL-28	**0.737±0.016**	0.634±0.026	0.680±0.016	0.660±0.014	0.703±0.015	0.700±0.021	–	0.581±0.005
SK-MEL-5	**0.738±0.010**	0.616±0.024	0.680±0.014	0.653±0.021	0.711±0.009	0.690±0.023	–	0.554±0.001
UACC-257	**0.783±0.011**	0.671±0.030	0.715±0.022	0.667±0.018	0.766±0.012	0.755±0.031	–	0.551±0.002

Note: Values shown in bold indicate the best performance when compared to each metric in each model. Due to AttenSyn did not report the predictive performance for the regression task, nor did it provide details on the hyperparameter settings, we have omitted including a report on the regression task.

S8 Predictive Performance on Regression Task Across Cell Lines of FRIEDMAN Study Dataset

Table S8 The predictive performance on regression task across cell lines of FRIEDMAN study dataset, with difference training models.

Cell Line	SynProtX-GATFP	SynProtX-GAT	SynProtX-GCN	SynProtX-AttFP	DeepSynergy	DeepDDS	AttenSyn	XGBoost
**RMSE**								
A2058	**9.163±0.089**	9.914±0.129	9.854±0.206	9.169±0.064	9.350±0.124	9.550±0.116	–	11.471±0.003
G-361	**8.791±0.076**	9.324±0.170	9.625±0.145	8.896±0.095	9.209±0.071	9.193±0.158	–	11.488±0.014
IPC-298	**7.563±0.126**	8.544±0.244	8.550±0.186	7.851±0.100	7.874±0.121	7.914±0.269	–	10.829±0.010
RVH-421	12.145±0.173	12.598±0.209	12.656±0.346	**11.969±0.094**	12.359±0.105	12.539±0.177	–	14.660±0.018
SK-MEL-2	**9.134±0.108**	9.700±0.228	9.706±0.109	9.270±0.089	9.322±0.125	9.472±0.127	–	12.022±0.012
SK-MEL-28	10.423±0.114	11.209±0.282	10.957±0.274	**10.322±0.105**	10.463±0.171	10.893±0.183	–	15.641±0.015
SK-MEL-5	**9.009±0.093**	9.985±0.196	9.938±0.161	9.171±0.123	9.492±0.176	9.738±0.204	–	11.444±0.015
UACC-257	**8.702±0.066**	9.061±0.143	9.417±0.236	8.697±0.075	8.814±0.135	8.882±0.131	–	10.248±0.014
**MAE**								
A2058	**7.089±0.085**	7.575±0.113	7.600±0.166	7.087±0.074	7.197±0.099	7.361±0.112	–	8.887±0.004
G-361	**6.692±0.061**	7.106±0.096	7.288±0.112	6.751±0.074	7.022±0.055	7.011±0.098	–	8.872±0.007
IPC-298	**5.949±0.088**	6.707±0.221	6.738±0.170	6.179±0.086	6.199±0.102	6.174±0.212	–	8.411±0.007
RVH-421	9.295±0.140	9.585±0.206	9.646±0.251	**9.119±0.091**	9.437±0.123	9.636±0.164	–	11.189±0.016
SK-MEL-2	**6.979±0.079**	7.372±0.180	7.376±0.073	7.042±0.095	7.142±0.112	7.222±0.116	–	8.980±0.004
SK-MEL-28	8.125±0.103	8.727±0.209	8.484±0.224	**8.028±0.103**	8.181±0.158	8.494±0.185	–	12.072±0.012
SK-MEL-5	**6.986±0.098**	7.752±0.145	7.694±0.149	7.077±0.097	7.386±0.150	7.512±0.185	–	8.936±0.010
UACC-257	6.789±0.052	7.105±0.149	7.339±0.161	**6.782±0.087**	6.876±0.117	6.929±0.104	–	7.954±0.011
**PCC**								
A2058	**0.702±0.006**	0.638±0.015	0.644±0.017	0.698±0.005	0.695±0.009	0.673±0.009	–	0.511±0.001
G-361	**0.735±0.006**	0.693±0.016	0.675±0.010	0.724±0.006	0.709±0.007	0.714±0.009	–	0.514±0.003

Cell Line	SynProtX-GATFP	SynProtX-GAT	SynProtX-GCN	SynProtX-AttFP	DeepSynergy	DeepDDS	AttenSyn	XGBoost
IPC-298	**0.817±0.007**	0.771±0.015	0.760±0.013	0.805±0.005	0.801±0.006	0.798±0.017	–	0.666±0.001
RVH-421	0.668±0.009	0.638±0.018	0.630±0.026	**0.676±0.007**	0.660±0.007	0.646±0.011	–	0.541±0.003
SK-MEL-2	**0.741±0.007**	0.704±0.017	0.700±0.008	0.731±0.006	0.733±0.006	0.720±0.008	–	0.539±0.002
SK-MEL-28	0.800±0.005	0.768±0.013	0.776±0.013	**0.803±0.004**	0.798±0.007	0.780±0.008	–	0.486±0.004
SK-MEL-5	**0.720±0.007**	0.641±0.018	0.647±0.015	0.708±0.008	0.687±0.012	0.668±0.015	–	0.546±0.002
UACC-257	**0.627±0.007**	0.574±0.016	0.541±0.023	0.620±0.008	0.625±0.010	0.609±0.012	–	0.390±0.005

Note: Values shown in bold indicate the best performance when compared to each metric in each model. Due to AttenSyn did not report the predictive performance for the regression task, nor did it provide details on the hyperparameter settings, we have omitted including a report on the regression task.

S9 Predictive Performance on Regression Task Across Cell Lines of ONEIL Study Dataset

Table S9 The predictive performance on regression task across cell lines of ONEIL study dataset, with difference training models.

Cell Line	SynProtX-GATFP	SynProtX-GAT	SynProtX-GCN	SynProtX-AttFP	DeepSynergy	DeepDDS	AttenSyn	XGBoost
**RMSE**								
A2058	8.339±0.397	9.255±0.632	9.696±0.657	8.389±0.908	**7.774±0.634**	9.401±0.575	–	8.262±0.031
A2780	7.868±0.503	8.578±0.870	8.439±0.785	8.169±0.574	**7.728±0.537**	9.968±1.104	–	8.585±0.074
A427	5.915±0.455	7.648±0.578	7.233±0.611	6.594±0.659	**5.910±0.492**	8.039±1.116	–	6.897±0.045
NCI-H460	**8.881±0.659**	10.481±0.483	9.720±0.530	10.374±0.629	9.484±0.537	10.538±0.636	–	9.165±0.048
RKO	**9.383±0.917**	10.014±0.402	9.544±0.788	9.626±0.845	10.197±0.642	10.928±0.668	–	10.170±0.065
SK-OV-3	**9.767±0.806**	12.635±0.746	11.121±1.082	10.835±1.080	11.591±0.807	13.622±1.165	–	12.939±0.097
SW837	13.769±0.526	14.098±0.346	14.137±0.628	14.088±0.590	13.892±0.570	14.824±0.643	–	**13.636±0.026**
**MAE**								
A2058	**5.667±0.267**	6.604±0.520	6.800±0.451	6.024±0.595	5.692±0.450	6.896±0.411	–	6.083±0.019
A2780	**5.208±0.390**	5.973±0.536	5.533±0.498	5.534±0.273	5.411±0.440	6.786±0.927	–	5.885±0.067
A427	4.178±0.308	5.426±0.451	5.073±0.429	4.640±0.401	**4.145±0.268**	5.586±0.759	–	4.774±0.009
NCI-H460	6.217±0.482	6.758±0.266	6.796±0.330	7.393±0.515	6.634±0.418	7.147±0.508	–	**6.199±0.023**
RKO	**6.308±0.560**	6.825±0.344	6.544±0.502	6.745±0.525	7.471±0.515	7.678±0.311	–	6.779±0.033
SK-OV-3	**6.684±0.534**	8.486±0.404	7.797±0.651	7.695±0.679	7.874±0.550	8.756±0.574	–	8.317±0.046
SW837	**8.616±0.561**	9.159±0.371	9.325±0.567	9.166±0.386	9.264±0.455	9.692±0.514	–	8.827±0.031
**PCC**								
A2058	0.822±0.018	0.808±0.021	0.757±0.030	0.825±0.041	**0.855±0.022**	0.767±0.036	–	0.838±0.001
A2780	0.809±0.024	0.755±0.067	0.771±0.045	0.802±0.026	**0.812±0.026**	0.676±0.077	–	0.752±0.005
A427	**0.874±0.021**	0.800±0.030	0.801±0.041	0.835±0.036	0.871±0.023	0.767±0.058	–	0.842±0.003

**Cell Line**	**SynProtX-GATFP**	**SynProtX-GAT**	**SynProtX-GCN**	**SynProtX-AttFP**	**DeepSynergy**	**DeepDDS**	**AttenSyn**	**XGBoost**
NCI-H460	**0.791±0.033**	0.710±0.033	0.742±0.035	0.704±0.044	0.757±0.030	0.696±0.041	–	0.787±0.004
RKO	**0.843±0.030**	0.813±0.016	0.836±0.024	0.832±0.024	0.825±0.023	0.787±0.016	–	0.804±0.003
SK-OV-3	**0.839±0.032**	0.716±0.046	0.780±0.050	0.793±0.048	0.767±0.032	0.663±0.060	–	0.706±0.006
SW837	0.685±0.027	0.651±0.017	0.649±0.032	0.674±0.029	**0.693±0.027**	0.625±0.032	–	0.681±0.002

Note: Values shown in bold indicate the best performance when compared to each metric in each model. Due to AttenSyn did not report the predictive performance for the regression task, nor did it provide details on the hyperparameter settings, we have omitted including a report on the regression task.

S10 Predictive Performance on Classification Task Across Cell Lines of ALMANAC-Breast Tissue Dataset

Table S10 The predictive performance on classification task across cell lines of ALMANAC-Breast tissue dataset, with difference training models.

Cell Line	SynProtX-GATFP	SynProtX-GAT	SynProtX-GCN	SynProtX-AttFP	DeepSynergy	DeepDDS	AttenSyn	XGBoost
**AUROC**								
BT-549	**0.806±0.009**	0.798±0.008	0.793±0.009	0.794±0.012	0.780±0.017	0.776±0.013	0.734±0.017	0.655±0.004
MCF7	0.803±0.009	**0.812±0.006**	0.798±0.010	0.809±0.009	0.772±0.011	0.792±0.013	0.750±0.016	0.665±0.015
MDA-MB-231	0.764±0.008	**0.769±0.008**	0.759±0.017	0.755±0.017	0.731±0.014	0.729±0.017	0.691±0.017	0.593±0.025
MDA-MB-468	**0.805±0.006**	0.799±0.005	0.792±0.009	0.797±0.008	0.788±0.009	0.788±0.007	0.771±0.007	0.660±0.008
**AUCPR**								
BT-549	**0.595±0.014**	0.573±0.014	0.538±0.020	0.552±0.028	0.551±0.023	0.526±0.021	0.440±0.027	0.405±0.021
MCF7	**0.551±0.016**	0.542±0.013	0.532±0.018	0.548±0.017	0.498±0.022	0.503±0.025	0.427±0.030	0.378±0.033
MDA-MB-231	0.469±0.015	0.481±0.020	0.471±0.031	**0.494±0.036**	0.448±0.028	0.429±0.032	0.364±0.027	0.291±0.019
MDA-MB-468	**0.671±0.008**	0.670±0.006	0.664±0.014	0.664±0.016	0.656±0.008	0.639±0.011	0.583±0.015	0.501±0.002
**ACC**								
BT-549	**0.821±0.006**	0.813±0.006	0.808±0.007	0.810±0.011	0.816±0.009	0.805±0.007	0.787±0.006	0.784±0.007
MCF7	0.806±0.006	0.809±0.007	0.811±0.011	**0.814±0.007**	0.802±0.007	0.800±0.011	0.796±0.010	0.791±0.010
MDA-MB-231	0.802±0.008	0.798±0.009	0.790±0.009	**0.803±0.012**	0.794±0.011	0.787±0.010	0.779±0.010	0.774±0.011
MDA-MB-468	**0.787±0.008**	0.786±0.006	0.784±0.008	**0.787±0.008**	0.777±0.005	0.777±0.008	0.761±0.007	0.743±0.001
**BACC**								
BT-549	**0.661±0.010**	0.651±0.018	0.640±0.016	0.644±0.021	0.644±0.028	0.640±0.021	0.598±0.018	0.552±0.009
MCF7	0.640±0.011	0.652±0.014	0.637±0.016	0.648±0.015	0.636±0.015	**0.654±0.020**	0.611±0.021	0.570±0.015
MDA-MB-231	**0.639±0.018**	0.638±0.016	0.615±0.021	0.632±0.025	0.618±0.026	0.627±0.023	0.574±0.017	0.529±0.020
MDA-MB-468	0.678±0.009	**0.684±0.009**	0.667±0.012	0.669±0.012	0.668±0.013	0.673±0.015	0.625±0.017	0.571±0.009

Note: Values shown in bold indicate the best performance when compared to each metric in each model.

S11 Predictive Performance on Classification Task Across Cell Lines of ALMANAC-Lung Tissue Dataset

Table S11 The predictive performance on classification task across cell lines of ALMANAC-Lung tissue dataset, with difference training models.

Cell Line	SynProtX-GATFP	SynProtX-GAT	SynProtX-GCN	SynProtX-AttFP	DeepSynergy	DeepDDS	AttenSyn	XGBoost
**AUROC**								
A549	**0.829±0.014**	0.783±0.026	0.743±0.019	0.796±0.005	0.778±0.015	0.748±0.019	0.728±0.018	0.628±0.001
EKVX	**0.759±0.015**	0.702±0.022	0.708±0.021	0.739±0.012	0.732±0.010	0.684±0.019	0.622±0.015	0.554±0.001
HOP-62	**0.809±0.014**	0.772±0.014	0.788±0.015	0.801±0.009	0.767±0.009	0.757±0.014	0.723±0.014	0.702±0.002
HOP-92	**0.787±0.008**	0.758±0.009	0.746±0.013	0.764±0.009	0.737±0.012	0.741±0.011	0.727±0.015	0.625±0.002
NCI-H226	**0.813±0.010**	0.760±0.017	0.751±0.011	0.801±0.010	0.773±0.011	0.758±0.011	0.697±0.014	0.654±0.005
NCI-H460	**0.786±0.013**	0.734±0.018	0.742±0.009	0.774±0.008	0.717±0.009	0.723±0.017	0.668±0.014	0.639±0.005
NCI-H522	**0.753±0.008**	0.730±0.012	0.731±0.010	0.751±0.009	0.726±0.007	0.704±0.009	0.682±0.014	0.591±0.002
**AUCPR**								
A549	**0.594±0.029**	0.499±0.055	0.471±0.028	0.528±0.022	0.482±0.026	0.434±0.037	0.416±0.029	0.361±0.001
EKVX	**0.523±0.024**	0.460±0.027	0.471±0.026	0.514±0.022	0.452±0.019	0.450±0.023	0.366±0.017	0.317±0.001
HOP-62	**0.553±0.036**	0.473±0.025	0.493±0.032	0.541±0.025	0.463±0.022	0.462±0.027	0.412±0.025	0.410±0.001
HOP-92	**0.511±0.016**	0.464±0.018	0.466±0.020	0.479±0.013	0.460±0.015	0.450±0.012	0.432±0.021	0.370±0.003
NCI-H226	**0.639±0.020**	0.539±0.028	0.522±0.017	0.621±0.016	0.582±0.015	0.523±0.031	0.454±0.018	0.431±0.002
NCI-H460	**0.515±0.035**	0.414±0.028	0.422±0.020	0.494±0.026	0.454±0.015	0.395±0.026	0.365±0.019	0.367±0.004
NCI-H522	**0.502±0.015**	0.455±0.019	0.437±0.023	0.508±0.018	0.466±0.020	0.430±0.013	0.417±0.018	0.353±0.002
**ACC**								
A549	**0.831±0.010**	0.812±0.012	0.808±0.008	0.818±0.007	0.804±0.010	0.801±0.008	0.797±0.008	0.788±0.000
EKVX	0.782±0.007	0.774±0.010	0.774±0.008	**0.785±0.010**	0.761±0.011	0.772±0.011	0.757±0.008	0.761±0.001
HOP-62	**0.829±0.012**	0.823±0.008	0.822±0.010	0.824±0.008	0.816±0.006	0.820±0.010	0.806±0.005	0.816±0.000

Cell Line	SynProtX-GATFP	SynProtX-GAT	SynProtX-GCN	SynProtX-AttFP	DeepSynergy	DeepDDS	AttenSyn	XGBoost
HOP-92	**0.785±0.005**	0.774±0.006	0.774±0.005	0.778±0.008	0.768±0.011	0.773±0.005	0.770±0.009	0.765±0.001
NCI-H226	**0.819±0.010**	0.781±0.010	0.781±0.008	0.806±0.006	0.805±0.010	0.777±0.012	0.766±0.008	0.770±0.000
NCI-H460	**0.798±0.012**	0.774±0.009	0.783±0.007	0.792±0.009	0.786±0.008	0.774±0.006	0.777±0.006	0.784±0.001
NCI-H522	0.778±0.006	0.763±0.010	0.766±0.007	**0.782±0.008**	0.764±0.011	0.761±0.008	0.761±0.008	0.762±0.001
**BACC**								
A549	**0.662±0.025**	0.598±0.044	0.602±0.017	0.630±0.014	0.623±0.025	0.572±0.015	0.574±0.015	0.529±0.000
EKVX	**0.632±0.012**	0.602±0.028	0.597±0.021	0.623±0.020	0.602±0.020	0.601±0.015	0.547±0.010	0.535±0.003
HOP-62	**0.664±0.027**	0.599±0.027	0.630±0.022	0.631±0.017	0.637±0.023	0.611±0.026	0.582±0.010	0.557±0.000
HOP-92	**0.610±0.008**	0.576±0.015	0.585±0.018	0.598±0.011	0.592±0.024	0.568±0.013	0.560±0.010	0.551±0.002
NCI-H226	**0.683±0.019**	0.608±0.020	0.612±0.018	0.666±0.015	0.663±0.021	0.611±0.023	0.570±0.013	0.566±0.001
NCI-H460	**0.635±0.028**	0.566±0.023	0.568±0.016	0.605±0.012	0.600±0.014	0.552±0.021	0.557±0.016	0.541±0.002
NCI-H522	**0.631±0.016**	0.611±0.023	0.597±0.018	0.626±0.011	0.619±0.020	0.574±0.021	0.559±0.012	0.539±0.002

Note: Values shown in bold indicate the best performance when compared to each metric in each model.

S12 Predictive Performance on Classification Task Across Cell Lines of ALMANAC-Ovary Tissue Dataset

Table S12 The predictive performance on classification task across cell lines of ALMANAC-Ovary tissue dataset, with difference training models.

Cell Line	SynProtX-GATFP	SynProtX-GAT	SynProtX-GCN	SynProtX-AttFP	DeepSynergy	DeepDDS	AttenSyn	XGBoost
**AUROC**								
OVCAR-4	**0.795±0.011**	0.769±0.017	0.749±0.022	0.782±0.015	0.772±0.013	0.775±0.013	0.710±0.012	0.649±0.003
OVCAR-5	**0.744±0.013**	0.687±0.015	0.696±0.039	0.730±0.012	0.687±0.018	0.698±0.011	0.660±0.021	0.606±0.004
OVCAR-8	**0.804±0.011**	0.784±0.013	0.784±0.029	0.785±0.011	0.763±0.010	0.780±0.011	0.741±0.016	0.726±0.002
SK-OV-3	**0.779±0.011**	0.739±0.011	0.751±0.023	0.772±0.010	0.760±0.010	0.755±0.009	0.724±0.017	0.717±0.002
**AUCPR**								
OVCAR-4	**0.553±0.017**	0.484±0.024	0.480±0.043	0.535±0.024	0.498±0.012	0.496±0.021	0.420±0.029	0.360±0.001
OVCAR-5	**0.484±0.022**	0.427±0.024	0.423±0.048	0.454±0.018	0.431±0.029	0.441±0.017	0.384±0.027	0.360±0.001
OVCAR-8	**0.585±0.021**	0.554±0.022	0.537±0.051	0.553±0.014	0.534±0.013	0.560±0.012	0.480±0.039	0.487±0.001
SK-OV-3	**0.555±0.021**	0.491±0.021	0.475±0.031	0.518±0.020	0.532±0.014	0.492±0.021	0.453±0.026	0.426±0.001
**ACC**								
OVCAR-4	**0.828±0.006**	0.817±0.008	0.819±0.010	0.827±0.006	0.818±0.006	0.815±0.007	0.813±0.008	0.805±0.000
OVCAR-5	**0.790±0.007**	0.776±0.011	0.777±0.006	0.782±0.005	0.778±0.008	0.778±0.011	0.774±0.006	0.771±0.000
OVCAR-8	**0.844±0.009**	0.836±0.008	0.832±0.011	0.840±0.005	0.837±0.005	0.841±0.005	0.824±0.008	0.833±0.001
SK-OV-3	**0.807±0.009**	0.790±0.009	0.782±0.008	0.792±0.008	0.800±0.006	0.793±0.009	0.784±0.008	0.777±0.000
**BACC**								
OVCAR-4	**0.642±0.013**	0.600±0.029	0.571±0.033	0.620±0.020	0.613±0.022	0.616±0.015	0.568±0.026	0.541±0.000
OVCAR-5	0.586±0.012	0.573±0.016	0.540±0.018	0.566±0.012	0.565±0.022	**0.590±0.016**	0.549±0.017	0.541±0.000
OVCAR-8	0.655±0.013	0.634±0.033	0.585±0.035	0.622±0.015	0.627±0.019	**0.658±0.015**	0.581±0.027	0.593±0.000
SK-OV-3	**0.636±0.017**	0.592±0.026	0.551±0.027	0.588±0.019	0.619±0.020	0.617±0.017	0.566±0.028	0.562±0.000

Note: Values shown in bold indicate the best performance when compared to each metric in each model.

S13 Predictive Performance on Classification Task Across Cell Lines of ALMANAC-Skin Tissue Dataset

Table S13 The predictive performance on classification task across cell lines of ALMANAC-Skin tissue dataset, with difference training models.

Cell Line	SynProtX-GATFP	SynProtX-GAT	SynProtX-GCN	SynProtX-AttFP	DeepSynergy	DeepDDS	AttenSyn	XGBoost
**AUROC**								
SK-MEL-2	**0.817±0.012**	0.782±0.018	0.798±0.012	0.759±0.019	0.802±0.009	0.784±0.018	0.720±0.017	0.715±0.007
SK-MEL-28	**0.771±0.009**	0.747±0.010	0.760±0.010	0.731±0.024	0.723±0.015	0.736±0.015	0.697±0.017	0.625±0.005
SK-MEL-5	0.776±0.008	0.736±0.012	**0.777±0.006**	0.734±0.025	0.732±0.021	0.734±0.009	0.689±0.014	0.661±0.007
UACC-257	0.784±0.007	0.772±0.011	**0.787±0.012**	0.733±0.017	0.767±0.008	0.762±0.013	0.713±0.008	0.664±0.001
**AUCPR**								
SK-MEL-2	**0.566±0.034**	0.507±0.040	0.502±0.027	0.468±0.044	0.561±0.027	0.503±0.041	0.408±0.025	0.452±0.012
SK-MEL-28	**0.433±0.019**	0.388±0.017	0.399±0.014	0.352±0.030	0.368±0.028	0.357±0.027	0.321±0.024	0.291±0.008
SK-MEL-5	**0.404±0.017**	0.368±0.023	0.390±0.017	0.353±0.029	0.350±0.024	0.370±0.015	0.329±0.023	0.297±0.006
UACC-257	**0.529±0.013**	0.505±0.025	0.520±0.020	0.461±0.026	0.513±0.021	0.499±0.024	0.423±0.022	0.379±0.001
**ACC**								
SK-MEL-2	**0.809±0.012**	0.797±0.012	0.797±0.012	0.793±0.013	0.805±0.010	0.792±0.014	0.781±0.008	0.791±0.002
SK-MEL-28	**0.818±0.005**	0.810±0.005	0.815±0.004	0.807±0.005	0.806±0.007	0.799±0.008	0.800±0.012	0.815±0.001
SK-MEL-5	**0.834±0.006**	0.832±0.005	0.829±0.007	0.831±0.006	0.825±0.010	0.829±0.006	0.827±0.006	0.834±0.002
UACC-257	**0.796±0.008**	0.791±0.008	**0.796±0.007**	0.787±0.011	**0.796±0.010**	0.788±0.012	0.779±0.007	0.779±0.002
**BACC**								
SK-MEL-2	0.636±0.025	0.632±0.024	0.611±0.028	0.587±0.027	**0.646±0.033**	0.625±0.023	0.551±0.025	0.523±0.003
SK-MEL-28	**0.578±0.014**	0.558±0.014	0.538±0.013	0.529±0.016	0.559±0.030	0.560±0.021	0.537±0.019	0.520±0.003
SK-MEL-5	**0.591±0.017**	0.562±0.018	0.544±0.015	0.544±0.021	0.568±0.020	0.582±0.023	0.560±0.022	0.517±0.004
UACC-257	**0.642±0.020**	0.624±0.028	0.603±0.021	0.575±0.028	0.633±0.027	0.632±0.024	0.548±0.018	0.523±0.004

Note: Values shown in bold indicate the best performance when compared to each metric in each model.

S14 Predictive Performance on Classification Task Across Cell Lines of FRIEDMAN Study Dataset

Table S14 The predictive performance on classification task across cell lines of FRIEDMAN study dataset, with difference training models.

**Cell Line**	**SynProtX-GATFP**	**SynProtX-GAT**	**SynProtX-GCN**	**SynProtX-AttFP**	**DeepSynergy**	**DeepDDS**	**AttenSyn**	**XGBoost**
**AUROC**								
A2058	**0.774±0.007**	0.757±0.008	0.732±0.015	0.745±0.015	0.762±0.006	0.746±0.014	0.651±0.011	0.734±0.003
G-361	**0.823±0.005**	0.807±0.007	0.801±0.013	0.807±0.017	0.819±0.005	0.806±0.007	0.704±0.014	0.800±0.002
IPC-298	0.828±0.005	0.818±0.008	0.779±0.015	0.799±0.010	**0.832±0.005**	0.794±0.010	0.700±0.010	0.794±0.000
RVH-421	**0.791±0.004**	0.764±0.008	0.753±0.013	0.765±0.012	0.784±0.007	0.756±0.015	0.610±0.008	0.739±0.003
SK-MEL-2	**0.825±0.004**	0.795±0.009	0.787±0.008	0.787±0.015	0.824±0.004	0.790±0.009	0.687±0.008	0.780±0.001
SK-MEL-28	**0.864±0.005**	0.838±0.010	0.835±0.011	0.846±0.010	0.850±0.006	0.822±0.017	0.656±0.008	0.795±0.003
SK-MEL-5	**0.792±0.007**	0.772±0.008	0.744±0.013	0.752±0.013	0.778±0.007	0.733±0.021	0.625±0.015	0.740±0.004
UACC-257	**0.759±0.004**	0.740±0.008	0.717±0.013	0.734±0.017	0.754±0.005	0.740±0.006	0.633±0.010	0.718±0.002
**AUCPR**								
A2058	**0.614±0.007**	0.576±0.014	0.567±0.012	0.583±0.014	0.598±0.009	0.594±0.012	0.487±0.015	0.583±0.004
G-361	**0.756±0.010**	0.725±0.011	0.729±0.018	0.740±0.019	0.754±0.006	0.732±0.011	0.621±0.016	0.741±0.006
IPC-298	0.712±0.008	0.693±0.013	0.644±0.023	0.667±0.014	**0.721±0.007**	0.671±0.017	0.549±0.015	0.678±0.003
RVH-421	**0.656±0.007**	0.593±0.011	0.586±0.023	0.615±0.018	0.645±0.008	0.590±0.024	0.396±0.009	0.561±0.003
SK-MEL-2	**0.739±0.007**	0.691±0.016	0.669±0.014	0.674±0.023	0.727±0.006	0.686±0.014	0.518±0.014	0.641±0.004
SK-MEL-28	**0.780±0.010**	0.760±0.014	0.739±0.020	0.758±0.017	0.772±0.007	0.730±0.022	0.537±0.016	0.688±0.004
SK-MEL-5	**0.701±0.008**	0.657±0.013	0.637±0.019	0.656±0.012	0.686±0.012	0.639±0.022	0.547±0.018	0.657±0.005
UACC-257	**0.703±0.007**	0.690±0.012	0.650±0.016	0.676±0.019	0.690±0.007	0.678±0.009	0.569±0.011	0.657±0.001
**ACC**								
A2058	**0.734±0.005**	0.718±0.007	0.710±0.008	0.715±0.007	0.724±0.007	0.723±0.007	0.684±0.009	0.701±0.002

Cell Line	SynProtX-GATFP	SynProtX-GAT	SynProtX-GCN	SynProtX-AttFP	DeepSynergy	DeepDDS	AttenSyn	XGBoost
G-361	**0.747±0.008**	0.738±0.006	0.726±0.016	0.733±0.014	0.745±0.006	0.729±0.012	0.654±0.006	0.707±0.002
IPC-298	0.760±0.007	0.745±0.012	0.723±0.019	0.741±0.011	**0.763±0.007**	0.736±0.008	0.676±0.008	0.718±0.003
RVH-421	**0.752±0.008**	0.735±0.009	0.727±0.013	0.735±0.014	0.743±0.008	0.719±0.014	0.658±0.009	0.718±0.003
SK-MEL-2	**0.759±0.008**	0.740±0.009	0.728±0.012	0.723±0.015	0.758±0.007	0.737±0.012	0.653±0.011	0.696±0.002
SK-MEL-28	**0.790±0.008**	0.749±0.018	0.763±0.013	0.774±0.013	0.782±0.008	0.746±0.019	0.649±0.011	0.715±0.004
SK-MEL-5	**0.742±0.008**	0.699±0.010	0.704±0.013	0.711±0.008	0.732±0.009	0.704±0.011	0.670±0.010	0.695±0.001
UACC-257	**0.688±0.007**	0.664±0.010	0.659±0.008	0.667±0.014	0.682±0.008	0.672±0.011	0.599±0.011	0.671±0.003
**BACC**								
A2058	**0.664±0.012**	0.620±0.016	0.625±0.019	0.626±0.020	0.649±0.013	0.632±0.019	0.575±0.011	0.567±0.003
G-361	**0.736±0.009**	0.719±0.009	0.718±0.018	0.718±0.018	0.735±0.008	0.719±0.013	0.597±0.008	0.674±0.003
IPC-298	0.724±0.011	0.696±0.019	0.678±0.027	0.694±0.014	**0.728±0.015**	0.684±0.011	0.582±0.013	0.624±0.004
RVH-421	**0.703±0.010**	0.640±0.025	0.647±0.015	0.665±0.015	0.690±0.014	0.659±0.030	0.538±0.011	0.597±0.004
SK-MEL-2	**0.721±0.009**	0.689±0.017	0.681±0.020	0.669±0.025	0.723±0.008	0.691±0.015	0.564±0.017	0.608±0.002
SK-MEL-28	**0.767±0.010**	0.698±0.030	0.742±0.019	0.756±0.011	0.755±0.016	0.709±0.028	0.576±0.011	0.647±0.005
SK-MEL-5	**0.694±0.010**	0.609±0.018	0.642±0.018	0.644±0.015	0.680±0.016	0.630±0.024	0.584±0.009	0.598±0.001
UACC-257	**0.680±0.007**	0.654±0.010	0.650±0.009	0.659±0.016	0.676±0.009	0.667±0.012	0.564±0.012	0.651±0.003

Note: Values shown in bold indicate the best performance when compared to each metric in each model.

S15 Predictive Performance on Classification Task Across Cell Lines of ONEIL Study Dataset

Table S15 The predictive performance on classification task across cell lines of ONEIL study dataset, with difference training models.

Cell Line	SynProtX-GATFP	SynProtX-GAT	SynProtX-GCN	SynProtX-AttFP	DeepSynergy	DeepDDS	AttenSyn	XGBoost
**AUROC**								
A2058	0.873±0.016	**0.889±0.025**	0.815±0.015	0.884±0.029	0.871±0.024	0.811±0.024	0.774±0.024	0.869±0.005
A2780	0.844±0.017	0.868±0.020	0.869±0.006	0.866±0.022	0.857±0.006	**0.872±0.009**	0.867±0.021	0.863±0.003
A427	0.895±0.020	0.877±0.018	0.802±0.010	0.841±0.029	0.895±0.018	0.842±0.019	0.818±0.015	**0.899±0.003**
NCI-H460	**0.819±0.024**	0.777±0.032	0.723±0.010	0.785±0.020	0.793±0.031	0.737±0.036	0.748±0.030	0.813±0.003
RKO	0.737±0.019	0.708±0.020	0.740±0.013	0.729±0.015	0.704±0.020	0.733±0.022	0.715±0.022	**0.752±0.001**
SK-OV-3	0.808±0.022	0.804±0.022	0.770±0.013	0.764±0.026	**0.820±0.015**	0.775±0.012	0.780±0.014	0.805±0.002
SW837	0.801±0.019	0.808±0.035	0.781±0.015	0.762±0.019	0.819±0.016	0.771±0.015	0.772±0.016	**0.823±0.005**
**AUCPR**								
A2058	0.832±0.022	**0.839±0.031**	0.758±0.019	0.835±0.046	0.830±0.034	0.748±0.035	0.707±0.035	0.838±0.006
A2780	0.906±0.016	0.923±0.015	**0.930±0.003**	0.924±0.014	0.913±0.009	0.926±0.015	0.925±0.012	0.907±0.005
A427	0.866±0.026	0.846±0.030	0.739±0.026	0.763±0.050	0.866±0.024	0.795±0.028	0.760±0.030	**0.882±0.004**
NCI-H460	**0.775±0.024**	0.753±0.034	0.697±0.015	0.734±0.025	0.774±0.028	0.698±0.039	0.694±0.034	**0.775±0.005**
RKO	0.532±0.035	0.484±0.020	0.522±0.018	0.522±0.023	0.546±0.021	0.499±0.028	0.476±0.037	**0.567±0.004**
SK-OV-3	0.719±0.052	0.744±0.046	0.741±0.018	0.669±0.048	**0.785±0.024**	0.759±0.007	0.771±0.020	0.766±0.003
SW837	0.656±0.038	0.624±0.046	0.557±0.024	0.554±0.031	**0.681±0.029**	0.567±0.023	0.566±0.022	0.656±0.005
**ACC**								
A2058	0.771±0.031	0.777±0.055	0.710±0.028	**0.787±0.040**	0.765±0.029	0.718±0.028	0.688±0.026	0.757±0.008
A2780	0.757±0.026	0.794±0.022	0.801±0.016	0.785±0.028	0.782±0.021	0.792±0.024	0.699±0.019	**0.808±0.006**
A427	**0.804±0.033**	0.778±0.034	0.709±0.018	0.753±0.041	0.792±0.027	0.749±0.029	0.725±0.023	0.791±0.010

Cell Line	SynProtX-GATFP	SynProtX-GAT	SynProtX-GCN	SynProtX-AttFP	DeepSynergy	DeepDDS	AttenSyn	XGBoost
NCI-H460	0.720±0.029	0.716±0.042	0.662±0.016	0.701±0.017	0.729±0.025	0.680±0.024	0.672±0.027	**0.734±0.006**
RKO	0.696±0.031	0.686±0.025	0.713±0.016	0.694±0.020	0.706±0.037	0.719±0.027	0.671±0.035	**0.747±0.010**
SK-OV-3	0.733±0.025	0.715±0.034	0.684±0.026	0.682±0.027	**0.739±0.026**	0.707±0.019	0.696±0.032	0.731±0.005
SW837	0.735±0.046	0.737±0.039	0.718±0.017	0.693±0.023	0.755±0.018	0.725±0.023	0.726±0.024	**0.769±0.005**
**BACC**								
A2058	0.779±0.030	0.792±0.044	0.715±0.034	**0.790±0.040**	0.768±0.024	0.713±0.030	0.685±0.031	0.775±0.015
A2780	0.719±0.042	**0.767±0.041**	0.770±0.033	0.755±0.035	0.763±0.032	0.750±0.045	0.759±0.017	0.761±0.009
A427	**0.806±0.033**	0.780±0.034	0.702±0.019	0.753±0.041	0.787±0.027	0.742±0.034	0.720±0.023	0.795±0.010
NCI-H460	0.697±0.035	0.692±0.040	0.634±0.017	0.673±0.024	0.703±0.025	0.654±0.024	0.658±0.028	**0.708±0.006**
RKO	0.649±0.033	0.606±0.023	0.650±0.028	0.632±0.021	0.661±0.030	0.645±0.043	0.657±0.030	**0.686±0.014**
SK-OV-3	0.732±0.026	0.714±0.035	0.679±0.030	0.679±0.029	**0.735±0.027**	0.705±0.018	0.698±0.032	0.732±0.006
SW837	0.733±0.035	0.734±0.031	0.706±0.021	0.695±0.032	0.749±0.020	0.708±0.020	0.726±0.026	**0.757±0.004**

Note: Values shown in bold indicate the best performance when compared to each metric in each model.

S16 Relevant Cancer-Associated Proteins of Cancer Drug Combinations from Gradient-Based Method

Table S16 Relevant cancer-associated proteins as derived from gradient-based method of SynProtX-GATFP, and potential cancer pathways by KEGG (enrichment analysis) of drug combinations.

Drug A	Drug B	Cell line	Top Cancer-Associated Protein from SynProtX-GATFP (Gradient-based method)	Potential Cancer Pathways by KEGG (Enrichment Analysis)
**ALMANAC-Breast**				
Vemurafenib	Raloxifene	MDA-MB-468	UBP16 FBLI1 TRM1L DDX31 PMM2 LPCT4 BAP18 NPA1P IKIP MRCKB KIF23 MOC2B ECHP MYEF2 OFUT1 JUN VPS52 AIDA SMC6 LDLR ARFP2 ENOG TIGAR SPAG7 TLE3 HMOX2 ZMY11 AASS JUND 8ODP MIER1 PLK1 GLYR1 NPAT SMTN SNF8 KPCA HOOK3 PRI1 DDX55 SC24B RL36L PAPOA ZNT1 PHRF1 CD123 GAPR1 LRP1 CSTFT C19L1	• Breast cancer<br>• Pathways in cancer<br>• Kaposi sarcoma<br>• MicroRNAs in cancer<br>• Cell cycle<br>• Human T-cell leukemia
**ALMANAC-Lung**				
Gefitinib	Mitoxantrone	NCI-H460	COG4 SMAG2 PP4C M3K20 AVEN ARFG3 CHMP5 PDRG1 ZN706 FKB15 AKTS1 MELPH GCR GSK3A MTFR1 DHRS1 PMF1 STALP JAK1 PKHA6 CNOT9 NS1BP VCIP1 EMAL3 FBLI1 ARBK1 ASTRA CP2S1 UBE2C VPS16 PAAF1 CFDP1 SETD3 SAAL1 PP2AB N6MT1 CSN7B DUS2L CO6A3 EI2BA EMAL2 KANK2 YTHD3 SPAS2 I2BP2 PSF2 CPLX2 HMCS1 HABP4 STK39	• Kaposi sarcoma<br>• Cell cycle<br>• Human T-cell leukemia<br>• PI3K-Akt signalling pathway<br>• Pancreatic cancer<br>• EGFR tyrosine kinase inhibitor resistance

S17 Top-ranked 50 Proteins by Integrated Gradients Method Across SynProtX-GATFP on the ALMANAC-Breast dataset for Vismodegib–Mitotane

Table S17 Top-ranked 50 proteins by integrated gradients method across SynProtX-GATFP of Vismodegib–Mitotane via MDA-MB-468 and MCF-7 cell lines on the ALMANAC-Breast dataset

Rank	Vismodegib–Mitotane (ALMANAC-Breast dataset)			
	MDA-MB-468		MCF-7	
	Protein	Value	Protein	Value
1	K1C17	0.0119120	H15	0.0053896
2	S10A8	0.0109378	GCSH	0.0051622
3	K2C5	0.0096419	ABCB6	0.0043620
4	CO1A2	0.0095085	LTOR5	0.0037625
5	NPT2B	0.0093752	K1C19	0.0037487
6	CASPE	0.0093715	KCRU	0.0036341
7	MGP	0.0093616	DREB	0.0034044
8	K1C16	0.0086739	RABP2	0.0033599
9	NEP	0.0085985	HSPB1	0.0031749
10	K2C7	0.0078972	PEPL	0.0030939
11	ZA2G	0.0078911	EPIPL	0.0030874
12	S10A9	0.0078694	SCG2	0.0027980
13	CE290	0.0077631	K2C73	0.0027439
14	S100P	0.0077570	DECR2	0.0027131
15	K2C4	0.0076877	MDHM	0.0027104
16	K1C23	0.0075942	CPT1A	0.0026259
17	S10A4	0.0075327	RB27B	0.0025848
18	RET1	0.0072928	F16P1	0.0025021
19	TACC2	0.0070346	GBG5	0.0024982
20	NGAL	0.0069978	NQO1	0.0024891
21	TRI29	0.0069687	UBP8	0.0024601
22	AGR2	0.0068253	PX11B	0.0024530
23	CP4X1	0.0067547	MEPCE	0.0024515
24	S35F6	0.0067291	ACPM	0.0024429
25	PCAT2	0.0067260	CADH1	0.0024248
26	PDZ1I	0.0066630	ISCU	0.0024225
27	SUSD2	0.0066283	CLUS	0.0024113
28	PADI2	0.0065693	SERA	0.0023975

Rank	Vismodegib–Mitotane (ALMANAC-Breast dataset)			
	**MDA-MB-468**		**MCF-7**	
	**Protein**	**Value**	**Protein**	**Value**
29	K2C6B	0.0064665	CING	0.0023697
30	CRBG1	0.0064557	LYRM4	0.0023654
31	GTPB8	0.0063506	NIPS1	0.0023539
32	H11	0.0063106	K1C15	0.0023418
33	CD14	0.0062871	DESP	0.0023404
34	ITB6	0.0062607	FPRP	0.0023292
35	RDH10	0.0061130	FUND2	0.0022939
36	BST2	0.0060431	RM52	0.0022875
37	AK1C2	0.0060327	TCPW	0.0022848
38	K1C13	0.0060074	SPTN2	0.0022708
39	CP27A	0.0059603	OCLN	0.0022525
40	CTL2	0.0059425	TSTD1	0.0022235
41	CALL5	0.0058364	CERS2	0.0022010
42	TPMT	0.0058052	BAK	0.0021878
43	TIG1	0.0057589	COA4	0.0021768
44	S10A7	0.0057302	RM21	0.0021610
45	STING	0.0057196	NECT1	0.0021573
46	ALAT2	0.0057018	COQ7	0.0021530
47	AACT	0.0056976	MMAB	0.0021399
48	K1C19	0.0056715	RPAB2	0.0021054
49	POSTN	0.0056620	LTOR2	0.0020720
50	LANC2	0.0056417	QCR2	0.0020510

S18 Top-ranked 50 Proteins by Integrated Gradients Method Across SynProtX-GATFP on the ALMANAC-Lung dataset for Vandetanib–Gefitinib

Table S18 Top-ranked 50 proteins by integrated gradients method across SynProtX-GATFP of Vandetanib–Gefitinib via NCI-H226 and A549 cell lines on the ALMANAC-Lung dataset

Rank	Vandetanib–Gefitinib (ALMANAC-Lung dataset)			
	NCI-H226		A549	
	Protein	Value	Protein	Value
1	CD63	0.0089934	AL3A1	0.0098799
2	LICH	0.0083760	AK1BA	0.0089786
3	KRT85	0.0073314	CPLX2	0.0059581
4	PXDC2	0.0073013	POSTN	0.0059466
5	GPNMB	0.0072258	AT2A3	0.0059057
6	NNMT	0.0070880	CE290	0.0057492
7	OGDHL	0.0070761	AK1D1	0.0056440
8	TSN3	0.0063796	AL1A1	0.0054685
9	CO1A1	0.0063565	CEAM6	0.0054327
10	IFIT3	0.0058148	AK1C3	0.0051395
11	MATN2	0.0056599	MUC5A	0.0049320
12	UPP1	0.0054183	CNTN1	0.0048365
13	CLN5	0.0053160	AK1C2	0.0048002
14	GDN	0.0052872	NR2C2	0.0045954
15	HOGA1	0.0052655	RBPMS	0.0044510
16	GABT	0.0052632	EST1	0.0044043
17	COCA1	0.0051626	GSH1	0.0042583
18	PLOD2	0.0051436	UGDH	0.0041464
19	AGRG6	0.0050949	1433S	0.0040982
20	UAP1	0.0050772	IRF3	0.0040133
21	GTPB8	0.0050471	GRB7	0.0039205
22	K2C7	0.0050070	ANX13	0.0039145
23	IFIT1	0.0049425	PP14A	0.0039137
24	RDH10	0.0049330	KYNU	0.0038757
25	ARL 1.00	0.0049099	CP24A	0.0038577
26	IL6RB	0.0048995	PMM2	0.0038500
27	RAB32	0.0048699	CBR3	0.0038374
28	HKDC1	0.0047895	TRI18	0.0038189

Rank	Vandetanib–Gefitinib (ALMANAC-Lung dataset)			
	**NCI-H226**		**A549**	
	**Protein**	**Value**	**Protein**	**Value**
29	DAAM1	0.0047765	PGDH	0.0038119
30	APOL2	0.0047717	PURA1	0.0038010
31	CASP6	0.0047571	R4RL2	0.0037489
32	RAI3	0.0047196	AAGAB	0.0037454
33	CALB2	0.0046617	CRIP1	0.0037289
34	TAGL	0.0046556	CEAM5	0.0037084
35	AASS	0.0046400	GALM	0.0036804
36	SYNE1	0.0046352	PTER	0.0036772
37	DDR2	0.0045986	CLIP2	0.0035841
38	DDB2	0.0045869	HERC2	0.0035629
39	NMES1	0.0045523	LXN	0.0035346
40	CADH2	0.0045358	ASURF	0.0035288
41	DPP4	0.0044612	AGR2	0.0035038
42	STAT2	0.0043902	GCSP	0.0035008
43	K2C8	0.0043878	DYN1	0.0034465
44	NIBA1	0.0043587	PDE3A	0.0033620
45	TMM 40	0.0043341	CRIPT	0.0033351
46	DGLB	0.0043133	CAH12	0.0033193
47	DDX60	0.0042690	S10A4	0.0033089
48	TAXB1	0.0042347	CAD17	0.0032577
49	GXLT1	0.0042262	KCD15	0.0032470
50	CBPA4	0.0042092	SEN34	0.0031988

S19 Hyperparameter Settings of SynProtX

Table S19 Details of hyperparameter settings with Bayesian optimization of SynProtX on each experimental setting for molecular structure of drugs: SynProtX-GATFP, SynProtX-GAT, SynProtX-GCN, and SynProtX-AttFP, and deep neural network for cancer gene expression and protein level.

Hyperparameter	Values
**SynProtX-GATFP**	
nheads	int [2, 8, step=1]
dropout_attention_rate	float [0.1, 0.9, step=0.1]
dropout_fpdnn_rate	float [0.1, 0.9, step=0.1]
output_units_num	int [25, 300, step=25]
**SynProtX-GAT**	
nheads	int [2, 8, step=1]
dropout_attention_rate	float [0.1, 0.9, step=0.1]
output_units_num	int [25, 300, step=25]
**SynProtX-GCN**	
dropout_convolution_rate	float [0.1, 0.9, step=0.1]
predictor_dropout	float [0.1, 0.9, step=0.1]
**SynProtX-AttFP**	
num_layers_attentive	int [1, 3, step=1]
num_timesteps	int [1, 5, step=1]
dropout_attentive_rate	float [0.1, 0.9, step=0.1]
fingerprint_dim	int [100, 300, step=25]
output_units_num	int [25, 300, step=25]
**Deep neural network**	
num_layers_predictor	int [2, 4, step=1]
MLPu1	1024, 2048, 4096
MLPu2	512, 1024
output_units_num	int [25, 300, step=25]
dropout_gene	float [0.1, 0.9, step=0.1]
dropout_predictor_rate	float [0.1, 0.9, step=0.1]
learning_rate	log [1e-2 – 1e-5]
weight_decay	log [1e-2 – 1e-5]

S20 Best Hyperparameter Settings of SynProtX-GATFP on Regression Task

Table S20 Details of best hyperparameter settings of SynProtX-GATFP on regression task across the Tissue Datasets: (1) ALMANAC-Breast, (2) ALMANAC-Lung, (3) ALMANAC-Ovary, and (4) ALMANAC-Skin; and the Study Datasets: (1) FRIEDMAN and (2) ONEIL.

Dataset	Hyperparameter	Best Value
**Tissue Datasets**		
ALMANAC-Breast	*nheads*	4
	dropout_attention_rate	0.44389613679790946
	dropout_fpdnn_rate	0.7564280434307596
	num_layers_predictor	3
	MLPu1	2048
	MLPu2	512
	output_units_num	50
	dropout_gene	0.37538971837545676
	dropout_predictor_rate	0.18854688564729968
	learning_rate	0.0004391945098190547
	weight_decay	1.0810932255300814e-05
ALMANAC-Lung	*nheads*	6
	dropout_attention_rate	0.3625547108965045
	dropout_fpdnn_rate	0.7277896458090426
	num_layers_predictor	3
	MLPu1	2048
	MLPu2	512
	output_units_num	250
	dropout_gene	0.4222230594224384
	dropout_predictor_rate	0.565977284215205
	learning_rate	0.0009729254149406361
	weight_decay	0.003537385949149189
ALMANAC-Ovary	*nheads*	5
	dropout_attention_rate	0.47770853084795734
	dropout_fpdnn_rate	0.7516502946102612
	num_layers_predictor	4
	MLPu1	2048
	MLPu2	512

Dataset	Hyperparameter	Best Value
	output_units_num	100
	dropout_gene	0.19260335942889573
	dropout_predictor_rate	0.7897227936187262
	learning_rate	0.00029679642628554993
	weight_decay	9.84410693251873e-05
ALMANAC-Skin	*nheads*	3
	dropout_attention_rate	0.46728939622201393
	dropout_fpdnn_rate	0.32412724844642415
	num_layers_predictor	2
	MLPu1	2048
	MLPu2	1024
	output_units_num	300
	dropout_gene	0.796536447927593
	dropout_predictor_rate	0.2652688296612887
	learning_rate	0.0008857326766369115
	weight_decay	0.00014533956108185242
**Study Datasets**		
FRIEDMAN (Skin)	*nheads*	8
	dropout_attention_rate	0.4294472590871302
	dropout_fpdnn_rate	0.6944856830721977
	num_layers_predictor	2
	MLPu1	1024
	MLPu2	512
	output_units_num	25
	dropout_gene	0.7112791765007452
	dropout_predictor_rate	0.13099268876913517
	learning_rate	0.00027826175893976956
	weight_decay	0.008114318351787454
ONEIL (Several Tissues)	*nheads*	3
	dropout_attention_rate	0.35576866415252906
	dropout_fpdnn_rate	0.7486659628105069
	num_layers_predictor	3
	MLPu1	4096
	MLPu2	512
	output_units_num	125

Dataset	Hyperparameter	Best Value
	dropout_gene	0.10852487585241928
	dropout_predictor_rate	0.6952111184454656
	learning_rate	0.0011091188863786184
	weight_decay	1.6260280213997754e-05

S21 Best Hyperparameter Settings of SynProtX-GATFP on Classification Task

Table S21 Details of best hyperparameter settings of SynProtX-GATFP on classification task across the Tissue Datasets: (1) ALMANAC-Breast, (2) ALMANAC-Lung, (3) ALMANAC-Ovary, and (4) ALMANAC-Skin; and the Study Datasets: (1) FRIEDMAN and (2) ONEIL.

**Dataset**	**Hyperparameter**	**Best Value**
**Tissue Datasets**		
ALMANAC-Breast	*nheads*	6
	dropout_attention_rate	0.3149202140836747
	dropout_fpdnn_rate	0.6312080891089443
	num_layers_predictor	2
	MLPu1	2048
	MLPu2	1024
	output_units_num	75
	dropout_gene	0.898339299916983
	dropout_predictor_rate	0.7018169381239597
	learning_rate	6.851538285575944e-05
	weight_decay	0.009790196756679997
ALMANAC-Lung	*nheads*	8
	dropout_attention_rate	0.2679680564399717
	dropout_fpdnn_rate	0.5967702399768787
	num_layers_predictor	2
	MLPu1	2048
	MLPu2	512
	output_units_num	100
	dropout_gene	0.7745204028481096
	dropout_predictor_rate	0.5457235157553214
	learning_rate	0.0004802034222196436
	weight_decay	9.518052360982848e-05
ALMANAC-Ovary	*nheads*	3
	dropout_attention_rate	0.2329596756262481
	dropout_fpdnn_rate	0.6066193828750519
	num_layers_predictor	2
	MLPu1	2048
	MLPu2	1024

Dataset	Hyperparameter	Best Value
	output_units_num	175
	dropout_gene	0.26661704130219266
	dropout_predictor_rate	0.6802607513001638
	learning_rate	0.0003511693373589024
	weight_decay	0.000935166117882642
ALMANAC-Skin	*nheads*	7
	dropout_attention_rate	0.4300872835788689
	dropout_fpdnn_rate	0.2046398398762181
	num_layers_predictor	2
	MLPu1	4096
	MLPu2	1024
	output_units_num	75
	dropout_gene	0.3936474332799164
	dropout_predictor_rate	0.7962702724031074
	learning_rate	5.3643004326442655e-05
	weight_decay	0.00023645043824771573
**Study Datasets**		
FRIEDMAN (Skin)	*nheads*	8
	dropout_attention_rate	0.4470489953903367
	dropout_fpdnn_rate	0.5750105068763585
	num_layers_predictor	3
	MLPu1	4096
	MLPu2	1024
	output_units_num	100
	dropout_gene	0.3889424243176259
	dropout_predictor_rate	0.634516894324329
	learning_rate	1.3265069570738596e-05
	weight_decay	7.759430731828581e-05
ONEIL (Several Tissues)	*nheads*	3
	dropout_attention_rate	0.7403539399507482
	dropout_fpdnn_rate	0.8996684873290127
	num_layers_predictor	3
	MLPu1	4096
	MLPu2	512
	output_units_num	175

Dataset	Hyperparameter	Best Value
	dropout_gene	0.24199915030282948
	dropout_predictor_rate	0.7921501991896348
	learning_rate	0.0007260425442666259
	weight_decay	3.448923261276046e-05

REFERENCES

[1] T. N. Kipf and M. Welling, "Semi-supervised classification with graph convolutional networks," *arXiv preprint arXiv:1609.02907,* 2016.

[2] P. Veličković, G. Cucurull, A. Casanova, A. Romero, P. Lio, and Y. Bengio, "Graph attention networks," *arXiv preprint arXiv:1710.10903,* 2017.

[3] Z. Xiong *et al.*, "Pushing the boundaries of molecular representation for drug discovery with the graph attention mechanism," *Journal of medicinal chemistry,* vol. 63, no. 16, pp. 8749-8760, 2019.
