# Supplementary material for: SynProtX: a large-scale proteomics-based deep learning model for predicting synergistic anticancer drug combinations
Source: Gigascience. 2025 Aug 12;14:giaf080. doi: 10.1093/gigascience/giaf080 (PMC12343095; doi:10.1093/gigascience/giaf080)
Supplement: giaf080_GIGA-D-24-00586_Original_Submission [file giaf080_giga-d-24-00586_original_submission.pdf]

GigaScience

SynProtX: A Large-Scale Proteomics-Based Deep Learning Model for Predicting Synergistic Anticancer Drug Combinations

--Manuscript Draft--

**Manuscript Number:**	GIGA-D-24-00586
**Full Title:**	SynProtX: A Large-Scale Proteomics-Based Deep Learning Model for Predicting Synergistic Anticancer Drug Combinations
**Article Type:**	Research
**Funding Information:**	Vidyasirimedhi Institute of Science and Technology — Mr. Bundit Boonyarit
**Abstract:**	Motivation: Drug combination therapy plays a pivotal role in addressing the molecular heterogeneity of cancer, improving treatment efficacy, minimizing resistance, and reducing toxicity. Deep learning approaches have significantly advanced drug combination discovery by addressing the limitations of conventional laboratory experiments, which are time consuming and costly. While most existing models rely on the molecular structure of drugs and gene expression data, incorporating protein-level expression provides a more accurate representation of cellular behavior and drug responses. In this study, we introduce SynProtX, an enhanced deep learning model that integrates large-scale protein and gene expression data with deep neural networks (DNNs) and the molecular structure of drugs with graph neural networks (GNNs).<br><br>Results: The SynProtX-GATFP model, which combines molecular graphs and fingerprints using a graph attention network architecture, demonstrated superior predictive performance for the FRIEDMAN-Skin dataset. Furthermore, SynProtX outperformed existing state-of-the-art models across diverse cancer tissue datasets from the ALMANAC study, including breast, lung, ovary, and skin tissues. By incorporating protein expression data, the model consistently achieved higher accuracy than gene expression-only models, reflecting the functional state of cancer cells. The generalizability of SynProtX was rigorously validated using leave-drug-combination-out cross-validation, which confirmed its reliability across different tissue types and datasets. Additionally, SynProtX identified key cancer-associated proteins and molecular substructures, offering novel insights into the biological mechanisms underlying drug synergy. These findings highlight the potential of integrating large-scale proteomics and multi-omics data to advance anticancer drug design and combination therapy strategies for personalized medicine.<br><br>Availability and implementation: https://github.com/manbaritone/SynProtX and https://doi.org/10.5281/zenodo.14040426.
**Corresponding Author:**	Bundit Boonyarit<br>Vidyasirimedhi Institute of Science and Technology<br>Wangchan, Rayong THAILAND
**Corresponding Author Secondary Information:**	
**Corresponding Author's Institution:**	Vidyasirimedhi Institute of Science and Technology
**Corresponding Author's Secondary Institution:**	
**First Author:**	Bundit Boonyarit
**First Author Secondary Information:**	
**Order of Authors:**	Bundit Boonyarit<br>Matin Kositchutima<br>Tisorn Naphattalung<br>Nattawin Yamprasert

Powered by Editorial Manager® and ProduXion Manager® from Aries Systems Corporation

	Chanitra Thuwajit
	Thanyada Rungrotmongkol
	Sarana Nutanong
**Order of Authors Secondary Information:**	
**Additional Information:**	
**Question**	**Response**
Are you submitting this manuscript to a special series or article collection?	No
**Experimental design and statistics**<br><br>Full details of the experimental design and statistical methods used should be given in the Methods section, as detailed in our Minimum Standards Reporting Checklist. Information essential to interpreting the data presented should be made available in the figure legends.<br><br>Have you included all the information requested in your manuscript?	Yes
**Resources**<br><br>A description of all resources used, including antibodies, cell lines, animals and software tools, with enough information to allow them to be uniquely identified, should be included in the Methods section. Authors are strongly encouraged to cite Research Resource Identifiers (RRIDs) for antibodies, model organisms and tools, where possible.<br><br>Have you included the information requested as detailed in our Minimum Standards Reporting Checklist?	Yes
**Availability of data and materials**<br><br>All datasets and code on which the conclusions of the paper rely must be either included in your submission or deposited in publicly available repositories (where available and ethically	Yes

appropriate), referencing such data using a unique identifier in the references and in the “Availability of Data and Materials” section of your manuscript.<br><br>Have you have met the above requirement as detailed in our Minimum Standards Reporting Checklist?	
GigaScience has policies and guidelines in place for the use of generative AI-writing tools such as ChatGPT. If you have used such writing tools to assist with writing the manuscript this must be declared and cited in the text. Authors should not list AI-writing tools and other AI-assisted technologies as an author or co-author and should acknowledge that they are fully responsible for text generated or refined by AI-writing tools.<p><br><br>A summary of use (particularly in the introduction or among methods) needs to be included at the end of the paper, and the outputs should also be included as a supplementary file hosted in GigaDB or other open repositories. Please read our guidelines for more information. <p><br><br>By submitting to GigaScience, you are aware of the journal's AI-writing tools policy, and if you have declared use of such tools below, you have acknowledged this where appropriate in your manuscript and have made a summary of use and outputs available. <p><br><b>AI-assisted writing tools have been used in the preparation of this manuscript?	Yes

Manuscript
Click here to access/download;Manuscript;SynProtX_main_V6.docx

SynProtX: A Large-Scale Proteomics-Based Deep Learning Model for Predicting Synergistic Anticancer Drug Combinations

Bundit Boonyarit[1], Matin Kositchutima[2], Tisorn Naphattalung[2], Nattawin Yamprasert[3], Chanitra Thuwajit[4], Thanyada Rungrotmongkol[5,6,*], Sarana Nutanong[1,*]

[1]School of Information Science and Technology, Vidyasirimedhi Institute of Science and Technology, Rayong, 21210, Thailand

[2]Kamnoetvidya Science Academy, Rayong, 21210, Thailand

[3]School of Information, Computer, and Communication Technology, Sirindhorn International Institute of Technology, Thammasat University, Pathum Thani, 12120, Thailand

[4]Department of Immunology, Faculty of Medicine Siriraj Hospital, Mahidol University, Bangkok, 10700, Thailand

[5]Program in Bioinformatics and Computational Biology, Graduate School, Chulalongkorn University, Bangkok, 10330, Thailand

[6]Center of Excellence in Structural and Computational Biology, Department of Biochemistry, Faculty of Science, Chulalongkorn University, Bangkok, 10330, Thailand

*** Corresponding authors**

(1) Sarana Nutanong – *School of Information Science and Technology, Vidyasirimedhi Institute of Science and Technology, Rayong, 21210, Thailand; Email: snutanon@vistec.ac.th*

(2) Thanyada Rungrotmongkol – *Program in Bioinformatics and Computational Biology, Graduate School, Chulalongkorn University, Bangkok, 10330, Thailand; Center of Excellence in*

Structural and Computational Biology, Department of Biochemistry, Faculty of Science, Chulalongkorn University, Bangkok, 10330, Thailand; Email: thanyada.r@chula.ac.th

GRAPHICAL ABSTRACT

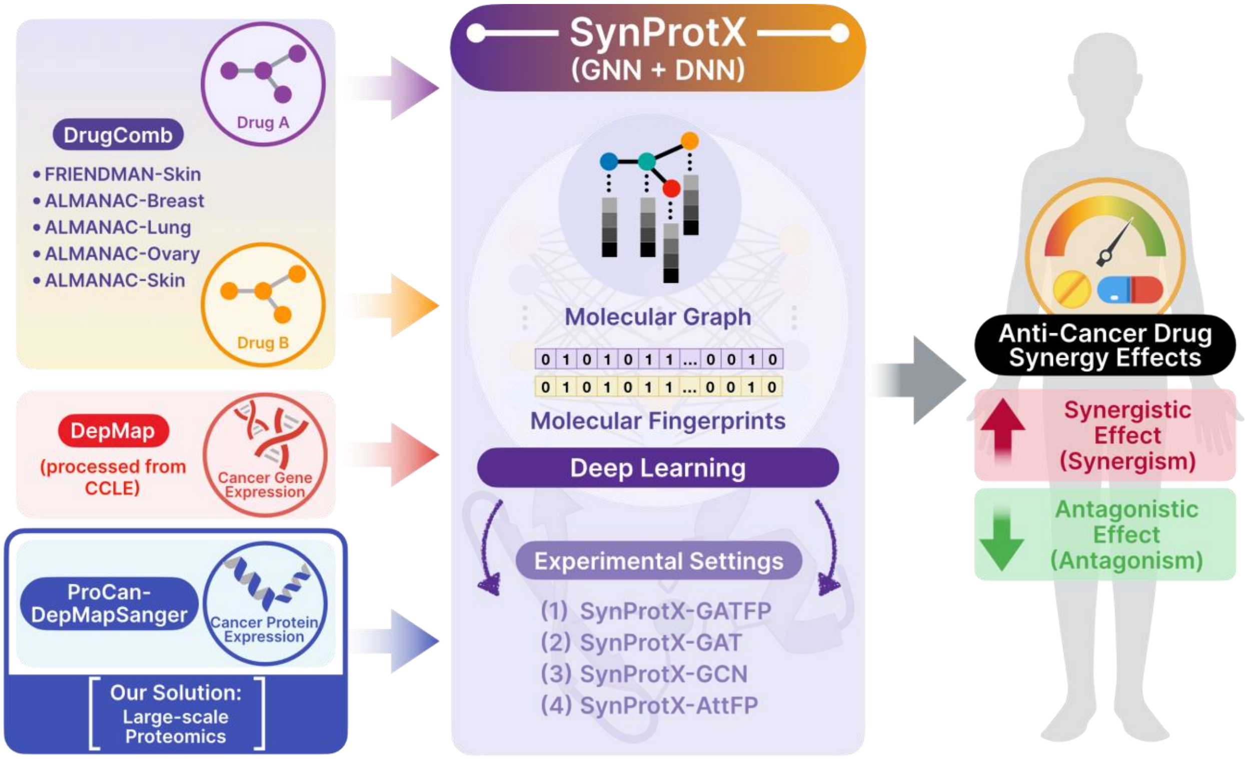

SynProtX, a deep learning model leveraging large-scale proteomics, molecular graphs, and fingerprints to enhance the prediction of the synergistic effects of anticancer drug combinations

ABSTRACT

Motivation: Drug combination therapy plays a pivotal role in addressing the molecular heterogeneity of cancer, improving treatment efficacy, minimizing resistance, and reducing toxicity. Deep learning approaches have significantly advanced drug combination discovery by addressing the limitations of conventional laboratory experiments, which are time consuming and costly. While most existing models rely on the molecular structure of drugs and gene expression data, incorporating protein-level expression provides a more accurate representation of cellular behavior and drug responses. In this study, we introduce SynProtX, an enhanced deep learning model that integrates large-scale protein and gene expression data with deep neural networks (DNNs) and the molecular structure of drugs with graph neural networks (GNNs).

Results: The SynProtX-GATFP model, which combines molecular graphs and fingerprints using a graph attention network architecture, demonstrated superior predictive performance for the FRIEDMAN-Skin dataset. Furthermore, SynProtX outperformed existing state-of-the-art models across diverse cancer tissue datasets from the ALMANAC study, including breast, lung, ovary, and skin tissues. By incorporating protein expression data, the model consistently achieved higher accuracy than gene expression-only models, reflecting the functional state of cancer cells. The generalizability of SynProtX was rigorously validated using leave-drug-combination-out cross-validation, which confirmed its reliability across different tissue types and datasets. Additionally, SynProtX identified key cancer-associated proteins and molecular substructures, offering novel insights into the biological mechanisms underlying drug synergy. These findings highlight the potential of integrating large-scale proteomics and multi-omics data to advance anticancer drug design and combination therapy strategies for personalized medicine.

Availability and implementation: https://github.com/manbaritone/SynProtX and https://doi.org/10.5281/zenodo.14040426.

***Keywords*:** cancer drug combination, deep learning, drug discovery, graph neural networks, machine learning, multi-omics, personalized medicine, proteomics, synergistic effect

BACKGROUND

Cancer treatment faces significant challenges, including tumor heterogeneity, which leads to resistance to monotherapy because of the diverse genetic and molecular profiles within tumors. This resistance arises because cancer cells can adapt and survive via various mechanisms [1, 2]. Chemotherapy and targeted therapies, while pivotal in treatment, are limited by severe side effects and rapid resistance development [3-5]. Single-agent therapies often fail to address multiple pathways involved in cancer progression [6]. Combination therapies, which target multiple pathways, offer improved efficacy, reduced resistance, and minimized adverse effects, making them essential for optimizing patient outcomes [7]. Despite the benefits of combination therapies, prediction of effective drug combinations remains challenging. Traditional methods are labor-intensive, time-consuming, costly, and often require extensive *in vitro* and *in vivo* testing [8, 9]. Moreover, the complex interactions between drugs and their molecular targets complicate the prediction of their synergistic effects.

Machine learning (ML) and deep learning (DL) approaches provide promising solutions for predicting effective drug combinations by analyzing large datasets and identifying complex patterns of multi-omics data, including genomics and transcriptomics [10]. DL algorithms, in particular, excel at learning nonlinear relationships from biological data, enhance drug discovery efficiency, and reduce experimental costs [11]. These models rely on multi-omics data, including genomics, transcriptomics, metabolomics, and proteomics, which are crucial for understanding cancer behavior and treatment response [12]. Important cancer cell multi-omics databases include the Cancer Cell Line Encyclopedia (CCLE), which compiles information on copy number variations, gene expression, gene mutations, and DNA methylation [13, 14]. However, the availability of multi-omics data is limited, often necessitating reliance on genomic and transcriptomic data for drug response studies [15].

Several ML and DL models have been developed to predict anticancer drug synergy. Celebi et al. (2019) compared multiple ML algorithms, including extreme gradient boosting (XGBoost), random forest (RF), support vector machine (SVM), Lasso, and linear regression, to classify drug combination efficacy [16]. DeepSynergy, a deep neural network model, incorporates the molecular structure of drugs and gene expression data to classify and predict synergy scores for anticancer

drug combinations [11]. The model processes the drug structure and gene expression data separately for each drug. DeepDDS utilizes graph convolutional networks and graph attention networks to learn the molecular structure of drugs combined with multilayer perceptrons for gene expression data [17]. AttenSyn further improves predictions by employing attention mechanisms to identify the relevant molecular structure of drugs and gene expression features [18].

Protein expression profiling at the protein level plays a critical role in cancer biology by capturing functional cellular states that are not reflected at the genomic or transcriptomic levels. Large-scale datasets, such as ProCan-DepMapSanger, provide protein-level expression data for 949 cancer cell lines, covering 8,498 proteins across over 40 cancer types, enabling the identification of biomarkers and therapeutic targets [19]. Advances in high-throughput proteomics, particularly mass spectrometry, allow for comprehensive protein-level analyses, which reveal phenotypic characteristics and offer a more comprehensive understanding of cancer biology and drug responses [20]. Protein expression data are crucial for understanding drug responses, as proteins can interact with drugs and various biomolecules, reflecting the disease’s pathways in living organisms [21, 22]. Differential protein expression can pinpoint potential drug targets, unravel mechanisms of drug resistance, and identify protein signatures predictive of therapeutic responses, supporting personalized treatment strategies [23].

Building on the significance of protein expression profiling, we developed SynProtX, a DL model designed to predict the effects of drug combinations by integrating gene and protein expression data with deep neural networks (DNNs) and molecular structure of drugs with graph neural networks (GNNs). SynProtX utilizes data from the DrugComb database [24, 25], CCLE [13, 14], and ProCan-DepMapSanger [19] to predict the synergistic effects of drug combinations. We employed graph convolutional networks (GCN) [26], graph attention networks (GAT) [27], Attentive FP [28], and graph attention networks combined with molecular fingerprint (GATFP) to compare the learning processes of the molecular structure of drugs with gene and protein expression data. Our findings demonstrate that SynProtX-GATFP, which combines molecular graphs and fingerprints through a GAT coupled with DNN, provides robust predictive performance in the FRIEDMAN-Skin study [29] and surpasses state-of-the-art and baseline models on the ALMANAC dataset across the breast, lung, ovary, and skin tissues [30]. The generalizability of SynProtX is evidenced by the leave-drug-combination-out validation method. Additionally,

SynProtX identifies cancer-associated proteins and molecular substructures of drugs, offering insights into the biological mechanisms underlying drug synergy. By leveraging proteomics, SynProtX improves the accuracy of synergy predictions and contributes to advancing anticancer drug discovery for personalized medicine.

Our contributions are summarized as follows:

- We introduced SynProtX, a DL model for predicting anticancer drug synergy utilizing new large-scale proteomics data, which aids in discovering effective anticancer drug combinations.
- We developed SynProtX using deep neural networks for gene and protein expression by integrating comprehensive molecular data from the CCLE and ProCan-DepMapSanger databases and graph neural networks for the molecular structure of drugs, thereby enhancing the accuracy of synergy predictions.
- We designed SynProtX to visualize the importance of each molecular substructure in synergistic effects by incorporating attention mechanisms within the model architecture, allowing researchers to better understand and interpret the model's predictions.
- We validated the performance of SynProtX using the leave-one-drug-combination-out validation method and demonstrated its robustness and generalizability across diverse cancer types.

PROBLEM FORMULATION

The SynProtX includes both classification and regression models. The classification model aims to classify the synergism of drug combinations, whereas the regression model aims to predict the synergy score of drug combinations. The models use the drug combination data, gene expression data, and protein expression data to calculate the predicted synergy score y_c for each drug combination. The predicted score, $y_c(A, B, g, p) \in \mathbb{R}$, depends on the drug combination $< A, B >$, the gene expression level g, and the protein expression level p.

For the calculation of the synergistic score S_{Loewe}, it is obtained from drug A with concentration x_1, which has an efficacy value of y_1, and drug B with concentration x_2, which has an efficacy value of y_2. For the classification task, Loewe scores [31] that are positive ($S_{Loewe} > 0$) indicated that the drug pair is considered synergistic, whereas for negative ($S_{Loewe} < 0$) scores, the drug pair is considered antagonistic. Loewe scores can be calculated using the following formula [32]:

$$S_{Loewe} = y_c - y_1(x_1 + x_2) = y_c - y_2(x_1 + x_2) \quad (1)$$

The dataset includes drug pairs i and j that have been modified to form molecular graphs M_A and M_B, respectively. The gene expression level g and protein expression level p are transformed into the vectors v_g and v_p, respectively. The drug combination (M_A and M_B), vector of gene expression (v_g), and vector of protein expression (v_p) data are used for synergy prediction.

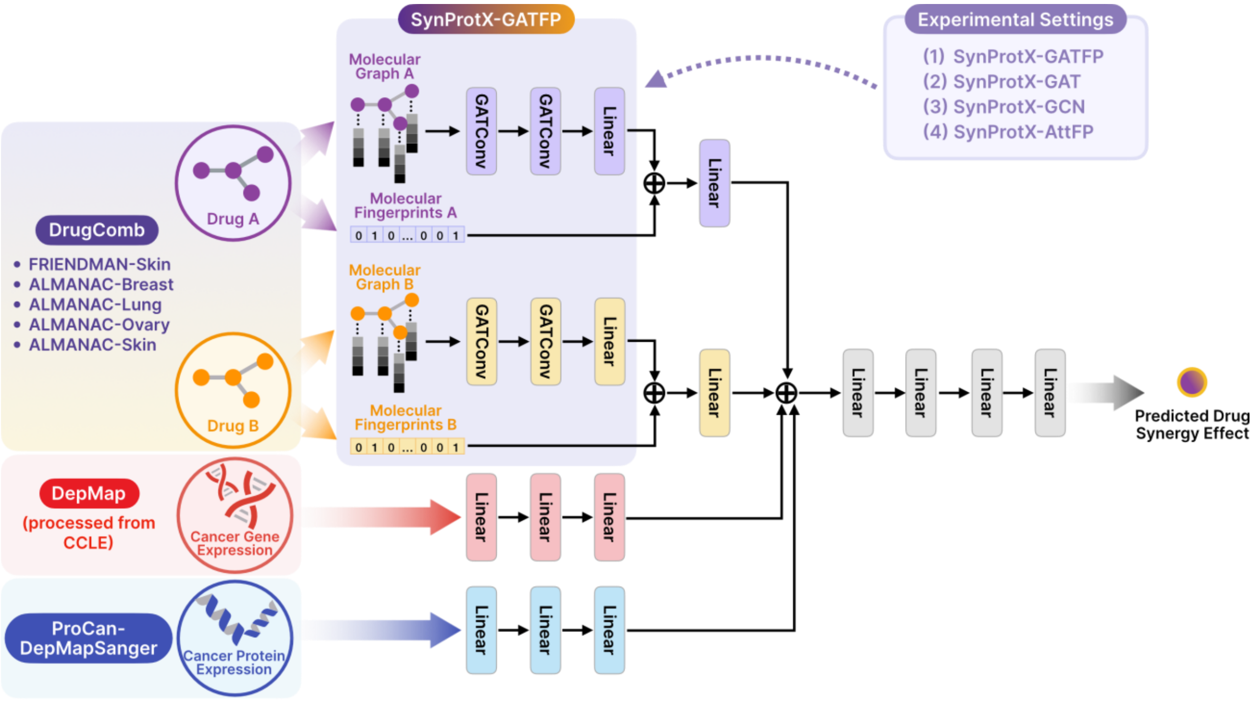

Figure 1. Overview of the SynProtX model architecture with the SynProtX-GATFP experimental setting for predicting the synergistic effects of anticancer drug combinations. The model leverages data from DrugComb, including the FRIEDMAN-Skin, ALMANAC-Breast, ALMANAC-Lung, ALMANAC-Ovary, and ALMANAC-Skin datasets, and incorporates gene and protein expressions from DepMap

(processed from CCLE) and ProCan-DepMapSanger, respectively. The model achieves deep feature extraction based on different experimental settings (SynProtX-GATFP, SynProtX-GAT, SynProtX-GCN, and SynProtX-AttFP) to enhance representation learning for the molecular structure of drug combinations.

MODEL ARCHITECTURE

For the architecture of the developed predictive model for enhancing the efficacy of combination drug therapy in cancer treatment, as illustrated in **Figure 1**, each drug combination consists of molecular graphs that enter the first layer along with gene expression and protein expression data for both drugs (Drug A and Drug B). Gene and protein expression data from cancer cells were learned through deep neural network algorithms, and the resulting embedding vectors of the drug combination, gene expression, and protein expression were concatenated to create a feature vector before being fed into the deep neural network algorithm.

The SynProtX-GATFP model integrates multiple data types, including molecular graphs, molecular fingerprints, gene expression, and protein expression data, to predict drug synergy effects. The following mathematical formulation outlines the model architecture and its components.

Input Representation

1. **Molecular Graphs**: Each drug i is represented by its molecular graph $M_i = (V_i, E_i)$, where V_i denotes the set of nodes (atoms) and E_i denotes the set of edges (bonds).

2. **Molecular Fingerprints**: Each drug i is represented by a binary vector of molecular fingerprints $F_i \in \{0, 1\}^d$, where d is the dimensionality of the fingerprint vector.

3. **Gene Expression**: The gene expression level for cancer cells is represented $by\ G_{exp} \in \mathbb{R}^n$, where n is the number of genes.

4. **Protein Expression**: The protein expression level for cancer cells is represented by $P_{exp} \in \mathbb{R}^m$, where m is the number of proteins.

Graph Neural Network (GNN)

For each drug i, the molecular graph M_i is processed using GAT to generate node embeddings. Let H_i^0 be the initial node feature matrix (generated atomic features).

$$H_i^{(l+1)} = GATLayer\left(H_i^{(l)}, E_i\right) \tag{2}$$

where H_i^l represents the node features at the l-th layer and $GATLayer$ applies the graph attention mechanism to update the node embeddings. The whole architecture of the $GATLayer$ along with other used architectures is shown in the Molecular Graph and Deep Neural Networks subsection of Methods.

The final node embeddings are then pooled to produce a graph-level embedding:

$$z_i = GlobalPooling(H_i^L) \tag{3}$$

Linear Transformations

The molecular fingerprint F_i, gene expression G_{exp}, and protein expression P_{exp} data are transformed through linear layers as follows:

$$f_i = W_f F_i + b_f \tag{4}$$

$$g_{exp} = W_g G_{exp} + b_g \tag{5}$$

$$p_{exp} = W_p P_{exp} + b_p \tag{6}$$

where W_f , W_g, and W_p are weight matrices and b_f, b_g, and b_p are biased terms.

Features Fusion

For each drug combination (A, B), the embeddings from the GNN and linear transformations are concatenated and processed through additional linear layers to integrate the information:

$$h_A = Concat(z_A, f_A) \tag{7}$$

$$h_B = Concat(z_B, f_B) \tag{8}$$

Interaction Modeling

The interaction between the two drugs is modeled by combining their embeddings:

$$h_{AB} = Concat(h_A, h_B, g_{exp}, p_{exp}) \tag{9}$$

This combined representation is passed through a series of fully connected layers to predict the drug synergy effect.

$$y = \sigma\left(W_3 \left(\sigma\left(W_2 \left(\sigma(W_1 h_{AB} + b_1)\right) + b_2\right)\right) + b_3\right) \tag{10}$$

$$p = sigmoid\left(W_3 \left(\sigma\left(W_2 \left(\sigma(W_1 h_{AB} + b_1)\right) + b_2\right)\right) + b_3\right) \tag{11}$$

where W_1, W_2, and W_3 are weight matrices, b_1, b_2, and b_3 are bias terms, and σ denotes a non-linear activation function (e.g., ReLU).

Output

The final output is y, the scalar value representing the predicted synergy score of the drug combination (A, B), or p, and the predicted probability of the drug combination (A, B) being synergistic.

RESULTS AND DISCUSSION

Protein and Gene Expressions Assisted SynProtX Development

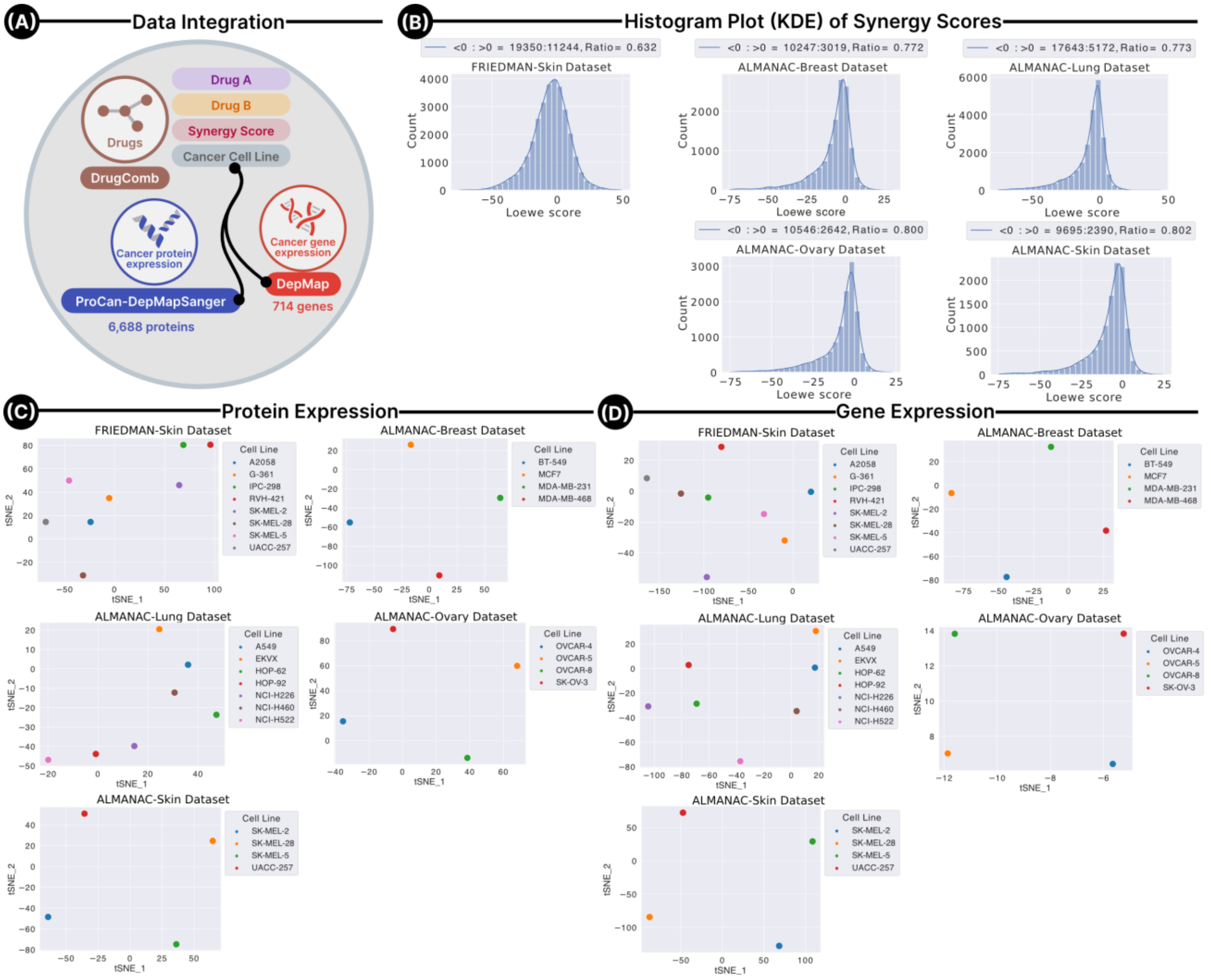

Figure 2. The data integration process and dataset analysis. **(A)** The process begins with the DrugComb database, where each drug combination includes Drug A, Drug B, a Synergy Score (Loewe Score), and a Cancer Cell Line. These combinations are integrated with 6,688 protein expression levels from ProCan-DepMapSanger and 714 gene expression levels from the DepMap database (processed from the CCLE database). **(B)** The distribution of the synergy score (Loewe score) for the FRIEDMAN-Skin, ALMANAC-Breast, ALMANAC-Lung, ALMANAC-Ovary, and ALMANAC-Skin datasets after data preprocessing. **(C)** The t-distributed stochastic neighbor embedding (t-SNE) visualization of cell line embeddings based on protein expression across the FRIEDMAN-Skin, ALMANAC-Breast, ALMANAC-Lung, ALMANAC-

Ovary, and ALMANAC-Skin datasets. **(D)** The t-SNE visualization of cell line embeddings based on gene expression across the same datasets.

The integration of protein and gene expression data is crucial for enhancing the predictive capabilities of SynProtX. The process began by sourcing drug combination data from the DrugComb database, including datasets such as FRIEDMAN-Skin, ALMANAC-Breast, ALMANAC-Lung, ALMANAC-Ovary, and ALMANAC-Skin (**Figure 2A**). Each drug combination included specific drugs (Drug A and Drug B), synergy scores (Loewe scores), and corresponding cancer cell lines. To further enrich this data, protein expression data from the ProCan-DepMapSanger database and gene expression data from the DepMap database, processed from the Cancer Cell Line Encyclopedia (CCLE), were integrated. After filtering and ensuring consistency, 91,944 drug combination experiments were identified, as detailed in **Supplementary Table 1**. This comprehensive dataset provided a robust foundation for model development, enabling extensive training and testing. It is important to note that not every drug pair was tested across all cancer cell types, highlighting the variability and specificity of the data. Integrating multimodal data, including protein and gene expression data, enabled SynProtX to capture the complex molecular signatures associated with drug responses across different cancer cell lines. The synergy scores from DrugComb served as target variables, indicating the efficacy of the drug combinations. Protein expression data provide insights into the proteomic landscape, while gene expression data offered a view of transcriptomic variations across cancer cell lines [33-36]. Together, these datasets allowed the model to understand the functional state and regulatory mechanisms within cells, which are crucial for accurate drug synergy predictions.

The distribution of synergy scores (Loewe scores) across the FRIEDMAN-Skin, ALMANAC-Breast, ALMANAC-Lung, ALMANAC-Ovary, and ALMANAC-Skin datasets was analyzed after data preprocessing (**Figure 2B**). The varying distributions and skewness of these Loewe scores across different datasets provide initial insights into how drug combinations interact within various cancer cell lines. This variability underscores the complexity of predicting drug synergy and highlights the importance of using advanced models to capture these effects accurately. To further explore the underlying structure of the integrated data, t-distributed stochastic neighbor embedding (t-SNE) [37] visualizations were employed. The t-SNE analysis of cell line embeddings based on

protein level (**Figure 2C**) and gene expression (**Figure 2D**) revealed distinct clustering patterns, reflecting the unique molecular characteristics of each cancer cell line. These clusters suggest that the model can effectively distinguish between different cancer cell lines and tissue types, which is essential for generalizing across various biological settings and making accurate drug synergy predictions. Separating clusters in the t-SNE plots indicates that the model can leverage these data to identify unique expression profiles that correlate with synergistic drug effects. The integration of protein and gene expression data allows the SynProtX model to account for the cellular environment and response to external stimuli and the transcriptional landscape within the cancer cells. By incorporating these data types, SynProtX can be used to predict how different drug combinations interact within specific cellular contexts, offering a more comprehensive and accurate tool for identifying synergistic drug combinations. This multi-omics approach enhances the ability of model to capture the intricate interplay between proteomic and transcriptomic factors that influence drug response [38, 39], ultimately leading to more reliable predictions and insights into the mechanisms of drug synergy.

SynProtX Elevated Model's Predictive Performance on Both Classification and Regression Tasks

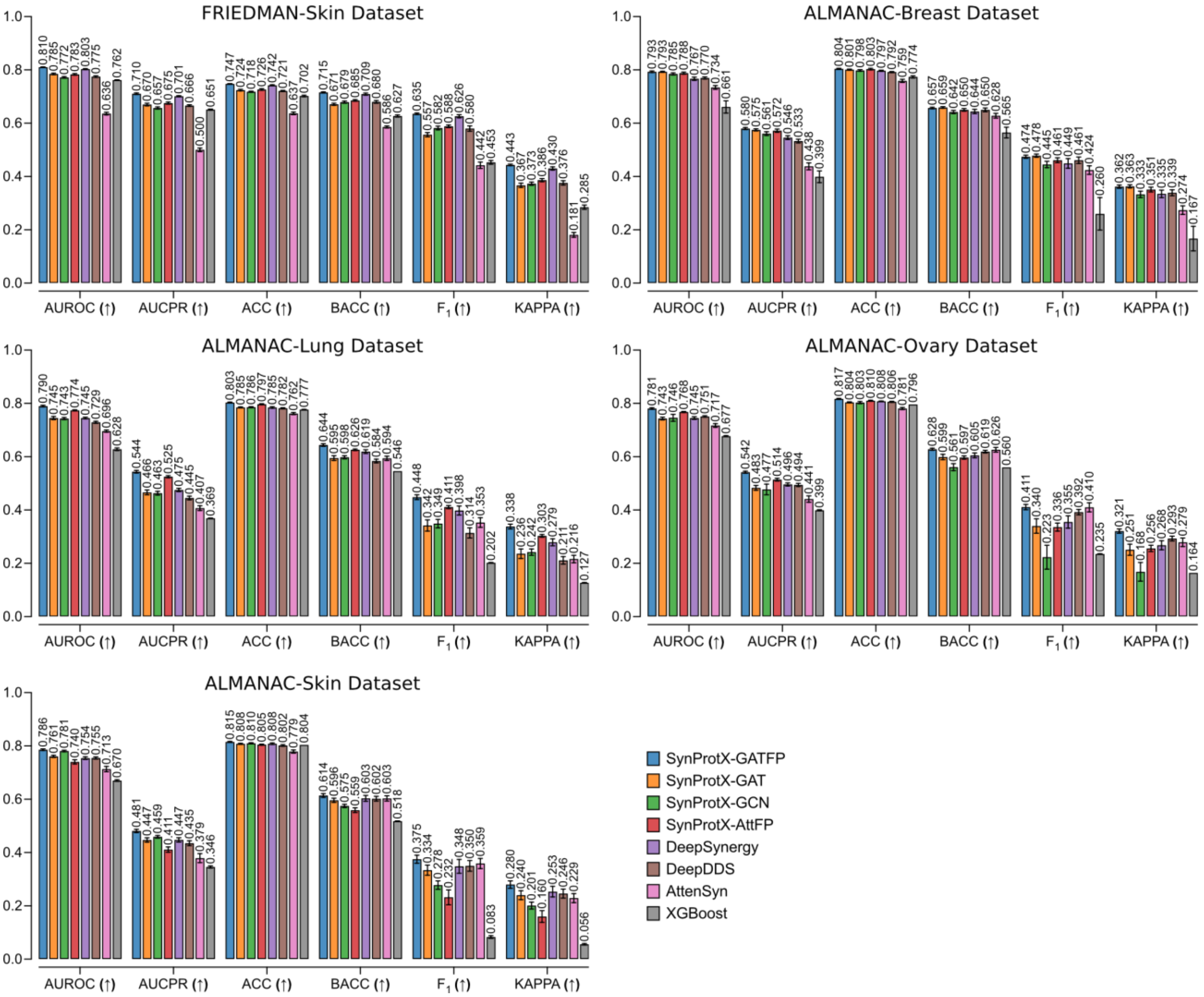

Figure 3. The predictive performance on classification tasks across FRIEDMAN-Skin, ALMANAC-Breast, ALMANAC-Lung, ALMANAC-Ovary, and ALMANAC-Skin datasets with different training models.

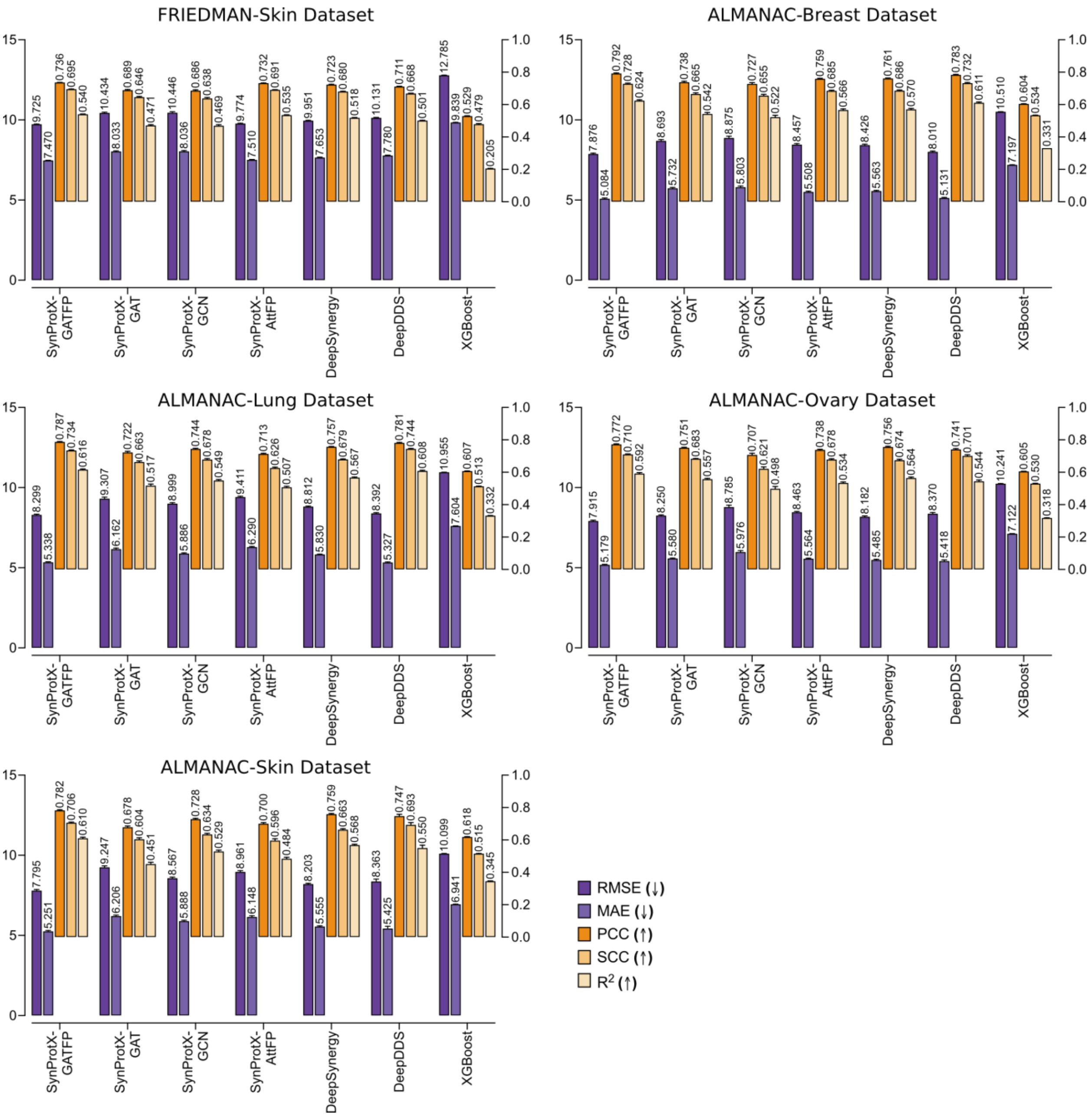

Figure 4. The predictive performance on regression tasks across FRIEDMAN-Skin, ALMANAC-Breast, ALMANAC-Lung, ALMANAC-Ovary, and ALMANAC-Skin datasets with different training models.

The SynProtX model demonstrated improvements in predictive performance across both classification and regression tasks when applied to multiple datasets, including FRIEDMAN-Skin, ALMANAC-Breast, ALMANAC-Lung, ALMANAC-Ovary, and ALMANAC-Skin. The performance of SynProtX for the classification tasks was notably superior across the FRIEDMAN-Skin, ALMANAC-Lung, ALMANAC-Ovary, and ALMANAC-Skin datasets. Specifically, SynProtX-GATFP achieved higher values in critical metrics such as the area under the receiver operating characteristic curve (AUROC), area under the precision-recall curve (AUCPR), accuracy (ACC), balanced accuracy (BACC), F_1 score, and Cohen's Kappa (KAPPA) (**Figure 3 and Supplementary Table S2 and Figures S1 and S2**). These improvements are crucial, indicating the ability of SynProtX to effectively capture the subtle nuances in the data that are pivotal for precise classification. The accuracy of drug synergy predictions can significantly impact downstream drug development processes [40, 41]. SynProtX-GATFP also demonstrated significant enhancements in regression tasks, where it consistently achieved lower Root Mean Square Error (RMSE) and Mean Absolute Error (MAE) values while obtaining higher Pearson Correlation Coefficient (PCC), Spearman's rank Correlation Coefficient (SCC), and R-squared (R^2) values across all datasets. These enhancements included relative performance increases of up to 4.97% and 2.30% for the RMSE and PCC metrics, respectively when compared to the baseline model (**Figure 4 and Supplementary Table S3 and Figure S3**). These results illustrate the robustness of SynProtX in handling regression tasks, especially in complex biological datasets where the accurate prediction of synergy scores is crucial for identifying effective drug combinations.

The PCA analysis provided further insights into predictive performance of SynProtX by visualizing the feature space distribution of drug combinations for each cell line before and after training (**Figure 5**). The PCA plots for the classification task showed that the SynProtX-GATFP model effectively separated different classes, demonstrating its ability to accurately capture the underlying structure of the data. This effective separation suggests that SynProtX can identify and utilize the most relevant features to distinguish between synergistic and non-synergistic drug combinations (**Figure 5A**). For the regression task, PCA visualizations highlighted capacity of SynProtX to closely align the predicted synergy scores with the ground truth. The plots show improved feature space alignment after training, indicating that the model successfully learned the complex relationships between the input features and synergy scores (**Figure 5B**). This alignment

is crucial to contribute reliable predictions across diverse biological settings and underscores capability of SynProtX to generalize effectively across different datasets. In summary, SynProtX significantly improves the predictive performance of classification and regression tasks across various datasets, consistently outperforming other state-of-the-art models. These improvements are particularly evident in the enhanced accuracy and robustness of predictions supported by PCA analysis, which highlights the ability of the model to effectively capture and utilize complex biological data.

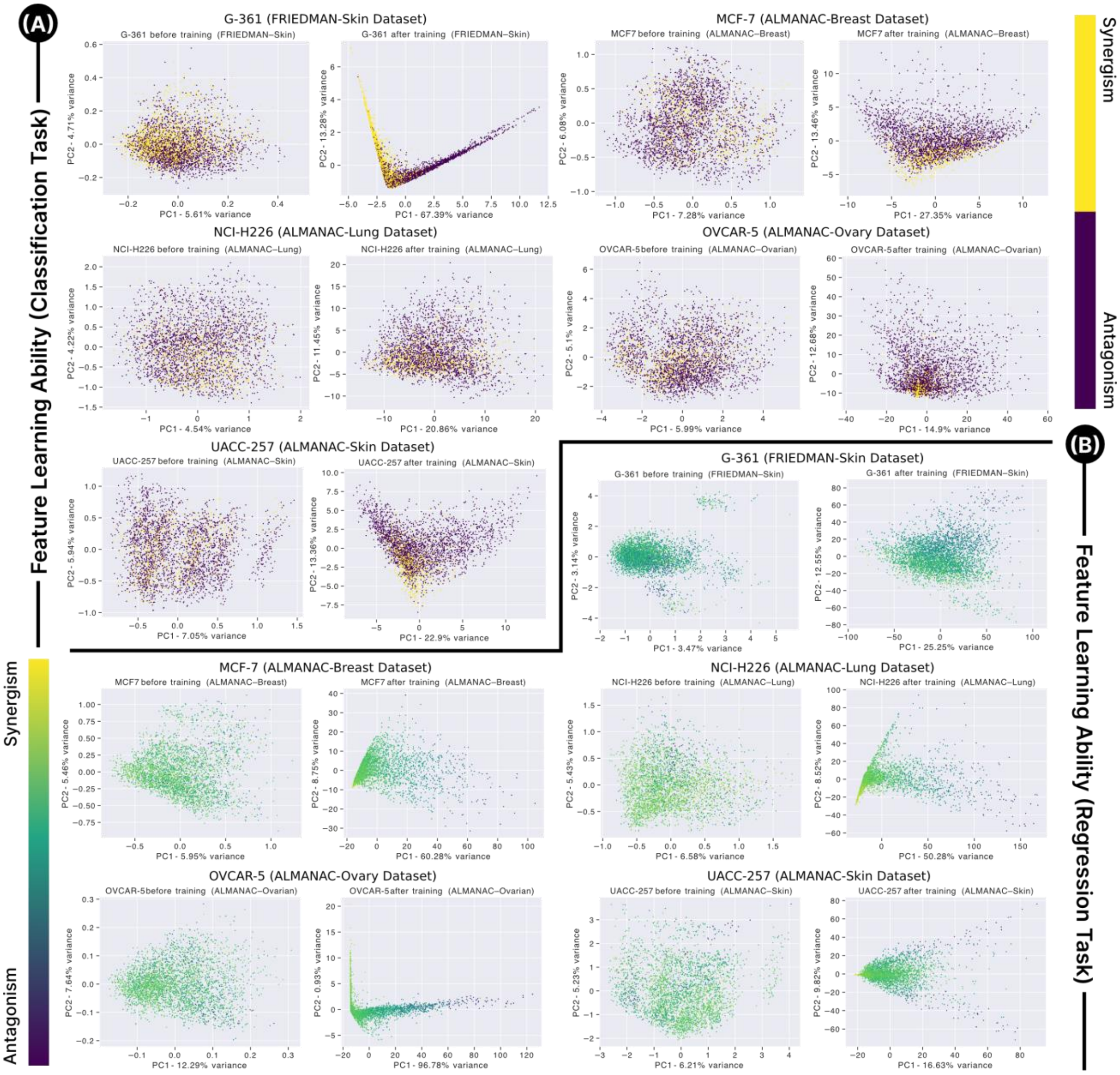

Figure 5. Principal Component Analysis (PCA) visualization of the feature space distribution of drug combinations on each cell line for the SynProtX-GATFP model in **(A)** the classification task and **(B)** the regression task, shown before and after the training process.

Molecular Fingerprints Enhanced Feature Representation and Generalization

The incorporation of molecular fingerprints into the SynProtX model significantly enhances its feature representation and generalization capabilities, thereby improving the predictive performance across various datasets. Integrating molecular fingerprints into SynProtX-GATFP resulted in marked improvements in the predictive performance over SynProtX-GAT across all datasets. In the classification tasks, SynProtX-GATFP consistently outperformed SynProtX-GAT, achieving higher AUROC, AUCPR, accuracy, balanced accuracy, F_1 score, and Cohen's Kappa values. For example, in the FRIEDMAN-Skin dataset, SynProtX-GATFP showed a 2.50% improvement in AUROC and a 2.30% enhancement in accuracy compared to SynProtX-GAT (**Figure 3 and Supplementary Table S2**). Similar trends were observed across the ALMANAC-Breast, ALMANAC-Lung, ALMANAC-Ovary, and ALMANAC-Skin datasets, in which SynProtX-GATFP exhibited improvements across various metrics. In regression tasks, SynProtX-GATFP also demonstrated superior performance, achieving lower RMSE and MAE values while obtaining higher PCC, SCC, and R^2 scores than SynProtX-GAT. These improvements were statistically significant, with PCC increasing by up to 10.4% and R^2 increasing by up to 15.9% (**Figure 4 and Supplementary Table S3**). These results underscore the significant impact of molecular fingerprints on the ability of the model to accurately predict drug synergy effects, reflecting the added value of this feature in capturing the chemical characteristics of drug combinations. Moreover, these improvements highlight the model's enhanced ability to predict synergy scores with greater accuracy and reliability, thereby validating the effectiveness of molecular fingerprints in improving the regression task performance.

The comparison of the distribution of the ground truth and predicted synergy scores further supports the enhanced feature representation and generalization achieved by SynProtX-GATFP. The violin plots in **Supplementary Figure S4** illustrate how SynProtX-GATFP's predictions align more closely with the ground truth distributions across all datasets than SynProtX-GAT. In the ALMANAC-Breast dataset, for example, SynProtX-GATFP displayed narrower and more centered distributions around the ground truth, indicating higher consistency and reduced prediction variance. This alignment is crucial to ensure that the model generalizes well across different datasets and accurately captures the underlying biological mechanisms that drive drug synergy. These results suggest that the molecular fingerprints allowed the model to better capture

the underlying relationships between the chemical structures of drug and their synergistic effects, leading to more precise predictions across different cancer types. The consistent performance improvements across both tasks highlight the importance of molecular fingerprints in enhancing the feature representation within the SynProtX-GATFP model, thereby improving its ability to generalize across various datasets. This enhanced generalization is critical for drug discovery, in which accurate predictions across different biological contexts and chemical spaces are essential. Overall, the inclusion of molecular fingerprints significantly strengthened the capabilities of SynProtX-GATFP, making it a more robust and reliable tool for predicting drug synergy in diverse cancer datasets.

Protein Level Improved Predictive Performance for SynProtX-GATFP

Table 1 The predictive performance on classification task of ablation studies between protein level and gene expression across FRIEDMAN-Skin, ALMANAC-Breast, ALMANAC-Lung, ALMANAC-Ovary, and ALMANAC-Skin datasets with SynProtX-GATFP.

	**Ablation**	**Classification Metrics**					
**Dataset**	**Study**	**AUROC (↑)**	**AUCPR (↑)**	**ACC (↑)**	**BACC (↑)**	**F1 (↑)**	**KAPPA (↑)**
FRIEDMAN-Skin	Protein Level	**0.810±0.001**	**0.710±0.002**	**0.745±0.002**	**0.712±0.003**	**0.629±0.006**	**0.437±0.006**
	Gene Expression	0.776±0.007	0.672±0.009	0.725±0.004	0.675±0.006	0.565±0.010	0.373±0.012
ALMANAC-Breast	Protein Level	**0.791±0.003**	**0.573±0.004**	**0.801±0.002**	**0.658±0.005**	**0.476±0.010**	**0.361±0.009**
	Gene Expression	0.779±0.003	0.525±0.007	0.791±0.001	0.614±0.003	0.385±0.007	0.279±0.006
ALMANAC-Lung	Protein Level	**0.794±0.002**	**0.555±0.004**	**0.805±0.002**	**0.650±0.004**	**0.460±0.008**	**0.351±0.007**
	Gene Expression	0.768±0.004	0.509±0.008	0.792±0.002	0.631±0.009	0.422±0.019	0.306±0.016
ALMANAC-Ovary	Protein Level	**0.779±0.003**	**0.536±0.007**	**0.816±0.002**	**0.624±0.005**	**0.400±0.012**	**0.311±0.011**
	Gene Expression	0.777±0.002	0.529±0.003	0.814±0.001	0.623±0.006	0.398±0.014	0.307±0.012

Dataset	Ablation Study	Classification Metrics					
		AUROC (↑)	AUCPR (↑)	ACC (↑)	BACC (↑)	F1 (↑)	KAPPA (↑)
ALMANAC-Skin	Protein Level	**0.787±0.003**	**0.498±0.006**	**0.816±0.002**	**0.613±0.005**	**0.374±0.011**	**0.282±0.010**
	Gene Expression	0.764±0.004	0.438±0.007	0.805±0.002	0.579±0.008	0.292±0.023	0.204±0.017

Note: Values shown in bold indicate the best performance when compared to each ablation study in each dataset.

Table 2 The predictive performance on regression task of ablation studies between protein level and gene expression across FRIEDMAN-Skin, ALMANAC-Breast, ALMANAC-Lung, ALMANAC-Ovary, and ALMANAC-Skin datasets with SynProtX-GATFP.

Dataset	Model	Regression Metrics				
		RMSE (↓)	MAE (↓)	PCC (↑)	SCC (↑)	R^2 (↑)
FRIEDMAN-Skin	Protein Level	**9.717±0.020**	**7.438±0.016**	**0.737±0.001**	**0.695±0.002**	**0.541±0.002**
	Gene Expression	10.239±0.082	7.888±0.065	0.701±0.006	0.656±0.006	0.490±0.008
ALMANAC-Breast	Protein Level	**7.960±0.083**	**5.140±0.067**	**0.786±0.005**	**0.720±0.006**	**0.616±0.008**
	Gene Expression	8.064±0.068	5.182±0.035	0.781±0.004	0.719±0.003	0.606±0.007
ALMANAC-Lung	Protein Level	**8.428±0.055**	**5.444±0.032**	**0.781±0.003**	**0.726±0.005**	**0.604±0.005**
	Gene Expression	8.735±0.076	5.624±0.045	0.761±0.005	0.709±0.004	0.575±0.007
ALMANAC-Ovary	Protein Level	**7.820±0.082**	**5.096±0.059**	**0.778±0.005**	**0.719±0.006**	**0.602±0.008**
	Gene Expression	8.023±0.042	5.203±0.026	0.766±0.003	0.709±0.003	0.581±0.004
ALMANAC-Skin	Protein Level	**7.740±0.072**	**5.236±0.038**	**0.785±0.005**	**0.707±0.004**	**0.615±0.007**
	Gene Expression	8.095±0.100	5.333±0.083	0.763±0.007	0.694±0.008	0.579±0.010

Note: Values shown in bold indicate the best performance when compared to each ablation study in each dataset.

We conducted ablation studies to determine the specific contributions of protein-level and gene expression data to the predictive performance of the SynProtX-GATFP model. By isolating and comparing the effects of each type of data, we aimed to understand which molecular features most significantly enhance the model's ability to accurately classify and predict drug synergy across various cancer datasets. In the classification task, SynProtX-GATFP incorporating protein-level data outperformed the version that used only gene expression data across the FRIEDMAN-Skin datasets (**Table 1**). Similar improvements were observed across the ALMANAC-Breast, ALMANAC-Lung, ALMANAC-Ovary, and ALMANAC-Skin datasets, with protein-level data consistently leading to better performance across key classification metrics such as balanced accuracy, F_1 score, and Cohen's Kappa. For instance, in the ALMANAC-Lung dataset, protein-level data improved the AUROC by 2.60% compared to gene expression data. The regression task analysis also demonstrated that the SynProtX-GATFP model with protein-level data yielded better results than the gene expression data alone. In the FRIEDMAN-Skin dataset, for example, the model with protein-level data achieved the RMSE of 9.717, which is a 5.09% reduction compared to the RMSE of 10.239 with gene expression data (**Table 2**).

Furthermore, the protein-level data improved the Pearson Correlation Coefficient (PCC) by 3.60% and R^2 by 4.92%, indicating a more accurate prediction of synergy scores. Across other datasets such as ALMANAC-Breast, ALMANAC-Lung, ALMANAC-Ovary, and ALMANAC-Skin, the model similarly showed better performance with protein-level data, with improvements in RMSE, MAE, SCC, and R^2 metrics. These results suggest that protein-level data provide a richer and more detailed molecular context than gene expression data, enabling SynProtX-GATFP to capture complex biological interactions and improve its predictive accuracy. Protein level reflects the functional state of cells more directly than gene expression [42, 43], which may explain why it leads to better model performance in classification and regression tasks. The consistent performance improvements across different datasets underscore the importance of integrating protein-level data in predictive models, such as SynProtX-GATFP. This integration not only enhances the model's ability to generalize across diverse biological contexts but also ensures more reliable and accurate predictions, which are crucial for drug discovery and optimization of combination therapies in cancer treatment.

SynProtX-GATFP Demonstrated Generalizability on Leave-Drug-Combinations-Out Validation

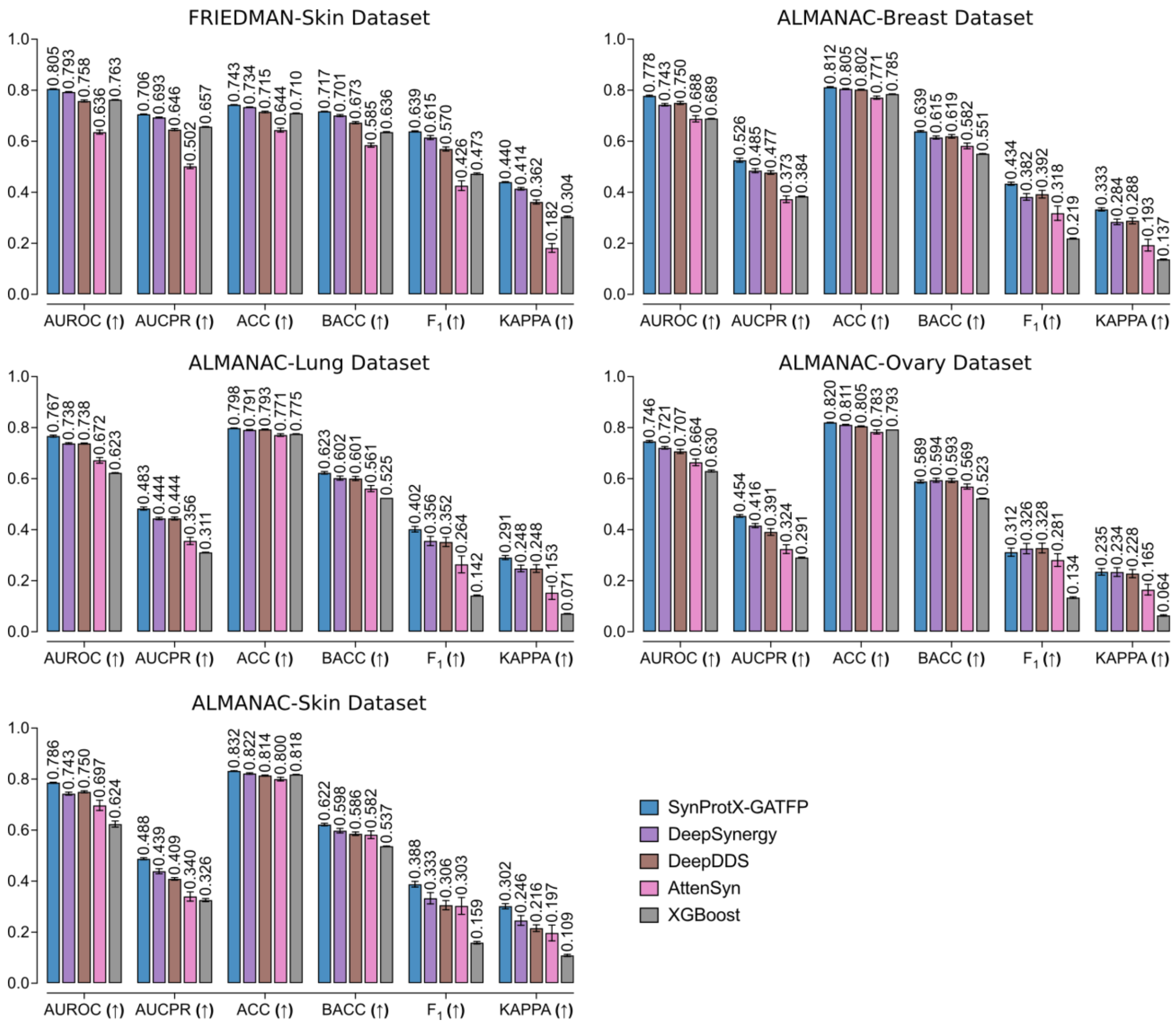

Figure 6. Model performance comparison on the classification task using the leave-drug-combinations-out cross-validation technique for the FRIEDMAN-Skin, ALMANAC-Breast, ALMANAC-Lung, ALMANAC-Ovary, and ALMANAC-Skin datasets.

We experimented with leave-drug-combinations-out validation to rigorously assess the generalizability of the SynProtX-GATFP model in predicting unseen drug combinations. This validation mimics real-world scenarios, in which models must predict the efficacy of novel drug combinations that have not been encountered during training. By excluding certain drug combinations during training and testing the model's performance on these unseen combinations, we can better evaluate the model's ability to generalize and make accurate predictions for practical drug discovery applications [11, 44]. In comparison to other models, SynProtX-GATFP consistently outperformed its counterparts across all datasets for classification task, including FRIEDMAN-Skin, ALMANAC-Breast, ALMANAC-Lung, ALMANAC-Ovary, and ALMANAC-Skin, as illustrated in **Figure 6**. The generalizability of SynProtX-GATFP was confirmed in the FRIEDMAN-Skin, ALMANAC-Breast, ALMANAC-Lung, ALMANAC-Ovary, and ALMANAC-Skin datasets, in which it consistently achieved higher performance metrics than the DeepSynergy, DeepDDS, and XGBoost models. The robust performance of the model across these diverse datasets underscores its ability to capture essential biological features and generalize across different cancer types and drug combinations. The results of the leave-drug-combinations-out validation underscore the generalizability and robustness of SynProtX-GATFP. By consistently outperforming other models across multiple datasets, SynProtX-GATFP demonstrated its capability to effectively generalize its predictions to novel drug combinations. This is a critical requirement in drug discovery, where models must be able to predict the efficacy of new and untested drug combinations with high accuracy. The superior performance of SynProtX-GATFP, particularly in terms of AUROC, AUCPR, and F_1 score, indicates that the model can reliably identify synergistic interactions, which are vital for developing effective combination therapies.

SynProtX-GATFP Validated Through Existing Research

Table 3 Rediscovering synergistic and antagonistic effects of FDA-approved drug combinations on the test set through SynProtX-GATFP classification model.

**Drug A**	**Drug B**	**Tissue**	**Cell Line**	**Predicted Score (Probability)**	**Publication (PMCID)**
**Synergistic Effect**					
Gefitinib	Vorinostat	Lung	NCI-H226	0.9928	25552401 [45], 30365122 [46]
Docetaxel	Lapatinib	Breast	MDA-MB-468	0.8477	22999386 [47]
Gefitinib	Dasatinib	Ovary	OVCAR-5	0.9374	28446239 [48]
**Antagonistic Effect**					
Mitoxantrone	Ifosfamide	Lung	HOP-62	1.2089E-06	3735704 [49]
Chlorambucil	6-Mercaptopurine	Breast	MDA-MB-468	3.8256E-05	31813938 [50]
Temozolomide	Romidepsin	Ovary	OVCAR-8	9.6136E-05	15196862 [51]

The SynProtX-GATFP model was validated by its ability to rediscover the known synergistic and antagonistic effects of FDA-approved drug combinations, as shown in **Table 3**. This validation is crucial as it not only demonstrates the predictive accuracy of the model, but also its relevance in real-world clinical scenarios. The model accurately predicted the synergistic effects of several drug combinations across different tissues and cell lines, which is consistent with the findings of existing research. For instance, the combination of gefitinib and vorinostat was predicted to have a high probability of synergy in the lung tissue, with a score of 0.9928. This prediction is supported by studies demonstrating the feasibility and efficacy of this combination in activating apoptosis and reducing the levels of key survival proteins such as EGFR, MET, and AKT [45, 46]. Another example is the docetaxel-lapatinib combination in breast tissue, with a predicted synergy score of 0.8477. This finding is corroborated by clinical studies that have confirmed the effectiveness of this combination as a neoadjuvant treatment for $HER2^+$ breast cancer [47]. Lastly, the gefitinib-dasatinib combination was predicted to be synergistic in ovarian tissue with a score of 0.9374, consistent with research showing its anti-tumor properties through the suppression of crucial signaling pathways [48].

In addition to identifying synergistic effects, SynProtX-GATFP also successfully predicted antagonistic interactions. For example, the mitoxantrone-ifosfamide combination was expected to have a negligible probability of synergy in lung tissue, with a score of 1.2089E-06. This prediction is validated by existing clinical data indicating the limited efficacy of this combination in treating non-small cell lung cancer (NSCLC) due to its low anti-tumor activity [49]. Similarly, the combination of chlorambucil and 6-mercaptopurine in breast tissue was predicted to have an antagonistic interaction, supported by clinical trials showing limited effectiveness and significant side effects in patients with breast cancer [50]. Lastly, temozolomide and romidepsin were predicted to exhibit antagonism in the ovarian tissue, which aligns with findings indicating a lack of clinical response in treated patients [51].

SynProtX-GATFP Supported Model Explainability

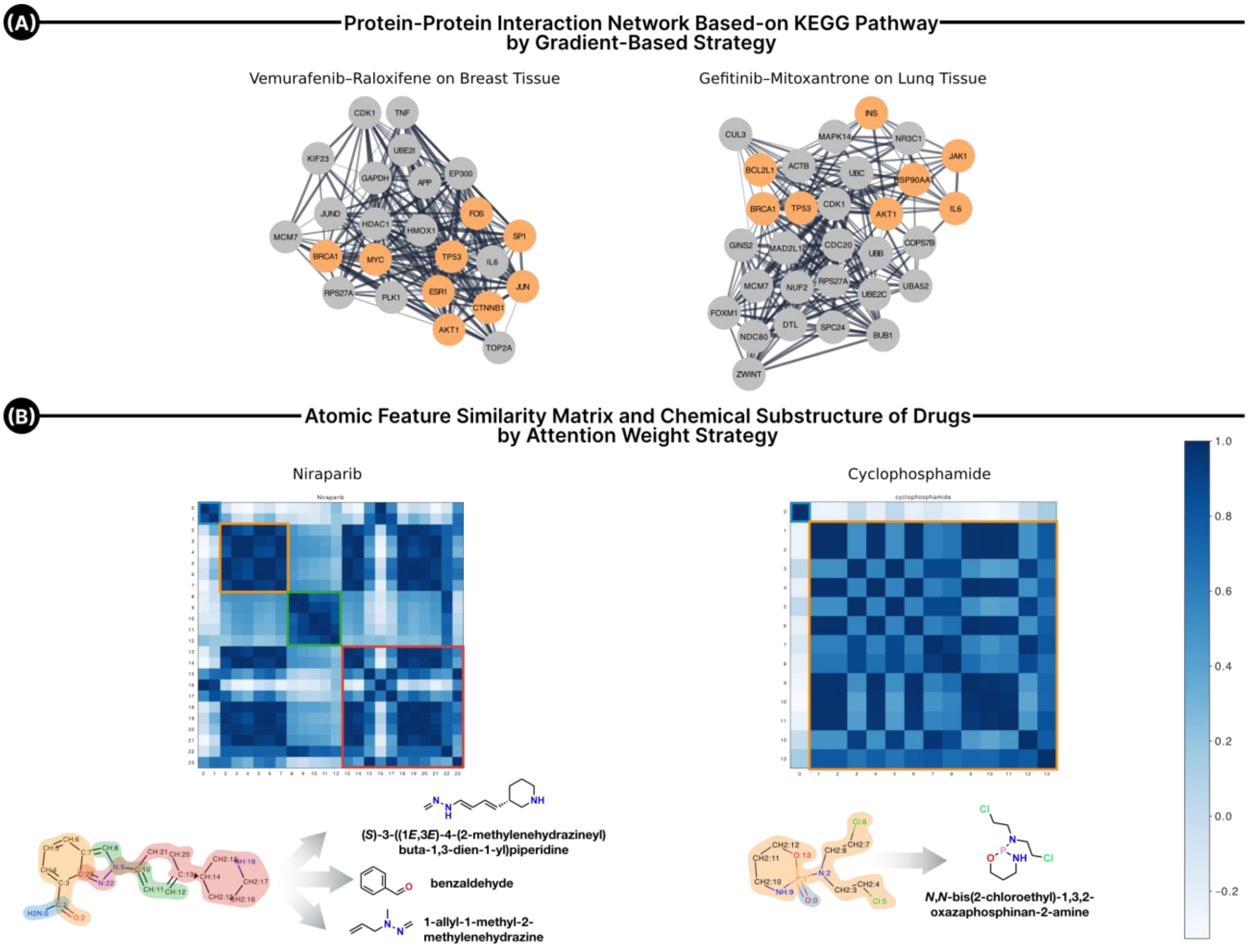

Figure 7. Model interpretability of SynProtX-GATFP. **(A)** Protein-protein interaction networks based on KEGG pathways using a gradient-based strategy for Vemurafenib–Raloxifene on breast tissue and Gefitinib–Mitoxantrone on lung tissue. Orange nodes indicate proteins associated with cancer pathways. **(B)** Atomic feature similarity matrix and chemical substructure analysis of drugs using an attention-weight strategy for Niraparib and Cyclophosphamide.

The SynProtX-GATFP model not only demonstrates high predictive accuracy but also offers significant explainability, which is crucial for understanding the underlying biological mechanisms driving its predictions. By leveraging gradient-based and attention-weight strategies, SynProtX-GATFP provides insights into relevant cancer-associated proteins and important chemical substructures that contribute to the model’s predictions, thus supporting its

interpretability. The gradient-based method from SynProtX-GATFP revealed the key cancer-associated proteins involved in the predicted synergistic effects of the drug combinations, as depicted in **Figure 7A**. For instance, the combination of vemurafenib and raloxifene in breast tissue revealed a protein-protein interaction (PPI) network with several proteins associated with breast cancer pathways, including PLK1, JUN, and MYEF2. These proteins are critical for cell cycle regulation and apoptosis, which are essential for cancer progression [52-54]. Similarly, the combination of gefitinib and mitoxantrone in lung tissue highlighted a PPI network involving proteins linked to the PI3K-Akt signaling pathway, such as AKTS1 and GSK3A, which are known to play a role in cancer cell survival and proliferation [55, 56]. The identification of these proteins and their associated pathways underscores the ability of the model to predict drug synergy and elucidate the molecular basis of these interactions. In addition to protein interactions, SynProtX-GATFP provides valuable insights into the chemical substructures driving drug synergy or antagonism through an attention-weight strategy. As shown in **Figure 7B**, the atomic feature similarity matrix for niraparib and cyclophosphamide revealed critical substructures, such as the methylenhydrazine and benzaldehyde groups in niraparib and the oxazaphosphorine derivative in cyclophosphamide, which are pivotal in their anticancer activity [57]. Piperidine, another compound highlighted in the analysis, has demonstrated significant anticancer potential in various cancer types, including breast, prostate, and colon cancers. The mechanism of action of piperidine involves the regulation of key signaling pathways such as NF-κB, PI3K/Aκt, and STAT-3, which are crucial for cancer cell survival and proliferation. Studies have shown that piperidine can induce apoptosis, inhibit cell migration, and arrest the cell cycle, making it a potent component of anticancer drug design [58]. By highlighting these specific chemical features, the model offers a deeper understanding of how molecular structure influence drug interactions, which is vital for rational drug design and development of more effective combination therapies. By providing insights into the biological and chemical features that drive its predictions, the model offers interpretable insights that help researchers better understand the factors contributing to its predictions, moving towards more transparent and explainable outputs. This level of interpretability is crucial for validating the predictions of the model in a clinical context and gaining trust in its applications for drug development.

METHODS

Data Collection and Preparation

The dataset for developing the deep learning models was sourced from the DrugComb database version 1.5. [24, 25] This database contains drug combination data from various diseases obtained through high-throughput laboratory screenings, including cancer, malaria, Ebola, and SARS-CoV-2. Specifically, for anticancer drug combination data, studies such as FRIEDMAN-Skin [29] and ALMANAC [30] were used to compile the dataset. The records in the dataset represent drug combinations tested against cell lines, with the respective Loewe scores documented. To preprocess the data, we eliminated all invalid entries, such as blank cells. Next, records with Loewe scores higher than 50 or lower than –75 were discarded to remove any potential outliers. Additionally, only drugs available in the ChEMBL database [59-61] were included, and cell lines were limited to those with overlapping between gene expression and protein expression data. Duplicate triplets of a cell line and the corresponding standard drug names were removed. As a result, a drug combination dataset with target values of 131,791 entries was obtained.

To prepare gene expression data from human cancer cells, we slightly modified the method described by Liu Q et al. [62]. Initially, data were collected from The Cancer Dependency Map (DepMap) version 22Q2, a database that processes omics data of cancer cells from the Cancer Cell Line Encyclopedia (CCLE).[13, 14] Since the gene expression data from DepMap includes genes present in each cell, regardless of their relevance to cancer development, it was necessary to filter for genes significant to cancer development. Gene names were sourced from the Catalogue of Somatic Mutations in Cancer (COSMIC) version 96 [63], a database that compares gene data from human cancer cells to the human genome reference (GRCh38). Finally, the identified 714 genes associated with cancer development were used for model development.

For protein expression data of cancer cells, we utilized the ProCan-DepMapSanger database [19], a comprehensive resource covering protein data for 8,498 proteins from 949 types of cancer cells and over 40 cancer types. These protein data were acquired through laboratory experiments using data-independent acquisition mass spectrometry (DIA-MS), which is noted for its high reproducibility and speed [64]. Ultimately, the identified 6,688 proteins were employed for model development.

Chemical Representations

Molecular Fingerprints: To represent the structural information of the molecules, we transformed it from a molecular graph into a molecular fingerprint. This fingerprint consists of Boolean molecular descriptors indicating the chemical and physical properties of a molecule. The Extended Connectivity Fingerprint 6 (ECFP6), a 2048-bit fingerprint generated by processing the Morgan fingerprint with a radius of 3, was used [65, 66]. ECFP6 has been widely used in traditional machine learning models, such as random forest (RF) and extreme gradient boosting (XGBoost), due to its strong performance across in various applications. The molecular fingerprints were utilized to develop the SynProtX-GATFP model and were subsequently compared with state-of-the-art models.

Molecular Graph: To transform the molecular structure data into a molecular graph format, the Simplified Molecular Input Line Entry System (SMILES) notation was used for the drug names present in the dataset. The SMILES notation was obtained from version 31 of the ChEMBL database [59, 60] and was subsequently converted into a molecular graph using the RDKit package. The molecular graph of each drug is defined as $M = (V, E)$, where V is a set of nodes and E is a set of edges displayed in an adjacency matrix A. Each node $v_u \in V$ represents an atom u in the molecule, and each edge $e_{uw} \in E$ represents the bond e_{uw} between atoms u and w.

The Graph Neural Network (GNN) utilizes this non-Euclidean and non-invariant molecular graph to extract compound features for predicting bioactivity. For each node, the atomic and bond features $x_i \in v_u$ are calculated to represent the identity of the node. These features are binary vectors that include implicit values (one-hot and integer). During the GNN learning process, these features are employed through a message-passing phase between each node and its neighboring nodes. In this work, we utilized *ConvMolFeaturizer* for atomic features by DeepChem [67].

Molecular Graph and Deep Neural Networks

Graph Convolutional Network (GCN) [26]: GCN is a graph algorithm that is widely used in image processing, natural language processing (NLP), chemical property prediction of molecules, and systems biology, among others. The propagation process of GCN is defined by the following equation.

$$H_u^{(l+1)} = \sigma\left(\widehat{D}^{-\frac{1}{2}}\hat{A}\widehat{D}^{-\frac{1}{2}}W^{(l)}h_u^{(l)}\right) \tag{12}$$

Let $\hat{A} = A + I$, where I is the identity matrix and $\widehat{D}$ is the degree matrix of the diagonal nodes obtained from $\widehat{D}_{uw} = \sum_w \hat{A}_{uw}$. Here, D and A are the degree and adjacency matrices, respectively, according to the order of $W^{(l)}$ and $h_u^{(l)}$, which are the weights and learnable parameters from node u of layer l, respectively. σ is the non-linear activation function. $\widehat{D}^{-\frac{1}{2}}\hat{A}\widehat{D}^{-\frac{1}{2}}$ adds self-connections to the nodes and maintains feature vector size. The message passing phase consists of two steps: 1) aggregation of the neighboring node vectors h_w to generate the node vector $\hat{h}_u$ and 2) linear projection of $\hat{h}_u$ with a non-linear activation function that can be calculated from the projection function, resulting in the following equation.

$$h_u = \sigma(W_u\hat{h}_u) \tag{13}$$

Graph Attention Network (GAT) [27]: GAT is an algorithm that is an extension of the GCN algorithm. The difference between GAT and GCN is that GAT assigns weights to nodes differently using an attention mechanism to facilitate the process of data aggregation. In GCN, the weights of neighboring atoms are calculated by normalizing the sum to be the same. The propagation process of GAT can be represented with the following equation.

$$H_u^{(l+1)} = \sigma\left(\sum_{v \in N(u)} \alpha_{uw}^{(l)} W^{(l)} h_u^{(l)}\right) \tag{14}$$

In this context, the notations W, $N(u)$, and σ denote the trainable weight matrix, the set of neighboring nodes of node u, and non-linear activation function, respectively. Moreover, the

symbol $\alpha_{uw}^{(l)}$ represents the normalized attention score between node u and its neighbor w in the l_{th} graph convolutional layer, which is computed by the softmax function:

$$\alpha_{uw} = softmax_w(e_{uw}) = \frac{exp(e_{uw})}{\sum_{w \in N(u)} exp(e_{uw})} \tag{15}$$

The variable e_{uw} denotes the output of the initial layer, which is a feed-forward neural network using the non-linear activation function of LeakyReLU and without projection (i.e., a bias vector). This can be formulated as follows:

$$e_{uw} = LeakyReLU(W \cdot [e_u, e_w]) \tag{16}$$

The attention mechanism leverages the ability to selectively attend to different aspects of information to improve performance compared to traditional Graph Convolutional Networks (GCN).

Attentive FP [68]: Attentive FP is a graph neural network architecture that employs a recurrent neural network (RNN) to aggregate information from neighboring and distant atoms to the focal atom of interest. Attentive FP is a state-of-the-art algorithm that can efficiently predict the chemical properties of benchmark molecular datasets with high accuracy on many sub-datasets. One of the reasons why Attentive FP can achieve high performance is due to the fact that during message passing, M, the attention mechanism gathers information from neighboring nodes via gated recurrent units (GRUs), which helps filter unnecessary data and select relevant data for learning. The output stage uses a summation function as the output function to convert the graph vector representation h_G, before the next prediction step, as shown in the following equation.

Message passing step:

$$m_u^{(k+1)} = \sum_{v \in N(u)} M^{(k)}\left(h_u^{(k)}, h_w^{(k)}\right) \tag{17}$$

$$h_u^{(k+1)} = GRU^{(k)}\left(m_u^{(k+1)}, h_u^{(k)}\right) \tag{18}$$

Output step:

$$h_G = Sum\left(h_u^{(K)} \middle| u \in G\right) \tag{19}$$

Deep Neural Network (DNN): Deep neural networks are composed of multiple interconnected neurons that gather information from other neurons. The collected data is subsequently processed through non-linear activation functions to compute the probability. The learning process of deep neural networks involves intricate and rapidly changing non-linear functions that enable them to extract hierarchical features from the input data. The activation vector h of the first hidden unit and each hidden unit at $l + 1$ can be computed using the following equation.

$$h_i^{(1)} = \sigma^{(1)}\left(\sum_j W_{ij}^{(1)} x_j + b_i^{(1)}\right) \tag{20}$$

$$h_i^{(l+1)} = \sigma^{(l+1)}\left(\sum_j W_{ij}^{(l+1)} h_j^{(l)} + b_i^{(l+1)}\right) \tag{21}$$

where x_j is the input data vector used to calculate the activation vector of the first layer $h_i^{(1)}$ and $h_j^{(l)}$ is the activation vector of layer l, W_{ij} is the weight matrix that can be learned, b is the bias vector, and σ is a non-linear activation function

In the classification task, the sigmoid activation function was added to calculate p, which is the probability of the input being a synergistic drug combination, as shown in the following equation.

$$p = sigmoid\left(\sum_j W_j^{out} h_j^l + b^{out}\right) \tag{22}$$

Model Development

The SynProtX model was developed to predict anticancer drug synergy in a particular cell line using proteomics data. This approach involves conducting both classification and regression tasks across datasets categorized by study (FRIEDMAN-Skin dataset) and cancer tissues (ALMANAC-Breast, ALMANAC-Lung, ALMANAC-Ovary, and ALMANAC-Skin). We utilized the PyTorch [69], PyTorch Geometric (PyG) [70], and DeepChem [67] packages as their primary toolkits for developing the model.

For molecular graph learning of compounds, we conducted experimental settings through the following approaches to compare the learning of drug molecule structures:

1. **SynProtX-GATFP:** employs a graph attention network (GAT) for learning molecular structures and deep neural networks (DNNs) for molecular fingerprints
2. **SynProtX-GAT:** employs only a graph attention network (GAT) for learning molecular structures
3. **SynProtX-GCN:** employs only a graph convolutional network (GCN) for learning molecular structures
4. **SynProtX-AttFP:** employs only an Attentive FP (AttFP) for learning molecular structures

In the comparative analysis of methodologies in drug discovery, our approach incorporated the use of advanced models such as DeepSynergy, DeepDDS, and AttenSyn. In addition to these advanced models, we compared our models with classical algorithms such as XGBoost to establish a baseline. XGBoost is recognized for its state-of-the-art performance across various machine learning challenges and is widely adopted for its robustness and efficiency in handling sparse data. This comprehensive comparison allowed us to thoroughly evaluate the efficacy of our model in the context of both contemporary and classical methodologies.

Competitive State-Of-The-Art (SOTA) Models

The study employed three advanced SOTA deep learning models including, DeepSynergy, DeepDDS, and AttenSyn to predict the synergistic effects of anticancer drug combinations.

DeepSynergy [71]**:** DeepSynergy integrates chemical descriptors and gene expression data into a fully connected neural network, utilizing features from both domains to enhance the predictive accuracy. It is particularly effective in assessing the potential of drug pairs to work synergistically by using established metrics such as AUROC and AUPR for evaluation.

DeepDDS [72]**:** DeepDDS leverages graph neural networks, specifically GCNs and GATs, to learn drug-embedding vectors from molecular graphs, integrating cell line-specific data to enhance prediction accuracy. The attention mechanism of the model captures the complex interactions between drugs and cell lines, enabling precise synergy prediction.

AttenSyn [73]**:** AttenSyn extends this approach by incorporating an attention-based pooling module that identifies and focuses on key substructures within drug molecules. This attention mechanism not only improves the model's performance but also enhances its interpretability, making it possible to discern the specific molecular features contributing to the predicted outcomes. These models collectively provide a robust framework for predicting drug synergy, supporting drug discovery, and potentially improving combination therapy strategies in oncology.

To ensure a fair comparison, all competitive SOTA models were evaluated using the same criteria as for SynProtX. This included training and testing on identical datasets using the same performance metrics and applying the same cross-validation techniques. By adhering to these consistent evaluation standards, this study provides a robust comparison of model performance, ensuring that the reported results are directly comparable and reflective of each model's true predictive capabilities.

Model Evaluation

During model training, we randomly partitioned the dataset into training and test sets using an 80:20 ratio with the random splitting method in DeepChem [67]. To ensure robustness, we performed 5-fold cross-validation [74]. The dataset was split into 5 parts, with the model trained on 4 parts and validated on the remaining part. This process was repeated 5 times, each time with a different validation part. We employed Bayesian optimization with 30 trials using Optuna [75] to find the optimal set of hyperparameters, chosen for its efficiency and reduced time consumption with flexible neural network architectures. To further improve the training process and prevent overfitting, we implemented an early stopping mechanism. This early stopping mechanism monitors the validation loss and stops the training process if the performance does not improve for a specified number of epochs, thereby preventing overfitting and saving computational resources. Specifically, training stops if the validation loss does not improve for 30 consecutive epochs or if it increases for 10 consecutive epochs.

To ensure reliable model evaluation, we generated models with different seed numbers independently using the test set. This process was repeated 15 times. The final model in each cross-validation iteration was employed to assess the predictive performance of the model on the test set using selected metrics to compare the ground truth with the predicted drug synergies. We then calculated the mean and confidence interval ($\alpha = 0.05$) of these results. For performance evaluation, we used distinct metrics for the classification and regression tasks. For the classification, we used the Area Under the Receiver Operating Characteristic Curve (AUROC), Area Under the Precision-Recall Curve (AUCPR), Accuracy (ACC), Balanced Accuracy (BACC), F_1 score (F_1), and Cohen's Kappa Coefficient (KAPPA). For the regression, we used the Root Mean Square Error (RMSE), Mean Absolute Error (MAE), Pearson Correlation Coefficient (PCC), Spearman's Rank Correlation Coefficient (SCC), and Coefficient of Determination (R^2). The classification and regression metrics are presented in the Metrics for Model Evaluation subsection of **Supplementary S1**.

AVAILABILITY OF SOURCE CODE AND REQUIREMENTS

- **Project Name:** SynProtX
- **Project Homepage:** https://github.com/manbaritone/SynProtX
- **Operating System(s):** Platform independent
- **Programming Language:** Python
- **Other Requirements:** python 3.7.9, deepchem 2.5.0, dgl 0.9.0, dgllife 0.3.2, numpy 1.21.5, openbabel 2.4.1, optuna 3.0.5, pandas 1.3.5, pyg 2.2.0, pytorch 1.12.0, pytorch-cluster 1.6.0, pytorch-scatter 2.1.0, pytorch-sparse 0.6.16, rdkit 2019.03.2, scikit-learn 1.0.2, scipy 1.7.3, torchaudio 0.12.0, torchmetrics 0.11.0, torchvision 0.13.0
- **License:** MIT

DATA AVAILABILITY

The source code for SynProtX is available at https://github.com/manbaritone/SynProtX. The datasets, hyperparameters, and model checkpoints can be retrieved from https://doi.org/10.5281/zenodo.14040426.

ADDITIONAL FILES

Supplementary Method S1. Metrics for Model Evaluation
Supplementary Figure S1. Comparison of AUROC Curve
Supplementary Figure S2. Comparison of AUCPR Curve
Supplementary Figure S3. Comparison of Scatter Plot between Ground Truth and Prediction Synergy Scores
Supplementary Figure S4. Comparison of Distribution between Ground Truth and Prediction Synergy Scores
Supplementary Figure S5. Comparison of Classification Loss Curve
Supplementary Figure S6. Comparison of Regression Loss Curve
Supplementary Table S1. Statistic of Processed Data of Anticancer Drug Combinations

Supplementary Table S2. Predictive Performance on Classification Task

Supplementary Table S3. Predictive Performance on Regression Task

Supplementary Table S4. Relevant Cancer-Associated Proteins from Gradient-Based Method

Supplementary Table S5. Hyperparameter Settings of SynProtX

Supplementary Table S6. Hyperparameter Settings of SynProtX-GATFP on Classification Task

Supplementary Table S7. Hyperparameter Settings of SynProtX-GATFP on Regression Task

LIST OF ABBREVIATIONS

ACC: Accuracy; AttFP: Attentive FP; AUCPR: Area Under the Precision-Recall Curve; AUROC: Area Under the Receiver Operating Characteristic Curve; BACC: Balanced Accuracy (BACC); CCLE: Cancer Cell Line Encyclopedia; DL: deep learning; DNN: deep neural network; F_1: F_1 score; GAT: graph attention network; GATFP: graph attention network and molecular fingerprint; GCN: graph convolutional network; GNN: graph neural network; KAPPA: Cohen's Kappa Coefficient; MAE: Mean Absolute Error; RMSE: M[76]L: machine learning; MLP: multilayer perceptron; Root Mean Square Error; PCA: principal component analysis; PCC: Pearson Correlation Coefficient; R^2: Coefficient of Determination; ReLU: rectified linear unit; RF: random forest; SCC: Spearman's Rank Correlation Coefficient; SMILE: simplified molecular-input line-entry System; SVM: support vector machine; t-SNE: t-distributed stochastic neighbor embedding; XGBoost: extreme gradient boosting;

AUTHORS' CONTRIBUTIONS

B.B.: Conceptualization, Data curation, Formal analysis, Investigation, Methodology, Validation, Visualization, Writing – original draft, and Writing – review & editing. **M.K.:** Conceptualization, Data curation, Formal analysis, Investigation, Methodology, Writing – original draft, and Writing – review & editing. **T.N.:** Conceptualization, Formal analysis, Investigation, Methodology, and Writing – review & editing**.** **N.Y.:** Formal analysis, Methodology, Validation, and Writing – review & editing. **C.T.:** Formal analysis, Validation, and Writing – review & editing. **T.R.:** Formal

analysis, Supervision, Validation, Visualization, and Writing – review & editing. **S.N.:** Formal analysis, Supervision, Validation, and Writing – review & editing.

FUNDING

This work was supported by Vidyasirimedhi Institute of Science and Technology (VISTEC) and Program Management Unit for Human Resources & Institutional Development, Research and Innovation (PMU-B).

COMPETING INTERESTS

The authors declare that they have no competing interests.

ACKNOWLEDGEMENTS

The authors sincerely thank **Can Udomcharoenchaikit** for the beneficial comments on machine learning-related concerns. The authors also thank **Thanasan Nilsu** from the Department of Biology and Environmental Science at Kamnoetvidya Science Academy (KVIS) for his valuable suggestions and advisement for his support of **Matin Kositchutima**, **Tisorn Naphattalung**, and **Kittiphat Pongarunotai** during their time as research students at KVIS. The computational resources were provided by the School of Information Science and Technology, Vidyasirimedhi Institute of Science and Technology (VISTEC). **Bundit Boonyarit** is supported by a full-time Ph.D. study scholarship from VISTEC.

REFERENCES

1. Swayden M, Chhouri H, Anouar Y and Grumolato L. Tolerant/Persister Cancer Cells and the Path to Resistance to Targeted Therapy. Cells. 2020;9 12 doi:10.3390/cells9122601.

2. Arceci RJ. Mechanisms of resistance to therapy and tumor cell survival. Curr Opin Hematol. 1995;2 4:268-74. doi:10.1097/00062752-199502040-00006.

3. Hanahan D. Hallmarks of Cancer: New Dimensions. Cancer Discov. 2022;12 1:31-46. doi:10.1158/2159-8290.CD-21-1059.

4. Hanahan D and Weinberg RA. Hallmarks of cancer: the next generation. Cell. 2011;144 5:646-74. doi:10.1016/j.cell.2011.02.013.

5. Holohan C, Van Schaeybroeck S, Longley DB and Johnston PG. Cancer drug resistance: an evolving paradigm. Nat Rev Cancer. 2013;13 10:714-26. doi:10.1038/nrc3599.

6. Chou TC. Drug combination studies and their synergy quantification using the Chou-Talalay method. Cancer Res. 2010;70 2:440-6. doi:10.1158/0008-5472.CAN-09-1947.

7. Keith CT, Borisy AA and Stockwell BR. Multicomponent therapeutics for networked systems. Nat Rev Drug Discov. 2005;4 1:71-8. doi:10.1038/nrd1609.

8. Al-Lazikani B, Banerji U and Workman P. Combinatorial drug therapy for cancer in the post-genomic era. Nat Biotechnol. 2012;30 7:679-92. doi:10.1038/nbt.2284.

9. Bulusu KC, Guha R, Mason DJ, Lewis RP, Muratov E, Kalantar Motamedi Y, et al. Modelling of compound combination effects and applications to efficacy and toxicity: state-of-the-art, challenges and perspectives. Drug Discov Today. 2016;21 2:225-38. doi:10.1016/j.drudis.2015.09.003.

10. Oh M, Park S, Lee S, Lee D, Lim S, Jeong D, et al. DRIM: A Web-Based System for Investigating Drug Response at the Molecular Level by Condition-Specific Multi-Omics Data Integration. Front Genet. 2020;11:564792. doi:10.3389/fgene.2020.564792.

11. Preuer K, Lewis RPI, Hochreiter S, Bender A, Bulusu KC and Klambauer G. DeepSynergy: predicting anti-cancer drug synergy with Deep Learning. Bioinformatics. 2018;34 9:1538-46. doi:10.1093/bioinformatics/btx806.

12. Cai Z, Poulos RC, Liu J and Zhong Q. Machine learning for multi-omics data integration in cancer. iScience. 2022;25 2:103798. doi:10.1016/j.isci.2022.103798.

13. Barretina J, Caponigro G, Stransky N, Venkatesan K, Margolin AA, Kim S, et al. The Cancer Cell Line Encyclopedia enables predictive modelling of anticancer drug sensitivity. Nature. 2012;483 7391:603-7. doi:10.1038/nature11003.

14. Ghandi M, Huang FW, Jane-Valbuena J, Kryukov GV, Lo CC, McDonald ER, 3rd, et al. Next-generation characterization of the Cancer Cell Line Encyclopedia. Nature. 2019;569 7757:503-8. doi:10.1038/s41586-019-1186-3.

15. Malone JH and Oliver B. Microarrays, deep sequencing and the true measure of the transcriptome. BMC Biol. 2011;9:34. doi:10.1186/1741-7007-9-34.

16. Celebi R, Bear Don't Walk Ot, Movva R, Alpsoy S and Dumontier M. In-silico Prediction of Synergistic Anti-Cancer Drug Combinations Using Multi-omics Data. Sci Rep. 2019;9 1:8949. doi:10.1038/s41598-019-45236-6.

17. Wang J, Liu X, Shen S, Deng L and Liu H. DeepDDS: deep graph neural network with attention mechanism to predict synergistic drug combinations. Brief Bioinform. 2022;23 1 doi:10.1093/bib/bbab390.

18. Wang T, Wang R and Wei L. AttenSyn: An Attention-Based Deep Graph Neural Network for Anticancer Synergistic Drug Combination Prediction. J Chem Inf Model. 2024;64 7:2854-62. doi:10.1021/acs.jcim.3c00709.

19. Goncalves E, Poulos RC, Cai Z, Barthorpe S, Manda SS, Lucas N, et al. Pan-cancer proteomic map of 949 human cell lines. Cancer Cell. 2022;40 8:835-49 e8. doi:10.1016/j.ccell.2022.06.010.

20. Aebersold R and Mann M. Mass-spectrometric exploration of proteome structure and function. Nature. 2016;537 7620:347-55. doi:10.1038/nature19949.

21. Crick F. Central dogma of molecular biology. Nature. 1970;227 5258:561-3. doi:10.1038/227561a0.

22. Spirin V and Mirny LA. Protein complexes and functional modules in molecular networks. Proc Natl Acad Sci U S A. 2003;100 21:12123-8. doi:10.1073/pnas.2032324100.

23. Mertins P, Mani DR, Ruggles KV, Gillette MA, Clauser KR, Wang P, et al. Proteogenomics connects somatic mutations to signalling in breast cancer. Nature. 2016;534 7605:55-62. doi:10.1038/nature18003.

24. Zagidullin B, Aldahdooh J, Zheng S, Wang W, Wang Y, Saad J, et al. DrugComb: an integrative cancer drug combination data portal. Nucleic Acids Res. 2019;47 W1:W43-W51. doi:10.1093/nar/gkz337.

25. Zheng S, Aldahdooh J, Shadbahr T, Wang Y, Aldahdooh D, Bao J, et al. DrugComb update: a more comprehensive drug sensitivity data repository and analysis portal. Nucleic Acids Res. 2021;49 W1:W174-W84. doi:10.1093/nar/gkab438.

26. Kipf TN and Welling M. Semi-supervised classification with graph convolutional networks. arXiv preprint arXiv:160902907. 2016.

27. Veličković P, Cucurull G, Casanova A, Romero A, Lio P and Bengio Y. Graph attention networks. arXiv preprint arXiv:171010903. 2017.

28. Xiong Z, Wang D, Liu X, Zhong F, Wan X, Li X, et al. Pushing the Boundaries of Molecular Representation for Drug Discovery with the Graph Attention Mechanism. J Med Chem. 2020;63 16:8749-60. doi:10.1021/acs.jmedchem.9b00959.

29. Friedman AA, Amzallag A, Pruteanu-Malinici I, Baniya S, Cooper ZA, Piris A, et al. Landscape of targeted anti-cancer drug synergies in melanoma identifies a novel BRAF-VEGFR/PDGFR combination treatment. PloS one. 2015;10 10:e0140310.

30. Holbeck SL, Camalier R, Crowell JA, Govindharajulu JP, Hollingshead M, Anderson LW, et al. The National Cancer Institute ALMANAC: A Comprehensive Screening Resource for the Detection of Anticancer Drug Pairs with Enhanced Therapeutic ActivityNCI ALMANAC of Approved Cancer Drug Combinations. Cancer research. 2017;77 13:3564-76.

31. Loewe S. The problem of synergism and antagonism of combined drugs. Arzneimittelforschung. 1953;3:285-90.

32. Malyutina A, Majumder MM, Wang W, Pessia A, Heckman CA and Tang J. Drug combination sensitivity scoring facilitates the discovery of synergistic and efficacious drug combinations in cancer. PLoS computational biology. 2019;15 5:e1006752.

33. Hyung D, Baek MJ, Lee J, Cho J, Kim HS, Park C, et al. Protein-gene Expression Nexus: Comprehensive characterization of human cancer cell lines with proteogenomic analysis. Comput Struct Biotechnol J. 2021;19:4759-69. doi:10.1016/j.csbj.2021.08.022.

34. Lobo V, Shcherbinina E, Westholm JO, Nowak I, Huang HC, Angeletti D, et al. Integrative transcriptomic and proteomic profiling of the effects of cell confluency on gene expression. Sci Data. 2024;11 1:617. doi:10.1038/s41597-024-03465-z.

35. Wang H and Hanash SM. Contributions of proteome profiling to the molecular analysis of cancer. Technol Cancer Res Treat. 2002;1 4:237-46. doi:10.1177/153303460200100404.

36. Chen MM, Li J, Mills GB and Liang H. Predicting Cancer Cell Line Dependencies From the Protein Expression Data of Reverse-Phase Protein Arrays. JCO Clin Cancer Inform. 2020;4:357-66. doi:10.1200/CCI.19.00144.

37. Bunte K, Haase S, Biehl M and Villmann T. Stochastic neighbor embedding (SNE) for dimension reduction and visualization using arbitrary divergences. Neurocomputing. 2012;90:23-45.

38. Sun Y, Sheng Z, Ma C, Tang K, Zhu R, Wu Z, et al. Combining genomic and network characteristics for extended capability in predicting synergistic drugs for cancer. Nat Commun. 2015;6:8481. doi:10.1038/ncomms9481.

39. Sun YV and Hu YJ. Integrative Analysis of Multi-omics Data for Discovery and Functional Studies of Complex Human Diseases. Adv Genet. 2016;93:147-90. doi:10.1016/bs.adgen.2015.11.004.

40. Torkamannia A, Omidi Y and Ferdousi R. A review of machine learning approaches for drug synergy prediction in cancer. Brief Bioinform. 2022;23 3 doi:10.1093/bib/bbac075.

41. Wu L, Wen Y, Leng D, Zhang Q, Dai C, Wang Z, et al. Machine learning methods, databases and tools for drug combination prediction. Brief Bioinform. 2022;23 1 doi:10.1093/bib/bbab355.

42. Schwanhausser B, Busse D, Li N, Dittmar G, Schuchhardt J, Wolf J, et al. Global quantification of mammalian gene expression control. Nature. 2011;473 7347:337-42. doi:10.1038/nature10098.

43. Vogel C and Marcotte EM. Insights into the regulation of protein abundance from proteomic and transcriptomic analyses. Nat Rev Genet. 2012;13 4:227-32. doi:10.1038/nrg3185.

44. Liu Q and Xie L. TranSynergy: Mechanism-driven interpretable deep neural network for the synergistic prediction and pathway deconvolution of drug combinations. PLoS Comput Biol. 2021;17 2:e1008653. doi:10.1371/journal.pcbi.1008653.

45. Han JY, Lee SH, Lee GK, Yun T, Lee YJ, Hwang KH, et al. Phase I/II study of gefitinib (Iressa((R))) and vorinostat (IVORI) in previously treated patients with advanced non-small cell lung cancer. Cancer Chemother Pharmacol. 2015;75 3:475-83. doi:10.1007/s00280-014-2664-9.

46. Park SE, Kim DE, Kim MJ, Lee JS, Rho JK, Jeong SY, et al. Vorinostat enhances gefitinib-induced cell death through reactive oxygen species-dependent cleavage of HSP90 and its clients in non-small cell lung cancer with the EGFR mutation. Oncol Rep. 2019;41 1:525-33. doi:10.3892/or.2018.6814.

47. Bonnefoi H, Zaman K, Debled M, Fiche M, Fournier M, Nobahar M, et al. An European Organisation for Research and Treatment of Cancer phase I study of lapatinib and

docetaxel as neoadjuvant treatment for human epidermal growth factor receptor 2 (HER2) positive locally-advanced/inflammatory or large operable breast cancer. Eur J Cancer. 2013;49 2:281-9. doi:10.1016/j.ejca.2012.08.006.

48. Thibault B and Jean-Claude B. Dasatinib + Gefitinib, a non platinum-based combination with enhanced growth inhibitory, anti-migratory and anti-invasive potency against human ovarian cancer cells. J Ovarian Res. 2017;10 1:31. doi:10.1186/s13048-017-0319-2.

49. Suga J, Saijo N, Shinkai T, Eguchi K, Sasaki Y, Sakurai M, et al. Phase II study of mitoxantrone in patients with non-small cell lung cancer. Jpn J Clin Oncol. 1986;16 2:147-51. doi:10.1093/oxfordjournals.jjco.a039131.

50. Roberts C, Strauss VY, Kopijasz S, Gourley C, Hall M, Montes A, et al. Results of a phase II clinical trial of 6-mercaptopurine (6MP) and methotrexate in patients with BRCA-defective tumours. Br J Cancer. 2020;122 4:483-90. doi:10.1038/s41416-019-0674-4.

51. Judson PL, Blair Harkness C, Boente MP, Downs LS, Jr., Argenta PA and Carson LF. A phase II evaluation of Temozolomide in patients with recurrent epithelial ovarian cancer. Gynecol Oncol. 2004;93 3:667-70. doi:10.1016/j.ygyno.2004.03.020.

52. Uchiumi T, Longo DL and Ferris DK. Cell cycle regulation of the human polo-like kinase (PLK) promoter. J Biol Chem. 1997;272 14:9166-74. doi:10.1074/jbc.272.14.9166.

53. King KL and Cidlowski JA. Cell cycle regulation and apoptosis. Annu Rev Physiol. 1998;60:601-17. doi:10.1146/annurev.physiol.60.1.601.

54. Wisdom R, Johnson RS and Moore C. c-Jun regulates cell cycle progression and apoptosis by distinct mechanisms. EMBO J. 1999;18 1:188-97. doi:10.1093/emboj/18.1.188.

55. Pap M and Cooper GM. Role of glycogen synthase kinase-3 in the phosphatidylinositol 3-Kinase/Akt cell survival pathway. J Biol Chem. 1998;273 32:19929-32. doi:10.1074/jbc.273.32.19929.

56. Fang X, Yu SX, Lu Y, Bast RC, Jr., Woodgett JR and Mills GB. Phosphorylation and inactivation of glycogen synthase kinase 3 by protein kinase A. Proc Natl Acad Sci U S A. 2000;97 22:11960-5. doi:10.1073/pnas.220413597.

57. Taetle R and Howell SB. Preclinical re-evaluation of benzaldehyde as a chemotherapeutic agent. Cancer Treat Rep. 1983;67 6:561-6.

58. Mitra S, Anand U, Jha NK, Shekhawat MS, Saha SC, Nongdam P, et al. Anticancer Applications and Pharmacological Properties of Piperidine and Piperine: A Comprehensive Review on Molecular Mechanisms and Therapeutic Perspectives. Front Pharmacol. 2021;12:772418. doi:10.3389/fphar.2021.772418.

59. Davies M, Nowotka M, Papadatos G, Dedman N, Gaulton A, Atkinson F, et al. ChEMBL web services: streamlining access to drug discovery data and utilities. Nucleic Acids Res. 2015;43 W1:W612-20. doi:10.1093/nar/gkv352.

60. Mendez D, Gaulton A, Bento AP, Chambers J, De Veij M, Felix E, et al. ChEMBL: towards direct deposition of bioassay data. Nucleic Acids Res. 2019;47 D1:D930-D40. doi:10.1093/nar/gky1075.

61. Zdrazil B, Felix E, Hunter F, Manners EJ, Blackshaw J, Corbett S, et al. The ChEMBL Database in 2023: a drug discovery platform spanning multiple bioactivity data types and time periods. Nucleic acids research. 2024;52 D1:D1180-D92.

62. Liu Q, Hu Z, Jiang R and Zhou M. DeepCDR: a hybrid graph convolutional network for predicting cancer drug response. Bioinformatics. 2020;36 Supplement_2:i911-i8.

63. Sondka Z, Bamford S, Cole CG, Ward SA, Dunham I and Forbes SA. The COSMIC Cancer Gene Census: describing genetic dysfunction across all human cancers. Nature Reviews Cancer. 2018;18 11:696-705.

64. Krasny L and Huang PH. Data-independent acquisition mass spectrometry (DIA-MS) for proteomic applications in oncology. Molecular omics. 2021;17 1:29-42.

65. Rogers D, Brown RD and Hahn M. Using extended-connectivity fingerprints with Laplacian-modified Bayesian analysis in high-throughput screening follow-up. J Biomol Screen. 2005;10 7:682-6. doi:10.1177/1087057105281365.

66. Rogers D and Hahn M. Extended-connectivity fingerprints. J Chem Inf Model. 2010;50 5:742-54. doi:10.1021/ci100050t.

67. Ramsundar B, Eastman P, Walters P and Pande V. Deep learning for the life sciences: applying deep learning to genomics, microscopy, drug discovery, and more. O'Reilly Media; 2019.

68. Xiong Z, Wang D, Liu X, Zhong F, Wan X, Li X, et al. Pushing the boundaries of molecular representation for drug discovery with the graph attention mechanism. Journal of medicinal chemistry. 2019;63 16:8749-60.

69. Paszke A, Gross S, Massa F, Lerer A, Bradbury J, Chanan G, et al. Pytorch: An imperative style, high-performance deep learning library. Advances in neural information processing systems. 2019;32.

70. Fey M and Lenssen JE. Fast graph representation learning with PyTorch Geometric. arXiv preprint arXiv:190302428. 2019.

71. Preuer K, Lewis RP, Hochreiter S, Bender A, Bulusu KC and Klambauer G. DeepSynergy: predicting anti-cancer drug synergy with Deep Learning. Bioinformatics. 2018;34 9:1538-46.

72. Wang J, Liu X, Shen S, Deng L and Liu H. DeepDDS: deep graph neural network with attention mechanism to predict synergistic drug combinations. Briefings in Bioinformatics. 2022;23 1:bbab390.

73. Wang T, Wang R and Wei L. AttenSyn: an attention-based deep graph neural network for anticancer synergistic drug combination prediction. Journal of Chemical Information and Modeling. 2023.

74. Baumann D and Baumann K. Reliable estimation of prediction errors for QSAR models under model uncertainty using double cross-validation. J Cheminform. 2014;6 1:47. doi:10.1186/s13321-014-0047-1.

75. Akiba T, Sano S, Yanase T, Ohta T and Koyama M. Optuna: A next-generation hyperparameter optimization framework. In: *Proceedings of the 25th ACM SIGKDD international conference on knowledge discovery & data mining* 2019, pp.2623-31.

76. OpenAI. ChatGPT (GPT-4o, May 2024 Version) [Large language model]. Response to query made by Bundit Boonyarit. https://chat.openai.com/chat. 2024.

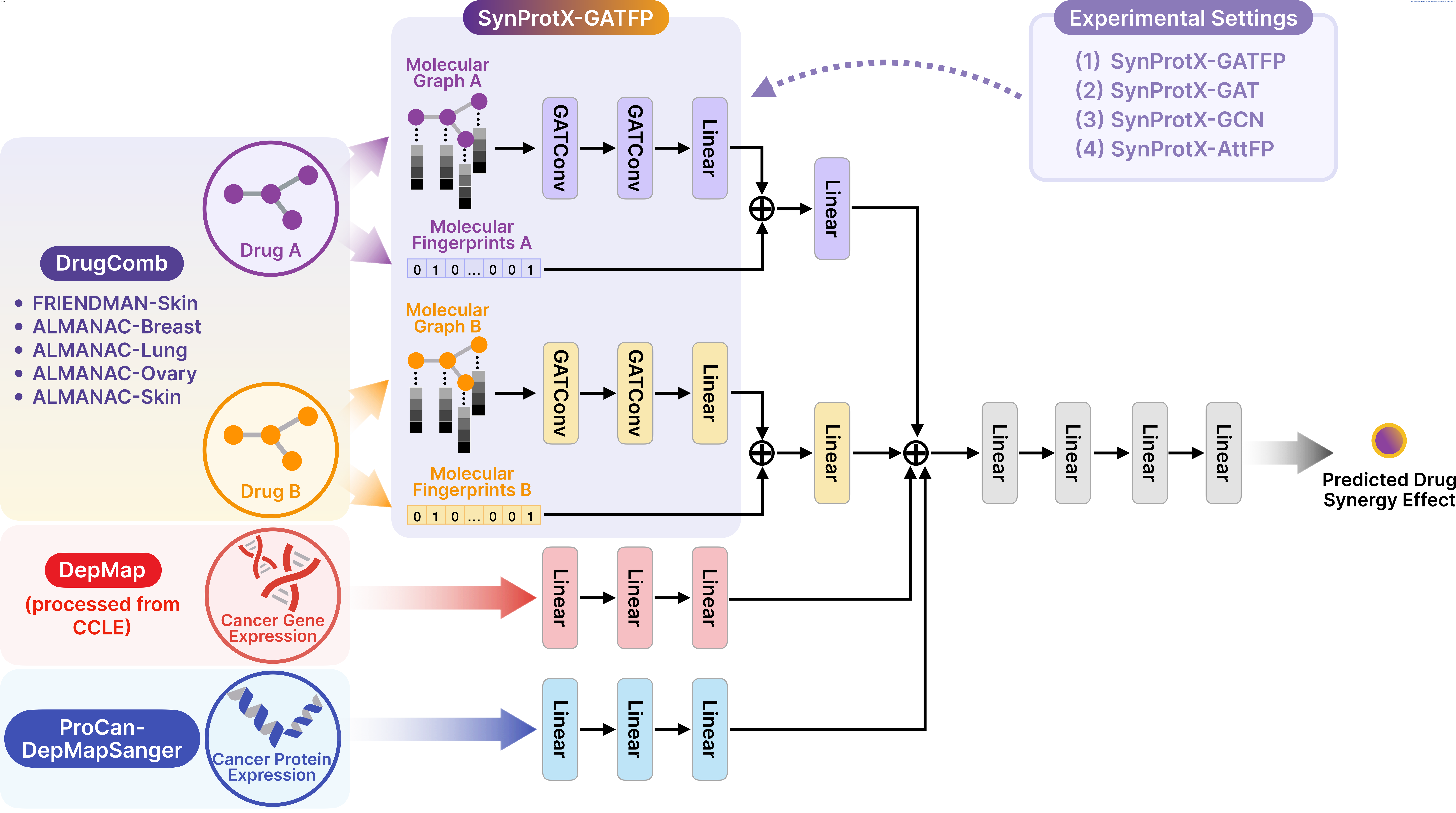
SynProtX-GATFP
Experimental Settings
(1) SynProtX-GATFP
(2) SynProtX-GAT
(3) SynProtX-GCN
(4) SynProtX-AttFP
Molecular Graph A
GATConv
GATConv
Linear
Linear
Drug A
Molecular Fingerprints A
0 1 0 ... 0 0 1
DrugComb
FRIENDMAN-Skin
ALMANAC-Breast
ALMANAC-Lung
ALMANAC-Ovary
ALMANAC-Skin
Molecular Graph B
GATConv
GATConv
Linear
Linear
Drug B
Molecular Fingerprints B
0 1 0 ... 0 0 1
Linear
Linear
Linear
Linear
Predicted Drug Synergy Effect
DepMap
(processed from CCLE)
Cancer Gene Expression
Linear
Linear
Linear
ProCan-DepMapSanger
Cancer Protein Expression
Linear
Linear
Linear

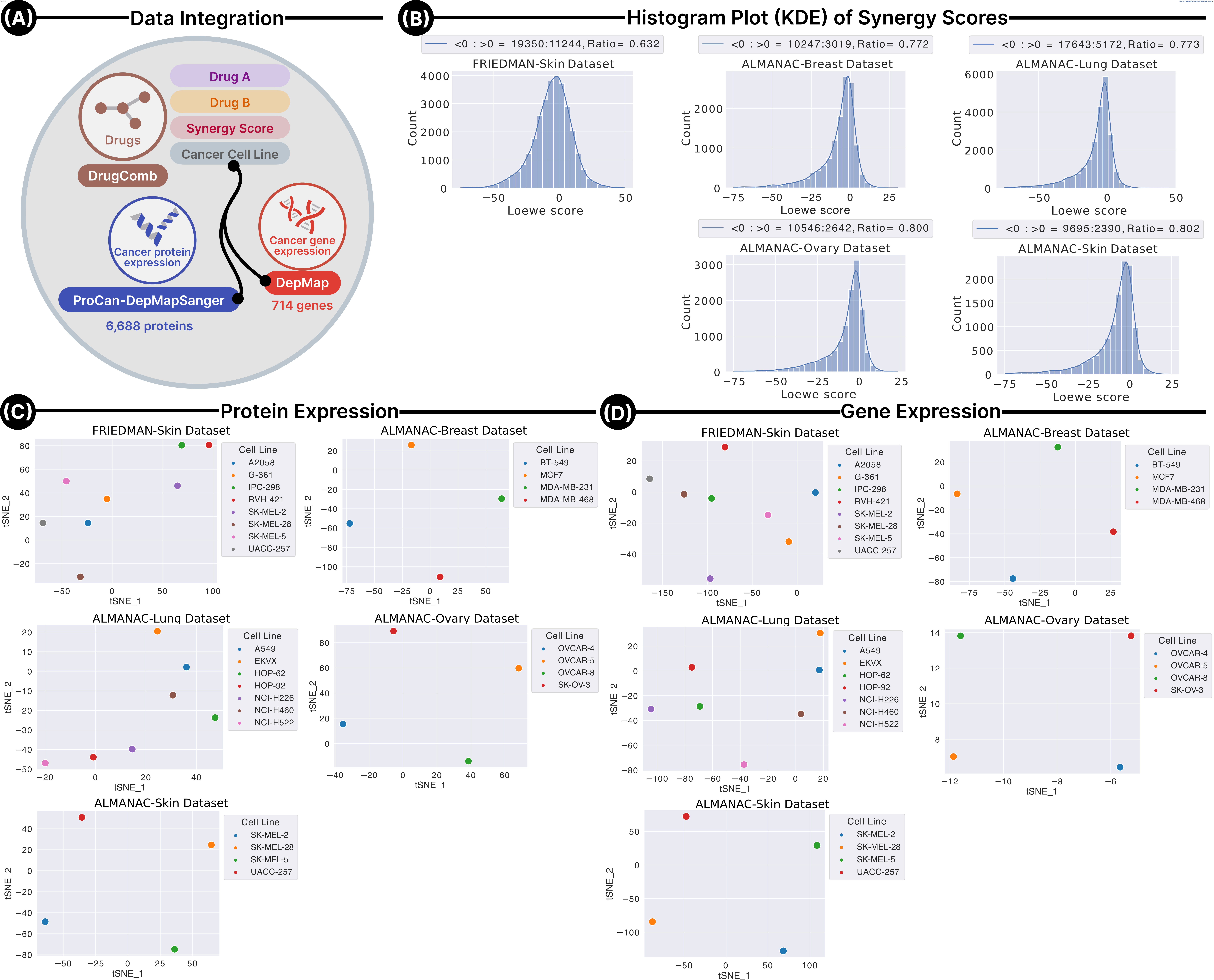
(A)
Data Integration
Drugs
DrugComb
Drug A
Drug B
Synergy Score
Cancer Cell Line
Cancer protein expression
ProCan-DepMapSanger
6,688 proteins
Cancer gene expression
DepMap
714 genes
(B)
Histogram Plot (KDE) of Synergy Scores
<0 : >0 = 19350:11244, Ratio= 0.632
FRIEDMAN-Skin Dataset
<0 : >0 = 10247:3019, Ratio= 0.772
ALMANAC-Breast Dataset
<0 : >0 = 17643:5172, Ratio= 0.773
ALMANAC-Lung Dataset
<0 : >0 = 10546:2642, Ratio= 0.800
ALMANAC-Ovary Dataset
<0 : >0 = 9695:2390, Ratio= 0.802
ALMANAC-Skin Dataset
Count
Loewe score
(C)
Protein Expression
(D)
Gene Expression
FRIEDMAN-Skin Dataset
ALMANAC-Breast Dataset
ALMANAC-Lung Dataset
ALMANAC-Ovary Dataset
ALMANAC-Skin Dataset
tSNE_1
tSNE_2
Cell Line
A2058
G-361
IPC-298
RVH-421
SK-MEL-2
SK-MEL-28
SK-MEL-5
UACC-257
BT-549
MCF7
MDA-MB-231
MDA-MB-468
A549
EKVX
HOP-62
HOP-92
NCI-H226
NCI-H460
NCI-H522
OVCAR-4
OVCAR-5
OVCAR-8
SK-OV-3

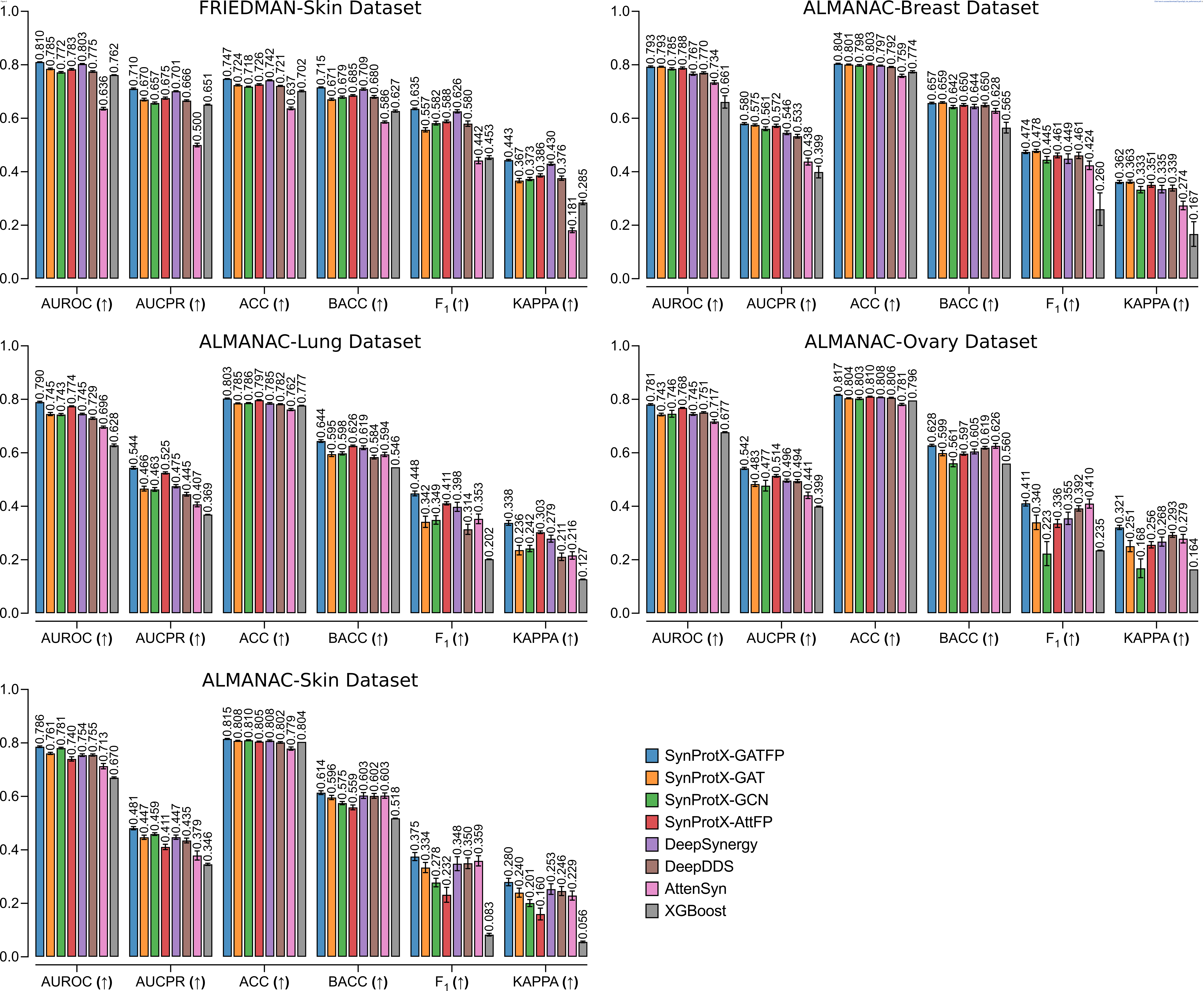
FRIEDMAN-Skin Dataset
0.810 0.785 0.772 0.783 0.803 0.775 0.636 0.762
0.710 0.670 0.657 0.675 0.701 0.666 0.500 0.651
0.747 0.724 0.718 0.726 0.742 0.721 0.637 0.702
0.715 0.671 0.679 0.685 0.709 0.680 0.586 0.627
0.635 0.557 0.582 0.588 0.626 0.580 0.442 0.453
0.443 0.367 0.373 0.386 0.430 0.376 0.181 0.285
ALMANAC-Breast Dataset
0.793 0.793 0.785 0.788 0.767 0.770 0.734 0.661
0.580 0.575 0.561 0.572 0.546 0.533 0.438 0.399
0.804 0.801 0.798 0.803 0.797 0.792 0.759 0.774
0.657 0.659 0.642 0.650 0.644 0.650 0.628 0.565
0.474 0.478 0.445 0.461 0.449 0.461 0.424 0.260
0.362 0.363 0.333 0.351 0.335 0.339 0.274 0.167
ALMANAC-Lung Dataset
0.790 0.745 0.743 0.774 0.745 0.729 0.696 0.628
0.544 0.466 0.463 0.525 0.475 0.445 0.407 0.369
0.803 0.785 0.786 0.797 0.785 0.782 0.762 0.777
0.644 0.595 0.598 0.626 0.619 0.584 0.594 0.546
0.448 0.342 0.349 0.411 0.398 0.314 0.353 0.202
0.338 0.236 0.242 0.303 0.279 0.211 0.216 0.127
ALMANAC-Ovary Dataset
0.781 0.743 0.746 0.768 0.745 0.751 0.717 0.677
0.542 0.483 0.477 0.514 0.496 0.494 0.441 0.399
0.817 0.804 0.803 0.810 0.808 0.806 0.781 0.796
0.628 0.599 0.561 0.597 0.605 0.619 0.626 0.560
0.411 0.340 0.223 0.336 0.355 0.392 0.410 0.235
0.321 0.251 0.168 0.256 0.268 0.293 0.279 0.164
ALMANAC-Skin Dataset
0.786 0.761 0.781 0.740 0.754 0.755 0.713 0.670
0.481 0.447 0.459 0.411 0.447 0.435 0.379 0.346
0.815 0.808 0.810 0.805 0.808 0.802 0.779 0.804
0.614 0.596 0.575 0.559 0.603 0.602 0.603 0.518
0.375 0.334 0.278 0.232 0.348 0.350 0.359 0.083
0.280 0.240 0.201 0.160 0.253 0.246 0.229 0.056
0.0 0.2 0.4 0.6 0.8 1.0
AUROC (↑)
AUCPR (↑)
ACC (↑)
BACC (↑)
F_1 (↑)
KAPPA (↑)
SynProtX-GATFP
SynProtX-GAT
SynProtX-GCN
SynProtX-AttFP
DeepSynergy
DeepDDS
AttenSyn
XGBoost

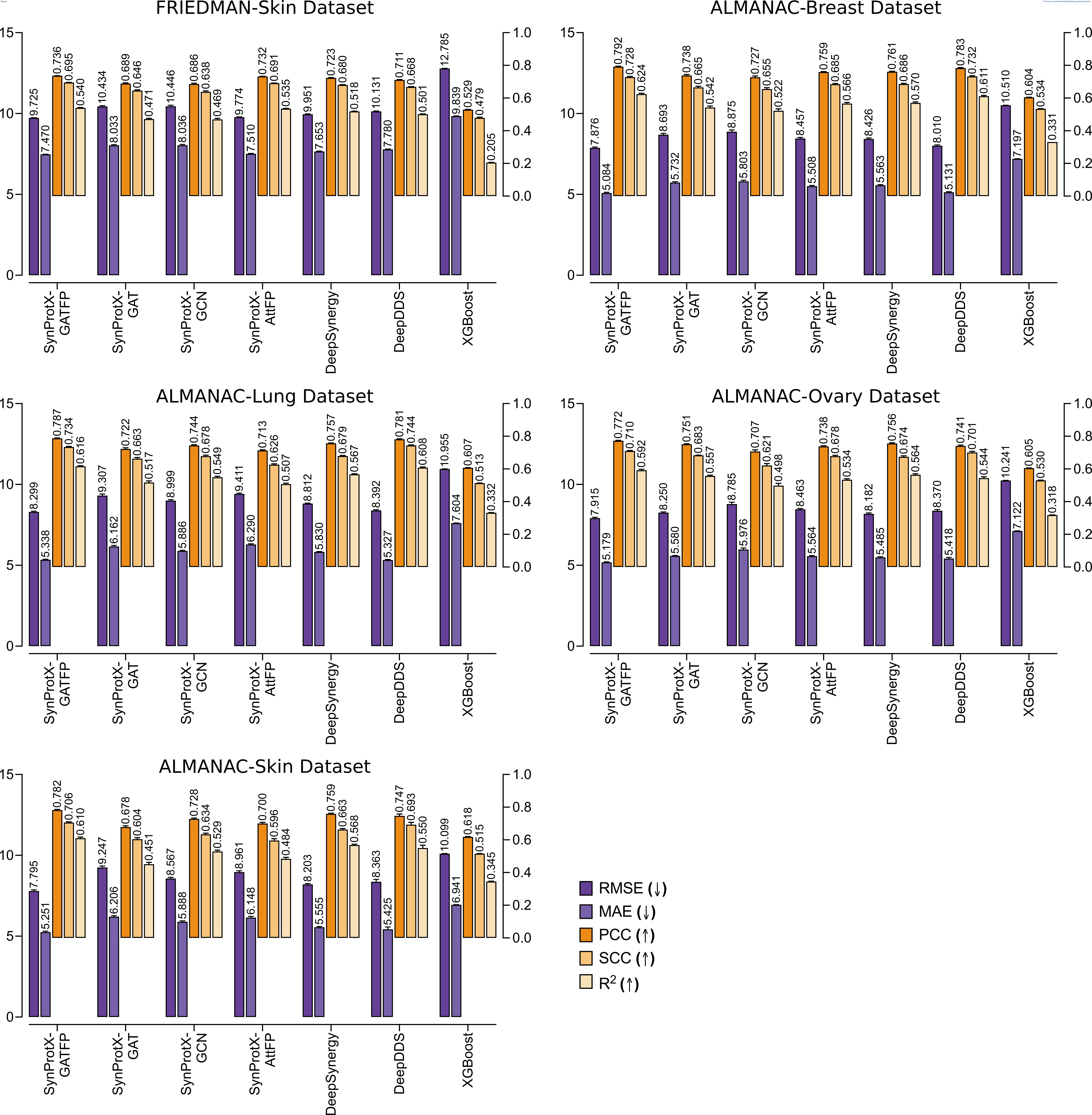

FRIEDMAN-Skin Dataset
SynProtX-GATFP 9.725 7.470 0.736 0.695 0.540
SynProtX-GAT 10.434 8.033 0.689 0.646 0.471
SynProtX-GCN 10.446 8.036 0.686 0.638 0.469
SynProtX-AttFP 9.774 7.510 0.732 0.691 0.535
DeepSynergy 9.951 7.653 0.723 0.680 0.518
DeepDDS 10.131 7.780 0.711 0.668 0.501
XGBoost 12.785 9.839 0.529 0.479 0.205
ALMANAC-Breast Dataset
SynProtX-GATFP 7.876 5.084 0.792 0.728 0.624
SynProtX-GAT 8.693 5.732 0.738 0.665 0.542
SynProtX-GCN 8.875 5.803 0.727 0.655 0.522
SynProtX-AttFP 8.457 5.508 0.759 0.685 0.566
DeepSynergy 8.426 5.563 0.761 0.686 0.570
DeepDDS 8.010 5.131 0.783 0.732 0.611
XGBoost 10.510 7.197 0.604 0.534 0.331
ALMANAC-Lung Dataset
SynProtX-GATFP 8.299 5.338 0.787 0.734 0.616
SynProtX-GAT 9.307 6.162 0.722 0.663 0.517
SynProtX-GCN 8.999 5.886 0.744 0.678 0.549
SynProtX-AttFP 9.411 6.290 0.713 0.626 0.507
DeepSynergy 8.812 5.830 0.757 0.679 0.567
DeepDDS 8.392 5.327 0.781 0.744 0.608
XGBoost 10.955 7.604 0.607 0.513 0.332
ALMANAC-Ovary Dataset
SynProtX-GATFP 7.915 5.179 0.772 0.710 0.592
SynProtX-GAT 8.250 5.580 0.751 0.683 0.557
SynProtX-GCN 8.785 5.976 0.707 0.621 0.498
SynProtX-AttFP 8.463 5.564 0.738 0.678 0.534
DeepSynergy 8.182 5.485 0.756 0.674 0.564
DeepDDS 8.370 5.418 0.741 0.701 0.544
XGBoost 10.241 7.122 0.605 0.530 0.318
ALMANAC-Skin Dataset
SynProtX-GATFP 7.795 5.251 0.782 0.706 0.610
SynProtX-GAT 9.247 6.206 0.678 0.604 0.451
SynProtX-GCN 8.567 5.888 0.728 0.634 0.529
SynProtX-AttFP 8.961 6.148 0.700 0.596 0.484
DeepSynergy 8.203 5.555 0.759 0.663 0.568
DeepDDS 8.363 5.425 0.747 0.693 0.550
XGBoost 10.099 6.941 0.618 0.515 0.345
RMSE (↓)
MAE (↓)
PCC (↑)
SCC (↑)
R^2 (↑)

Click here to access/download;Figure;fig5_feature_learning.pdf

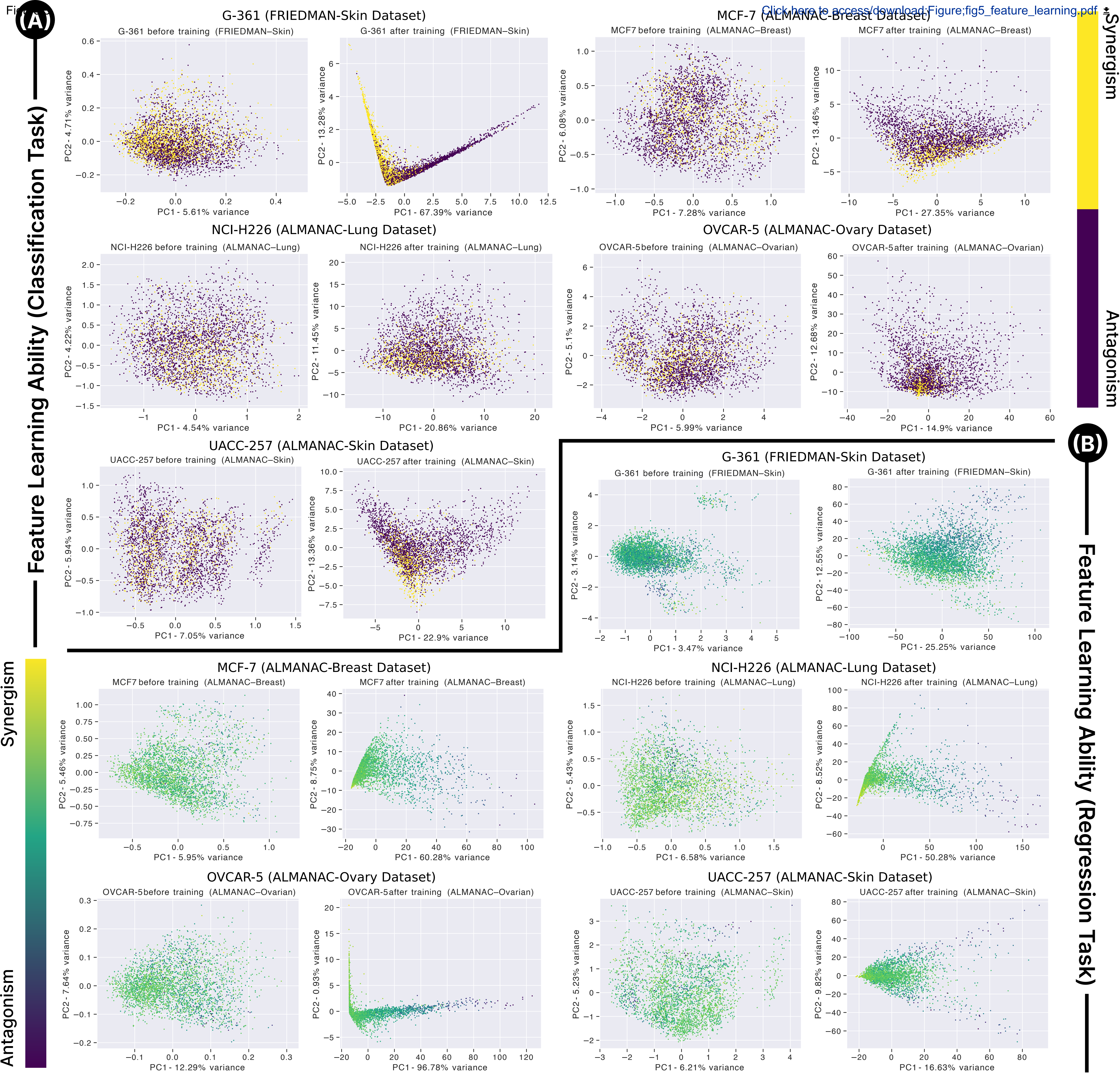

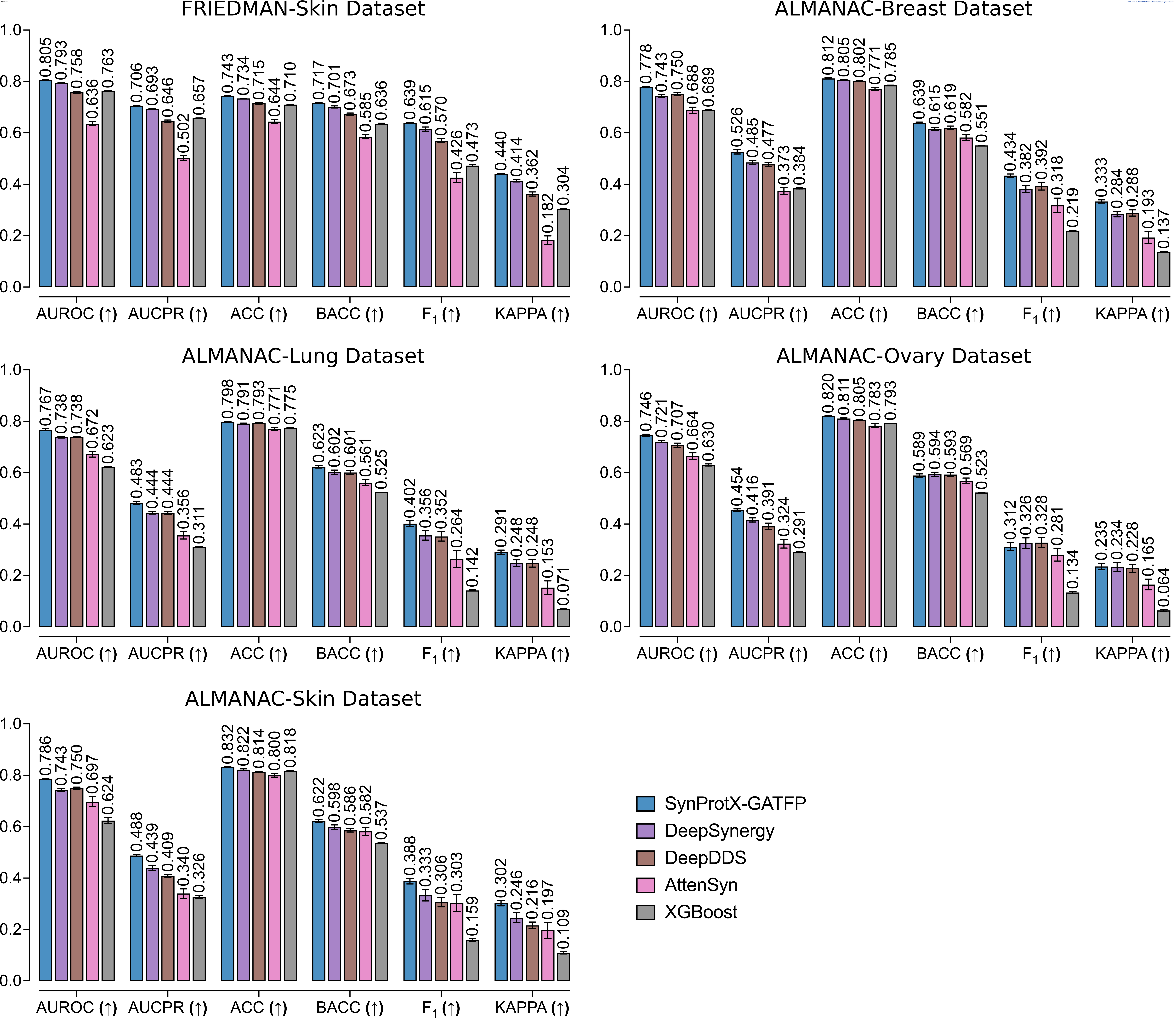
FRIEDMAN-Skin Dataset
0.805 0.793 0.758 0.636 0.763
0.706 0.693 0.646 0.502 0.657
0.743 0.734 0.715 0.644 0.710
0.717 0.701 0.673 0.585 0.636
0.639 0.615 0.570 0.426 0.473
0.440 0.414 0.362 0.182 0.304
ALMANAC-Breast Dataset
0.778 0.743 0.750 0.688 0.689
0.526 0.485 0.477 0.373 0.384
0.812 0.805 0.802 0.771 0.785
0.639 0.615 0.619 0.582 0.551
0.434 0.382 0.392 0.318 0.219
0.333 0.284 0.288 0.193 0.137
ALMANAC-Lung Dataset
0.767 0.738 0.738 0.672 0.623
0.483 0.444 0.444 0.356 0.311
0.798 0.791 0.793 0.771 0.775
0.623 0.602 0.601 0.561 0.525
0.402 0.356 0.352 0.264 0.142
0.291 0.248 0.248 0.153 0.071
ALMANAC-Ovary Dataset
0.746 0.721 0.707 0.664 0.630
0.454 0.416 0.391 0.324 0.291
0.820 0.811 0.805 0.783 0.793
0.589 0.594 0.593 0.569 0.523
0.312 0.326 0.328 0.281 0.134
0.235 0.234 0.228 0.165 0.064
ALMANAC-Skin Dataset
0.786 0.743 0.750 0.697 0.624
0.488 0.439 0.409 0.340 0.326
0.832 0.822 0.814 0.800 0.818
0.622 0.598 0.586 0.582 0.537
0.388 0.333 0.306 0.303 0.159
0.302 0.246 0.216 0.197 0.109
0.0
0.2
0.4
0.6
0.8
1.0
AUROC (↑)
AUCPR (↑)
ACC (↑)
BACC (↑)
F_1 (↑)
KAPPA (↑)
SynProtX-GATFP
DeepSynergy
DeepDDS
AttenSyn
XGBoost

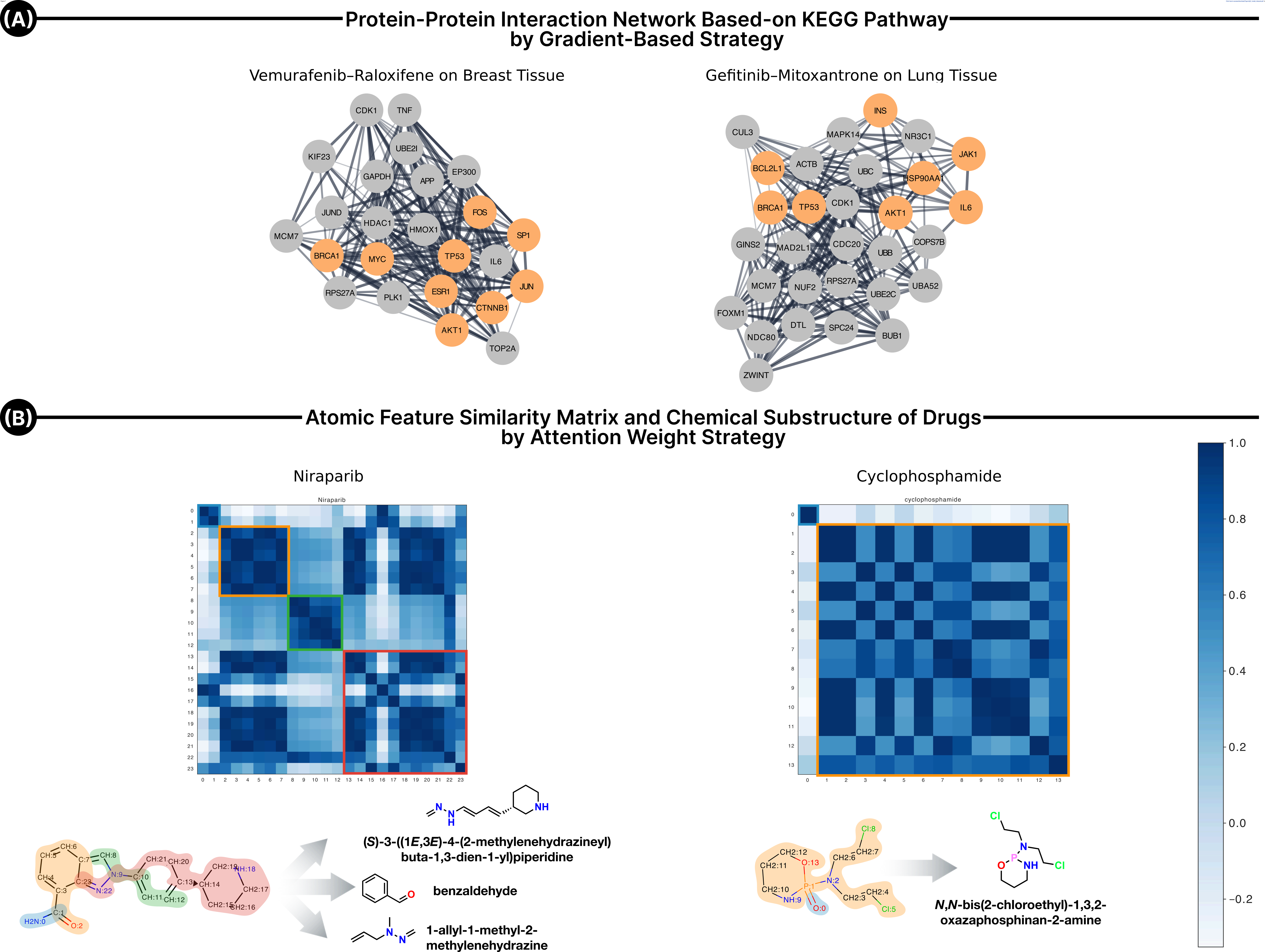

(A)
Protein-Protein Interaction Network Based-on KEGG Pathway by Gradient-Based Strategy
Vemurafenib–Raloxifene on Breast Tissue
Gefitinib–Mitoxantrone on Lung Tissue
CDK1
TNF
KIF23
UBE2I
GAPDH
APP
EP300
JUND
HDAC1
HMOX1
FOS
SP1
MCM7
BRCA1
MYC
TP53
IL6
JUN
RPS27A
PLK1
ESR1
CTNNB1
AKT1
TOP2A
INS
CUL3
MAPK14
NR3C1
JAK1
BCL2L1
ACTB
UBC
HSP90AA1
TP53
CDK1
AKT1
IL6
GINS2
MAD2L1
CDC20
UBB
COPS7B
MCM7
NUF2
RPS27A
UBE2C
UBA52
FOXM1
DTL
SPC24
NDC80
BUB1
ZWINT
(B)
Atomic Feature Similarity Matrix and Chemical Substructure of Drugs by Attention Weight Strategy
Niraparib
Cyclophosphamide
(S)-3-((1E,3E)-4-(2-methylenehydrazineyl)buta-1,3-dien-1-yl)piperidine
benzaldehyde
1-allyl-1-methyl-2-methylenehydrazine
N,N-bis(2-chloroethyl)-1,3,2-oxazaphosphinan-2-amine
1.0
0.8
0.6
0.4
0.2
0.0
−0.2

Click here to access/download
Supplementary Material
SynProtX_supp_V6.pdf

Personal Cover Click here to access/download;Personal Cover;cover_letter_ggs.pdf

School of Information Science and Technology (IST)
Vidyasirimedhi Institute of Science and Technology (VISTEC)
555 Moo 1, Payupnai, Wangchan, Rayong 21210, Thailand
Tel: +66 (0) 33-01 4444 | Fax: +66 (0) 33-01 4445
www.vistec.ac.th

December 22, 2024

To
Editor-in-Chief
GigaScience

Subject: Manuscript Submission "SynProtX: A Large-Scale Proteomics-Based Deep Learning Model for Predicting Synergistic Anticancer Drug Combinations"

Dear Editor-in-Chief,

We are pleased to submit our manuscript entitled "**SynProtX: A Large-Scale Proteomics-Based Deep Learning Model for Predicting Synergistic Anticancer Drug Combinations**" for consideration in ***GigaScience***. We affirm that this work is original, unpublished, and has not been submitted elsewhere for publication. All listed authors have made substantive contributions to this research and have given their approval for the manuscript's current version to be submitted.

Our work introduces SynProtX, an innovative deep learning framework that leverages large-scale proteomics and molecular structural data to predict synergistic anticancer drug combinations. By integrating Graph Neural Networks (GNNs) for molecular structure analysis with deep neural networks (DNNs) for gene and protein expression data, SynProtX establishes a biologically relevant and robust prediction framework. The model's performance, validated across diverse cancer datasets, demonstrates its superiority over existing methods in both classification and regression tasks.

Aligned with ***GigaScience***'s commitment to Open Science, SynProtX showcases transparency and reproducibility by openly sharing all datasets, code, and computational workflows through GitHub (https://github.com/manbaritone/SynProtX) and Zenodo (https://doi.org/10.5281/zenodo.14040426). Furthermore, the study employs state-of-the-art techniques to advance open data integration and enhance the reproducibility of results in biochemical/biomedical research.

Key highlights of our work include:

1. **Innovative Computational Approach:** Utilizing Graph Neural Networks (GNNs) for molecular structure analysis and Deep Neural Networks (DNNs) for proteomics and multi-omics data integration, SynProtX predicts drug synergy with high accuracy across diverse datasets.
2. **Integration of Proteomics and Multi-Omics Data:** Leveraging large-scale proteomics data from ProCan-DepMapSanger, SynProtX reveals insights into protein-level drug responses, providing a more functional perspective compared to transcriptomic-level analyses.
3. **Real-World Validation:** SynProtX has been rigorously tested on datasets like FRIEDMAN-Skin and ALMANAC, demonstrating robust generalizability across cancer types and enhancing insights into precision oncology and translational research.

School of Information Science and Technology (IST)
Vidyasirimedhi Institute of Science and Technology (VISTEC)
555 Moo 1, Payupnai, Wangchan, Rayong 21210, Thailand
Tel: +66 (0) 33-01 4444 | Fax: +66 (0) 33-01 4445
www.vistec.ac.th

We also wish to disclose that AI-assisted technologies, including OpenAI's **ChatGPT 4o**, were utilized during the manuscript drafting process. These tools were employed solely for linguistic refinement, improving clarity and coherence, while the scientific content and interpretation were generated by the authors. We have ensured the accuracy and originality of the work, adhering to all ethical guidelines for responsible AI use in research.

This manuscript directly aligns with ***GigaScience***'s scope, as it combines **Big Data** approaches with open, FAIR principles to address challenges in high-throughput biological research. Additionally, the manuscript to emphasize its contributions to **computational biology and bioinformatics**, including applications of deep learning, graph neural networks, in biochemical data prediction and the integration of high-throughput proteomics data to tackle pressing issues in cancer treatment. We believe that SynProtX's integration of novel computational tools with publicly available multi-omics data serves as an exemplary model for Open Science dissemination, delivering high-impact utility to the journal's readership.

We appreciate your consideration of this submission and are happy to address any comments or suggestions. We remain committed to advancing reproducibility, usability, and open collaboration in scientific research and are excited about the opportunity to contribute to ***GigaScience***.

Thank you for your time and consideration. We look forward to the opportunity to contribute to your journal.

Sincerely Yours,

Assoc. Prof. Sarana Nutanong, Ph.D.
School of Information Science and Technology, VISTEC
Email: snutanon@vistec.ac.th

Bundit Boonyarit
Ph.D. candidate
School of Information Science and Technology, VISTEC
Email: bundit.b_s18@vistec.ac.th

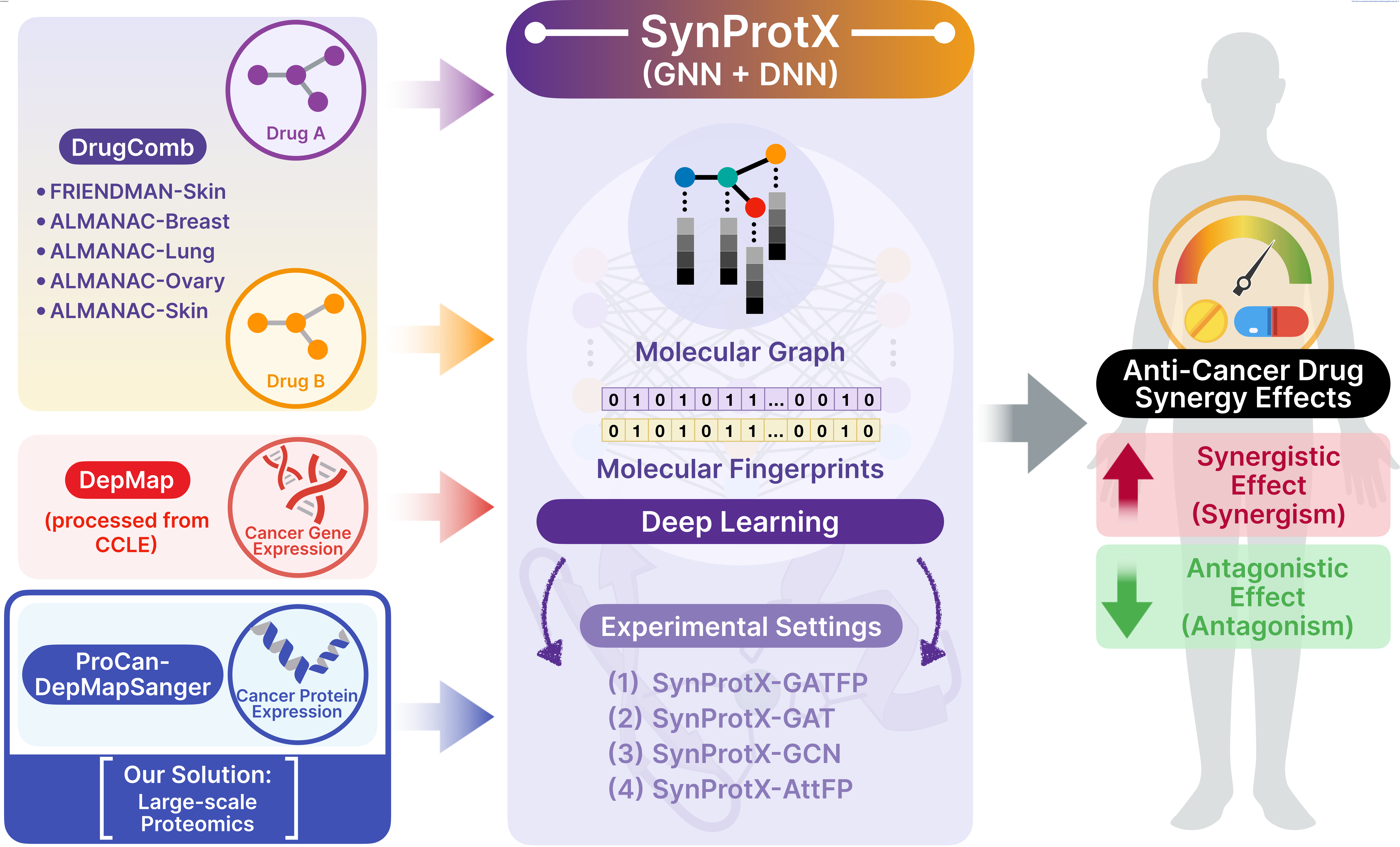
SynProtX
(GNN + DNN)
DrugComb
Drug A
Drug B
• FRIENDMAN-Skin
• ALMANAC-Breast
• ALMANAC-Lung
• ALMANAC-Ovary
• ALMANAC-Skin
DepMap
(processed from CCLE)
Cancer Gene Expression
ProCan-DepMapSanger
Cancer Protein Expression
Our Solution: Large-scale Proteomics
Molecular Graph
0 1 0 1 0 1 1 ... 0 0 1 0
0 1 0 1 0 1 1 ... 0 0 1 0
Molecular Fingerprints
Deep Learning
Experimental Settings
(1) SynProtX-GATFP
(2) SynProtX-GAT
(3) SynProtX-GCN
(4) SynProtX-AttFP
Anti-Cancer Drug Synergy Effects
Synergistic Effect (Synergism)
Antagonistic Effect (Antagonism)
