# Supplementary material for: SynProtX: a large-scale proteomics-based deep learning model for predicting synergistic anticancer drug combinations
Source: Gigascience. 2025 Aug 12;14:giaf080. doi: 10.1093/gigascience/giaf080 (PMC12343095; doi:10.1093/gigascience/giaf080)
Supplement: giaf080_GIGA-D-24-00586_Revision_1 [file giaf080_giga-d-24-00586_revision_1.pdf]

GigaScience

SynProtX: A Large-Scale Proteomics-Based Deep Learning Model for Predicting Synergistic Anticancer Drug Combinations

--Manuscript Draft--

Manuscript Number:	GIGA-D-24-00586R1
Full Title:	SynProtX: A Large-Scale Proteomics-Based Deep Learning Model for Predicting Synergistic Anticancer Drug Combinations
Article Type:	Research
Funding Information:	Vidyasirimedhi Institute of Science and Technology — Mr. Bundit Boonyarit<br>Program Management Unit for Human Resources & Institutional Development, Research and Innovation (B38G680006) — Dr. Thanyada Rungrotmongkol
Abstract:	Motivation: Drug combination therapy plays a pivotal role in addressing the molecular heterogeneity of cancer, improving treatment efficacy, minimizing resistance, and reducing toxicity. Deep learning approaches have significantly advanced drug combination discovery by addressing the limitations of conventional laboratory experiments, which are time consuming and costly. While most existing models rely on the molecular structure of drugs and gene expression data, incorporating protein-level expression provides a more accurate representation of cellular behavior and drug responses. In this study, we introduce SynProtX, an enhanced deep learning model that explicitly integrates large-scale proteomics with deep neural networks (DNNs) and the molecular structure of drugs with graph neural networks (GNNs).<br><br>Results: The SynProtX-GATFP model, which combines molecular graphs and fingerprints using a graph attention network architecture, demonstrated superior predictive performance for the FRIEDMAN-Skin dataset. Furthermore, SynProtX outperformed existing state-of-the-art models across diverse cancer tissue datasets from the ALMANAC study, including breast, lung, ovary, and skin tissues. By incorporating protein expression data, the model consistently achieved higher accuracy than gene expression-only models, reflecting the functional state of cancer cells. The generalizability of SynProtX was rigorously validated using cold-start prediction, including leave-drug-combination-out, leave-drug-out, and leave-cell-line-out validation strategies, highlighting its robust performance and potential for clinical applicability. Additionally, SynProtX identified key cancer-associated proteins and molecular substructures, offering novel insights into the biological mechanisms underlying drug synergy. These findings highlight the potential of integrating large-scale proteomics and multi-omics data to advance anticancer drug design and combination therapy strategies for personalized medicine.<br><br>Availability and implementation: https://github.com/manbaritone/SynProtX and https://doi.org/10.5281/zenodo.14040426.
Corresponding Author:	Bundit Boonyarit<br>Vidyasirimedhi Institute of Science and Technology<br>Wangchan, Rayong THAILAND
Corresponding Author Secondary Information:	
Corresponding Author's Institution:	Vidyasirimedhi Institute of Science and Technology
Corresponding Author's Secondary Institution:	
First Author:	Bundit Boonyarit
First Author Secondary Information:	
Order of Authors:	Bundit Boonyarit

Powered by Editorial Manager® and ProduXion Manager® from Aries Systems Corporation

	Matin Kositchutima
	Tisorn Na Phattalung
	Nattawin Yamprasert
	Chanitra Thuwajit
	Thanyada Rungrotmongkol
	Sarana Nutanong
**Order of Authors Secondary Information:**	
**Response to Reviewers:**	Note: The sections that have been revised are highlighted in yellow in the main manuscript.<br><br>Reviewer #1:<br><br>Synergistic drug combination prediction is important for disease treatment. However, it remains an open problem to identify the effective drug combinations in real clinical treatments. Computational models that can predict the drug combinations by integrating chemical and omic features can be helpful for the drug combination research. In this study, the authors proposed a deep learning model for drug combination prediction. Here are some comments might be helpful.<br><br>Author's response:<br>We sincerely thank Reviewer #1 for their thoughtful and constructive feedback. Your comments have been invaluable in refining the manuscript and improving the clarity of our findings. We have carefully addressed each of your concerns, and below, we provide detailed responses to your points. We believe the revisions have significantly strengthened our work, and we appreciate your contribution to enhancing the manuscript.<br><br>1. The model design innovation is limited and also comparison results showed limited improvements of the proposed method than other existing models. So it is important to clearly indicate the unique contributions of the proposed model than existing models.<br><br>Author's response:<br>We understand the concern regarding the novelty of our model design. The primary innovation in our study lies in the explicit integration of large-scale proteomics data alongside molecular graphs and molecular fingerprints. This large-scale proteomics data with the slightly modified approach has not been extensively explored in the anticancer drug combination prediction. Our model, SynProtX, incorporates multi-omics data (proteomics, transcriptomics, and drug molecular structure) to predict drug synergy. This comprehensive methodology has demonstrated improvements in predictive performance compared to existing state-of-the-art models, especially in the ablation study – w/o protein level data can significantly drop predictive performance when compared to w/o gene expression data. We have enhanced the Background section (Lines 87–159) to emphasize how current models primarily rely on genomic or transcriptomic data, which limits their ability to fully capture the functional states of cells. By explicitly integrating proteomics data, we improve the accuracy of synergy predictions by providing a more holistic view of the cellular environment. Furthermore, we stated the research question: Can large-scale proteomics data, combined with molecular graphs and fingerprints, improve model performance compared to traditional gene expression data alone? Additionally, we included the research evidence of "DeePathNet", a transformer-based deep learning model for cancer monotherapy drug response prediction. DeePathNet integrates protein expression data from the ProCan-DepMapSanger database, demonstrating the value of incorporating proteomics for improving predictive accuracy.<br><br>Additionally, we evaluated the O'Neil (ONEIL) dataset as a small-scale dataset to observe the model's ability to handle smaller, multi-tissue data scenarios. This evaluation is important for understanding how SynProtX performs on more limited datasets. We found that while SynProtX performed well across tissue-specific datasets, the ONEIL dataset revealed some interesting phenomena regarding the model's adaptability to smaller-scale data, which are discussed in the Results and Discussion

section.

Furthermore, we have added a Limitations of the Study section (Lines 759–791) that discusses the constraints of SynProtX model, such as model's ability to generalize to unseen data, particularly in cold-start scenarios, and model overfitting in ONEIL dataset in small-scaled dataset and cell lines span multiple tissue types.

In the Potential Implications section (Lines 793–820), we discuss how the incorporation of proteomics data in SynProtX has important implications for personalized medicine to other diseases, drug toxicity prediction, and cancer biomarker discovery. The ability to predict drug synergy with a more comprehensive dataset can lead to more precise treatment strategies, offering new avenues for improving therapy outcomes.

2. From a large number of features, it remains challenging to identify the important features contributing to the model prediction. Without systematic evaluation, it is not sound to conclude the importance of the identified features. So it is important to systematically evaluate the importance/score distributions of the all the features/the top-ranked features.

Author's response:
We agree that a more systematic evaluation of feature importance is necessary. To address this, we have conducted a comprehensive feature importance analysis using integrated gradients (IG) (Figures 9 and 10). This allows us to rank and evaluate the contribution of each feature (including protein expression and gene expression) to the model's predictions. The revised manuscript now includes a detailed description of these results in "Protein Level Improves Model Predictive Performance for SynProtX-GATFP" section (Lines 470–578). Here, we provide insights into how individual features influence the model's performance across different datasets. Specifically, we found that protein expression plays a significant role in improving the model's predictive accuracy.

In addition, we have included research evidence supporting the top-ranked proteins based on their involvement in biochemical pathways specific to the tissue types in the datasets. This highlights how protein-level features can contribute to understanding the mechanisms driving drug synergy.
The methodology of IG is elaborated further in the "Model Interpretability" section (Lines 1021–1073). Moreover, this section describes the attention weight strategy used to support the results presented in Figure 14. This attention mechanism enhances the interpretability of the model, allowing us to identify the most influential chemical substructure features in drug synergy predictions.

3. There are some 'block effects' of some drugs, which means that some drugs always works better than other drugs. Are these features associated with these drugs? If yes, not much meaningful or informative as it is just a simple correlation. It is important to identify the key mechanisms of cell-specific response drugs.

Author's response:
We acknowledge that the "block effects" observed in some drug combinations need to be explored in greater depth. In response, we have expanded the analysis to include pathway-enrichment analyses (GSEA) and molecular fragment analysis in the section "Integrated Gradients (IG) Method of SynProtX-GATFP Explains Block Effects" (Lines 697–758 and Supplementary Tables S17 and S18). For the methodology, we applied IG to assess the contribution of individual protein features, focusing on the most influential features identified through ablation studies, to the drug combination synergy prediction. Specifically, we focused on combinations of top-proportion drugs found in the test set, including vismodegib–mitotane via the MDA-MB-468 and MCF-7 cell lines of the ALMANAC-Breast dataset, and vandetanib–gefitinib via the NCI-H226 and A549 cell lines of the ALMANAC-Lung dataset.

The results of these analyses show that vismodegib–mitotane and vandetanib–gefitinib exhibit consistent block effects across these cell lines, suggesting that certain drugs in the combinations play dominant roles in driving synergy. The pathway-enrichment analysis further revealed that specific signaling pathways, such as EGFR and MAPK pathways, were significantly enriched in the most influential proteins. This deeper

Powered by Editorial Manager® and ProduXion Manager® from Aries Systems Corporation

mechanistic understanding goes beyond simple correlation, providing novel insights into the key biological mechanisms responsible for drug synergy.

4. It is important to evaluate the model performance on cell-specific.

Author’s response:
We have now included an evaluation of the model’s performance on cell-specific data, which was not adequately addressed in the initial version. In the revised manuscript, we have dedicated the section titled “SynProtX-GATFP Affirms the Performance on Cell-specific Responses” (Lines 406-469) to thoroughly describe the model's performance on individual cell lines within specific cancer types. The evaluation of cell-specific responses is critical for assessing how well SynProtX can predict drug synergy within different cancer cell lines, which may vary in their drug response profiles.

This evaluation is supported by Supplementary Tables S4–S15, which provide detailed performance metrics across cell lines for different datasets such as ALMANAC-Breast, ALMANAC-Lung, ALMANAC-Ovary, ALMANAC-Skin, FRIEDMAN, and ONEIL for regression and classification tasks. The results demonstrate that SynProtX is capable of making highly accurate predictions for individual cell lines, accounting for their unique molecular and proteomic profiles. The ability to evaluate cell-specific data is a major strength of our model, as it enhances the precision and relevance of predictions for personalized cancer treatments.

We sincerly thank Reviwer #1 for their invaluable suggestion to evaluate the model performance on cell-specific. We agree that this is an important aspect to consider, as different cell lines may exhibit distinct drug response profiles. By evaluating SynProtX on cell-specific data, we can better understand how the model performs across various biological contexts and ensure that it captures the unique molecular and proteomic characteristics of each cell type. This approach significantly enhances the model's ability to predict drug synergy in a more personalized manner

5. It is important to use ‘cold-start’ (remove all the information of given cells or given drugs from the training data to evaluate the model performance).

Author’s response:
To address concerns about generalizability, we have implemented cold-start evaluation methods, including leave-drug-combination-out, leave-drug-out, and leave-cell-line-out strategies, to assess the generalizability of our model on unseen data (Figures 11–13). We would like to note that the leave-drug-combination-out result was included in the original manuscript.

The results of these cold-start validations are now presented in the section “SynProtX-GATFP Demonstrates Generalization Capability on Cold-start Scenarios” (Lines 579–622), demonstrating the model’s ability to perform well even with previously unseen drug combinations and cell lines. This is discussed in the revised manuscript, where we emphasize the robustness and applicability of our model in clinical settings. However, we address the constraints of both leave-drug-out and leave-cell-line-out strategies.

Leave-Drug-Combination-Out: This strategy involves excluding drug combinations from the training data and evaluating the model’s ability to predict drug synergy for these unseen combinations. The strength of this method is that it provides a more robust evaluation of the model’s ability to generalize across different drug interactions, which is critical in drug synergy prediction. It ensures that the model can predict combinations it has never seen before, making it highly relevant for clinical scenarios where new drug combinations are constantly being explored. The strength of this method lies in its ability to simulate real-world applications where new drug combinations are tested.

Leave-Drug-Out: In this strategy, one drug is excluded from the training data, and the model’s performance is evaluated based on the prediction of drug synergy for combinations involving these unseen drugs. The constraint of this method lies in the fact that the model may have limited exposure to the drug’s specific interactions with other drugs, which may affect prediction accuracy for novel drug combinations. This strategy is essential for evaluating the model’s ability to generalize to unseen drugs.

Powered by Editorial Manager® and ProduXion Manager® from Aries Systems Corporation

Leave-Cell-Line-Out: Here, the training data is split by excluding specific cell lines, and the model is tested on its ability to predict drug synergy in these cell lines. The constraint of this method is that it may reduce the representativeness of the training data, as the model is not exposed to the specific molecular and phenotypic characteristics of the excluded cell lines during training. This may result in reduced prediction accuracy for cell lines with distinct molecular profiles, especially if these cell lines significantly differ from those in the training set.

Reviewer #2:

In this paper, the authors propose a drug combination synergy prediction model using molecular graphs, molecular fingerprints, gene expression profiles, and proteomics information. The model is evaluated on five single cancer type sub-datasets of the DrugComb dataset instead of utilizing the full dataset, which limits generalizability. The use of DNN architectures and graph neural networks are not innovative, given that similar methods (e.g. DeepDDS) have already been widely explored. Although the proposed model outperforms the compared baselines, it is unclear whether baselines are strong enough.

Due to the poor performance (low AUROC), lack of generalizability, and insufficient novelty, I do not recommend acceptance of this manuscript in its current form.

Author’s response:

We thank Reviewer #2 for their comments. Your feedback has been incredibly valuable in helping us refine the manuscript, particularly with regard to the generalizability of our approach. We have carefully considered each of your points and have made several revisions to address the concerns raised. In the following, we provide detailed responses to your comments, along with our rebuttal and clarifications. We believe these changes not only address your concerns but also contribute to enhancing the clarity and impact of our findings.

Generalizability Across Datasets:
We understand your concern regarding the generalizability of our model, especially since our initial evaluation was focused on single cancer-type datasets. To better evaluate the generalizability of SynProtX, we have employed leave-one-out validation (cold-start experiment), which includes leave-drug-combination-out, leave-drug-out, and leave-cell-line-out strategies across both tissue-specific (ALMANAC-Breast, ALMANAC-Lung, ALMANAC-Ovary, ALMANAC-Skin) and study datasets (ONEIL and FRIEDMAN). This expanded validation approach allows us to demonstrate that SynProtX generalizes effectively across different biological contexts, making it a more robust model for drug synergy prediction. This highlight is presented in the section of “SynProtX-GATFP Demonstrates Generalization Capability on Cold-start Scenarios” (Lines 579–622).

Furthermore, we need to clarify that using the full DrugComb dataset could introduce challenges that may affect model reliability due to data quality and heterogeneity. Each sub-dataset in the DrugComb collection comes from different experiments, and the dose-response matrix size (Supplementary Table S2) varies between them, which can lead to inconsistent data distribution across the full dataset. This variability is an important factor to consider when evaluating model performance. Specifically, the subsets of the DrugComb dataset represent data collected from different experimental protocols and data collection methodologies, which can lead to significant variations in data quality and completeness. These discrepancies introduce heterogeneity, making it difficult to train a single model on the entire dataset due to the inconsistency in experimental conditions. Another important issue arises from cell line redundancy within each sub-dataset. Many sub-datasets, such as those from the ALMANAC-Skin or FRIDEMAN collections, may contain repeated use of the same cell lines in multiple drug combinations. This redundancy can lead to overfitting and an unfair or biased evaluation, as the model may inadvertently learn patterns from overrepresented cell lines. In such cases, the model becomes highly specialized in recognizing these familiar patterns, which limits its ability to generalize effectively to novel cell lines or

drug combinations that were underrepresented or entirely absent in the training data.

Given these challenges, we chose to evaluate SynProtX on tissue-specific and study sub-datasets rather than the full DrugComb dataset. This allows us to focus on more homogeneous data, thereby mitigating issues arising from cell line redundancy, experimental variability, and dose-response matrix size variability. By using focused sub-datasets, we ensure that the evaluation is based on consistent and uniform data, leading to more accurate and reliable predictions.

Comparison with Existing Models & Novelty:
We appreciate your comments on the novelty of our approach, particularly given that models like DeepDDS and DeepSynergy have already been widely explored. While these models have significantly contributed to the field, we believe that our approach represents a substantial advancement due to its integration of large-scale proteomics data alongside molecular graphs and fingerprints. The hypothesis guiding this study was: “Can large-scale proteomics data, combined with molecular graphs and fingerprints, improve model performance compared to traditional gene expression data alone?” (Lines 100–125). This multi-omics approach offers a more comprehensive understanding of drug synergy by providing a holistic view of the cellular environment, which ultimately improves predictive accuracy. Furthermore, we are pleased to submit this manuscript to GigaScience, a prominent journal in the field of big data-driven life sciences applications, as we believe our work aligns with the journal’s focus on computational approaches for solving complex biological problems.

To clearly demonstrate the performance of SynProtX, we have added a more detailed comparison with existing models in Figures 3 and 4 and Supplementary Tables S2 and S3. These comparisons highlight SynProtX’s high performance in both regression and classification tasks. Additionally, the Results and Discussion sections (Lines 309–365, 406–469, and 470–578) further demonstrate the advantages of our approach in comparison to other baseline models.

We expanded the Background section (Lines 87–125) to address the limitations of current models, such as their reliance on genomic data alone, which often fails to capture the complexity of drug interactions. Our multi-omics approach, particularly the inclusion of proteomics data, overcomes these limitations, leading to a more accurate prediction of drug synergy.

Model Performance & Cell-Specific Response Evaluation:
We appreciate your comment on the AUROC score and agree that the performance across different datasets warrants further clarification. While SynProtX performs well on several tissue-specific datasets, we observed some variability in the O’Neil (ONEIL) dataset (expanded experiment in revised version), where it was marginally outperformed by XGBoost. We have now included a detailed discussion of these, where we suggest that ensemble-based methods like XGBoost may outperform individual deep learning models in certain scenarios, particularly with smaller or more heterogeneous datasets.

In addition, we expanded the experiment on cell-specific responses to provide a more thorough analysis of how SynProtX performs across different cancer cell lines. In the revised manuscript, we present a dedicated section titled “SynProtX-GATFP Affirms the Performance on Cell-Specific Responses” (Lines 406–469), where we evaluate the model’s performance on specific cancer cell lines within the ALMANAC-Breast, ALMANAC-Lung, ALMANAC-Ovary, ALMANAC-Skin, FRIEDMAN, and ONEIL datasets. The results demonstrate that SynProtX is capable of making highly accurate predictions for individual cell lines, accounting for their unique molecular and proteomic profiles. The ability to evaluate cell-specific data is a major strength of our model, as it enhances the precision and relevance of predictions for personalized cancer treatments.

We investigated block effects in drug combinations in section of “Integrated Gradients (IG) Method of SynProtX-GATFP Explains Block Effects” (Lines: 697–758). Block effects occur when one drug in a combination dominates the synergistic effect, causing the synergy to be primarily driven by this drug across multiple cell lines. To identify these block effects, we applied IG to assess the contribution of individual protein

	features, focusing on the most influential features, to the drug combination synergy prediction. The IG method is particularly useful for identifying the most influential proteins for predicting drug synergy, especially in cell-specific contexts. Our results show that SynProtX performs robustly across different cancer cell lines, accurately identifying drug synergy patterns that are unique to each cancer type. This was particularly evident in the ALMANAC-Breast and ALMANAC-Lung datasets, where we observed consistent predictive accuracy. These findings demonstrate that SynProtX is capable of capturing cell-specific drug synergy by accounting for the unique molecular and proteomic characteristics of each cell line. This ability to tailor predictions based on the specific features of individual cell lines underscores the model's potential for personalized drug combination strategies, which is one of the key strengths of SynProtX.<br><br>Furthermore, we have conducted an ablation study titled "Protein Level Improves Model Predictive Performance for SynProtX-GATFP" (Lines 470–578). This ablation study demonstrates the significant impact of protein-level data on model performance. By isolating the influence of protein-level features, we show that including proteomics data substantially improves the model's predictive accuracy compared to models relying solely on gene expression data. This study provides strong evidence supporting the importance of protein data for predicting drug synergy.
**Additional Information:**	
**Question**	**Response**
Are you submitting this manuscript to a special series or article collection?	No
**Experimental design and statistics**<br><br>Full details of the experimental design and statistical methods used should be given in the Methods section, as detailed in our Minimum Standards Reporting Checklist. Information essential to interpreting the data presented should be made available in the figure legends.<br><br>Have you included all the information requested in your manuscript?	Yes
**Resources**<br><br>A description of all resources used, including antibodies, cell lines, animals and software tools, with enough information to allow them to be uniquely identified, should be included in the Methods section. Authors are strongly encouraged to cite Research Resource Identifiers (RRIDs) for antibodies, model organisms and tools, where possible.<br><br>Have you included the information	Yes

Powered by Editorial Manager® and ProduXion Manager® from Aries Systems Corporation

requested as detailed in our Minimum Standards Reporting Checklist?	
**Availability of data and materials**<br><br>All datasets and code on which the conclusions of the paper rely must be either included in your submission or deposited in publicly available repositories (where available and ethically appropriate), referencing such data using a unique identifier in the references and in the "Availability of Data and Materials" section of your manuscript.<br><br>Have you have met the above requirement as detailed in our Minimum Standards Reporting Checklist?	Yes
GigaScience has policies and guidelines in place for the use of generative AI-writing tools such as ChatGPT. If you have used such writing tools to assist with writing the manuscript this must be declared and cited in the text. Authors should not list AI-writing tools and other AI-assisted technologies as an author or co-author and should acknowledge that they are fully responsible for text generated or refined by AI-writing tools.<p><br><br>A summary of use (particularly in the introduction or among methods) needs to be included at the end of the paper, and the outputs should also be included as a supplementary file hosted in GigaDB or other open repositories. Please read our guidelines for more information. <p><br><br>By submitting to GigaScience, you are aware of the journal's AI-writing tools policy, and if you have declared use of such tools below, you have acknowledged this where appropriate in your manuscript and have made a summary of use and outputs available. <p><br><b>AI-assisted writing tools have been used in the preparation of this manuscript?	Yes

Manuscript

Click here to access/download;Manuscript;SynProtX_main_V7.docx

SynProtX: A Large-Scale Proteomics-Based Deep Learning Model for Predicting Synergistic Anticancer Drug Combinations

Bundit Boonyarit[1], Matin Kositchutima[2], Tisorn Na Phattalung[2], Nattawin Yamprasert[3], Chanitra Thuwajit[4], Thanyada Rungrotmongkol[5,6,*], Sarana Nutanong[1,*]

[1]School of Information Science and Technology, Vidyasirimedhi Institute of Science and Technology, Rayong, 21210, Thailand

[2]Kamnoetvidya Science Academy, Rayong, 21210, Thailand

[3]School of Information, Computer, and Communication Technology, Sirindhorn International Institute of Technology, Thammasat University, Pathum Thani, 12120, Thailand

[4]Department of Immunology, Faculty of Medicine Siriraj Hospital, Mahidol University, Bangkok, 10700, Thailand

[5]Program in Bioinformatics and Computational Biology, Graduate School, Chulalongkorn University, Bangkok, 10330, Thailand

[6]Center of Excellence in Structural and Computational Biology, Department of Biochemistry, Faculty of Science, Chulalongkorn University, Bangkok, 10330, Thailand

*** Corresponding authors**

(1) Sarana Nutanong – *School of Information Science and Technology, Vidyasirimedhi Institute of Science and Technology, Rayong, 21210, Thailand; Email: snutanon@vistec.ac.th*

(2) Thanyada Rungrotmongkol – *Program in Bioinformatics and Computational Biology, Graduate School, Chulalongkorn University, Bangkok, 10330, Thailand; Center of Excellence in*

Structural and Computational Biology, Department of Biochemistry, Faculty of Science, Chulalongkorn University, Bangkok, 10330, Thailand; Email: thanyada.r@chula.ac.th

GRAPHICAL ABSTRACT

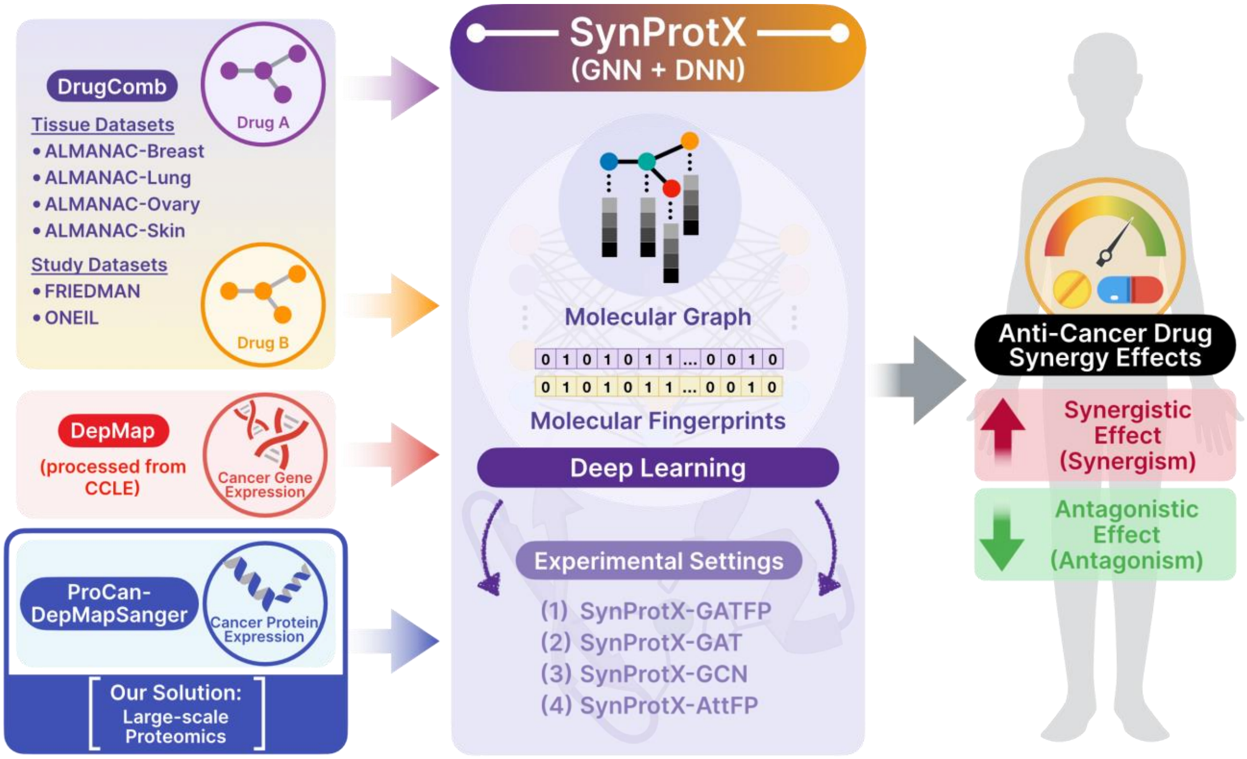

SynProtX, a deep learning model leveraging large-scale proteomics, molecular graphs, and fingerprints to enhance the prediction of the synergistic effects of anticancer drug combinations

ABSTRACT

Motivation: Drug combination therapy plays a pivotal role in addressing the molecular heterogeneity of cancer, improving treatment efficacy, minimizing resistance, and reducing toxicity. Deep learning approaches have significantly advanced drug combination discovery by addressing the limitations of conventional laboratory experiments, which are time consuming and costly. While most existing models rely on the molecular structure of drugs and gene expression data, incorporating protein-level expression provides a more accurate representation of cellular behavior and drug responses. In this study, we introduce SynProtX, an enhanced deep learning model that explicitly integrates large-scale proteomics with deep neural networks (DNNs) and the molecular structure of drugs with graph neural networks (GNNs).

Results: The SynProtX-GATFP model, which combines molecular graphs and fingerprints using a graph attention network architecture, demonstrated superior predictive performance for the FRIEDMAN-Skin dataset. Furthermore, SynProtX outperformed existing state-of-the-art models across diverse cancer tissue datasets from the ALMANAC study, including breast, lung, ovary, and skin tissues. By incorporating protein expression data, the model consistently achieved higher accuracy than gene expression-only models, reflecting the functional state of cancer cells. The generalizability of SynProtX was rigorously validated using cold-start prediction, including leave-drug-combination-out, leave-drug-out, and leave-cell-line-out validation strategies, highlighting its robust performance and potential for clinical applicability. Additionally, SynProtX identified key cancer-associated proteins and molecular substructures, offering novel insights into the biological mechanisms underlying drug synergy. These findings highlight the potential of integrating large-scale proteomics and multi-omics data to advance anticancer drug design and combination therapy strategies for personalized medicine.

Availability and implementation: https://github.com/manbaritone/SynProtX and https://doi.org/10.5281/zenodo.14040426.

***Keywords*:** cancer drug combination, deep learning, drug discovery, graph neural networks, machine learning, multi-omics, personalized medicine, proteomics, synergistic effect

BACKGROUND

Cancer treatment faces significant challenges, including tumor heterogeneity, which leads to resistance to monotherapy due to the different underlying genetic and molecular characteristics within each tumor. This resistance possesses the ability to bypass treatment through various mechanisms [1, 2]. Chemotherapy and targeted treatment approaches, although pivotal in treatment, come with limitations such as severe side effects and rapid development of drug resistance [3-5]. Single-agent treatments often fail to address the multiple pathways involved in cancer progression, leading to unsatisfactory therapeutic outcomes [6]. Combination therapies or multi-target approaches, which target multiple pathways, offer improved efficacy, reduced resistance, and minimized adverse effects, making them essential for optimizing patient outcomes [7]. Despite the benefits of combination therapies, prediction of effective drug combinations remains challenging. Traditional methods are labor-intensive, time-consuming, costly, and often require extensive *in vitro* and *in vivo* testing [8, 9]. Moreover, the complex interactions between drugs and their molecular targets complicate the prediction of synergistic effects, especially given the dynamic nature of cancer biology

Computational approaches, particularly machine learning (ML) and deep learning (DL) provide promising solutions for predicting effective drug combinations by analyzing large datasets and identifying complex patterns of multi-omics data, including genomics and transcriptomics [10]. By integrating multi-omics data, including genomics and transcriptomics, these models provide insights into complex drug-target interactions. DL algorithms, in particular, excel in predicting drug synergy by learning from vast biological data and identifying non-linear relationships, enhance drug discovery efficiency, and reduce experimental costs [11]. Developing ML and DL models for predicting drug synergy requires patient-specific cancer cell data. Each cancer cell possesses a unique microenvironment and multi-omics information, including genomics, transcriptomics, metabolomics, epigenomics, and phosphoproteomics, which are crucial for understanding cancer behavior and treatment response [12]. Important cancer cell multi-omics databases include the Cancer Cell Line Encyclopedia (CCLE), which compiles information on copy number variations, gene expression, gene mutations, and DNA methylation [13, 14]. However, the availability of multi-omics data is limited, often necessitating reliance on genomic and transcriptomic data for drug response studies [15].

Currently, existing ML and DL models can predict the synergistic effects of anticancer drug combinations. Celebi et al. [16] compared multiple ML algorithms, including extreme gradient boosting (XGBoost), random forest (RF), support vector machine (SVM), Lasso, and linear regression, to classify drug combination efficacy. DeepSynergy [11], a deep neural network model, incorporates the molecular structure of drugs and gene expression data to classify and predict synergy scores for anticancer drug combinations using the Loewe Additivity score. The model processes the drug structure and gene expression data separately for each drug. DeepDDS [17] extended this by combining graph convolutional networks (GCNs), graph attention networks (GATs), and multilayer perceptrons (MLPs) to jointly model molecular drug structures and gene expression profiles. AttenSyn [18] further improves predictions by employing attention mechanisms to identify the relevant molecular structure of drugs and gene expression features. Accurately predicting synergistic effects of anticancer drug combinations is critical for advancing personalized cancer therapy.

Recent advances in precision oncology emphasize the critical role of proteomic data in cancer biology. Protein expression profiling at the protein level captures functional cellular states not reflected at the genomic or transcriptomic levels. Large-scale datasets, such as ProCan-DepMapSanger [19], provide protein-level expression data for 949 cancer cell lines, covering 8,498 proteins, enabling comprehensive analysis of over 8,000 proteins across diverse cancer types. Advances in high-throughput proteomics, particularly mass spectrometry, allow for comprehensive protein-level analyses, which reveal phenotypic characteristics and offer a more comprehensive understanding of cancer biology and drug responses [20]. Differential protein expression can pinpoint potential drug targets, unravel mechanisms of drug resistance, and identify protein signatures predictive of therapeutic responses, supporting personalized treatment strategies [21]. Zheng et al. highlighted the superiority of proteomics in single drug response prediction tasks compared to transcriptomics using graph neural networks, emphasizing proteomics' enhanced ability to reflect the downstream functional consequences of genomic alterations, directly influencing protein functions and therapeutic responses [22]. Further advancing this approach, Cai et al. developed DeePathNet [23], a transformer-based deep learning model integrating multi-omic data with cancer-specific pathways, demonstrating that the incorporation of pathway-level insights and proteomic profiles based on ProCan-DepMapSanger significantly enhances predictive accuracy and interpretability in single drug response. DeePathNet explicitly leveraged proteomic

data to identify novel biomarkers and therapeutic targets, marking significant progress in cancer omic integration and predictive modeling. Protein expression data, thus, offers crucial insights to understand cancer drug responses, as proteins can interact with drugs and various biomolecules, reflecting the disease's pathways in living organisms [24, 25]. Despite these advancements, the explicit integration of proteomic data into ML/DL models for predicting cancer drug combinations remains relatively unexplored. This leads us to address this gap and answer an important question: Can large-scale proteomics data, combined with molecular graphs and fingerprints, improve model performance compared to traditional gene expression data alone?

Building upon these recent advances and in response to the critical gap and question, we introduce SynProtX, a computational DL framework that explicitly integrates comprehensive proteomic datasets (ProCan-DepMapSanger and CCLE) with molecular fingerprints and structural information through a graph attention network. Unlike existing computational models, SynProtX uniquely leverages proteomic profiles to advance drug combination prediction accuracy, interpretability, and clinical relevance beyond existing genomic- and transcriptomic-based models. SynProtX utilizes data from the DrugComb database [26, 27], CCLE [13, 14], and ProCan-DepMapSanger [19] to predict the synergistic effects of drug combinations. We employed graph convolutional networks (GCN) [28], graph attention networks (GAT) [29], Attentive FP [30], and graph attention networks combined with molecular fingerprint (GATFP) to compare the learning processes of the molecular structure of drugs with gene and protein expression data. Our findings demonstrate that SynProtX-GATFP, which combines molecular graphs and fingerprints through a GAT coupled with DNN, provides robust predictive performance in the FRIEDMAN-Skin study [31] and surpasses state-of-the-art and baseline models on the ALMANAC dataset across the breast, lung, ovary, and skin tissues [32]. To rigorously assess generalizability, we validated SynProtX using cold-start scenarios, including leave-drug-combination-out, leave-drug-out, and leave-cell-line-out validation strategies, highlighting its robust performance and potential for clinical applicability. Additionally, SynProtX identifies cancer-associated proteins and molecular substructures of drugs, offering insights into the biological mechanisms underlying drug synergy. By leveraging proteomics, SynProtX improves the accuracy of synergy predictions and contributes to advancing anticancer drug discovery for personalized medicine.

Our contributions are summarized as follows:

- We introduced SynProtX, a DL model for predicting anticancer drug synergy utilizing new large-scale proteomics data, which aids in discovering effective anticancer drug combinations.
- We developed SynProtX explicitly integrates comprehensive proteomic datasets from the CCLE and ProCan-DepMapSanger databases and graph neural networks for the molecular structure of drugs, thereby enhancing the accuracy of synergy predictions.
- We designed SynProtX to visualize the importance of each molecular substructure in synergistic effects by incorporating attention mechanisms within the model architecture, allowing researchers to better understand and interpret the model's predictions.
- We validated the performance of SynProtX using the cold-start prediction, including leave-drug-combination-out, leave-drug-out, and leave-cell-line-out validation strategies, to demonstrate its robustness and generalizability.

PROBLEM FORMULATION

The SynProtX includes both regression and classification models. The regression model aims to predict the synergy score of drug combinations, whereas classification model aims to classify the synergism of drug combinations. The models use the drug combinations, gene expression, and protein expression data to calculate the predicted synergy score y_c for each drug combination. The predicted score, $y_c(A, B, g, p) \in \mathbb{R}$, depends on the drug combination $< A, B >$, the gene expression level g, and the protein expression level p.

For the calculation of the synergistic score S_{Loewe}, it is obtained from drug A with concentration x_1, which has an efficacy value of y_1, and drug B with concentration x_2, which has an efficacy value of y_2. For the classification task, Loewe scores [33] that are positive ($S_{Loewe} > 0$) indicated that the drug pair is considered synergistic, whereas for negative ($S_{Loewe} < 0$) scores, the drug pair is considered antagonistic. Loewe scores can be calculated using the following formula [34]:

$$S_{Loewe} = y_c - y_1(x_1 + x_2) = y_c - y_2(x_1 + x_2) \tag{1}$$

The dataset includes drug pairs i and j that have been modified to form molecular graphs M_A and M_B, respectively. The gene expression level g and protein expression level p are transformed into the vectors v_g and v_p, respectively. The drug combination (M_A and M_B), vector of gene expression (v_g), and vector of protein expression (v_p) data are used for synergy prediction.

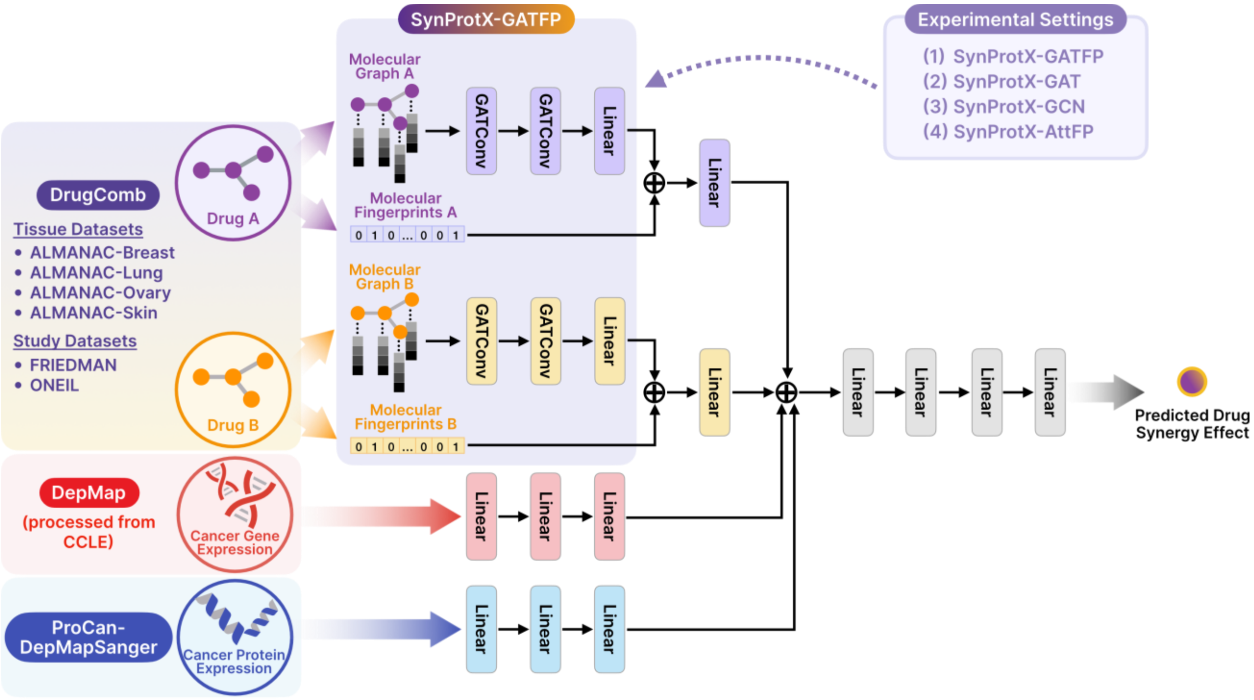

Figure 1. Overview of the SynProtX model architecture based on SynProtX-GATFP experimental setting for predicting the synergistic effects of anticancer drug combinations. The model leverages data from DrugComb, including the Tissue Datasets: (1) ALMANAC-Breast, (2) ALMANAC-Lung, (3) ALMANAC-Ovary, and (4) ALMANAC-Skin datasets and Study Datasets: (1) FRIEDMAN and (2) ONEIL. The model incorporates gene and protein expressions from DepMap (processed from CCLE) and ProCan-DepMapSanger, respectively. The model achieves deep feature extraction based on different experimental settings (SynProtX-GATFP, SynProtX-GAT, SynProtX-GCN, and SynProtX-AttFP) to enhance representation learning for the molecular structure of drug combinations.

MODEL ARCHITECTURE

The architecture of the developed predictive model, designed to explicitly integrate large-scale proteomics data for enhancing the efficacy of combination drug therapy in cancer treatment, is illustrated in **Figure 1**. Each drug combination includes two molecular graphs of individual drugs (Drug A and Drug B) that enter the first layer along with gene expression and protein expression data. Gene and protein expression data from cancer cells are learned through deep neural network layers. The resulting embedding vectors of the drug combination, gene expression, and protein expression are concatenated to form a unified feature vector. This vector is subsequently passed through additional layers of the deep neural network for prediction.

The proposed SynProtX-GATFP model integrates multiple data types, including molecular graphs, molecular fingerprints, gene expression, and protein expression data, to predict drug synergy effects. The following mathematical formulation outlines the model architecture and its components.

Input Representation

1. **Molecular Graphs**: Each drug i is represented by its molecular graph $M_i = (V_i, E_i)$, where V_i denotes the set of nodes (atoms) and E_i denotes the set of edges (bonds).

2. **Molecular Fingerprints**: Each drug i is represented by a binary vector of molecular fingerprints $F_i \in \{0, 1\}^d$, where d is the dimensionality of the fingerprint vector.

3. **Gene Expression**: The gene expression level for cancer cells is represented $by\ G_{exp} \in \mathbb{R}^n$, where n is the number of genes.

4. **Protein Expression**: The protein expression level for cancer cells is represented by $P_{exp} \in \mathbb{R}^m$, where m is the number of proteins.

Graph Neural Network (GNN)

For each drug i, the molecular graph M_i is processed using GAT to generate node embeddings. Let H_i^0 be the initial node feature matrix (generated atomic features).

$$H_i^{(l+1)} = GATLayer\left(H_i^{(l)}, E_i\right) \quad 2$$

where H_i^l represents the node features at the l-th layer and $GATLayer$ applies the graph attention mechanism to update the node embeddings. The whole architecture of the $GATLayer$ along with other used architectures is shown in the Molecular Graph and Deep Neural Networks subsection of Methods.

The final node embeddings are then pooled to produce a graph-level embedding:

$$z_i = GlobalPooling(H_i^L) \quad 3$$

Linear Transformations

The molecular fingerprint F_i, gene expression G_{exp}, and protein expression P_{exp} data are transformed through linear layers as follows:

$$f_i = W_f F_i + b_f \quad 4$$

$$g_{exp} = W_g G_{exp} + b_g \quad 5$$

$$p_{exp} = W_p P_{exp} + b_p \quad 6$$

where W_f , W_g, and W_p are weight matrices and b_f, b_g, and b_p are biased terms.

Features Fusion

For each drug combination (A, B), the embeddings from the GNN and linear transformations are concatenated and processed through additional linear layers to integrate the information:

$$h_A = Concat(z_A, f_A) \tag{7}$$

$$h_B = Concat(z_B, f_B) \tag{8}$$

Interaction Modeling

The interaction between the two drugs is modeled by combining their embeddings:

$$h_{AB} = Concat\left(h_A, h_B, g_{exp}, p_{exp}\right) \tag{9}$$

This combined representation is passed through a series of fully connected layers to predict the drug synergy effect.

$$y = \sigma\left(W_3 \left(\sigma\left(W_2 \left(\sigma(W_1 h_{AB} + b_1)\right) + b_2\right)\right) + b_3\right) \tag{10}$$

$$p = sigmoid\left(W_3 \left(\sigma\left(W_2 \left(\sigma(W_1 h_{AB} + b_1)\right) + b_2\right)\right) + b_3\right) \tag{11}$$

where W_1, W_2, and W_3 are weight matrices, b_1, b_2, and b_3 are bias terms, and σ denotes a non-linear activation function (e.g., ReLU).

Output

The final output is y, the scalar value representing the predicted synergy score of the drug combination (A, B), or p, and the predicted probability of the drug combination (A, B) being synergistic.

RESULTS AND DISCUSSION

Protein and Gene Expressions Assisted SynProtX Development

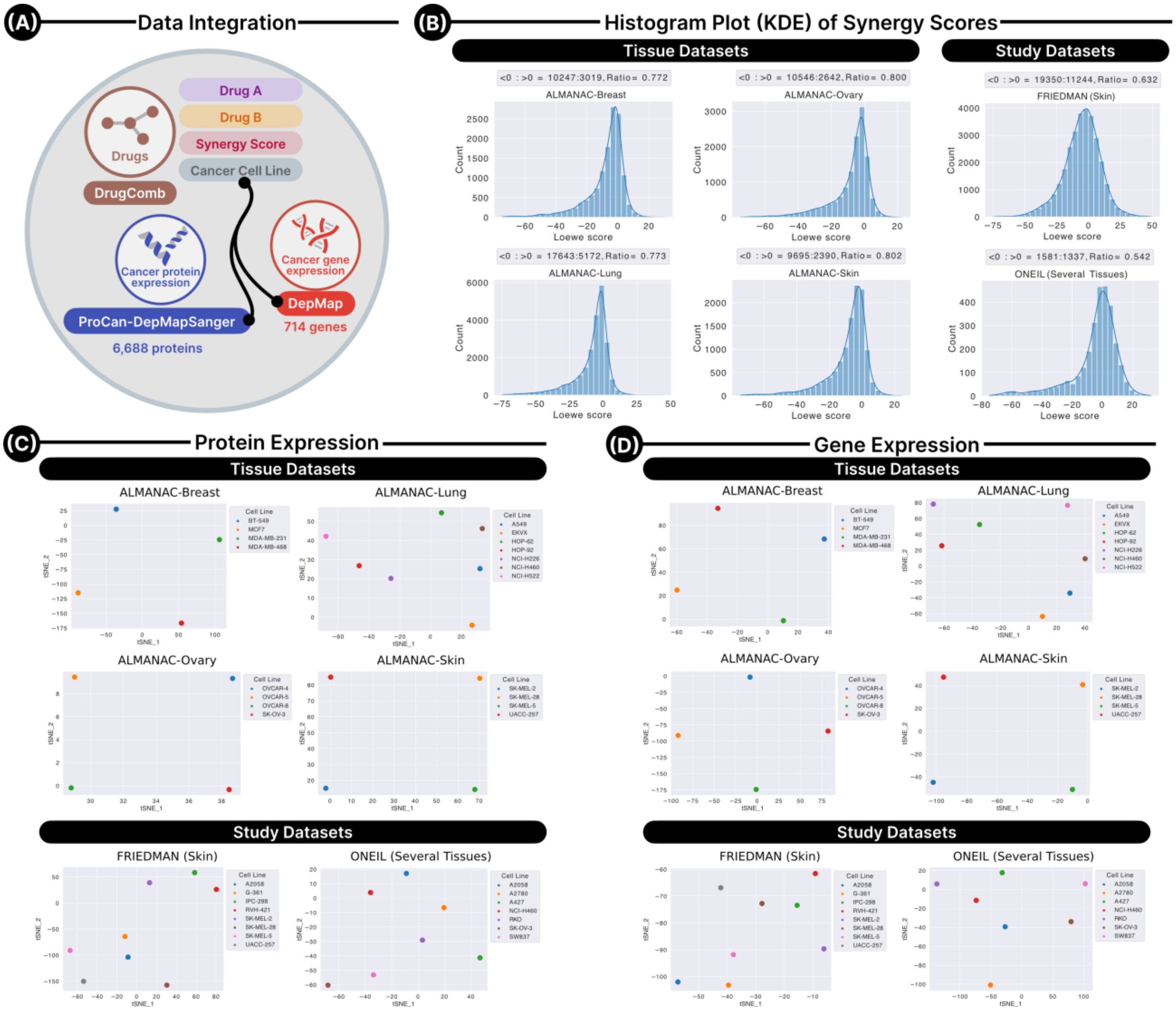

Figure 2. The data integration process and dataset analysis. **(A)** The process begins with the DrugComb database, which provides drug combination data comprising Drug A, Drug B, the Synergy Score (Loewe score), and the corresponding cancer cell line. These combinations are integrated with 6,688 protein expression levels from ProCan-DepMapSanger dataset and 714 gene expression levels from the DepMap database (processed from the CCLE database). **(B)** Distribution of Loewe synergy scores following data preprocessing across the Tissue Datasets: (1) ALMANAC-Breast, (2) ALMANAC-Lung, (3) ALMANAC-Ovary, and (4) ALMANAC-Skin; and the Study Datasets: (1) FRIEDMAN and (2) ONEIL. **(C)** The t-distributed stochastic neighbor embedding (t-SNE) visualization of cell line embeddings based on protein

expression across the across the aforementioned Tissue and Study Datasets. **(D)** The t-SNE visualization of cell line embeddings based on gene expression across the same datasets.

Integrating protein and gene expression data is crucial for enhancing the predictive performance of SynProtX. The process commenced with the extraction of drug combination data from the DrugComb database, encompassing datasets such as ALMANAC-Breast, ALMANAC-Lung, ALMANAC-Ovary, ALMANAC-Skin, FRIEDMAN, and ONEIL (**Figure 2A**). Each entry consisted of a drug pair (Drug A and Drug B), a corresponding synergy score (Loewe score), and the associated cancer cell line. To further enrich this data, protein expression data from the ProCan-DepMapSanger database and gene expression data from the DepMap database, processed from the Cancer Cell Line Encyclopedia (CCLE), were incorporated. After filtering and ensuring consistency, a total of 94,862 drug combination experiments were identified, as detailed in **Supplementary Table S1**. This integrated dataset offered a comprehensive and robust foundation for training and evaluating the model.

Notably, each drug pair was not uniformly tested across all cancer cell types, reflecting the inherent variability and context-specific nature of the data. It is also important to distinguish that while the ALMANAC-Breast, ALMANAC-Lung, ALMANAC-Ovary, ALMANAC-Skin, and FRIEDMAN datasets are derived from single tissue types, the ONEIL dataset spans multiple tissues, including skin, lung, ovary, and large intestine. This diversity in tissue types mitigates potential issues arising from cell line redundancy, which could otherwise introduce bias in model predictions. By generating sub-datasets with varying tissue types and studies, we ensure a fair evaluation of the model's performance, as redundancy could lead to overfitting and inaccurate generalization.

The integration of multimodal data, including protein and gene expression data, enabled SynProtX to capture the complex molecular signatures underlying drug responses across diverse cancer cell lines. The synergy scores from DrugComb served as target variables, indicating the efficacy of each drug combination. Protein expression data provide insights into the proteomic landscape, while gene expression data offered a view of transcriptomic variations across cancer cell lines [35-

38]. Together, these layers of biological information allowed the model to infer cellular functional states and regulatory mechanisms critical for accurate prediction of drug synergy.

The distribution of synergy scores (Loewe scores) across the ALMANAC-Breast, ALMANAC-Lung, ALMANAC-Ovary, ALMANAC-Skin, FRIEDMAN, and ONEIL datasets was analyzed after data preprocessing (**Figure 2B**). The varying distributions and skewness of these Loewe scores across different datasets provide initial insights into how drug combinations interact within various cancer cell lines. This variability underscores the complexity of predicting drug synergy and highlights the importance of using advanced models to capture these effects accurately. To further explore the underlying structure of the integrated data, t-distributed stochastic neighbor embedding (t-SNE) [39] visualizations were employed. The t-SNE analysis of cell line embeddings based on protein level (**Figure 2C**) and gene expression (**Figure 2D**) revealed distinct clustering patterns, reflecting the unique molecular characteristics of each cancer cell line. These clusters suggest that the model can effectively distinguish between different cancer cell lines and tissue types, which is essential for generalizing across various biological settings and making accurate drug synergy predictions. Separating clusters in the t-SNE plots indicates that the model can leverage these data to identify unique expression profiles that correlate with synergistic drug effects. The integration of protein and gene expression data allows the SynProtX model to account for the cellular environment and response to external stimuli and the transcriptional landscape within the cancer cells. By incorporating these data types, SynProtX can be used to predict how different drug combinations interact within specific cellular contexts, offering a more comprehensive and accurate tool for identifying synergistic drug combinations. This multi-omics approach enhances the ability of model to capture the intricate interplay between proteomic and transcriptomic factors that influence drug response [40, 41], ultimately leading to more reliable predictions and insights into the mechanisms of drug synergy.

SynProtX Elevates Model's Predictive Performance on Regression and Classification Tasks

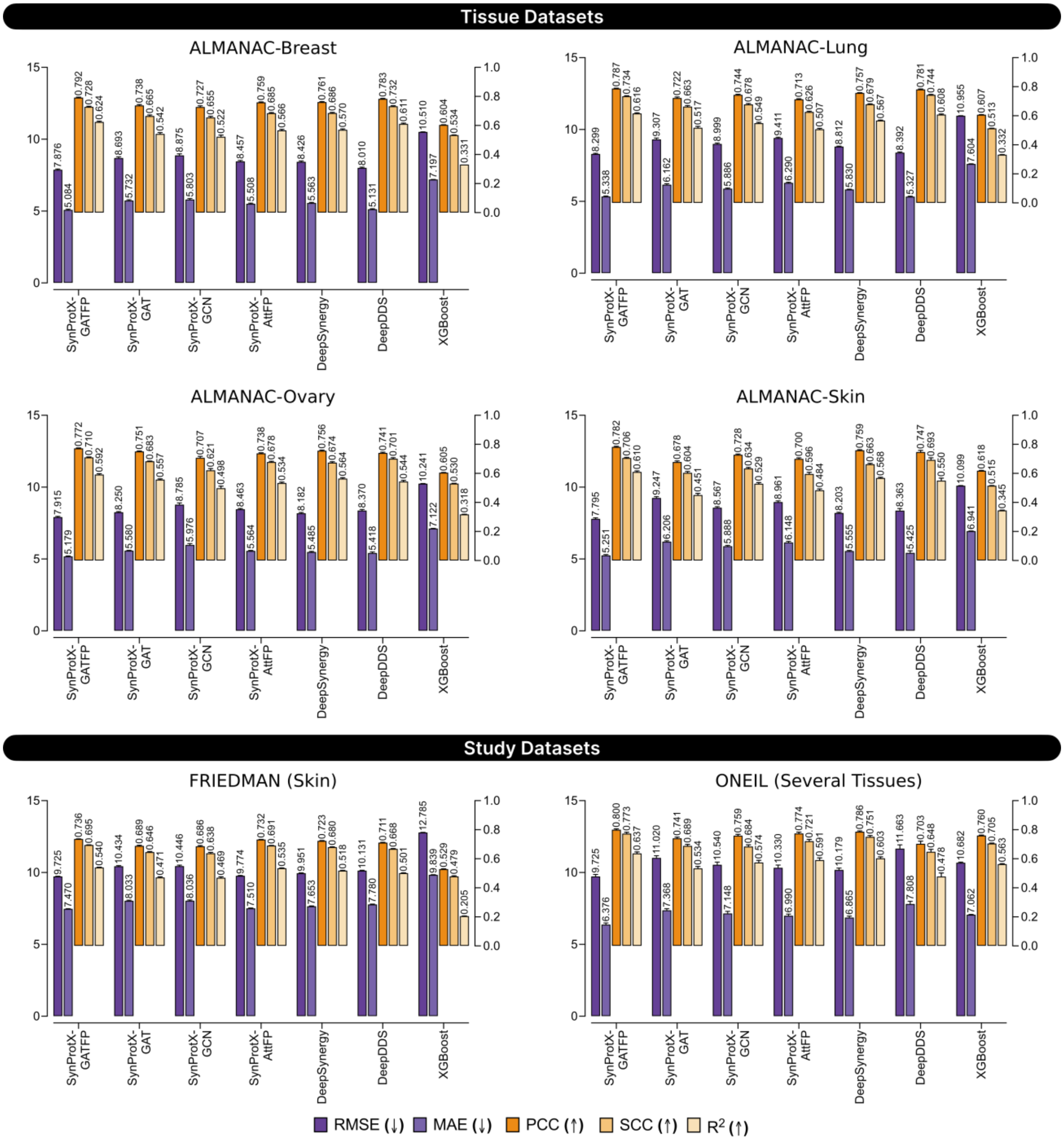

Figure 3. The predictive performance on regression tasks across the Tissue Datasets: (1) ALMANAC-Breast, (2) ALMANAC-Lung, (3) ALMANAC-Ovary, and (4) ALMANAC-Skin; and the Study Datasets: (1) FRIEDMAN and (2) ONEIL with different training models.

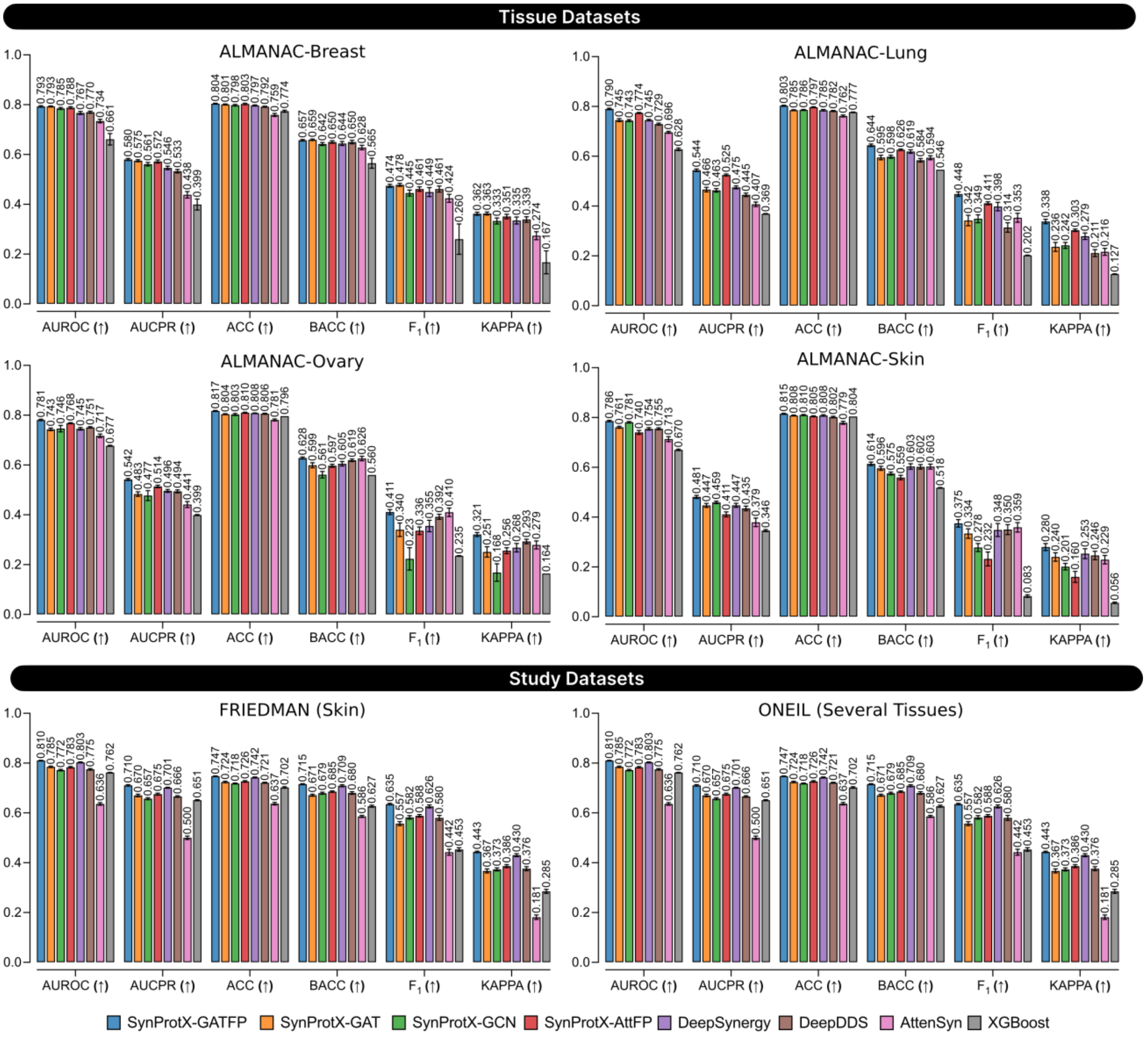

Figure 4. The predictive performance on classification tasks across the Tissue Datasets: (1) ALMANAC-Breast, (2) ALMANAC-Lung, (3) ALMANAC-Ovary, and (4) ALMANAC-Skin; and the Study Datasets: (1) FRIEDMAN and (2) ONEIL with different training models.

The SynProtX-GATFP model consistently demonstrated improvements in predictive performance across both regression and classification tasks for tissue and study datasets. In the regression task, SynProtX-GATFP achieved the lowest RMSE and MAE, along with the highest Pearson correlation coefficient (PCC) and R^2 scores across all ALMANAC tissue datasets (**Figure 3 and Supplementary Table S2 and Figure S1**). Specifically, it achieved RMSE values of 7.876, 8.299,

7.915, and 7.795 for the Breast, Lung, Ovary, and Skin datasets, respectively, along with corresponding PCC values ranging from 0.772 to 0.792. These results underscore the model's capability to accurately capture synergy patterns in tissue-specific contexts. Compared to other SynProtX variants (GAT, GCN, AttFP) and traditional baselines such as DeepSynergy, DeepDDS, and XGBoost, SynProtX-GATFP consistently ranked highest across nearly all regression metrics. This trend continued in the study datasets, with SynProtX-GATFP achieving the best regression performance in both FRIEDMAN (RMSE: 9.725 and PCC: 0.736) and ONEIL (RMSE: 9.725 and PCC: 0.800) datasets.

In classification tasks, SynProtX-GATFP also outperformed most models across all individual tissue-specific datasets (**Figure 4 and Supplementary Table S3 and Figures S2 and S3**). It attained the highest AUROC in ALMANAC-Breast (0.793), Lung (0.790), Ovary (0.781), and Skin (0.786), along with top AUCPR, F_1, and Kappa scores, highlighting its effectiveness in both balanced and imbalanced settings. In the FRIEDMAN dataset, it again led across all metrics (AUROC: 0.810, AUCPR: 0.710, F_1: 0.635, Kappa: 0.443), confirming its consistent classification capability. However, in the ONEIL dataset, although SynProtX-GATFP achieved strong classification performance (AUROC: 0.831, AUCPR: 0.777, F_1: 0.710, Kappa: 0.482), it was marginally outperformed by XGBoost, which recorded the highest AUROC (0.842) and AUCPR (0.801). This exception suggests that while SynProtX-GATFP is highly generalizable, classical ensemble-based methods like XGBoost may still retain an edge in certain classification scenarios with smaller, multi-tissue datasets.

The PCA analysis provided further insights into predictive performance of SynProtX by visualizing the feature space distribution of drug combinations for each cell line before and after training (**Figure 5**). The PCA plots for the regression task highlighted capacity of SynProtX to closely align the predicted synergy scores with the ground truth. The plots show improved feature space alignment after training, indicating that the model successfully learned the complex relationships between the input features and synergy scores (**Figure 5A**). For the classification task, PCA visualizations showed that the SynProtX-GATFP model effectively separated different classes, demonstrating its ability to accurately capture the underlying structure of the data. This effective separation suggests that SynProtX can identify and utilize the most relevant features to distinguish between synergistic and non-synergistic drug combinations (**Figure 5B**). This

alignment is crucial to contribute reliable predictions across diverse biological settings and underscores capability of SynProtX to generalize effectively across different datasets. In summary, SynProtX significantly improves the predictive performance of classification and regression tasks across various datasets, consistently outperforming other state-of-the-art models. These improvements are particularly evident in the enhanced accuracy and robustness of predictions supported by PCA analysis, which highlights the ability of the model to effectively capture and utilize complex biological data.

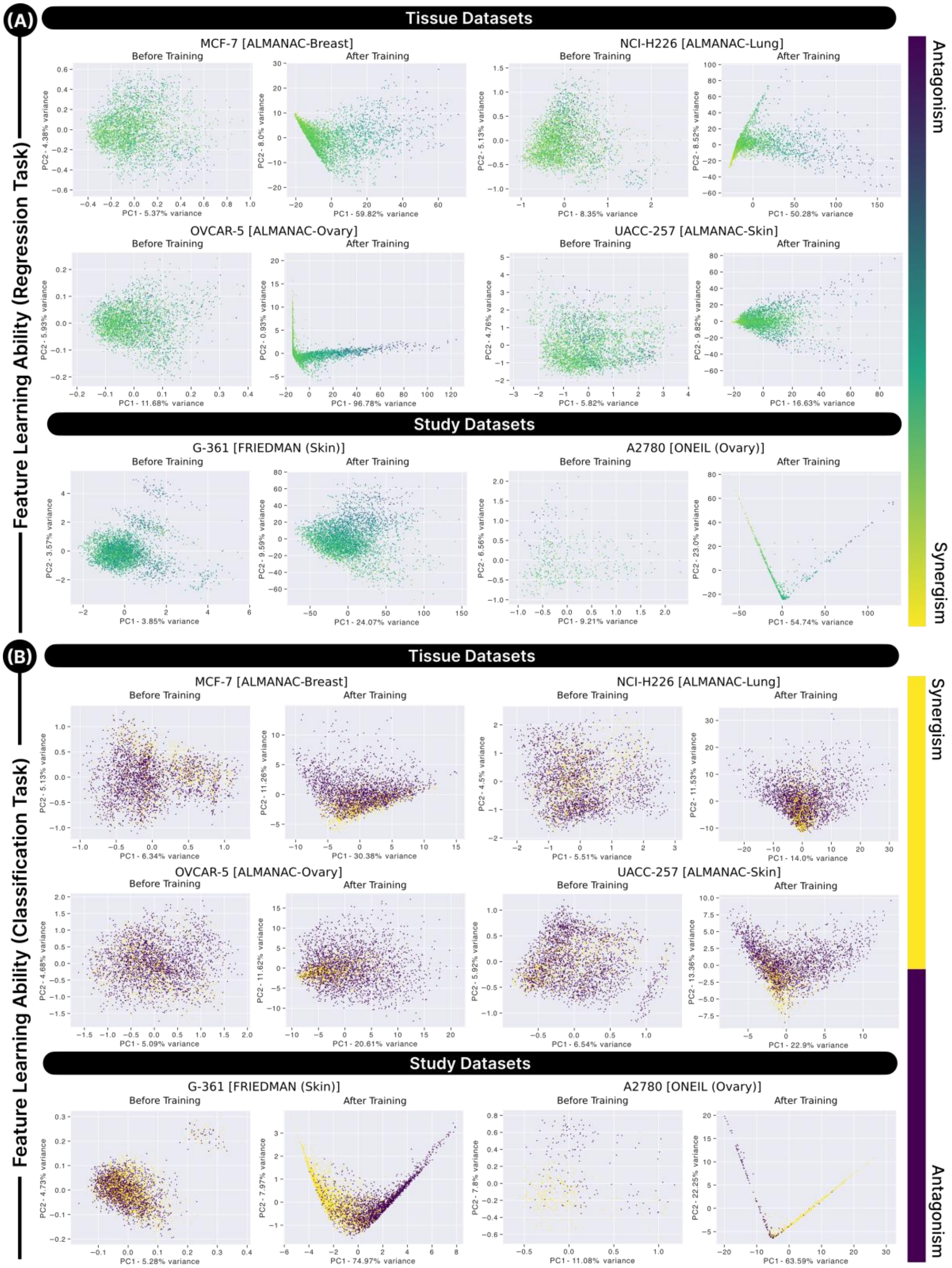

Figure 5. Principal Component Analysis (PCA) visualization of the feature space distribution of drug combinations on specific cell line for the SynProtX-GATFP model in **(A)** the regression task and **(B)** the classification task, shown before and after the training process.

Molecular Fingerprints Enhance Feature Representation and Model Generalization

The incorporation of molecular fingerprints into the SynProtX model significantly enhances its feature representation and generalization capabilities, thereby improving the predictive performance across various datasets. Integrating molecular fingerprints into SynProtX-GATFP resulted in marked improvements in the predictive performance over SynProtX-GAT across all datasets. In regression tasks, SynProtX-GATFP also demonstrated superior performance, achieving lower RMSE and MAE values while obtaining higher PCC, SCC, and R^2 scores than SynProtX-GAT. These improvements were statistically significant, with PCC increasing by up to 10.4% and R^2 increasing by up to 15.9% (**Figure 3 and Supplementary Table S2**). In the classification tasks, SynProtX-GATFP consistently outperformed SynProtX-GAT, achieving higher AUROC, AUCPR, accuracy, balanced accuracy, F_1, and Cohen's Kappa values. For example, in the FRIEDMAN-Skin dataset, SynProtX-GATFP showed a 2.50% improvement in AUROC and a 2.30% enhancement in accuracy compared to SynProtX-GAT (**Figure 4 and Supplementary Table S3**). Similar trends were observed across the ALMANAC-Breast, ALMANAC-Lung, ALMANAC-Ovary, and ALMANAC-Skin datasets, in which SynProtX-GATFP exhibited improvements across various metrics. These results underscore the significant impact of molecular fingerprints on the ability of the model to accurately predict drug synergy effects, reflecting the added value of this feature in capturing the chemical characteristics of drug combinations. Moreover, these improvements highlight the model's enhanced ability to predict synergy scores with greater accuracy and reliability, thereby validating the effectiveness of molecular fingerprints in improving the regression task performance.

The comparison of the distribution of the ground truth and predicted synergy scores further supports the enhanced feature representation and generalization achieved by SynProtX-GATFP. The violin plots in **Supplementary Figure S4** illustrate how SynProtX-GATFP's predictions align more closely with the ground truth distributions across all datasets than SynProtX-GAT. In the ALMANAC-Breast dataset, for example, SynProtX-GATFP displayed narrower and more centered distributions around the ground truth, indicating higher consistency and reduced prediction variance. This alignment is crucial to ensure that the model generalizes well across different datasets and accurately captures the underlying biological mechanisms that drive drug synergy. These results suggest that the molecular fingerprints allowed the model to better capture

the underlying relationships between the chemical structures of drug and their synergistic effects, leading to more precise predictions across different cancer types. The consistent performance improvements across both tasks highlight the importance of molecular fingerprints in enhancing the feature representation within the SynProtX-GATFP model, thereby improving its ability to generalize across various datasets. This enhanced generalization is critical for drug discovery, in which accurate predictions across different biological contexts and chemical spaces are essential. Overall, the inclusion of molecular fingerprints significantly strengthened the capabilities of SynProtX-GATFP, making it a more robust and reliable tool for predicting drug synergy in diverse cancer datasets.

SynProtX-GATFP Affirms the Performance on Cell-specific Responses

The SynProtX-GATFP model demonstrated strong performance across both regression and classification tasks at the individual cancer cell line level, reinforcing its robustness in capturing fine-grained multi-omics and drug-related features. For regression task, SynProtX-GATFP achieved the lowest RMSE and MAE, along with the highest PCC, across the vast majority of cell lines in both tissue and study datasets (**Figure 6 and Supplementary Tables S4–S9**). In the ALMANAC-Breast dataset, SynProtX-GATFP outperformed competing models across all three cell lines—BT-549, MDA-MB-231, and MDA-MB-468—with RMSEs ranging from 7.231 to 8.474 and PCCs from 0.758 to 0.822, notably higher than those of DeepSynergy, DeepDDS, and XGBoost. Similar trends were observed in ALMANAC-Lung, where SynProtX-GATFP led in five out of seven cell lines, achieving the highest PCC and lowest RMSE including A549, EKVX, HOP-62, HOP-92, and NCI-H226. The model also delivered consistently accurate predictions across all cell lines in the ALMANAC-Ovary and ALMANAC-Skin datasets. In the FRIEDMAN dataset, which includes eight skin cancer cell lines, SynProtX-GATFP led across nearly all regression metrics. For instance, in the IPC-298 cell line, it achieved a PCC of 0.817, outperforming DeepSynergy (0.801) and XGBoost (0.666), while also attaining the lowest RMSE (7.563) and MAE (5.949). Even in the heterogeneous ONEIL dataset—which spans cell lines from multiple tissue origins such as lung, ovary, skin, and colon—SynProtX-GATFP maintained top-

tier performance in most cases, including A427 (PCC: 0.874) and SK-OV-3 (PCC: 0.839), further demonstrating its cross-tissue generalizability.

Figure 7 presents violin plots of predicted synergy scores across individual cell lines in both tissue and study datasets. SynProtX-GATFP showed tight prediction distributions with centered medians across nearly all cell lines, especially in ALMANAC-Breast and ALMANAC-Lung, indicating a strong alignment between predicted and true synergy values. Similar consistency was observed in ALMANAC-Ovary, ALMANAC-Skin, and FRIEDMAN datasets, reflecting the model's ability to capture synergy patterns in diverse biological settings. Even within the more complex ONEIL dataset, the model preserved stable predictive behavior across multiple tissue types. The density and shape of the violin plots further suggest that SynProtX-GATFP effectively learned nuanced, cell-specific patterns rather than overfitting to global trends. These results collectively support the model's capacity to generalize across both homogeneous and heterogeneous cellular contexts.

In the classification task, SynProtX-GATFP also maintained high performance across key evaluation metrics, including AUROC, AUCPR, F_1, precision, and recall, across most cell lines (**Figure 8 and Supplementary Tables S10–S15**). In the ALMANAC-Breast dataset, it achieved AUROC values exceeding 0.8 for BT-549 and MDA-MB-468, outperforming classical machine learning baselines such as XGBoost, as well as several state-of-the-art (SOTA) deep learning models. The model also recorded top AUROC values in lung, ovary, and skin cell lines, including challenging examples such as SK-MEL-5. Within the more heterogeneous FRIEDMAN and ONEIL datasets, SynProtX-GATFP remained highly competitive, though it was marginally outperformed by XGBoost in select cases, suggesting the potential benefit of ensemble-based approaches. Overall, these findings underscore that the integration of proteomic and transcriptomic data enhances the model's ability to capture functional heterogeneity in drug responses at the cellular level. The cell-specific insights derived from SynProtX-GATFP reinforce its promise for personalized drug combination therapy and precision oncology applications.

Despite its strong overall performance, SynProtX-GATFP exhibited variability across certain cell lines, particularly within the diverse ONEIL dataset. For instance, while it performed well in A427 and SK-OV-3, the model's predictions were less centered and more dispersed in challenging cases such as RKO and SW837 (**Figure 7**), indicating potential difficulties in capturing synergy behavior

in cell lines with limited training data or greater biological complexity. These observations highlight areas for future improvement, such as enhancing data diversity, incorporating dynamic proteomic responses, and refining strategies for underrepresented cell types.

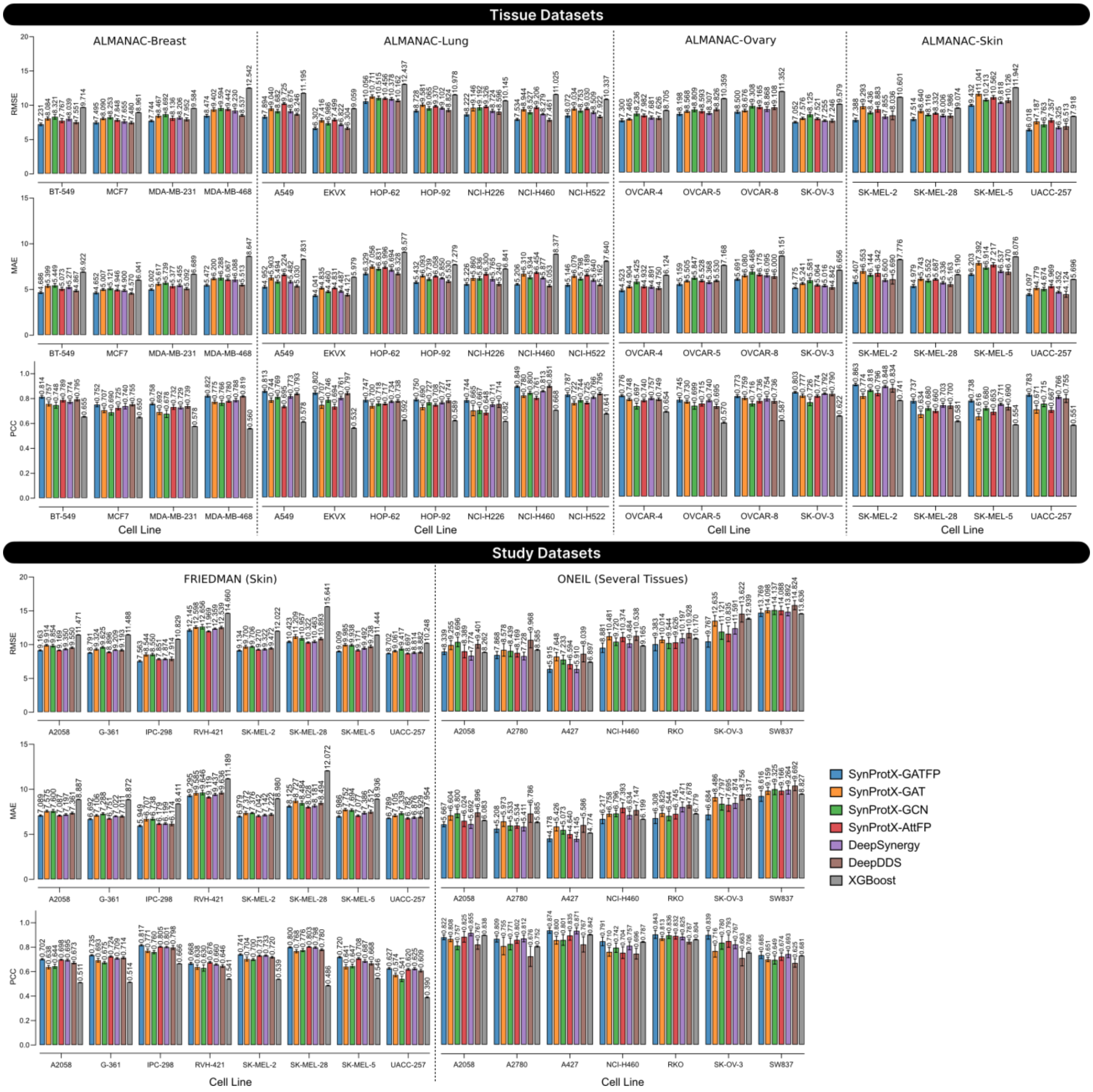

Figure 6. The predictive cell-line-specific performance on regression tasks across the Tissue Datasets: (1) ALMANAC-Breast, (2) ALMANAC-Lung, (3) ALMANAC-Ovary, and (4) ALMANAC-Skin; and the Study Datasets: (1) FRIEDMAN and (2) ONEIL with different training models.

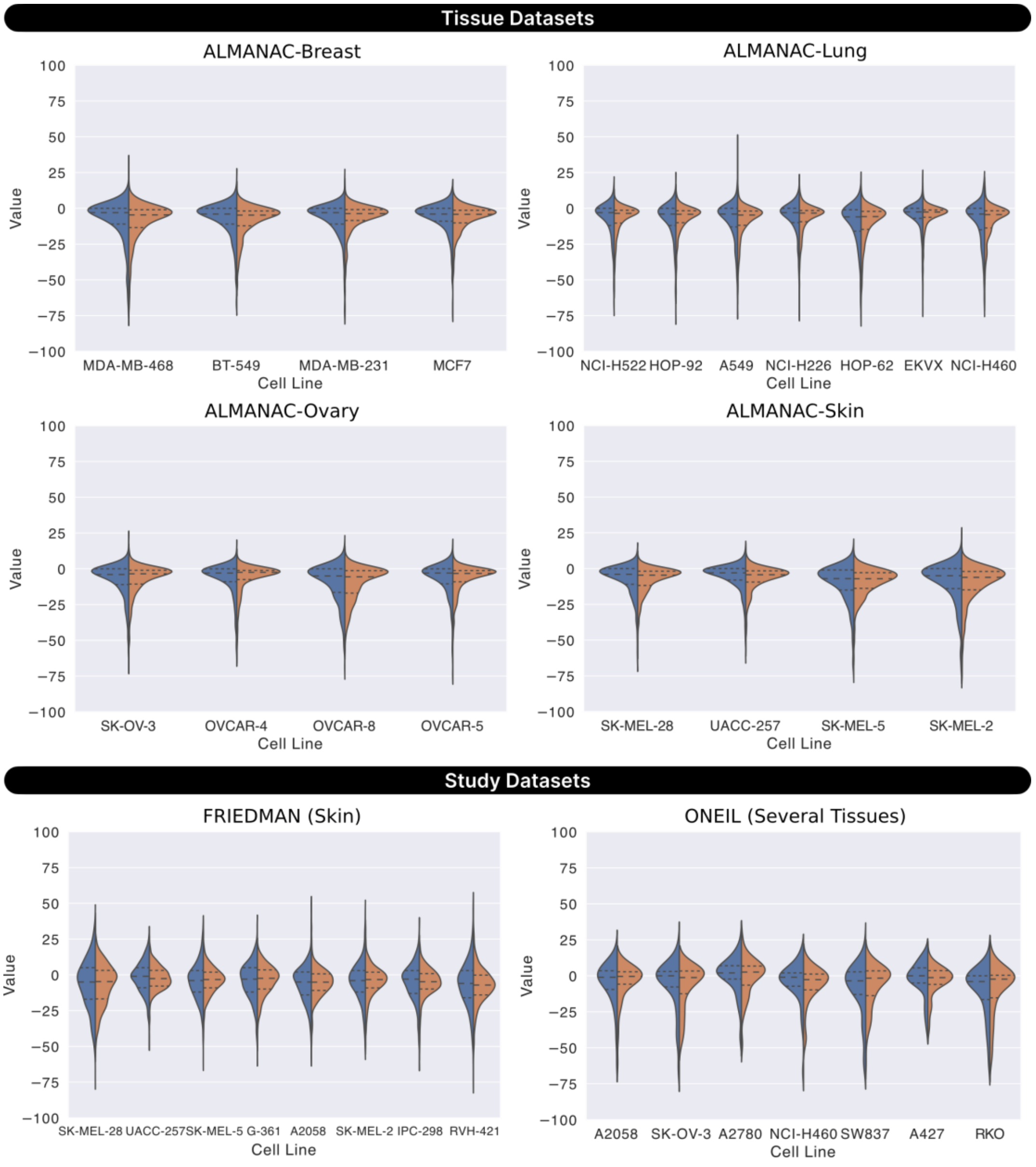

Figure 7. Comparison of distribution of ground truth and prediction synergy scores on test set across cell lines based on SynProtX-GATFP for the Tissue Datasets: (1) ALMANAC-Breast, (2) ALMANAC-Lung, (3) ALMANAC-Ovary, and (4) ALMANAC-Skin; and the Study Datasets: (1) FRIEDMAN and (2) ONEIL.

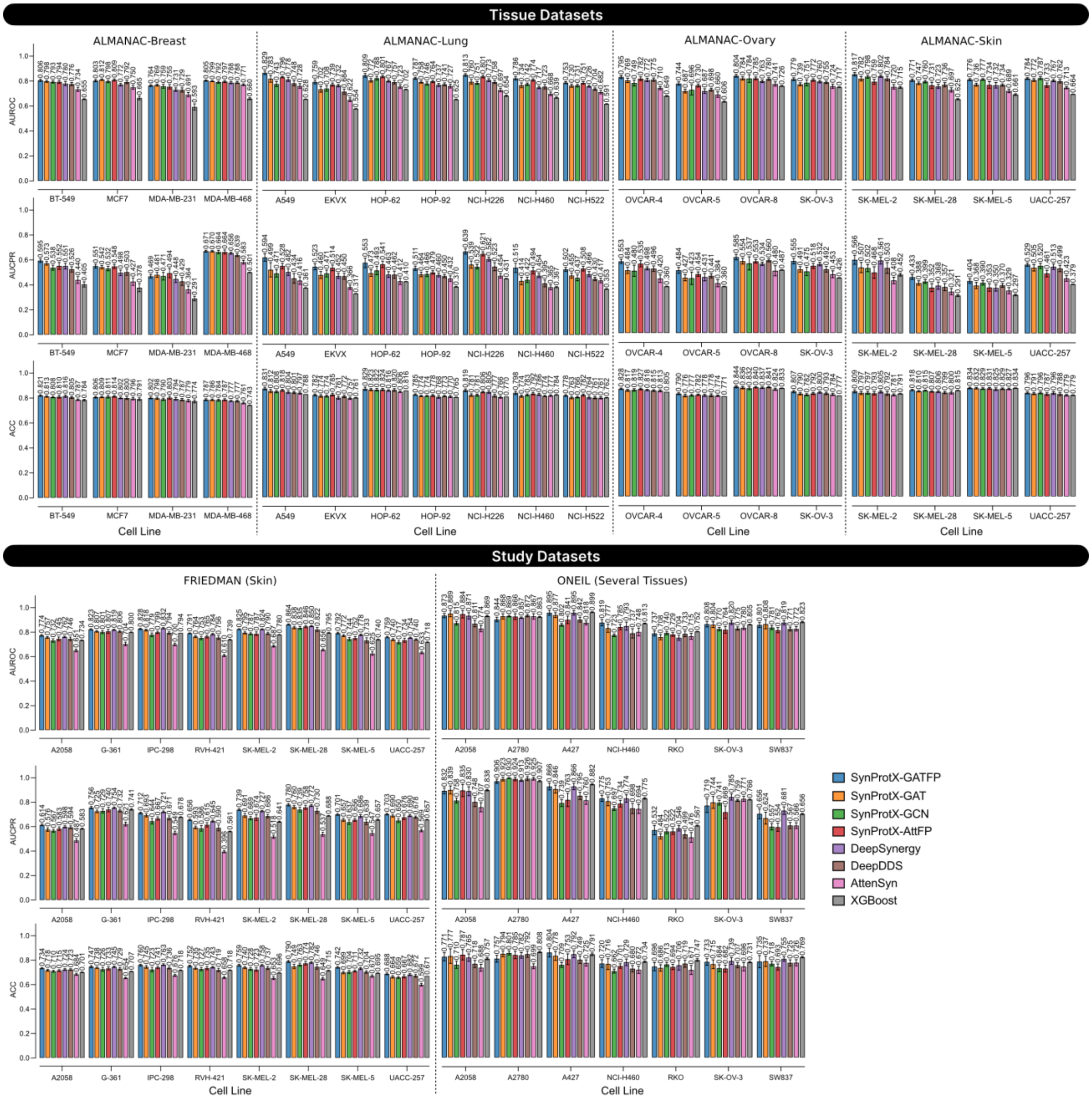

Figure 8. The predictive cell-line-specific performance on classification tasks across the Tissue Datasets: (1) ALMANAC-Breast, (2) ALMANAC-Lung, (3) ALMANAC-Ovary, and (4) ALMANAC-Skin; and the Study Datasets: (1) FRIEDMAN and (2) ONEIL with different training models.

Protein Level Improves Model Predictive Performance for SynProtX-GATFP

Table 1 The predictive performance on regression task of ablation studies between protein level and gene expression across the Tissue Datasets: (1) ALMANAC-Breast, (2) ALMANAC-Lung, (3) ALMANAC-Ovary, and (4) ALMANAC-Skin; and the Study Datasets: (1) FRIEDMAN and (2) ONEIL.

**Dataset**	**Ablation Study**	**Regression Metrics**				
		**RMSE (↓)**	**MAE (↓)**	**PCC (↑)**	**SCC (↑)**	**R^2 (↑)**
**Tissue Datasets**						
ALMANAC-Breast	w/o Prot Level	8.064±0.068	5.182±0.035	0.781±0.004	0.719±0.003	0.606±0.007
	w/o Gene Exp	7.960±0.083	5.140±0.067	0.786±0.005	0.720±0.006	0.616±0.008
ALMANAC-Lung	w/o Prot Level	8.735±0.076	5.624±0.045	0.761±0.005	0.709±0.004	0.575±0.007
	w/o Gene Exp	8.428±0.055	5.444±0.032	0.781±0.003	0.726±0.005	0.604±0.005
ALMANAC-Ovary	w/o Prot Level	8.023±0.042	5.203±0.026	0.766±0.003	0.709±0.003	0.581±0.004
	w/o Gene Exp	7.820±0.082	5.096±0.059	0.778±0.005	0.719±0.006	0.602±0.008
ALMANAC-Skin	w/o Prot Level	8.095±0.100	5.333±0.083	0.763±0.007	0.694±0.008	0.579±0.010
	w/o Gene Exp	7.740±0.072	5.236±0.038	0.785±0.005	0.707±0.004	0.615±0.007
**Study Datasets**						
FRIEDMAN (Skin)	w/o Prot Level	10.239±0.082	7.888±0.065	0.701±0.006	0.656±0.006	0.490±0.008
	w/o Gene Exp	9.717±0.020	7.438±0.016	0.737±0.001	0.695±0.002	0.541±0.002
ONEIL (Several Tissues)	w/o Prot Level	11.084±0.114	7.468±0.074	0.728±0.007	0.672±0.007	0.529±0.010
	w/o Gene Exp	9.734±0.138	6.429±0.098	0.801±0.006	0.769±0.008	0.637±0.010

Note: w/o Prot Level denotes exclusion of the protein level data and w/o Gene Exp denotes exclusion of the gene expression data.

Table 2 The predictive performance on classification task of ablation studies between protein level and gene expression across the Tissue Datasets: (1) ALMANAC-Breast, (2) ALMANAC-Lung, (3) ALMANAC-Ovary, and (4) ALMANAC-Skin; and the Study Datasets: (1) FRIEDMAN and (2) ONEIL.

**Dataset**	**Ablation Study**	**Classification Metrics**					
		**AUROC (↑)**	**AUCPR (↑)**	**ACC (↑)**	**BACC (↑)**	**F_1 (↑)**	**KAPPA (↑)**
**Tissue Datasets**							
ALMANAC-Breast	w/o Prot Level	0.779±0.003	0.525±0.007	0.791±0.001	0.614±0.003	0.385±0.007	0.279±0.006
	w/o Gene Exp	0.791±0.003	0.573±0.004	0.801±0.002	0.658±0.005	0.476±0.010	0.361±0.009
ALMANAC-Lung	w/o Prot Level	0.768±0.004	0.509±0.008	0.792±0.002	0.631±0.009	0.422±0.019	0.306±0.016
	w/o Gene Exp	0.794±0.002	0.555±0.004	0.805±0.002	0.650±0.004	0.460±0.008	0.351±0.007

Dataset	Ablation Study	Classification Metrics					
		**AUROC (↑)**	**AUCPR (↑)**	**ACC (↑)**	**BACC (↑)**	**F_1 (↑)**	**KAPPA (↑)**
ALMANAC-Ovary	w/o Prot Level	0.777±0.002	0.529±0.003	0.814±0.001	0.623±0.006	0.398±0.014	0.307±0.012
	w/o Gene Exp	0.779±0.003	0.536±0.007	0.816±0.002	0.624±0.005	0.400±0.012	0.311±0.011
ALMANAC-Skin	w/o Prot Level	0.764±0.004	0.438±0.007	0.805±0.002	0.579±0.008	0.292±0.023	0.204±0.017
	w/o Gene Exp	0.787±0.003	0.498±0.006	0.816±0.002	0.613±0.005	0.374±0.011	0.282±0.010
**Study Datasets**							
FRIEDMAN (Skin)	w/o Prot Level	0.776±0.007	0.672±0.009	0.725±0.004	0.675±0.006	0.565±0.010	0.373±0.012
	w/o Gene Exp	0.810±0.001	0.710±0.002	0.745±0.002	0.712±0.003	0.629±0.006	0.437±0.006
ONEIL (Several Tissues)	w/o Prot Level	0.801±0.003	0.751±0.005	0.689±0.008	0.445±0.011	0.727±0.005	0.724±0.006
	w/o Gene Exp	0.817±0.009	0.770±0.010	0.693±0.010	0.469±0.016	0.741±0.008	0.734±0.008

Note: w/o Prot Level denotes exclusion of the protein level data and w/o Gene Exp denotes exclusion of the gene expression data.

To quantify the contribution of protein-level expression data to predictive modeling, we conducted ablation studies comparing SynProtX-GATFP variants in which either protein-level or gene expression features were excluded. The results, summarized in **Table 1** for regression task and **Table 2** for classification task, demonstrate that the inclusion of protein-level features substantially enhances model performance across both tissue and study datasets. In regression tasks, the exclusion of gene expression features consistently led to lower RMSE and higher correlation metrics (PCC and SCC) compared to models without protein-level data. For example, in the ALMANAC-Breast dataset, excluding gene expression improved RMSE from 8.064 to 7.960 and increased PCC from 0.781 to 0.786, indicating that protein features alone capture functionally relevant variance. Similar trends were observed in ALMANAC-Lung, Ovary, and Skin datasets, suggesting that protein-level data contributes robustly to prediction accuracy. This effect was even more pronounced in the FRIEDMAN and ONEIL datasets. In ONEIL, the model with only protein-level data achieved a PCC of 0.801 compared to 0.728 without protein data, and a 10.8% increase in R^2, underscoring the generalization strength of protein features in heterogeneous, multi-tissue contexts. In classification tasks, models that retained protein-level features consistently outperformed those lacking them across all evaluation metrics, including AUROC, AUCPR, F1-score, and Cohen's Kappa. For instance, in ALMANAC-Lung, removing gene expression improved AUROC to 0.794 compared to 0.768 when protein data were excluded, a 2.6% gain.

Similar improvements were seen in the FRIEDMAN and ONEIL datasets, confirming that protein-level features significantly enhance the model's ability to distinguish synergistic versus non-synergistic drug combinations.

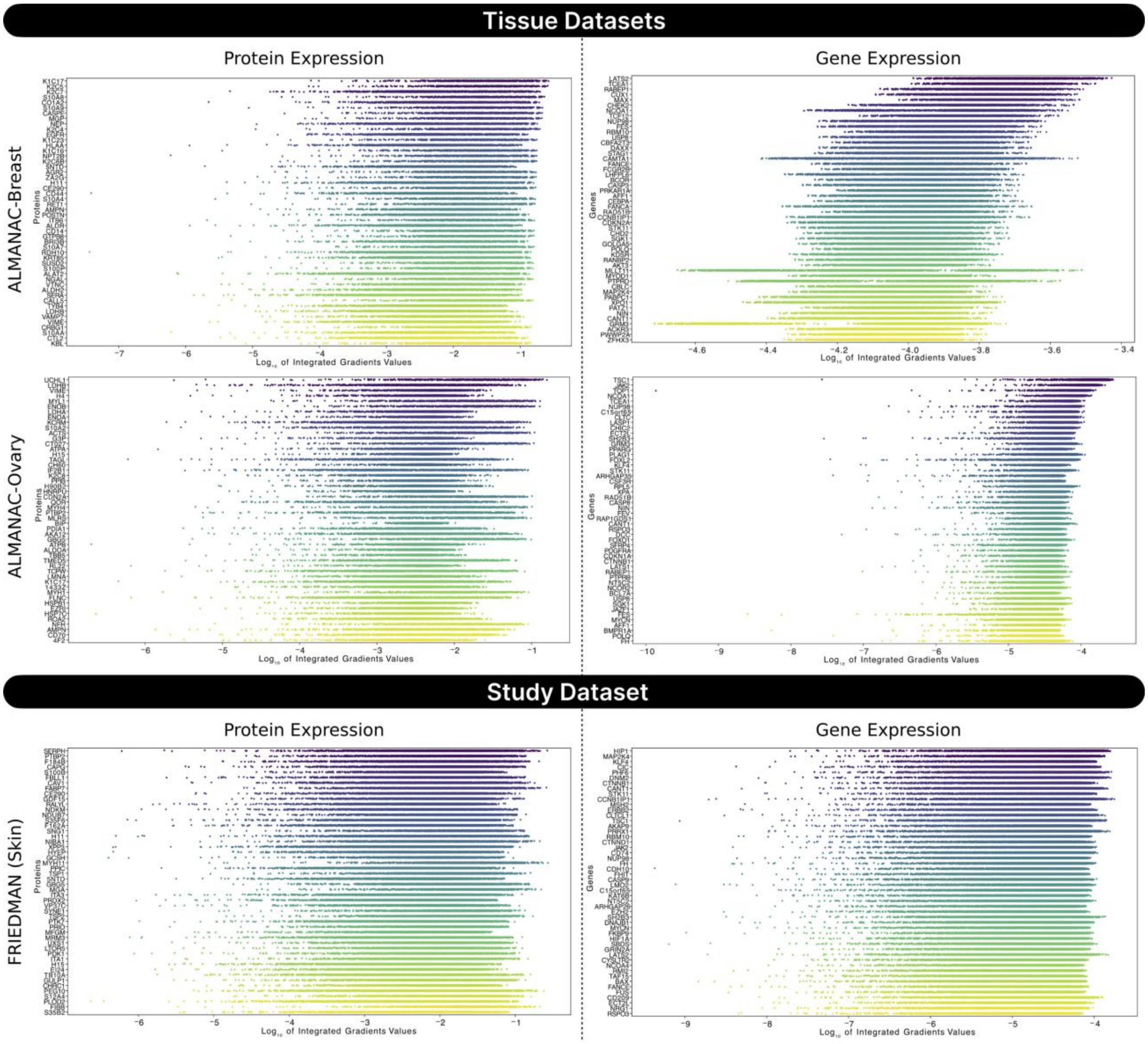

Figure 9. IG attribution scores for the top 50 protein and gene features identified by the SynProtX-GATFP regression model across Tissue Datasets (ALMANAC-Breast and ALMANAC-Ovary) and a Study Dataset (FRIEDMAN-Skin). Features are ranked based on the $\log_{10}$-transformed mean IG attribution scores, highlighting those most influential in the predictions. IG scores of exactly zero in gene features across

ALMANAC-Lung, ALMANAC-Skin, and ONEIL (Several Tissues) potentially reflect ReLU-induced gradient sparsity.

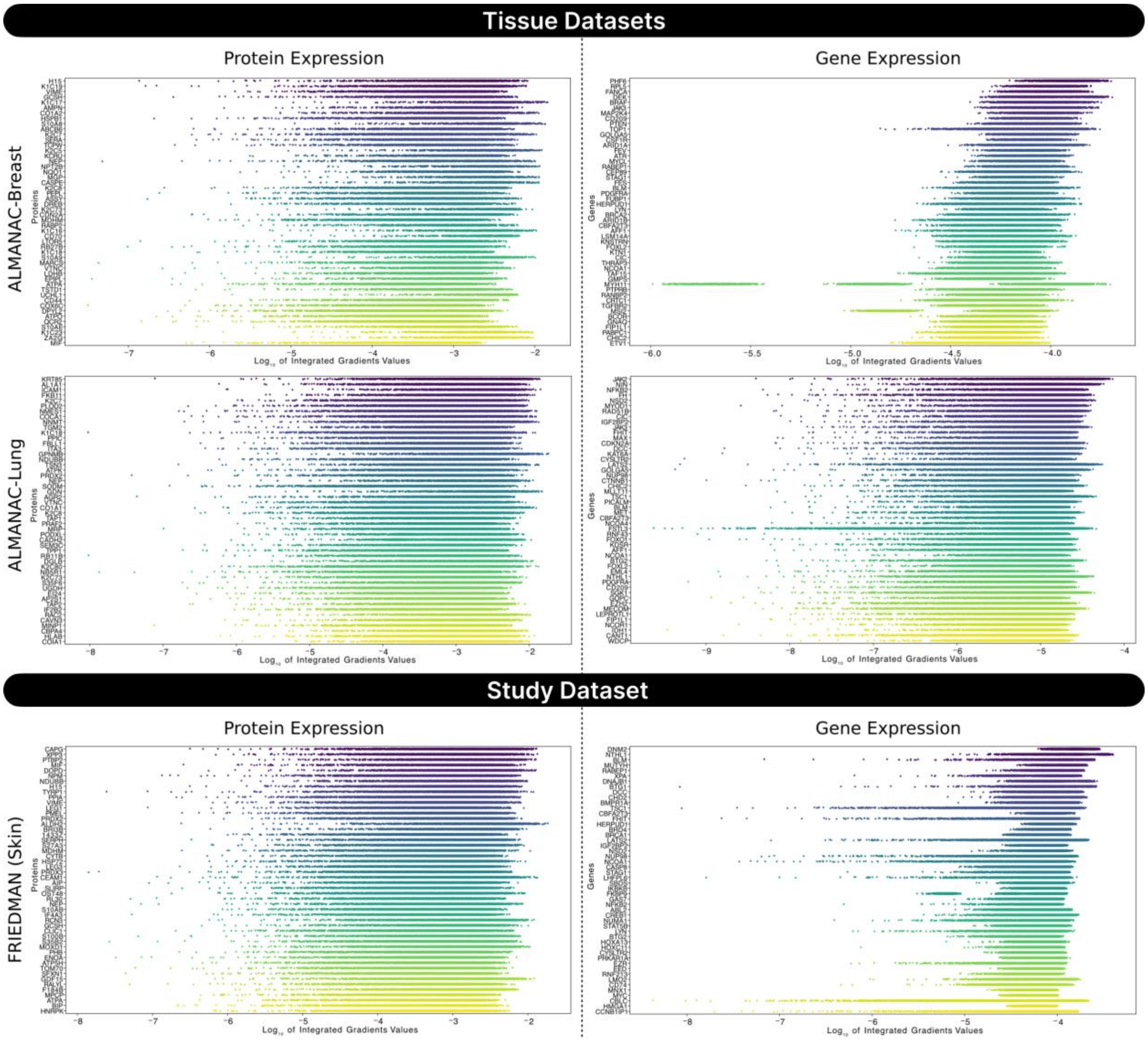

Figure 10. IG attribution scores for the top 50 protein and gene features identified by the SynProtX-GATFP classification model across Tissue Datasets (ALMANAC-Breast and ALMANAC-Lung) and a Study Dataset (FRIEDMAN-Skin). Features are ranked based on the $\log_{10}$-transformed mean IG attribution scores, highlighting those most influential in the predictions. IG scores of exactly zero in gene features across ALMANAC-Ovary potentially reflect ReLU-induced gradient sparsity.

To interpret the contribution of specific features, we employed integrated gradients (IG), a widely used attribution method designed to assess the importance of input features in deep learning models. This method satisfies key axioms such as sensitivity and implementation invariance, making it suitable for analyzing complex biological models. IG enables identification of which proteins or genes drive the model's predictions, offering transparency and mechanistic insight into the black-box architecture of SynProtX-GATFP. Using IG, we identified the top 50 most influential protein and gene features across regression (**Figure 9**) and classification (**Figure 10**) tasks. In all examined datasets, especially ALMANAC-Breast, ALMANAC-Lung, and FRIEDMAN, protein features consistently showed higher log10-transformed IG scores than gene features.

To explore the biological relevance of these top protein features, we analyzed the top 50 proteins ranked by IG scores in the ALMANAC-Breast regression model. These features were selected for their significant contribution to model predictions and provide insight into the underlying mechanisms of drug response. Several of these proteins are well-established players in breast cancer pathogenesis, particularly in aggressive subtypes. Keratins, including KRT5 and KRT17, are structural proteins typically expressed in basal epithelial cells and have been identified as markers for basal-like and triple-negative breast cancer (TNBC)—subtypes that lack hormone receptors and HER2 expression. Their elevated expression correlates with higher tumor grade, chemoresistance, and poor prognosis [42]. Similarly, KRT7 and KRT16 have been implicated in metastatic progression. KRT7 stabilizes epithelial identity and contributes to cell migration, while KRT16 expression is associated with increased tumor cell proliferation and shorter relapse-free survival in breast cancer cohorts [43, 44]. The S100 protein family plays multifunctional roles in inflammation, immune regulation, and metastasis. Notably, S100A4, S100A8, S100A9, and S100P have been found to promote epithelial–mesenchymal transition (EMT), enhance tumor invasiveness, and facilitate immune evasion mechanisms in breast cancer. Elevated expression of these proteins is commonly linked to poor survival and recurrence outcomes [45, 46]. EGFR (epidermal growth factor receptor), a transmembrane tyrosine kinase, is frequently overexpressed in TNBC and drives cellular proliferation, survival, and angiogenesis. It remains a clinically relevant therapeutic target, particularly in basal-like tumors [47]. The extracellular matrix protein COL1A2, encoding collagen type I alpha 2 chain, is upregulated in breast tumors and contributes to desmoplasia, tumor stiffness, and metastasis. Its increased expression is associated with poor

recurrence-free survival, likely due to its role in creating a pro-tumorigenic microenvironment [48]. Additional important contributors identified include CASP8, a key initiator of extrinsic apoptosis, with germline mutations associated with familial breast cancer predisposition and tumor immune evasion [49]. MGP (matrix gla protein) functions in tissue remodeling and is upregulated in TNBC; it enhances migration and proliferation through NF-κB signaling and is emerging as a potential therapeutic target [50, 51]. NEP (CD10), a membrane metallopeptidase, is typically downregulated in aggressive breast tumors, and its loss has been associated with increased invasiveness and poor prognosis [52]. Finally, HLA-A, a major histocompatibility complex class I molecule, is involved in antigen presentation and immune recognition. Aberrant expression or allelic variation of HLA-A can impair immune surveillance and promote tumor escape from cytotoxic T lymphocytes [53, 54].

Similarly, in the FRIEDMAN (Skin) classification model, IG analysis highlighted several high-attribution proteins with well-documented roles in melanoma biology. TYRP1 (tyrosinase-related protein 1) is a melanosomal enzyme involved in melanin synthesis and is overexpressed in metastatic melanoma, making it a viable target for CAR-T cell therapy due to its melanocyte specificity [55]. S100B, a calcium-binding protein, is a clinically validated biomarker in melanoma; its serum levels are used for monitoring disease progression and response to treatment, and elevated levels strongly correlate with worse survival [56, 57]. Vimentin (VIME), an intermediate filament protein, is a classical EMT marker that facilitates cytoskeletal reorganization, invasion, and metastatic spread. Its expression is a hallmark of aggressive melanoma phenotypes [58]. The antioxidant enzyme PRDX2 (peroxiredoxin-2) has been shown to inhibit EMT and reduce invasiveness in melanoma, likely through modulation of redox-sensitive transcription factors [59]. CEACAM1 (carcinoembryonic antigen-related cell adhesion molecule 1) is overexpressed in melanoma and functions as both an adhesion molecule and immune checkpoint modulator. It promotes tumor cell proliferation and facilitates immune escape by inhibiting NK and T cell-mediated cytotoxicity [60]. Lastly, GDF15 (growth differentiation factor 15), a stress-induced cytokine, has emerged as a biomarker of poor prognosis and is implicated in resistance to immune checkpoint inhibitors and reduced anti-tumor immunity in melanoma patients [61].

SynProtX-GATFP Demonstrates Generalization Capability on Cold-start Scenarios

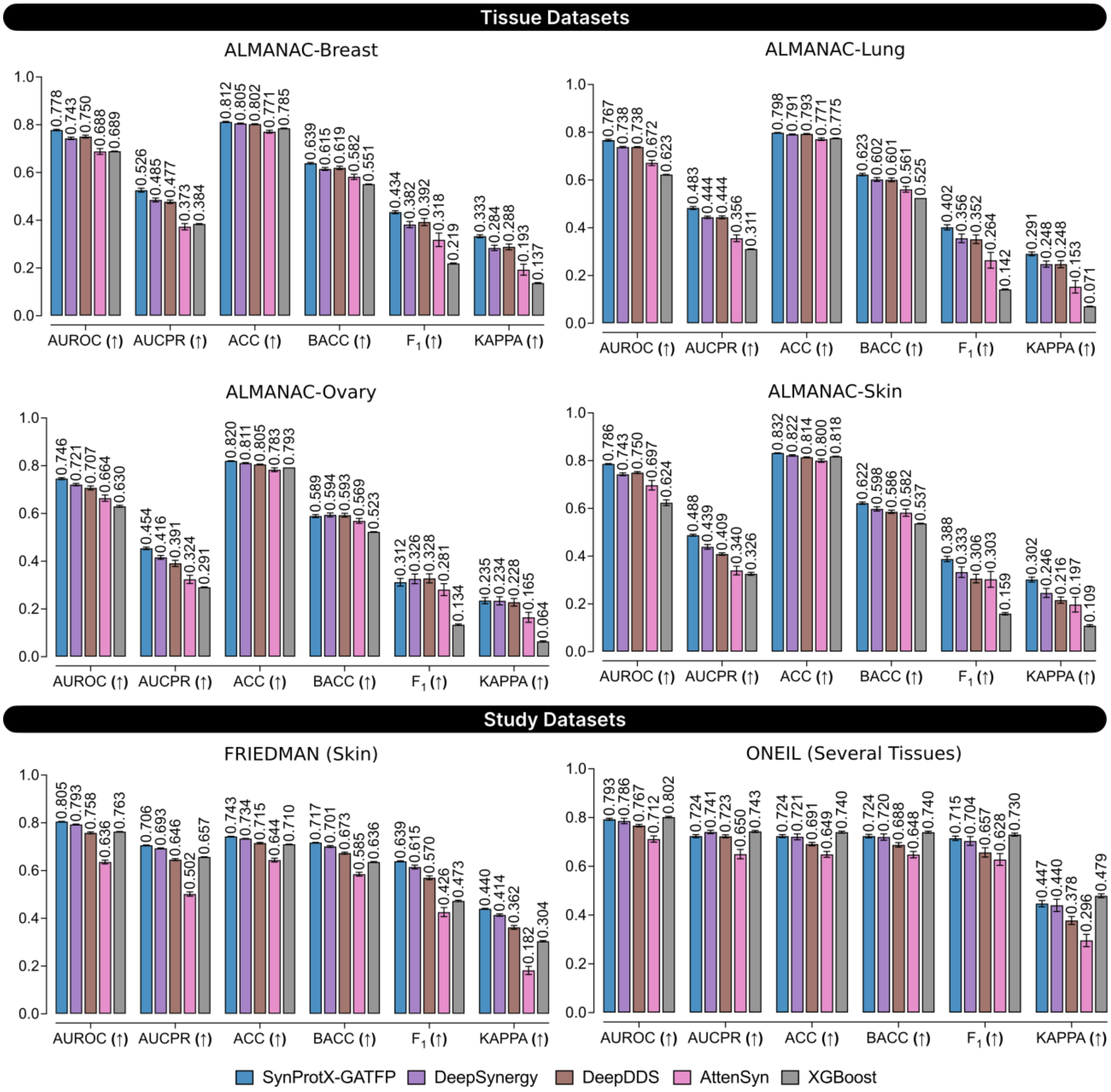

Figure 11. Model performance comparison on the classification task using the leave-drug-combination-out cross-validation technique for the Tissue Datasets: (1) ALMANAC-Breast, (2) ALMANAC-Lung, (3) ALMANAC-Ovary, and (4) ALMANAC-Skin; and the Study Datasets: (1) FRIEDMAN and (2) ONEIL with different training models.

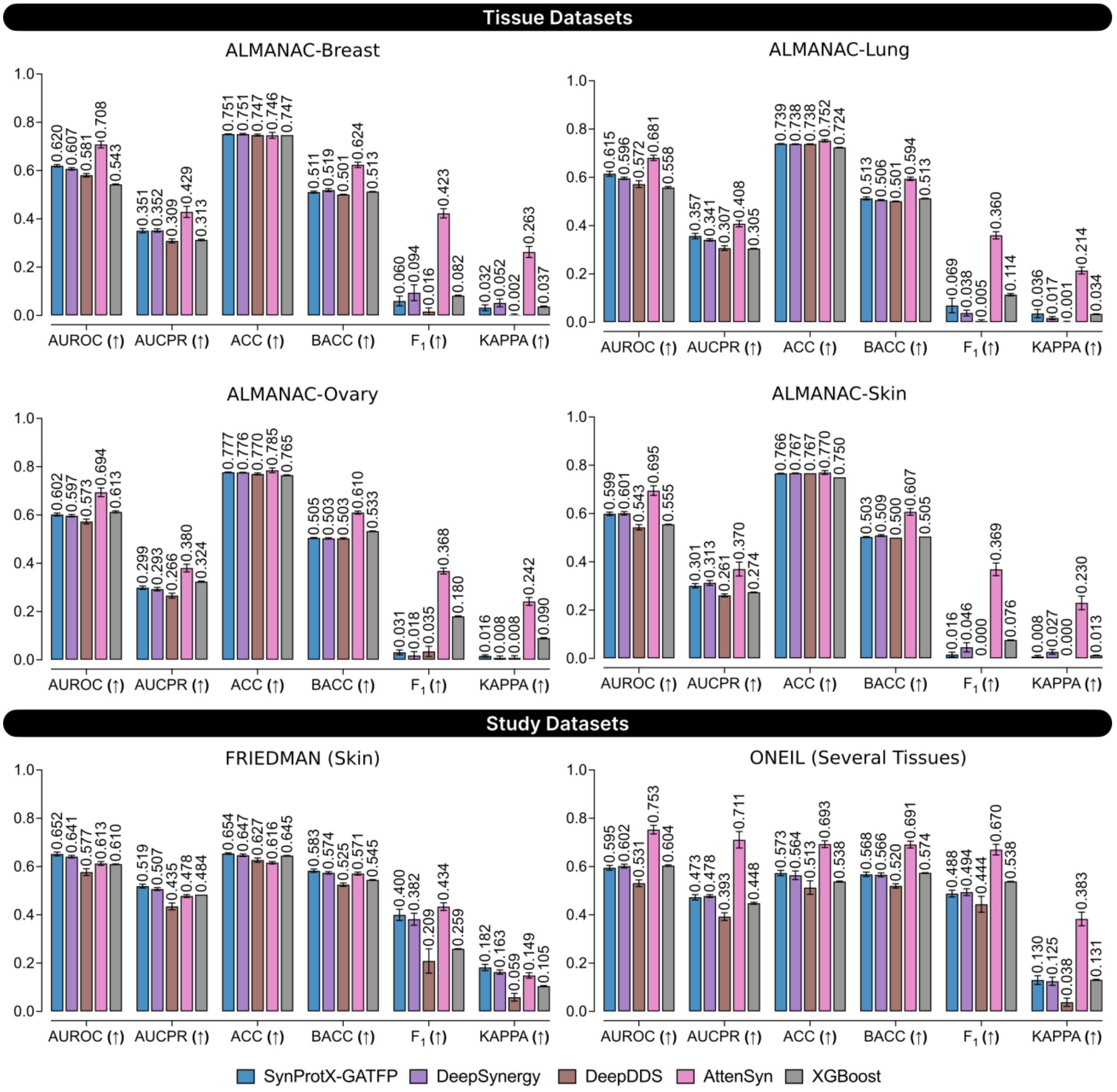

Figure 12. Model performance comparison on the classification task using the leave-drug-out cross-validation technique for the Tissue Datasets: (1) ALMANAC-Breast, (2) ALMANAC-Lung, (3) ALMANAC-Ovary, and (4) ALMANAC-Skin; and the Study Datasets: (1) FRIEDMAN and (2) ONEIL with different training models.

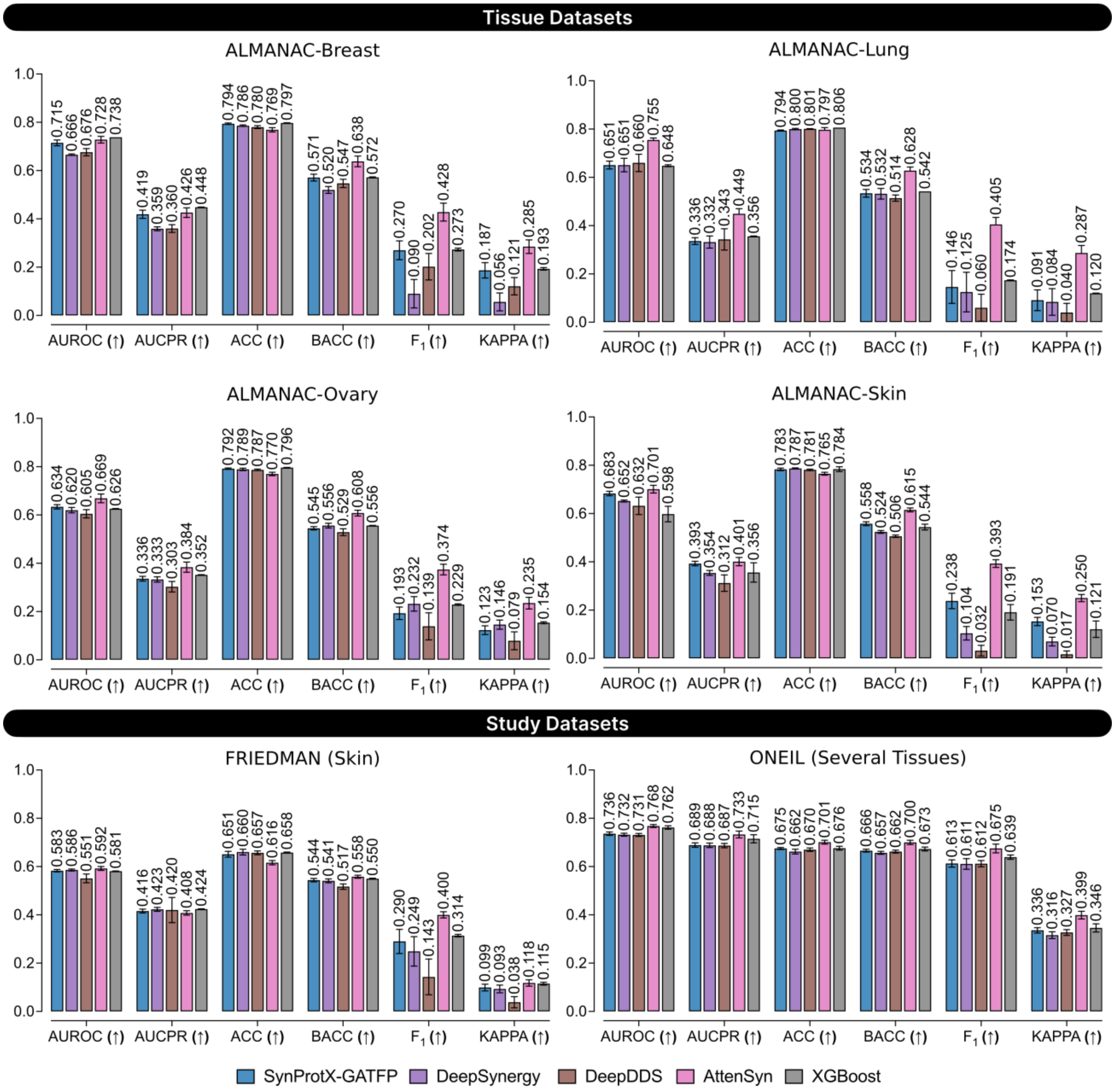

Figure 13. Model performance comparison on the classification task using the leave-cell-lines-out cross-validation technique for the Tissue Datasets: (1) ALMANAC-Breast, (2) ALMANAC-Lung, (3) ALMANAC-Ovary, and (4) ALMANAC-Skin; and the Study Datasets: (1) FRIEDMAN and (2) ONEIL with different training models.

To assess the generalizability of SynProtX-GATFP in realistic drug discovery settings, we conducted three cold-start validation experiments on classification task: (i) leave-drug-combination-out, (ii) leave-drug-out, and (iii) leave-cell-line-out. These scenarios simulate the prediction of synergy for unseen drug pairs, novel drugs, and uncharacterized cell lines, respectively. By systematically excluding specific drugs, combinations, or cell lines during training, and testing performance on these unseen cases, we can better evaluate the model's ability to generalize and practical applicability in real-world drug development pipelines [11, 62].

As shown in **Figure 11**, under the leave-drug-combination-out validation, SynProtX-GATFP consistently achieved superior performance across all metrics and datasets. Its AUROC and F_1 were notably higher in datasets like ALMANAC-Ovary, ALMANAC-Skin, and ONEIL, highlighting its strength in learning drug-pair interactions even when combinations were unseen during training. Under the more stringent leave-drug-out validation (**Figure 12**), where entire drug compounds were held out, performance decreased substantially across all models, including SynProtX-GATFP. While it still outperformed the baselines in AUROC and BACC, its F_1 and Kappa scores were considerably lower, sometimes approaching near-zero levels in datasets like FRIEDMAN and ALMANAC-Skin. These results underscore a known limitation of deep learning models in generalizing to unseen chemical space, particularly when the structural or functional characteristics of the novel compounds differ significantly from those in the training set [63, 64]. The leave-cell-lines-out validation (**Figure 13**), simulating predictions in previously unseen biological systems, presented the most difficult setting. While SynProtX-GATFP continued to outperform competing methods in AUROC and BACC, it exhibited noticeable reductions in AUCPR, F_1, and Kappa metrics, especially in datasets such as ALMANAC-Lung and ALMANAC-Breast. These results suggest a potential overfitting to cell line–specific signatures and limited capacity to adapt across different biological contexts, an issue previously reported in drug response prediction models that fail to incorporate comprehensive biological context or pan-cancer features [65, 66].

Rediscovery of Clinically Validated Synergistic and Antagonistic Drug Combinations

Table 3 Rediscovering synergistic and antagonistic effects of FDA-approved drug combinations on the test set through SynProtX-GATFP classification model.

**Drug A**	**Drug B**	**Tissue**	**Cell Line**	**Predicted Score (Probability)**	**Publication (PMCID)**
**Synergistic Effect**					
Gefitinib	Vorinostat	Lung	NCI-H226	0.9928	25552401 [67], 30365122 [68]
Docetaxel	Lapatinib	Breast	MDA-MB-468	0.8477	22999386 [69]
Gefitinib	Dasatinib	Ovary	OVCAR-5	0.9374	28446239 [70]
**Antagonistic Effect**					
Mitoxantrone	Ifosfamide	Lung	HOP-62	1.2089E-06	3735704 [71]
Chlorambucil	6-Mercaptopurine	Breast	MDA-MB-468	3.8256E-05	31813938 [72]
Temozolomide	Romidepsin	Ovary	OVCAR-8	9.6136E-05	15196862 [73]

The SynProtX-GATFP model was validated by its ability to rediscover the known synergistic and antagonistic effects of FDA-approved drug combinations, as shown in **Table 3**. This validation is crucial as it not only demonstrates the predictive accuracy of the model, but also its relevance in real-world clinical scenarios. The model accurately predicted the synergistic effects of several drug combinations across different tissues and cell lines, which is consistent with the findings of existing research. For instance, the combination of gefitinib and vorinostat was predicted to have a high probability of synergy in the lung tissue, with a score of 0.9928. This prediction is supported by studies demonstrating the feasibility and efficacy of this combination in activating apoptosis and reducing the levels of key survival proteins such as EGFR, MET, and AKT [67, 68]. Another example is the docetaxel-lapatinib combination in breast tissue, with a predicted synergy score of 0.8477. This finding is corroborated by clinical studies that have confirmed the effectiveness of this combination as a neoadjuvant treatment for $HER2^+$ breast cancer [69]. Lastly, the gefitinib-dasatinib combination was predicted to be synergistic in ovarian tissue with a score of 0.9374, consistent with research showing its anti-tumor properties through the suppression of crucial signaling pathways [70].

In addition to identifying synergistic effects, SynProtX-GATFP also successfully predicted antagonistic interactions. For example, the mitoxantrone-ifosfamide combination was expected to have a negligible probability of synergy in lung tissue, with a score of 1.2089E-06. This prediction is validated by existing clinical data indicating the limited efficacy of this combination in treating non-small cell lung cancer (NSCLC) due to its low anti-tumor activity [71]. Similarly, the combination of chlorambucil and 6-mercaptopurine in breast tissue was predicted to have an antagonistic interaction, supported by clinical trials showing limited effectiveness and significant side effects in patients with breast cancer [72]. Lastly, temozolomide and romidepsin were predicted to exhibit antagonism in the ovarian tissue, which aligns with findings indicating a lack of clinical response in treated patients [73].

SynProtX-GATFP Supports Model Explainability via Protein Networks and Molecular Substructures for Drug Combinations

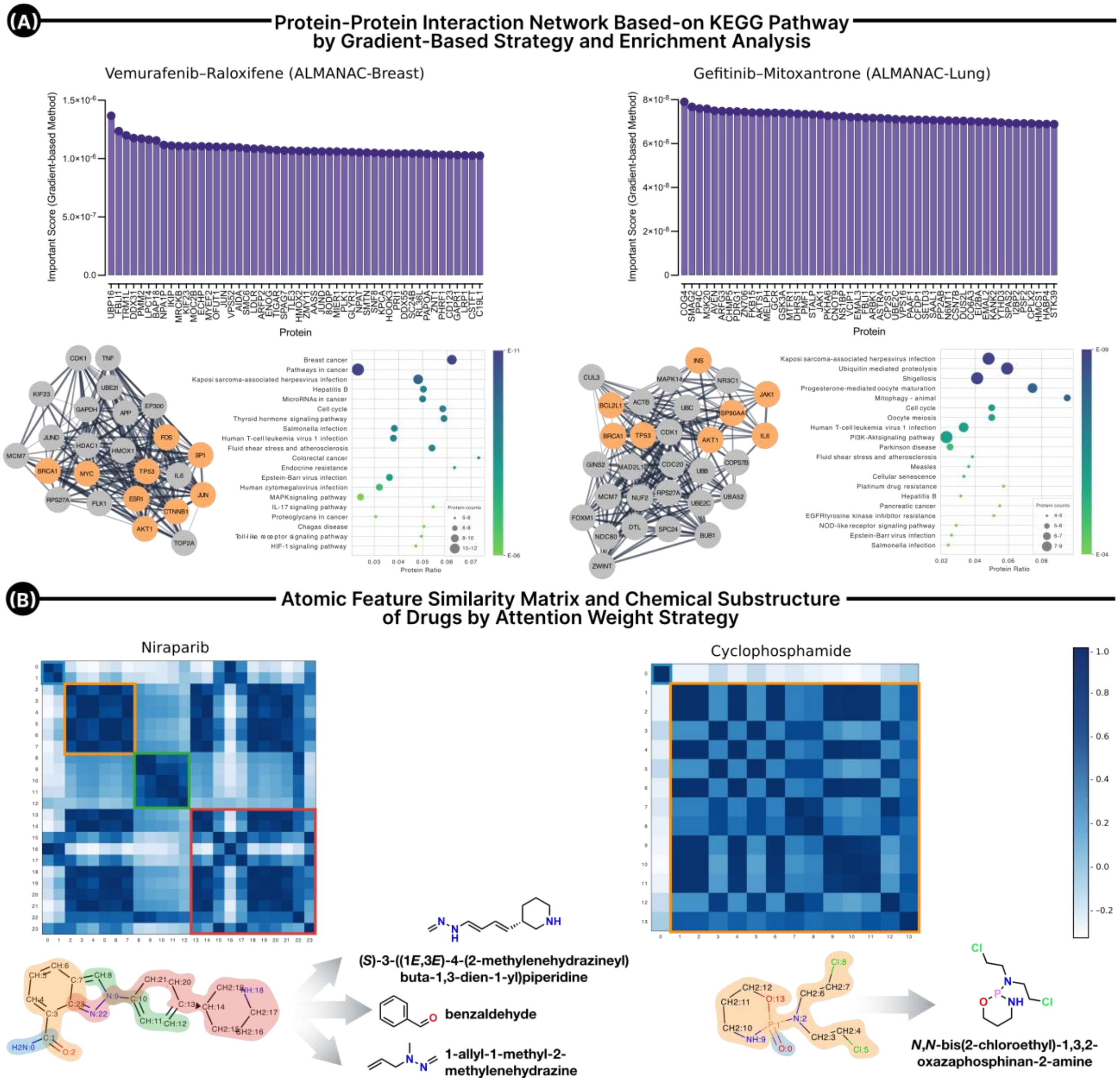

Figure 14. Model interpretability of SynProtX-GATFP. **(A)** Protein-protein interaction networks based on KEGG pathways using a gradient-based strategy and enrichment analysis for vemurafenib–raloxifene on ALMANAC-Breast dataset and gefitinib–mitoxantrone on ALMANAC-Lung dataset. Orange nodes indicate proteins associated with cancer pathways. **(B)** Atomic feature similarity matrix and chemical substructure analysis of drugs using an attention-weight strategy for niraparib and cyclophosphamide.

The SynProtX-GATFP model not only demonstrates high predictive accuracy but also offers significant explainability, which is crucial for understanding the underlying biological mechanisms driving its predictions. By leveraging gradient-based and attention-weight strategies, SynProtX-GATFP provides insights into relevant cancer-associated proteins and important chemical substructures that contribute to the model's predictions, thus supporting its interpretability. The gradient-based method from SynProtX-GATFP revealed the key cancer-associated proteins involved in the predicted synergistic effects of the drug combinations, as depicted in **Figure 14A** and **Supplementary Table S16**. For instance, the combination of vemurafenib and raloxifene in breast tissue revealed a protein-protein interaction (PPI) network with several proteins associated with breast cancer pathways, including PLK1, JUN, and MYEF2. These proteins are critical for cell cycle regulation and apoptosis, which are essential for cancer progression [74-76]. Similarly, the combination of gefitinib and mitoxantrone in lung tissue highlighted a PPI network involving proteins linked to the PI3K-Akt signaling pathway, such as AKTS1 and GSK3A, which are known to play a role in cancer cell survival and proliferation [77, 78]. The identification of these proteins and their associated pathways underscores the ability of the model to predict drug synergy and elucidate the molecular basis of these interactions. In addition to protein interactions, SynProtX-GATFP provides valuable insights into the chemical substructures driving drug synergy or antagonism through an attention-weight strategy. As shown in **Figure 14B** and **Supplementary Table S16**, the atomic feature similarity matrix for niraparib and cyclophosphamide revealed critical substructures, such as the methylenhydrazine and benzaldehyde groups in niraparib and the oxazaphosphorine derivative in cyclophosphamide, which are pivotal in their anticancer activity [79]. Piperidine, another compound highlighted in the analysis, has demonstrated significant anticancer potential in various cancer types, including breast, prostate, and colon cancers. The mechanism of action of piperidine involves the regulation of key signaling pathways such as NF-κB, PI3K/Aκt, and STAT-3, which are crucial for cancer cell survival and proliferation. Studies have shown that piperidine can induce apoptosis, inhibit cell migration, and arrest the cell cycle, making it a potent component of anticancer drug design [80]. By highlighting these specific chemical features, the model offers a deeper understanding of how molecular structure influence drug interactions, which is vital for rational drug design and development of more effective combination therapies. By providing insights into the biological and chemical features that drive its predictions, the model offers interpretable insights that help

researchers better understand the factors contributing to its predictions, moving towards more transparent and explainable outputs. This level of interpretability is crucial for validating the predictions of the model in a clinical context and gaining trust in its applications for drug development.

Integrated Gradients (IG) Method of SynProtX-GATFP Explains Block Effects

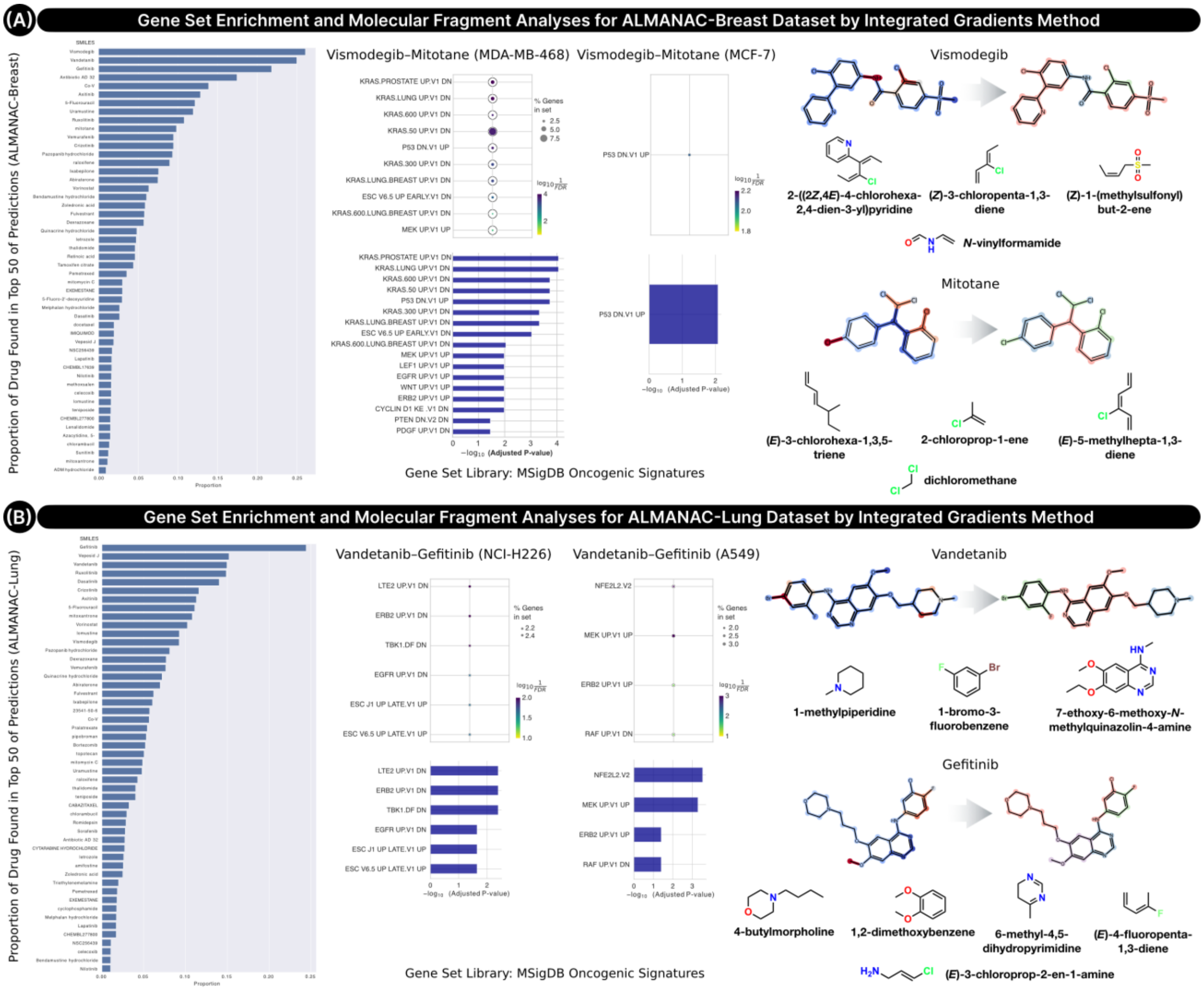

Figure 15. Block effect analysis of SynProtX-GATFP based on the top-ranked 50 proteins by IG method. **(A)** Gene set enrichment and molecular fragment analyses of vismodegib–mitotane via MDA-MB-468 and MCF-7 cell lines on the ALMANAC-Breast dataset. **(B)** Gene set enrichment and molecular fragment

analyses of vandetanib–gefitinib via NCI-H226 and A549 cell lines on the ALMANAC-Lung dataset. The gene set enrichment analysis is based on a gene set library of MSigDB Oncogenic Signature.

To investigate the observed block effects, where certain drugs consistently perform better across multiple cell lines, we conducted a mechanistic interpretability analysis using the IG method on the SynProtX-GATFP model. We selected top proportion drug found in the top 50 from the ALMANAC-Breast and ALMANAC-Lung test sets and extracted the 50 most influential proteins for each drug–cell line pair based on IG attribution scores. To elucidate the molecular mechanisms underlying these effects, we performed gene set enrichment analysis (GSEA) using the MSigDB Oncogenic Signature library, which includes curated gene sets associated with oncogenic signaling pathways and cancer driver genes. Significance was determined using false discovery rate (FDR)-adjusted p-values. Furthermore, we conducted molecular fragment analysis to identify key structural features associated with the observed pathway activations. Our analyses focused on two representative combinations: vismodegib–mitotane in breast cancer cell lines (MDA-MB-468 and MCF-7) and vandetanib–gefitinib in lung cancer cell lines (NCI-H226 and A549).

In the MDA-MB-468 cell line (TNBC), GSEA revealed significant enrichment of downregulated KRAS-associated pathways (e.g., *KRAS.LUNG.BREAST UP.V1 DN*, *KRAS.600 UP.V1 DN*) and upregulation of *P53 DN.V1 UP*, indicating suppression of KRAS signaling and activation of the p53 stress response (**Figure 15A** and **Supplementary Table S17**). This aligns with prior studies showing that vismodegib, a Hedgehog pathway inhibitor, targets hypoxic and stress-associated regions in TNBC tumors [81-83]. Mitotane, known for its broad cytotoxicity, likely enhances synergy in this context, although direct evidence in TNBC remains limited. In contrast, the MCF-7 cell line (estrogen receptor-positive) showed more selective pathway enrichment, with only *P53 DN.V1 UP* significantly activated. This suggests a p53-driven mechanism specific to this hormone-responsive subtype. Prior studies have shown mitotane's ability to suppress proliferation and alter mitochondrial metabolism in MCF-7 cells, supporting its pathway-specific activity [84, 85]. Fragment analysis further corroborates these findings. Vismodegib contains a 2-arylpyridine core and halogenated aromatic rings, facilitating its interaction with the Smoothened (SMO) receptor, an essential component of Hedgehog signaling. These features are particularly relevant in TNBC, where Hedgehog pathway components are frequently overexpressed [86]. Mitotane, structurally

derived from DDT, carries chlorinated aromatic groups that localize to the mitochondria and endoplasmic reticulum, triggering ATF4/ATF3-mediated ER stress and apoptosis, mechanisms consistent with p53 pathway activation in MCF-7 cells [87].

In the NCI-H226 cell line (squamous cell lung carcinoma), GSEA identified downregulation of multiple receptor tyrosine kinase pathways, including *EGFR UP.V1 DN*, *ERBB2 UP.V1 DN*, and *RAF UP.V1 DN* (**Figure 15B** and **Supplementary Table S18**). These findings are supported by preclinical studies showing that vandetanib inhibits tumor growth in NCI-H226-derived mesothelioma via EGFR and VEGFR2 inhibition [88]. Gefitinib has also been shown to enhance antitumor efficacy when combined with agents targeting EGFR and hypoxia-related pathways [89]. In contrast, A549 cells (EGFR wild-type adenocarcinoma) exhibited enrichment in *MEK UP.V1 UP*, *ERBB2 UP.V1 UP*, and *NFE2L2.V2* pathways, indicating activation of MAPK and oxidative stress signaling. These pathways likely reflect adaptive resistance mechanisms. Consistent with this, vandetanib suppresses p-ERK and p-JNK signaling in A549 cells [88], while gefitinib modulates the PI3K/AKT/mTOR axis and induces autophagy [90]. Molecular fragment analysis provides additional insight into these mechanisms. Both vandetanib and gefitinib share a quinazoline core, a hallmark scaffold in tyrosine kinase inhibitors, enabling selective binding to ATP-binding pockets of kinases like EGFR and VEGFR. Halogenated aromatic substitutions of vandetanib enhance its binding affinity across multiple targets, including RET, VEGFR, and EGFR, aligning with the downregulated signaling observed in NCI-H226 [91-93]. Activity of gefitinib in A549 cells is linked to its ability to modulate redox and autophagic pathways, consistent with NFE2L2 and MEK pathway enrichment [90, 94].

Together, these findings demonstrate that the observed block effects are not merely statistical artifacts but are biologically grounded in specific pathway activations, structural drug features, and cell-line contexts. The integration of IG-based feature attribution with GSEA and fragment analysis enables a biologically interpretable framework for error analysis in deep learning models. This approach enhances the mechanistic understanding of drug synergy and cell-specific responses in combination therapy prediction.

Limitations of the Study

SynProtX demonstrates strong performance in predicting the synergistic effects of anticancer drug combinations by integrating large-scale proteomic data, gene expression profiles, and molecular structures. By leveraging graph neural networks, attention mechanisms, and deep learning, the model captures complex biological interactions and provides interpretable predictions that are critical for advancing precision oncology. Despite these strengths, several limitations remain that present opportunities for further development. One key limitation lies in the model's ability to generalize to unseen data, particularly in cold-start scenarios. While SynProtX-GATFP demonstrates strong performance under traditional validation settings and leave-drug-combination-out, its predictive accuracy diminishes in more challenging scenarios—such as leave-drug-out and leave-cell-line-out evaluations. This reflects a fundamental difficulty in generalizing to novel drugs or unfamiliar cellular contexts. To address this, self-supervised pretraining on molecular graphs or few-shot learning techniques could enhance extrapolation. For example, Meta-MGNN applies molecular graph neural networks to learn molecular representations and utilizes a meta-learning framework to optimize model performance, particularly in scenarios with limited data [95]. Additionally, the model shows signs of overfitting to specific cell line signatures. While SynProtX performs well within homogeneous datasets, its consistency decreases in heterogeneous datasets, such as the ONEIL dataset, where number of datapoint is limited and cell lines span multiple tissue types. This suggests that SynProtX may not fully capture the cross-tissue biological variation. To improve generalizability, incorporating pathway-informed features or pan-cancer biological representations could help. Pathway activation models, such as DIPx, have shown improvements in personalized drug synergy predictions by focusing on tumor- and drug-specific pathway activation scores [96-98]. Another limitation of SynProtX is its reliance on static multi-omics inputs, which may not adequately reflect the dynamic nature of cellular responses to drug treatment. Cancer cells often undergo rapid molecular reprogramming in response to therapeutic agents. Integrating time-resolved omics data, such as phosphoproteomics or perturbation-induced transcriptomic profiles (e.g., LINCS L1000), could provide a more accurate representation of these adaptive processes and improve the prediction of synergistic effects under real-world conditions [99, 100]. Furthermore, data imbalance across drug–cell line pairs poses a challenge, as some cell lines or tissue types are underrepresented in the training data, leading to

inconsistencies in model performance. Future work could address this by utilizing generative modeling techniques, such as GANs or VAEs, to create synthetic omics profiles or by adopting transfer learning approaches to enrich training for rare or sparsely sampled conditions [101].

POTENTIAL IMPLICATIONS

While the primary focus of SynProtX is on predicting synergistic effects of anticancer drug combinations, the methodology and data presented in this work open up several potential avenues for broader applications that extend beyond cancer research. The integration of large-scale proteomic data, gene expression profiles, and molecular structures, paired with advanced deep learning models such as graph neural networks, can provide valuable insights for a wide range of scientific fields and industries.

Drug Discovery for Personalized Medicine to Other Diseases

SynProtX's integration of proteomic, gene expression, and molecular structure data can be adapted for drug discovery in diseases beyond cancer, such as cardiovascular, diabetes, or neurological disorders. By using patient-specific omics data, the model can predict drug interactions tailored to individual molecular profiles, improving the effectiveness and safety of treatments. This approach can help design personalized drug regimens for conditions like Alzheimer's or Parkinson's, optimizing therapy based on specific disease mechanisms and reducing adverse effects.

Pharmacoproteomics for Drug Toxicity Prediction

SynProtX's integration of proteomic data can enhance pharmacoproteomics by predicting drug toxicity. By analyzing protein expression profiles, the model can identify molecular mechanisms of drug-induced toxicity, such as liver or heart toxicity. Incorporating time-resolved proteomics and perturbation data could improve predictions of adverse effects over time, helping identify toxic compounds early in the drug development process. This could lead to safer drugs and more efficient screening methods, reducing the risk of late-stage failures.

Data-driven Biomarker Discovery

SynProtX's data-driven approach to drug synergy prediction can be applied to biomarker discovery for various diseases. By analyzing protein expression and drug interactions, the model can identify biomarkers linked to disease progression or treatment response. This could accelerate the identification of biomarkers for conditions such as autoimmune diseases, chronic inflammation, or infections, improving diagnostic accuracy and enabling personalized treatment strategies.

METHODS

Data Collection and Preparation

The dataset for developing the deep learning models was sourced from the DrugComb database version 1.5. [26, 27] This database contains drug combination data from various diseases obtained through high-throughput laboratory screenings, including cancer, malaria, Ebola, and SARS-CoV-2. Specifically, for anticancer drug combination data, studies such as and ALMANAC [32], FRIEDMAN [31], and ONEIL (O'Neil) [102] were used to compile the dataset. The records in the dataset represent drug combinations tested against cell lines, with the respective Loewe scores documented. To preprocess the data, we eliminated all invalid entries, such as blank cells. Next, records with Loewe scores higher than 50 or lower than –75 were discarded to remove any potential outliers. Additionally, only drugs available in the ChEMBL database [103-105] were included, and cell lines were limited to those with overlapping between gene expression and protein expression data. Duplicate triplets of a cell line and the corresponding standard drug names were removed. As a result, a drug combination dataset with target values of 94,862 entries was obtained, including the Tissue Datasets: (1) ALMANAC-Breast, (2) ALMANAC-Lung, (3) ALMANAC-Ovary, and (4) ALMANAC-Skin; and the Study Datasets: (1) FRIEDMAN and (2) ONEIL. (**Supplementary Table S1**).

To prepare gene expression data from human cancer cells, we slightly modified the method described by Liu Q et al. [106]. Initially, data were collected from The Cancer Dependency Map (DepMap) version 22Q2, a database that processes omics data of cancer cells from the Cancer Cell Line Encyclopedia (CCLE).[13, 14] Since the gene expression data from DepMap includes genes

present in each cell, regardless of their relevance to cancer development, it was necessary to filter for genes significant to cancer development. Gene names were sourced from the Catalogue of Somatic Mutations in Cancer (COSMIC) version 96 [107], a database that compares gene data from human cancer cells to the human genome reference (GRCh38). Finally, the identified 714 genes associated with cancer development were used for model development.

For protein expression data of cancer cells, we utilized the ProCan-DepMapSanger database [19], a comprehensive resource covering protein data for 8,498 proteins from 949 types of cancer cells and over 40 cancer types. These protein data were acquired through laboratory experiments using data-independent acquisition mass spectrometry (DIA-MS), which is noted for its high reproducibility and speed [108]. Ultimately, the identified 6,688 proteins were employed for model development.

Chemical Representations

Molecular Fingerprints: To represent the structural information of the molecules, we transformed it from a molecular graph into a molecular fingerprint. This fingerprint consists of Boolean molecular descriptors indicating the chemical and physical properties of a molecule. The Extended Connectivity Fingerprint 6 (ECFP6), a 2048-bit fingerprint generated by processing the Morgan fingerprint with a radius of 3, was used [109, 110]. ECFP6 has been widely used in traditional machine learning models, such as random forest (RF) and extreme gradient boosting (XGBoost), due to its strong performance across in various applications. The molecular fingerprints were utilized to develop the SynProtX-GATFP model and were subsequently compared with state-of-the-art models.

Molecular Graph: To transform the molecular structure data into a molecular graph format, the Simplified Molecular Input Line Entry System (SMILES) notation was used for the drug names present in the dataset. The SMILES notation was obtained from version 31 of the ChEMBL database [103, 104] and was subsequently converted into a molecular graph using the RDKit package. The molecular graph of each drug is defined as $M = (V, E)$, where V is a set of nodes

and E is a set of edges displayed in an adjacency matrix A. Each node $v_u \in V$ represents an atom u in the molecule, and each edge $e_{uw} \in E$ represents the bond e_{uw} between atoms u and w.

The Graph Neural Network (GNN) utilizes this non-Euclidean and non-invariant molecular graph to extract compound features for predicting bioactivity. For each node, the atomic and bond features $x_i \in v_u$ are calculated to represent the identity of the node. These features are binary vectors that include implicit values (one-hot and integer). During the GNN learning process, these features are employed through a message-passing phase between each node and its neighboring nodes. In this work, we utilized *ConvMolFeaturizer* for atomic features by DeepChem [111].

Molecular Graph and Deep Neural Networks

Graph Convolutional Network (GCN) [28]: GCN is a graph algorithm that is widely used in image processing, natural language processing (NLP), chemical property prediction of molecules, and systems biology, among others. The propagation process of GCN is defined by the following equation.

$$H_u^{(l+1)} = \sigma\left(\widehat{D}^{-\frac{1}{2}}\hat{A}\widehat{D}^{-\frac{1}{2}}W^{(l)}h_u^{(l)}\right) \qquad 12$$

Let $\hat{A} = A + I$, where I is the identity matrix and $\widehat{D}$ is the degree matrix of the diagonal nodes obtained from $\widehat{D}_{uw} = \sum_w \hat{A}_{uw}$. Here, D and A are the degree and adjacency matrices, respectively, according to the order of $W^{(l)}$ and $h_u^{(l)}$, which are the weights and learnable parameters from node u of layer l, respectively. σ is the non-linear activation function. $\widehat{D}^{-\frac{1}{2}}\hat{A}\widehat{D}^{-\frac{1}{2}}$ adds self-connections to the nodes and maintains feature vector size. The message passing phase consists of two steps: 1) aggregation of the neighboring node vectors h_w to generate the node vector $\hat{h}_u$ and 2) linear projection of $\hat{h}_u$ with a non-linear activation function that can be calculated from the projection function, resulting in the following equation.

$$h_u = \sigma\left(W_u \hat{h}_u\right) \qquad 13$$

Graph Attention Network (GAT) [29]: GAT is an algorithm that is an extension of the GCN algorithm. The difference between GAT and GCN is that GAT assigns weights to nodes differently using an attention mechanism to facilitate the process of data aggregation. In GCN, the weights of neighboring atoms are calculated by normalizing the sum to be the same. The propagation process of GAT can be represented with the following equation.

$$H_u^{(l+1)} = \sigma\left(\sum_{v \in N(u)} \alpha_{uw}^{(l)} W^{(l)} h_u^{(l)}\right) \quad 14$$

In this context, the notations W, $N(u)$, and σ denote the trainable weight matrix, the set of neighboring nodes of node u, and non-linear activation function, respectively. Moreover, the symbol $\alpha_{uw}^{(l)}$ represents the normalized attention score between node u and its neighbor w in the l_{th} graph convolutional layer, which is computed by the softmax function:

$$\alpha_{uw} = softmax_w(e_{uw}) = \frac{exp(e_{uw})}{\sum_{w \in N(u)} exp(e_{uw})} \quad 15$$

The variable e_{uw} denotes the output of the initial layer, which is a feed-forward neural network using the non-linear activation function of LeakyReLU and without projection (i.e., a bias vector). This can be formulated as follows:

$$e_{uw} = LeakyReLU(W \cdot [e_u, e_w]) \quad 16$$

The attention mechanism leverages the ability to selectively attend to different aspects of information to improve performance compared to traditional Graph Convolutional Networks (GCN).

Attentive FP [112]: Attentive FP is a graph neural network architecture that employs a recurrent neural network (RNN) to aggregate information from neighboring and distant atoms to the focal atom of interest. Attentive FP is a state-of-the-art algorithm that can efficiently predict the chemical properties of benchmark molecular datasets with high accuracy on many sub-datasets. One of the reasons why Attentive FP can achieve high performance is due to the fact that during

message passing, M, the attention mechanism gathers information from neighboring nodes via gated recurrent units (GRUs), which helps filter unnecessary data and select relevant data for learning. The output stage uses a summation function as the output function to convert the graph vector representation h_G, before the next prediction step, as shown in the following equation.

Message passing step:

$$m_u^{(k+1)} = \sum_{v \in N(u)} M^{(k)}\left(h_u^{(k)}, h_w^{(k)}\right) \quad 17$$

$$h_u^{(k+1)} = GRU^{(k)}\left(m_u^{(k+1)}, h_u^{(k)}\right) \quad 18$$

Output step:

$$h_G = Sum\left(h_u^{(K)} \middle| u \in G\right) \quad 19$$

Deep Neural Network (DNN): Deep neural networks are composed of multiple interconnected neurons that gather information from other neurons. The collected data is subsequently processed through non-linear activation functions to compute the probability. The learning process of deep neural networks involves intricate and rapidly changing non-linear functions that enable them to extract hierarchical features from the input data. The activation vector h of the first hidden unit and each hidden unit at $l+1$ can be computed using the following equation.

$$h_i^{(1)} = \sigma^{(1)}\left(\sum_j W_{ij}^{(1)} x_j + b_i^{(1)}\right) \quad 20$$

$$h_i^{(l+1)} = \sigma^{(l+1)}\left(\sum_j W_{ij}^{(l+1)} h_j^{(l)} + b_i^{(l+1)}\right) \quad 21$$

where x_j is the input data vector used to calculate the activation vector of the first layer $h_i^{(1)}$ and $h_j^{(l)}$ is the activation vector of layer l, W_{ij} is the weight matrix that can be learned, b is the bias vector, and σ is a non-linear activation function

In the classification task, the sigmoid activation function was added to calculate p, which is the probability of the input being a synergistic drug combination, as shown in the following equation.

$$p = sigmoid\left(\sum_j W_j^{out} h_j^l + b^{out}\right) \qquad 22$$

Model Development

The SynProtX model was developed to predict anticancer drug synergy in a particular cell line using proteomics data. This approach involves conducting both classification and regression tasks across datasets categorized by study datasets (FRIEDMAN and ONEIL) and cancer tissue datasets (ALMANAC-Breast, ALMANAC-Lung, ALMANAC-Ovary, and ALMANAC-Skin). We utilized the PyTorch [113], PyTorch Geometric (PyG) [114], and DeepChem [111] packages as their primary toolkits for developing the model.

For molecular graph learning of compounds, we conducted experimental settings through the following approaches to compare the learning of drug molecule structures:

1. **SynProtX-GATFP:** employs a graph attention network (GAT) for learning molecular structures and deep neural networks (DNNs) for molecular fingerprints
2. **SynProtX-GAT:** employs only a graph attention network (GAT) for learning molecular structures
3. **SynProtX-GCN:** employs only a graph convolutional network (GCN) for learning molecular structures
4. **SynProtX-AttFP:** employs only an Attentive FP (AttFP) for learning molecular structures

In the comparative analysis of methodologies in drug discovery, our approach incorporated the use of advanced models such as DeepSynergy, DeepDDS, and AttenSyn. In addition to these advanced

models, we compared our models with classical algorithms such as XGBoost to establish a baseline. XGBoost is recognized for its state-of-the-art performance across various machine learning challenges and is widely adopted for its robustness and efficiency in handling sparse data. This comprehensive comparison allowed us to thoroughly evaluate the efficacy of our model in the context of both contemporary and classical methodologies.

Competitive State-Of-The-Art (SOTA) Models

The study employed three advanced SOTA deep learning models including, DeepSynergy, DeepDDS, and AttenSyn to predict the synergistic effects of anticancer drug combinations.

DeepSynergy [115]**:** DeepSynergy integrates chemical descriptors and gene expression data into a fully connected neural network, utilizing features from both domains to enhance the predictive accuracy. It is particularly effective in assessing the potential of drug pairs to work synergistically by using established metrics such as AUROC and AUPR for evaluation.

DeepDDS [116]**:** DeepDDS leverages graph neural networks, specifically GCNs and GATs, to learn drug-embedding vectors from molecular graphs, integrating cell line-specific data to enhance prediction accuracy. The attention mechanism of the model captures the complex interactions between drugs and cell lines, enabling precise synergy prediction.

AttenSyn [117]**:** AttenSyn extends this approach by incorporating an attention-based pooling module that identifies and focuses on key substructures within drug molecules. This attention mechanism not only improves the model's performance but also enhances its interpretability, making it possible to discern the specific molecular features contributing to the predicted outcomes. These models collectively provide a robust framework for predicting drug synergy, supporting drug discovery, and potentially improving combination therapy strategies in oncology.

To ensure a fair comparison, all competitive SOTA models were evaluated using the same criteria as for SynProtX. This included training and testing on identical datasets using the same performance metrics and applying the same cross-validation techniques. By adhering to these consistent evaluation standards, this study provides a robust comparison of model performance,

ensuring that the reported results are directly comparable and reflective of each model's true predictive capabilities.

Model Evaluation

During model training, we randomly partitioned the dataset into training and test sets using an 80:20 ratio with the random splitting method in DeepChem [111]. To ensure robustness, we performed 5-fold cross-validation [118]. The dataset was split into 5 parts, with the model trained on 4 parts and validated on the remaining part. This process was repeated 5 times, each time with a different validation part. We employed Bayesian optimization with 30 trials using Optuna [119] to find the optimal set of hyperparameters, chosen for its efficiency and reduced time consumption with flexible neural network architectures. To further improve the training process and prevent overfitting, we implemented an early stopping mechanism. This early stopping mechanism monitors the validation loss and stops the training process if the performance does not improve for a specified number of epochs, thereby preventing overfitting and saving computational resources. Specifically, training stops if the validation loss does not improve for 30 consecutive epochs or if it increases for 10 consecutive epochs.

To ensure reliable model evaluation, we generated models with different seed numbers independently using the test set. This process was repeated 15 times. The final model in each cross-validation iteration was employed to assess the predictive performance of the model on the test set using selected metrics to compare the ground truth with the predicted drug synergies. We then calculated the mean and confidence interval (α = 0.05) of these results. For performance evaluation, we used distinct metrics for the classification and regression tasks. For the classification, we used the Area Under the Receiver Operating Characteristic Curve (AUROC), Area Under the Precision-Recall Curve (AUCPR), Accuracy (ACC), Balanced Accuracy (BACC), F_1 score (F_1), and Cohen's Kappa Coefficient (KAPPA). For the regression, we used the Root Mean Square Error (RMSE), Mean Absolute Error (MAE), Pearson Correlation Coefficient (PCC), Spearman's Rank Correlation Coefficient (SCC), and Coefficient of Determination (R^2). The classification and regression metrics are presented in the Metrics for Model Evaluation subsection of **Supplementary S1**.

Model Interpretability

Integrated Gradients Guided Top-ranked Features and Block Effect Analysis

To enhance the interpretability of the SynProtX model and identify the key contributors to predicted drug synergy, we applied integrated gradients (IG). IG computes the integral of gradients along the straight-line path from a baseline to the actual input. This method was employed to attribute scores to both protein and gene features and to analyze block effects in drug combinations, with a particular focus on protein-level contributions to the model's predictions.

The first application of IG involved calculating attribution scores for protein and gene features that contribute to the model's prediction of synergy between drug combinations. The IG method assigns a contribution score to each feature by integrating the gradients of the model's output with respect to the input, relative to a baseline, typically set to zero. The attribution score for each feature i is computed as:

$$\text{AttributionScore}_i(x) = (x_i - x_i^0) \cdot \int_0^1 \nabla F\big(x' + \alpha(x - x^0)\big)\, d\alpha \tag{23}$$

where x_i represents the feature of interest (either protein or gene expression), x_i^0 is the baseline feature (set to zero for this analysis), $\nabla F(x)$ represents the gradient of the model's output with respect to the input x, and α is the scaling factor that moves from the baseline to the actual value of the feature.

In addition to feature attribution, we investigated block effects in drug combinations. Block effects occur when one drug in a combination dominates the synergistic effect, causing the synergy to be primarily driven by this drug across multiple cell lines. To identify these block effects, we applied IG to assess the contribution of individual protein features, focusing on the most influential features identified through ablation studies, to the drug combination synergy prediction. By analyzing the attribution scores for each drug combination, we identified the dominant protein features within specific combinations, revealing which proteins had a more substantial impact on synergy. This analysis focused on combinations of high effectiveness and determined the proportion of drugs found in the top 50 over all occurrences of the respective drug, both ALMANAC-Breast and ALMANAC-Lung datasets.

Attention Weight Strategy Elaborates Key Chemical Substructure

To improve the explainability of the SynProtX-GATFP model, we employed an attention weight strategy that leverages the atomic feature similarity matrix. This method highlights the most relevant molecular substructures by assigning attention weights to atoms in the drug molecules. The attention mechanism is particularly effective in identifying the key interactions between molecular features and predicting the synergistic effects of drug combinations.

The first step in the attention weight strategy is to construct an atomic feature similarity matrix, which represents the similarity between atoms based on their chemical properties. For each drug in the combination, we used a set of atomic features, such as atomic number, bond type, aromaticity, and electron density, among others. These atomic features are then compared across all atoms within the molecular graph of a drug to calculate a similarity score between each pair of atoms. The atomic feature similarity matrix S is formulated as follows:

$$S_{uw} = \mathrm{sim}(f_u, f_w) \quad (24)$$

where S_{uw} is the similarity score between atoms u and w, f_u and f_w are the feature vectors of atoms u and w, respectively, and $\mathrm{sim}(f_u, f_w)$ is the similarity function, which typically uses cosine similarity or Euclidean distance between the feature vectors of atoms.

Next, we computed the attention weights for each atom in the molecule based on graph attention network (GAT) of the model. In the context of SynProtX-GATFP, the attention mechanism is used to assign varying importance to different atoms in the molecular graph based on their relevance to the synergy prediction. The attention weight for each atom is computed by using a learnable attention coefficient, which is updated during training to optimize the model's predictive performance. The attention weight for the edge connecting atoms is computed as Equation 15. Once the attention weights are computed, they are integrated with the atomic feature similarity matrix to highlight the most significant atomic interactions for drug synergy. The weighted atomic features are then passed through the subsequent layers of the model to make predictions. The integration step can be represented as Equation 14.

AVAILABILITY OF SOURCE CODE AND REQUIREMENTS

- **Project Name:** SynProtX
- **Project Homepage:** https://github.com/manbaritone/SynProtX
- **Operating System(s):** Platform independent
- **Programming Language:** Python
- **Other Requirements:** python 3.7.9, deepchem 2.5.0, dgl 0.9.0, dgllife 0.3.2, numpy 1.21.5, openbabel 2.4.1, optuna 3.0.5, pandas 1.3.5, pyg 2.2.0, pytorch 1.12.0, pytorch-cluster 1.6.0, pytorch-scatter 2.1.0, pytorch-sparse 0.6.16, rdkit 2019.03.2, scikit-learn 1.0.2, scipy 1.7.3, torchaudio 0.12.0, torchmetrics 0.11.0, torchvision 0.13.0
- **License:** MIT

DATA AVAILABILITY

The source code for SynProtX is available at https://github.com/manbaritone/SynProtX. The datasets, hyperparameters, and model checkpoints can be retrieved from https://doi.org/10.5281/zenodo.14040426.

ADDITIONAL FILES

Supplementary Method S1. Metrics for Model Evaluation
Supplementary Figure S1. Comparison of Scatter Plot between Ground Truth and Prediction Synergy Scores
Supplementary Figure S2. Comparison of AUROC Curve
Supplementary Figure S3. Comparison of AUCPR Curve
Supplementary Figure S4. Comparison of Distribution between Ground Truth and Prediction Synergy Scores
Supplementary Figure S5. Comparison of Regression Loss Curve
Supplementary Figure S6. Comparison of Classification Loss Curve
Supplementary Table S1. Statistic of Processed Data of Anticancer Drug Combinations

Supplementary Table S2. Predictive Performance on Regression Task Across Tissue and Study Datasets

Supplementary Table S3. Predictive Performance on Classification Task Across Tissue and Study Datasets

Supplementary Table S4. Predictive Performance on Regression Task Across Cell Lines of ALMANAC-Breast Tissue Dataset

Supplementary Table S5. Predictive Performance on Regression Task Across Cell Lines of ALMANAC-Lung Tissue Dataset

Supplementary Table S6. Predictive Performance on Regression Task Across Cell Lines of ALMANAC-Ovary Tissue Dataset

Supplementary Table S7. Predictive Performance on Regression Task Across Cell Lines of ALMANAC-Skin Tissue Dataset

Supplementary Table S8. Predictive Performance on Regression Task Across Cell Lines of FRIEDMAN Study Dataset

Supplementary Table S9. Predictive Performance on Regression Task Across Cell Lines of ONEIL Study Dataset

Supplementary Table S10. Predictive Performance on Classification Task Across Cell Lines of ALMANAC-Breast Tissue Dataset

Supplementary Table S11. Predictive Performance on Classification Task Across Cell Lines of ALMANAC-Lung Tissue Dataset

Supplementary Table S12. Predictive Performance on Classification Task Across Cell Lines of ALMANAC-Ovary Tissue Dataset

Supplementary Table S13. Predictive Performance on Classification Task Across Cell Lines of ALMANAC-Skin Tissue Dataset

Supplementary Table S14. Predictive Performance on Classification Task Across Cell Lines of FRIEDMAN Study Dataset

Supplementary Table S15. Predictive Performance on Classification Task Across Cell Lines of ONEIL Study Dataset

Supplementary Table S16. Relevant Cancer-Associated Proteins of Cancer Drug Combinations from Gradient-Based Method

Supplementary Table S17. Top-ranked 50 Proteins by Integrated Gradients Method Across SynProtX-GATFP on the ALMANAC-Breast dataset for Vismodegib–Mitotane

Supplementary Table S18. Top-ranked 50 Proteins by Integrated Gradients Method Across SynProtX-GATFP on the ALMANAC-Lung dataset for Vandetanib–Gefitinib

Supplementary Table S19. Hyperparameter Settings of SynProtX

Supplementary Table S20. Best Hyperparameter Settings of SynProtX-GATFP on Classification Task

Supplementary Table S21. Best Hyperparameter Settings of SynProtX-GATFP on Regression Task

LIST OF ABBREVIATIONS

ACC: Accuracy; **AttFP:** Attentive FP; **AUCPR:** Area Under the Precision-Recall Curve; **AUROC:** Area Under the Receiver Operating Characteristic Curve; **BACC:** Balanced Accuracy; **CCLE:** Cancer Cell Line Encyclopedia; **DL:** deep learning; **DNN:** deep neural network; **ECFP:** Extended Connectivity Fingerprint; **F_1:** F_1 score; **FDR:** false discovery rate; **GAT:** graph attention network; **GATFP:** graph attention network and molecular fingerprint; **GCN:** graph convolutional network; **GNN:** graph neural network; **GRU:** gated recurrent unit; **GSEA:** gene set enrichment analysis; **IG:** integrated gradients; **KAPPA:** Cohen's Kappa Coefficient; **MAE:** Mean Absolute Error; **ML:** machine learning; **MLP:** multilayer perceptron; Root Mean Square Error; **PCA:** principal component analysis; **PCC:** Pearson Correlation Coefficient; **PPI:** protein-protein interaction; **R^2:** Coefficient of Determination; **ReLU:** rectified linear unit; **RF:** random forest; **RMSE:** Root Mean Square Error; **RNN:** Recurrent Neural Network; **SCC:** Spearman's Rank Correlation Coefficient; **SMILE:** simplified molecular-input line-entry System; **SOTA:** state-of-the-art; **SVM:** support vector machine; **t-SNE:** t-distributed stochastic neighbor embedding; **XGBoost:** extreme gradient boosting;

AUTHORS’ CONTRIBUTIONS

B.B.: Conceptualization, Data curation, Formal analysis, Investigation, Methodology, Software, Validation, Visualization, Writing – original draft, and Writing – review & editing. **M.K.:** Conceptualization, Data curation, Formal analysis, Investigation, Methodology, Software, Writing – original draft, and Writing – review & editing. **T.N.:** Conceptualization, Formal analysis, Investigation, Methodology, and Writing – review & editing**.** **N.Y.:** Formal analysis, Methodology, Software, Validation, and Writing – review & editing. **C.T.:** Formal analysis, Validation, and Writing – review & editing. **T.R.:** Formal analysis, Supervision, Validation, Visualization, and Writing – review & editing. **S.N.:** Formal analysis, Supervision, Validation, and Writing – review & editing.

FUNDING

This work was supported by Vidyasirimedhi Institute of Science and Technology (VISTEC). Additionally, this research has received funding support from the National Science, Research and Innovation Fund (NSRF) via the Program Management Unit for Human Resources & Institutional Development, Research and Innovation (PMU-B) [grant number: B38G680006].

COMPETING INTERESTS

The authors declare that they have no competing interests.

DISCLOSURE OF GENERATIVE AI AND AI-ASSISTED TECHNOLOGIES IN THE WRITING PROCESS

The authors utilized OpenAI’s ChatGPT-4 [120] during the manuscript drafting process to enhance linguistic clarity and coherence. All scientific content, data interpretation, and conclusions were

independently generated by the authors. We have rigorously ensured the accuracy and originality of the work, adhering to established ethical guidelines for the responsible use of AI in research.

ACKNOWLEDGEMENTS

The authors sincerely thank **Can Udomcharoenchaikit** for the beneficial comments on machine learning-related concerns. The authors also thank **Thanasan Nilsu** from the Department of Biology and Environmental Science at Kamnoetvidya Science Academy (KVIS) for his valuable suggestions and advisement for his support of **Matin Kositchutima**, **Tisorn Na Phattalung**, and **Kittiphat Pongarunotai** during their time as research students at KVIS. The computational resources were provided by the School of Information Science and Technology, Vidyasirimedhi Institute of Science and Technology (VISTEC). **Bundit Boonyarit** is supported by a full-time Ph.D. study scholarship from VISTEC.

AUTHORS' INFORMATION

B.B. is a Ph.D. candidate in Information Science and Technology at the School of Information Science and Technology (IST), Vidyasirimedhi Institute of Science and Technology (VISTEC), Thailand. His research interests focus on leveraging advanced artificial intelligence and computational techniques to develop novel computational frameworks and machine learning/deep learning models that drive innovations and discoveries in biomolecular science and biomedicine. His current research lines span three core areas: (I) Molecular Representation Learning & Computations, (II) Molecular Discovery & Design, and (III) Molecular Modeling & Simulation. **Websites:** https://manbaritone.com and https://baid.manbaritone.com.

M.K. is an incoming undergraduate student in Computer Science at the Department of Computer Science, Columbia University, USA. His academic interests include artificial intelligence and computational modeling. He is sponsored by the Thai government to pursue his studies through to a Ph.D. level.

T.N. is an undergraduate student in Electrical and Electronics Engineering minor in Physics at the School of Electrical and Electronic Engineering, Nanyang Technological University (NTU), Singapore. His academic interest includes quantum information and computation, along with problems in quantum physics, and information theory. He is under ASEAN Scholarship sponsored by the Ministry of Education Singapore through his undergraduate study.

N.Y. is an undergraduate student in Computer Engineering at the School of Information, Computer, and Communication Technology (ICT), Sirindhorn International Institute of Technology (SIIT), Thammasat University, Pathum Thani, Thailand. His research interests encompass the innovation and application of contemporary artificial intelligence and computational methods, especially deep learning, to tackle the active problems with rapidly developed solutions in therapeutics.

C.T. is a Professor in Biochemistry at the Department of Immunology, Faculty of Medicine Siriraj Hospital, Mahidol University, Bangkok, Thailand, and a member of the Siriraj Center of Research Excellence for Cancer Immunotherapy (SiCORE-CIT). Her research primarily focuses on immunology and cancer biology, specifically in understanding the tumor microenvironment (TME), immune evasion mechanisms, and the development of novel immunotherapeutic strategies for cancer. **Website:** https://mahidol.elsevierpure.com/en/persons/chanitra-thuwajit.

T.R. is an Associate Professor in Chemistry at the Department of Biochemistry, Faculty of Science, Chulalongkorn University, Bangkok, Thailand. She leads the BioSIM lab. Her research interests include Computer-aided Drug Design & Discovery, Biomolecular Modeling and Simulation, Computational Chemistry, and Bioinformatics. **Website:** http://www.bc2.sc.chula.ac.th/thanyada-rungrotmongkol-assistant-professorph-d/.

S.N. is an Associate Professor in Computer Science at the School of Information Science and Technology, Vidyasirimedhi Institute of Science and Technology (VISTEC), Rayong, Thailand. He leads the Natural Language Processing and Representation Learning Lab (NRL). His research interests include Machine Learning, Deep Learning, Natural Language Processing (NLP), Large Language Model (LLM), Big Data Analytics, and Data Systems. **Website:** https://vistec.ac.th/faculty-adjunct-professors-ist-sarana-nutanong.

REFERENCES

1. Swayden M, Chhouri H, Anouar Y and Grumolato L. Tolerant/Persister Cancer Cells and the Path to Resistance to Targeted Therapy. Cells. 2020;9 12 doi:10.3390/cells9122601.

2. Arceci RJ. Mechanisms of resistance to therapy and tumor cell survival. Curr Opin Hematol. 1995;2 4:268-74. doi:10.1097/00062752-199502040-00006.

3. Hanahan D. Hallmarks of Cancer: New Dimensions. Cancer Discov. 2022;12 1:31-46. doi:10.1158/2159-8290.CD-21-1059.

4. Hanahan D and Weinberg RA. Hallmarks of cancer: the next generation. Cell. 2011;144 5:646-74. doi:10.1016/j.cell.2011.02.013.

5. Holohan C, Van Schaeybroeck S, Longley DB and Johnston PG. Cancer drug resistance: an evolving paradigm. Nat Rev Cancer. 2013;13 10:714-26. doi:10.1038/nrc3599.

6. Chou TC. Drug combination studies and their synergy quantification using the Chou-Talalay method. Cancer Res. 2010;70 2:440-6. doi:10.1158/0008-5472.CAN-09-1947.

7. Keith CT, Borisy AA and Stockwell BR. Multicomponent therapeutics for networked systems. Nat Rev Drug Discov. 2005;4 1:71-8. doi:10.1038/nrd1609.

8. Al-Lazikani B, Banerji U and Workman P. Combinatorial drug therapy for cancer in the post-genomic era. Nat Biotechnol. 2012;30 7:679-92. doi:10.1038/nbt.2284.

9. Bulusu KC, Guha R, Mason DJ, Lewis RP, Muratov E, Kalantar Motamedi Y, et al. Modelling of compound combination effects and applications to efficacy and toxicity: state-of-the-art, challenges and perspectives. Drug Discov Today. 2016;21 2:225-38. doi:10.1016/j.drudis.2015.09.003.

10. Oh M, Park S, Lee S, Lee D, Lim S, Jeong D, et al. DRIM: A Web-Based System for Investigating Drug Response at the Molecular Level by Condition-Specific Multi-Omics Data Integration. Front Genet. 2020;11:564792. doi:10.3389/fgene.2020.564792.

11. Preuer K, Lewis RPI, Hochreiter S, Bender A, Bulusu KC and Klambauer G. DeepSynergy: predicting anti-cancer drug synergy with Deep Learning. Bioinformatics. 2018;34 9:1538-46. doi:10.1093/bioinformatics/btx806.

12. Cai Z, Poulos RC, Liu J and Zhong Q. Machine learning for multi-omics data integration in cancer. iScience. 2022;25 2:103798. doi:10.1016/j.isci.2022.103798.

13. Barretina J, Caponigro G, Stransky N, Venkatesan K, Margolin AA, Kim S, et al. The Cancer Cell Line Encyclopedia enables predictive modelling of anticancer drug sensitivity. Nature. 2012;483 7391:603-7. doi:10.1038/nature11003.

14. Ghandi M, Huang FW, Jane-Valbuena J, Kryukov GV, Lo CC, McDonald ER, 3rd, et al. Next-generation characterization of the Cancer Cell Line Encyclopedia. Nature. 2019;569 7757:503-8. doi:10.1038/s41586-019-1186-3.

15. Malone JH and Oliver B. Microarrays, deep sequencing and the true measure of the transcriptome. BMC Biol. 2011;9:34. doi:10.1186/1741-7007-9-34.

16. Celebi R, Bear Don't Walk Ot, Movva R, Alpsoy S and Dumontier M. In-silico Prediction of Synergistic Anti-Cancer Drug Combinations Using Multi-omics Data. Sci Rep. 2019;9 1:8949. doi:10.1038/s41598-019-45236-6.

17. Wang J, Liu X, Shen S, Deng L and Liu H. DeepDDS: deep graph neural network with attention mechanism to predict synergistic drug combinations. Brief Bioinform. 2022;23 1 doi:10.1093/bib/bbab390.

18. Wang T, Wang R and Wei L. AttenSyn: An Attention-Based Deep Graph Neural Network for Anticancer Synergistic Drug Combination Prediction. J Chem Inf Model. 2024;64 7:2854-62. doi:10.1021/acs.jcim.3c00709.

19. Goncalves E, Poulos RC, Cai Z, Barthorpe S, Manda SS, Lucas N, et al. Pan-cancer proteomic map of 949 human cell lines. Cancer Cell. 2022;40 8:835-49 e8. doi:10.1016/j.ccell.2022.06.010.

20. Aebersold R and Mann M. Mass-spectrometric exploration of proteome structure and function. Nature. 2016;537 7620:347-55. doi:10.1038/nature19949.

21. Mertins P, Mani DR, Ruggles KV, Gillette MA, Clauser KR, Wang P, et al. Proteogenomics connects somatic mutations to signalling in breast cancer. Nature. 2016;534 7605:55-62. doi:10.1038/nature18003.

22. Zheng Z, Huang L, Wang F, Liu L, Yu J, Xie W, et al. Drug Response Modeling across Cancers: Proteomics vs. Transcriptomics. bioRxiv. 2024:2024.12. 04.626700.

23. Cai Z, Poulos RC, Aref A, Robinson PJ, Reddel RR and Zhong Q. DeePathNet: A Transformer-Based Deep Learning Model Integrating Multiomic Data with Cancer Pathways. Cancer Research Communications. 2024;4 12:3151-64.

24. Crick F. Central dogma of molecular biology. Nature. 1970;227 5258:561-3. doi:10.1038/227561a0.

25. Spirin V and Mirny LA. Protein complexes and functional modules in molecular networks. Proc Natl Acad Sci U S A. 2003;100 21:12123-8. doi:10.1073/pnas.2032324100.

26. Zagidullin B, Aldahdooh J, Zheng S, Wang W, Wang Y, Saad J, et al. DrugComb: an integrative cancer drug combination data portal. Nucleic Acids Res. 2019;47 W1:W43-W51. doi:10.1093/nar/gkz337.

27. Zheng S, Aldahdooh J, Shadbahr T, Wang Y, Aldahdooh D, Bao J, et al. DrugComb update: a more comprehensive drug sensitivity data repository and analysis portal. Nucleic Acids Res. 2021;49 W1:W174-W84. doi:10.1093/nar/gkab438.

28. Kipf TN and Welling M. Semi-supervised classification with graph convolutional networks. arXiv preprint arXiv:160902907. 2016.

29. Veličković P, Cucurull G, Casanova A, Romero A, Lio P and Bengio Y. Graph attention networks. arXiv preprint arXiv:171010903. 2017.

30. Xiong Z, Wang D, Liu X, Zhong F, Wan X, Li X, et al. Pushing the Boundaries of Molecular Representation for Drug Discovery with the Graph Attention Mechanism. J Med Chem. 2020;63 16:8749-60. doi:10.1021/acs.jmedchem.9b00959.

31. Friedman AA, Amzallag A, Pruteanu-Malinici I, Baniya S, Cooper ZA, Piris A, et al. Landscape of targeted anti-cancer drug synergies in melanoma identifies a novel BRAF-VEGFR/PDGFR combination treatment. PloS one. 2015;10 10:e0140310.

32. Holbeck SL, Camalier R, Crowell JA, Govindharajulu JP, Hollingshead M, Anderson LW, et al. The National Cancer Institute ALMANAC: A Comprehensive Screening Resource for the Detection of Anticancer Drug Pairs with Enhanced Therapeutic ActivityNCI ALMANAC of Approved Cancer Drug Combinations. Cancer research. 2017;77 13:3564-76.

33. Loewe S. The problem of synergism and antagonism of combined drugs. Arzneimittelforschung. 1953;3:285-90.

34. Malyutina A, Majumder MM, Wang W, Pessia A, Heckman CA and Tang J. Drug combination sensitivity scoring facilitates the discovery of synergistic and efficacious drug combinations in cancer. PLoS computational biology. 2019;15 5:e1006752.

35. Hyung D, Baek MJ, Lee J, Cho J, Kim HS, Park C, et al. Protein-gene Expression Nexus: Comprehensive characterization of human cancer cell lines with proteogenomic analysis. Comput Struct Biotechnol J. 2021;19:4759-69. doi:10.1016/j.csbj.2021.08.022.

36. Lobo V, Shcherbinina E, Westholm JO, Nowak I, Huang HC, Angeletti D, et al. Integrative transcriptomic and proteomic profiling of the effects of cell confluency on gene expression. Sci Data. 2024;11 1:617. doi:10.1038/s41597-024-03465-z.

37. Wang H and Hanash SM. Contributions of proteome profiling to the molecular analysis of cancer. Technol Cancer Res Treat. 2002;1 4:237-46. doi:10.1177/153303460200100404.

38. Chen MM, Li J, Mills GB and Liang H. Predicting Cancer Cell Line Dependencies From the Protein Expression Data of Reverse-Phase Protein Arrays. JCO Clin Cancer Inform. 2020;4:357-66. doi:10.1200/CCI.19.00144.

39. Bunte K, Haase S, Biehl M and Villmann T. Stochastic neighbor embedding (SNE) for dimension reduction and visualization using arbitrary divergences. Neurocomputing. 2012;90:23-45.

40. Sun Y, Sheng Z, Ma C, Tang K, Zhu R, Wu Z, et al. Combining genomic and network characteristics for extended capability in predicting synergistic drugs for cancer. Nat Commun. 2015;6:8481. doi:10.1038/ncomms9481.

41. Sun YV and Hu YJ. Integrative Analysis of Multi-omics Data for Discovery and Functional Studies of Complex Human Diseases. Adv Genet. 2016;93:147-90. doi:10.1016/bs.adgen.2015.11.004.

42. van de Rijn M, Perou CM, Tibshirani R, Haas P, Kallioniemi O, Kononen J, et al. Expression of Cytokeratins 17 and 5 Identifies a Group of Breast Carcinomas with Poor Clinical Outcome. The American Journal of Pathology. 2002;161 6:1991-6. doi:https://doi.org/10.1016/S0002-9440(10)64476-8.

43. Chen F, Chen Z, Guan T, Zhou Y, Ge L, Zhang H, et al. N6-Methyladenosine Regulates mRNA Stability and Translation Efficiency of KRT7 to Promote Breast Cancer Lung Metastasis. Cancer Research. 2021;81 11:2847-60. doi:10.1158/0008-5472.Can-20-3779.

44. Xu C, Wang S and Sun Y. The role of KRT7 in metastasis and prognosis of pancreatic cancer. Cancer Cell International. 2024;24 1:321. doi:10.1186/s12935-024-03500-4.

45. Gebhardt C, Németh J, Angel P and Hess J. S100A8 and S100A9 in inflammation and cancer. Biochemical Pharmacology. 2006;72 11:1622-31. doi:https://doi.org/10.1016/j.bcp.2006.05.017.

46. Salama I, Malone PS, Mihaimeed F and Jones JL. A review of the S100 proteins in cancer. European Journal of Surgical Oncology (EJSO). 2008;34 4:357-64. doi:https://doi.org/10.1016/j.ejso.2007.04.009.

47. Hsu JL and Hung M-C. The role of HER2, EGFR, and other receptor tyrosine kinases in breast cancer. Cancer and Metastasis Reviews. 2016;35 4:575-88. doi:10.1007/s10555-016-9649-6.

48. Rong L, Huang W, Tian S, Chi X, Zhao P and Liu F. COL1A2 is a Novel Biomarker to Improve Clinical Prediction in Human Gastric Cancer: Integrating Bioinformatics and Meta-Analysis. Pathology & Oncology Research. 2018;24 1:129-34. doi:10.1007/s12253-017-0223-5.

49. Cox A, Dunning AM, Garcia-Closas M, Balasubramanian S, Reed MWR, Pooley KA, et al. A common coding variant in CASP8 is associated with breast cancer risk. Nature Genetics. 2007;39 3:352-8. doi:10.1038/ng1981.

50. Li X, Wei R, Wang M, Ma L, Zhang Z, Chen L, et al. MGP Promotes Colon Cancer Proliferation by Activating the NF-κB Pathway through Upregulation of the Calcium Signaling Pathway. Molecular Therapy - Oncolytics. 2020;17:371-83. doi:10.1016/j.omto.2020.04.005.

51. Tan H, Zhang M, Xu L, Zhang X and Zhao Y. Gypensapogenin H suppresses tumor growth and cell migration in triple-negative breast cancer by regulating PI3K/AKT/NF-κB/MMP-9 signaling pathway. Bioorganic Chemistry. 2022;126:105913. doi:https://doi.org/10.1016/j.bioorg.2022.105913.

52. Makretsov NA, Hayes M, Carter BA, Dabiri S, Gilks CB and Huntsman DG. Stromal CD10 expression in invasive breast carcinoma correlates with poor prognosis, estrogen receptor negativity, and high grade. Modern Pathology. 2007;20 1:84-9. doi:10.1038/modpathol.3800713.

53. Steven A and Seliger B. The Role of Immune Escape and Immune Cell Infiltration in Breast Cancer. Breast Care. 2018;13 1:16-21. doi:10.1159/000486585.

54. Cabrera T, Fernandez MA, Sierra A, Garrido A, Herruzo A, Escobedo A, et al. High frequency of altered HLA class I phenotypes in invasive breast carcinomas. Human Immunology. 1996;50 2:127-34. doi:https://doi.org/10.1016/0198-8859(96)00145-0.

55. Hackett CS, Hirschhorn D, Tang MS, Purdon TJ, Marouf Y, Piersigilli A, et al. TYRP1 directed CAR T cells control tumor progression in preclinical melanoma models. Molecular Therapy Oncology. 2024;32 3 doi:10.1016/j.omton.2024.200862.

56. Harpio R and Einarsson R. S100 proteins as cancer biomarkers with focus on S100B in malignant melanoma. Clinical Biochemistry. 2004;37 7:512-8. doi:https://doi.org/10.1016/j.clinbiochem.2004.05.012.

57. Guo HB, Stoffel-Wagner B, Bierwirth T, Mezger J and Klingmüller D. Clinical significance of serum S100 in metastatic malignant melanoma. European Journal of Cancer. 1995;31 6:924-8. doi:https://doi.org/10.1016/0959-8049(95)00087-9.

58. Satelli A and Li S. Vimentin in cancer and its potential as a molecular target for cancer therapy. Cellular and Molecular Life Sciences. 2011;68 18:3033-46. doi:10.1007/s00018-011-0735-1.

59. Lee DJ, Kang DH, Choi M, Choi YJ, Lee JY, Park JH, et al. Peroxiredoxin-2 Represses Melanoma Metastasis by Increasing E-Cadherin/β-Catenin Complexes in Adherens Junctions. Cancer Research. 2013;73 15:4744-57. doi:10.1158/0008-5472.Can-12-4226.

60. Gray-Owen SD and Blumberg RS. CEACAM1: contact-dependent control of immunity. Nature Reviews Immunology. 2006;6 6:433-46. doi:10.1038/nri1864.

61. Roth P, Junker M, Tritschler I, Mittelbronn M, Dombrowski Y, Breit SN, et al. GDF-15 Contributes to Proliferation and Immune Escape of Malignant Gliomas. Clinical Cancer Research. 2010;16 15:3851-9. doi:10.1158/1078-0432.Ccr-10-0705.

62. Liu Q and Xie L. TranSynergy: Mechanism-driven interpretable deep neural network for the synergistic prediction and pathway deconvolution of drug combinations. PLoS Comput Biol. 2021;17 2:e1008653. doi:10.1371/journal.pcbi.1008653.

63. Yang K, Swanson K, Jin W, Coley C, Eiden P, Gao H, et al. Analyzing learned molecular representations for property prediction. Journal of chemical information and modeling. 2019;59 8:3370-88.

64. Hu W, Liu B, Gomes J, Zitnik M, Liang P, Pande V, et al. Strategies for pre-training graph neural networks. arXiv preprint arXiv:190512265. 2019.

65. Dincer AB, Janizek JD and Lee S-I. Adversarial deconfounding autoencoder for learning robust gene expression embeddings. Bioinformatics. 2020;36 Supplement_2:i573-i82.

66. Ma J, Fong SH, Luo Y, Bakkenist CJ, Shen JP, Mourragui S, et al. Few-shot learning creates predictive models of drug response that translate from high-throughput screens to individual patients. Nature Cancer. 2021;2 2:233-44.

67. Han JY, Lee SH, Lee GK, Yun T, Lee YJ, Hwang KH, et al. Phase I/II study of gefitinib (Iressa((R))) and vorinostat (IVORI) in previously treated patients with advanced non-small cell lung cancer. Cancer Chemother Pharmacol. 2015;75 3:475-83. doi:10.1007/s00280-014-2664-9.

68. Park SE, Kim DE, Kim MJ, Lee JS, Rho JK, Jeong SY, et al. Vorinostat enhances gefitinib-induced cell death through reactive oxygen species-dependent cleavage of HSP90 and its clients in non-small cell lung cancer with the EGFR mutation. Oncol Rep. 2019;41 1:525-33. doi:10.3892/or.2018.6814.

69. Bonnefoi H, Zaman K, Debled M, Fiche M, Fournier M, Nobahar M, et al. An European Organisation for Research and Treatment of Cancer phase I study of lapatinib and docetaxel as neoadjuvant treatment for human epidermal growth factor receptor 2 (HER2) positive locally-advanced/inflammatory or large operable breast cancer. Eur J Cancer. 2013;49 2:281-9. doi:10.1016/j.ejca.2012.08.006.

70. Thibault B and Jean-Claude B. Dasatinib + Gefitinib, a non platinum-based combination with enhanced growth inhibitory, anti-migratory and anti-invasive potency against human ovarian cancer cells. J Ovarian Res. 2017;10 1:31. doi:10.1186/s13048-017-0319-2.

71. Suga J, Saijo N, Shinkai T, Eguchi K, Sasaki Y, Sakurai M, et al. Phase II study of mitoxantrone in patients with non-small cell lung cancer. Jpn J Clin Oncol. 1986;16 2:147-51. doi:10.1093/oxfordjournals.jjco.a039131.

72. Roberts C, Strauss VY, Kopijasz S, Gourley C, Hall M, Montes A, et al. Results of a phase II clinical trial of 6-mercaptopurine (6MP) and methotrexate in patients with BRCA-defective tumours. Br J Cancer. 2020;122 4:483-90. doi:10.1038/s41416-019-0674-4.

73. Judson PL, Blair Harkness C, Boente MP, Downs LS, Jr., Argenta PA and Carson LF. A phase II evaluation of Temozolomide in patients with recurrent epithelial ovarian cancer. Gynecol Oncol. 2004;93 3:667-70. doi:10.1016/j.ygyno.2004.03.020.

74. Uchiumi T, Longo DL and Ferris DK. Cell cycle regulation of the human polo-like kinase (PLK) promoter. J Biol Chem. 1997;272 14:9166-74. doi:10.1074/jbc.272.14.9166.

75. King KL and Cidlowski JA. Cell cycle regulation and apoptosis. Annu Rev Physiol. 1998;60:601-17. doi:10.1146/annurev.physiol.60.1.601.

76. Wisdom R, Johnson RS and Moore C. c-Jun regulates cell cycle progression and apoptosis by distinct mechanisms. EMBO J. 1999;18 1:188-97. doi:10.1093/emboj/18.1.188.

77. Pap M and Cooper GM. Role of glycogen synthase kinase-3 in the phosphatidylinositol 3-Kinase/Akt cell survival pathway. J Biol Chem. 1998;273 32:19929-32. doi:10.1074/jbc.273.32.19929.

78. Fang X, Yu SX, Lu Y, Bast RC, Jr., Woodgett JR and Mills GB. Phosphorylation and inactivation of glycogen synthase kinase 3 by protein kinase A. Proc Natl Acad Sci U S A. 2000;97 22:11960-5. doi:10.1073/pnas.220413597.

79. Taetle R and Howell SB. Preclinical re-evaluation of benzaldehyde as a chemotherapeutic agent. Cancer Treat Rep. 1983;67 6:561-6.

80. Mitra S, Anand U, Jha NK, Shekhawat MS, Saha SC, Nongdam P, et al. Anticancer Applications and Pharmacological Properties of Piperidine and Piperine: A Comprehensive Review on Molecular Mechanisms and Therapeutic Perspectives. Front Pharmacol. 2021;12:772418. doi:10.3389/fphar.2021.772418.

81. Wong H, Alicke B, West KA, Pacheco P, La H, Januario T, et al. Pharmacokinetic–pharmacodynamic analysis of vismodegib in preclinical models of mutational and ligand-dependent Hedgehog pathway activation. Clinical Cancer Research. 2011;17 14:4682-92.

82. Lama-Sherpa TD, Das S, Hinshaw DC, Kammerud SC, Song PN, Alsheikh HA, et al. Quantitative longitudinal imaging reveals that inhibiting hedgehog activity alleviates the hypoxic tumor landscape. Molecular Cancer Research. 2021;20 1:150-60.

83. Habib JG and O'Shaughnessy JA. The hedgehog pathway in triple-negative breast cancer. Cancer medicine. 2016;5 10:2989-3006.

84. Dworakowska D, Szyszka P, Waszut U, Weitsman G, Smith M, Diaz-Cano SJ, et al. FRI341: Mitotane Inhibits Proliferation and Affects Mitochondrial Metabolism in Human Breast, Lung and Colon Cancer Cell Lines. 2015.

85. Vickers PJ, Dickson RB, Shoemaker R and Cowan KH. A multidrug-resistant MCF-7 human breast cancer cell line which exhibits cross-resistance to antiestrogens and hormone-independent tumor growth in vivo. Molecular Endocrinology. 1988;2 10:886-92.

86. De Smaele E, Ferretti E and Gulino A. Vismodegib, a small-molecule inhibitor of the hedgehog pathway for the treatment of advanced cancers. Current opinion in investigational drugs (London, England: 2000). 2010;11 6:707-18.

87. Schiavon A, Saba L, Evaristo C, Petiti J, Pignochino Y, Ferrero G, et al. Mitotane activates ATF4/ATF3 axis triggering endoplasmic reticulum stress in adrenocortical carcinoma cells. Biomedicine & Pharmacotherapy. 2025;184:117917.

88. Zhou Y, Zhang Y, Zou H, Cai N, Chen X, Xu L, et al. The multi-targeted tyrosine kinase inhibitor vandetanib plays a bifunctional role in non-small cell lung cancer cells. Scientific Reports. 2015;5 1:8629.

89. Hu H, Miao X-K, Li J-Y, Zhang X-W, Xu J-J, Zhang J-Y, et al. YC-1 potentiates the antitumor activity of gefitinib by inhibiting HIF-1α and promoting the endocytic trafficking and degradation of EGFR in gefitinib-resistant non-small-cell lung cancer cells. European Journal of Pharmacology. 2020;874:172961.

90. Zhao Z-Q, Yu Z-Y, Li J and Ouyang X-N. Gefitinib induces lung cancer cell autophagy and apoptosis via blockade of the PI3K/AKT/mTOR pathway. Oncology letters. 2016;12 1:63-8.

91. Ciardiello F, Bianco R, Caputo R, Caputo R, Damiano V, Troiani T, et al. Antitumor activity of ZD6474, a vascular endothelial growth factor receptor tyrosine kinase inhibitor, in human cancer cells with acquired resistance to antiepidermal growth factor receptor therapy. Clinical Cancer Research. 2004;10 2:784-93.

92. Ciardiello F, Caputo R, Damiano V, Caputo R, Troiani T, Vitagliano D, et al. Antitumor effects of ZD6474, a small molecule vascular endothelial growth factor receptor tyrosine kinase inhibitor, with additional activity against epidermal growth factor receptor tyrosine kinase. Clinical Cancer Research. 2003;9 4:1546-56.

93. Ryan A and Wedge S. ZD6474–a novel inhibitor of VEGFR and EGFR tyrosine kinase activity. British journal of cancer. 2005;92 1:S6-S13.

94. Bokobza SM, Jiang Y, Weber AM, Devery AM and Ryan AJ. Combining AKT inhibition with chloroquine and gefitinib prevents compensatory autophagy and induces cell death in EGFR mutated NSCLC cells. Oncotarget. 2014;5 13:4765.

95. Guo Z, Zhang C, Yu W, Herr J, Wiest O, Jiang M, et al. Few-shot graph learning for molecular property prediction. In: *Proceedings of the web conference 2021* 2021, pp.2559-67.

96. Trac QT, Huang Y, Erkers T, Östling P, Bohlin A, Österroos A, et al. Pathway activation model for personalized prediction of drug synergy. eLife Sciences Publications, Ltd, 2025.

97. Tang Y-C and Gottlieb A. SynPathy: Predicting Drug Synergy through Drug-Associated Pathways Using Deep Learning. Molecular Cancer Research. 2022;20 5:762-9. doi:10.1158/1541-7786.Mcr-21-0735.

98. Liu Q and Xie L. TranSynergy: Mechanism-driven interpretable deep neural network for the synergistic prediction and pathway deconvolution of drug combinations. PLOS Computational Biology. 2021;17 2:e1008653. doi:10.1371/journal.pcbi.1008653.

99. Coker EA, Stewart A, Ozer B, Minchom A, Pickard L, Ruddle R, et al. Individualized Prediction of Drug Response and Rational Combination Therapy in NSCLC Using

Artificial Intelligence–Enabled Studies of Acute Phosphoproteomic Changes. Molecular Cancer Therapeutics. 2022;21 6:1020-9. doi:10.1158/1535-7163.Mct-21-0442.

100. Duan Q, Reid SP, Clark NR, Wang Z, Fernandez NF, Rouillard AD, et al. L1000CDS2: LINCS L1000 characteristic direction signatures search engine. npj Systems Biology and Applications. 2016;2 1:16015. doi:10.1038/npjsba.2016.15.

101. Ahmed KT, Sun J, Cheng S, Yong J and Zhang W. Multi-omics data integration by generative adversarial network. Bioinformatics. 2021;38 1:179-86. doi:10.1093/bioinformatics/btab608.

102. O'Neil J, Benita Y, Feldman I, Chenard M, Roberts B, Liu Y, et al. An Unbiased Oncology Compound Screen to Identify Novel Combination Strategies. Molecular Cancer Therapeutics. 2016;15 6:1155-62. doi:10.1158/1535-7163.Mct-15-0843.

103. Davies M, Nowotka M, Papadatos G, Dedman N, Gaulton A, Atkinson F, et al. ChEMBL web services: streamlining access to drug discovery data and utilities. Nucleic Acids Res. 2015;43 W1:W612-20. doi:10.1093/nar/gkv352.

104. Mendez D, Gaulton A, Bento AP, Chambers J, De Veij M, Felix E, et al. ChEMBL: towards direct deposition of bioassay data. Nucleic Acids Res. 2019;47 D1:D930-D40. doi:10.1093/nar/gky1075.

105. Zdrazil B, Felix E, Hunter F, Manners EJ, Blackshaw J, Corbett S, et al. The ChEMBL Database in 2023: a drug discovery platform spanning multiple bioactivity data types and time periods. Nucleic acids research. 2024;52 D1:D1180-D92.

106. Liu Q, Hu Z, Jiang R and Zhou M. DeepCDR: a hybrid graph convolutional network for predicting cancer drug response. Bioinformatics. 2020;36 Supplement_2:i911-i8.

107. Sondka Z, Bamford S, Cole CG, Ward SA, Dunham I and Forbes SA. The COSMIC Cancer Gene Census: describing genetic dysfunction across all human cancers. Nature Reviews Cancer. 2018;18 11:696-705.

108. Krasny L and Huang PH. Data-independent acquisition mass spectrometry (DIA-MS) for proteomic applications in oncology. Molecular omics. 2021;17 1:29-42.

109. Rogers D, Brown RD and Hahn M. Using extended-connectivity fingerprints with Laplacian-modified Bayesian analysis in high-throughput screening follow-up. J Biomol Screen. 2005;10 7:682-6. doi:10.1177/1087057105281365.

110. Rogers D and Hahn M. Extended-connectivity fingerprints. J Chem Inf Model. 2010;50 5:742-54. doi:10.1021/ci100050t.

111. Ramsundar B, Eastman P, Walters P and Pande V. Deep learning for the life sciences: applying deep learning to genomics, microscopy, drug discovery, and more. O'Reilly Media; 2019.

112. Xiong Z, Wang D, Liu X, Zhong F, Wan X, Li X, et al. Pushing the boundaries of molecular representation for drug discovery with the graph attention mechanism. Journal of medicinal chemistry. 2019;63 16:8749-60.

113. Paszke A, Gross S, Massa F, Lerer A, Bradbury J, Chanan G, et al. Pytorch: An imperative style, high-performance deep learning library. Advances in neural information processing systems. 2019;32.

114. Fey M and Lenssen JE. Fast graph representation learning with PyTorch Geometric. arXiv preprint arXiv:190302428. 2019.

115. Preuer K, Lewis RP, Hochreiter S, Bender A, Bulusu KC and Klambauer G. DeepSynergy: predicting anti-cancer drug synergy with Deep Learning. Bioinformatics. 2018;34 9:1538-46.

116. Wang J, Liu X, Shen S, Deng L and Liu H. DeepDDS: deep graph neural network with attention mechanism to predict synergistic drug combinations. Briefings in Bioinformatics. 2022;23 1:bbab390.

117. Wang T, Wang R and Wei L. AttenSyn: an attention-based deep graph neural network for anticancer synergistic drug combination prediction. Journal of Chemical Information and Modeling. 2023.

118. Baumann D and Baumann K. Reliable estimation of prediction errors for QSAR models under model uncertainty using double cross-validation. J Cheminform. 2014;6 1:47. doi:10.1186/s13321-014-0047-1.

119. Akiba T, Sano S, Yanase T, Ohta T and Koyama M. Optuna: A next-generation hyperparameter optimization framework. In: *Proceedings of the 25th ACM SIGKDD international conference on knowledge discovery & data mining* 2019, pp.2623-31.

120. OpenAI. ChatGPT (GPT-4o, May 2024 Version) [Large language model]. Response to query made by Bundit Boonyarit. https://chat.openai.com/chat. 2024.

Figure 1

Click here to access/download;Figure;fig1_model_architect.pdf

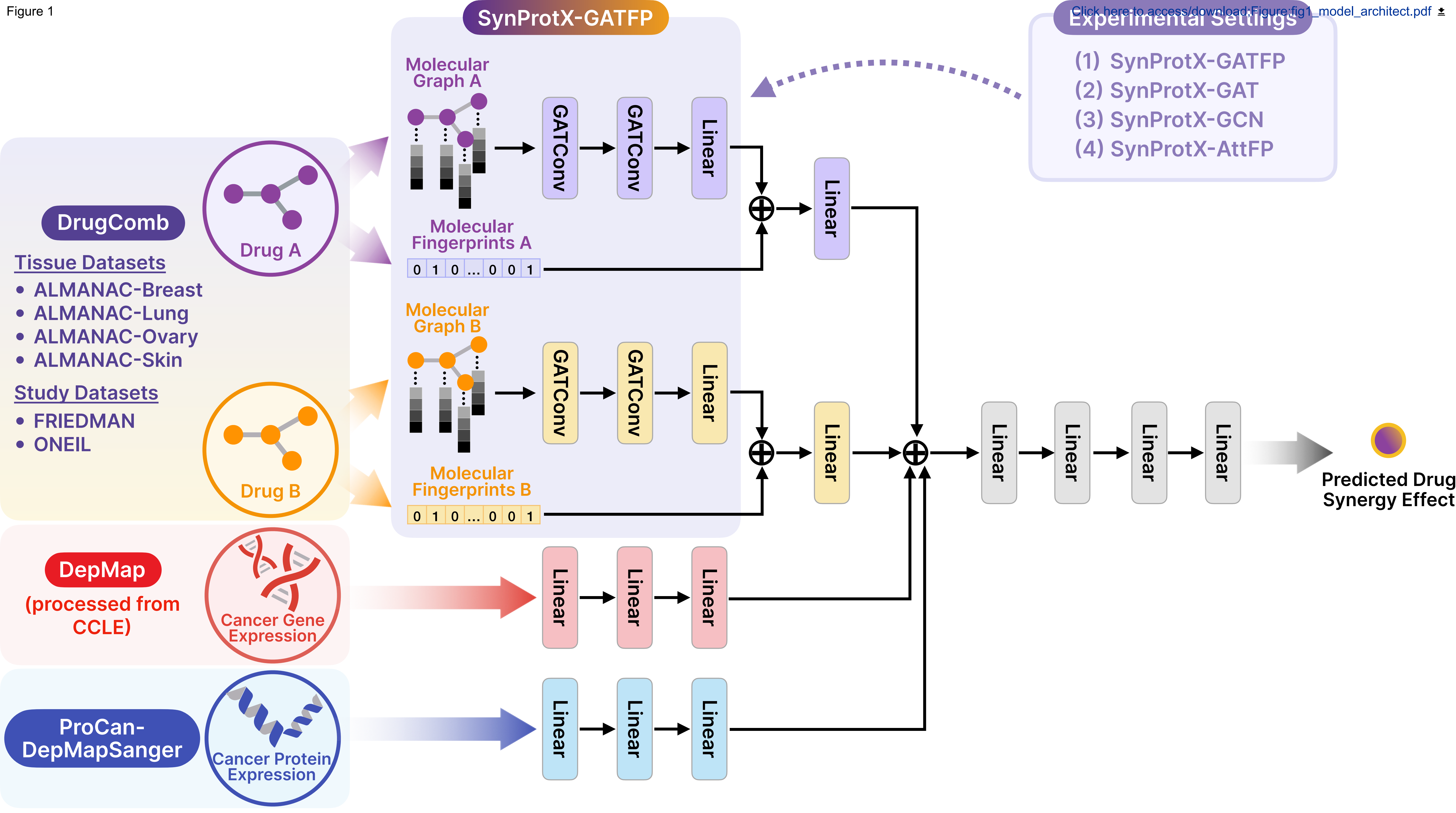

[illegible]e 2 Click here to access/download;Figure;fig2_data_viz.pdf

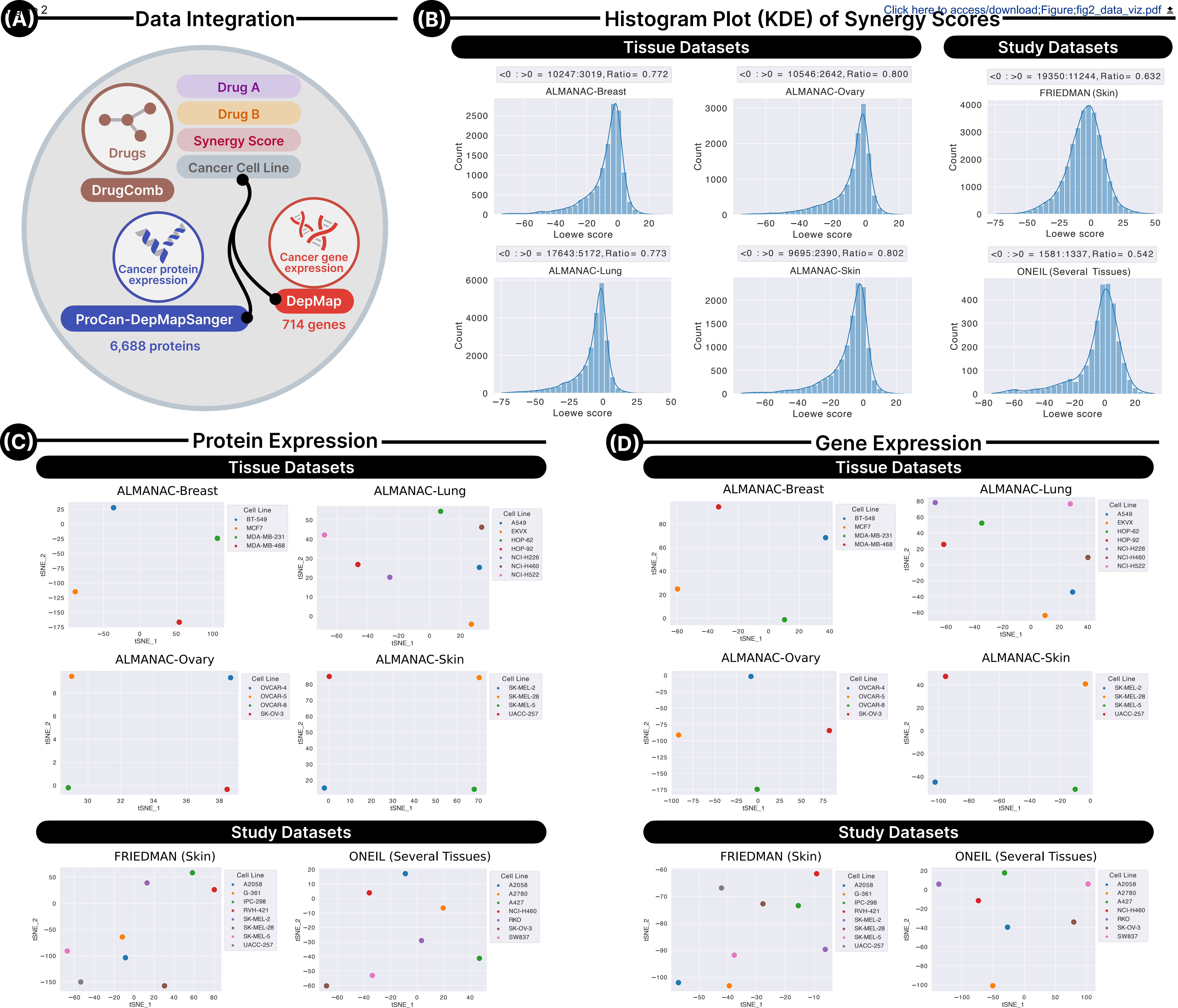

Click here to access/download;Figure;fig3_regr_performance.pdf

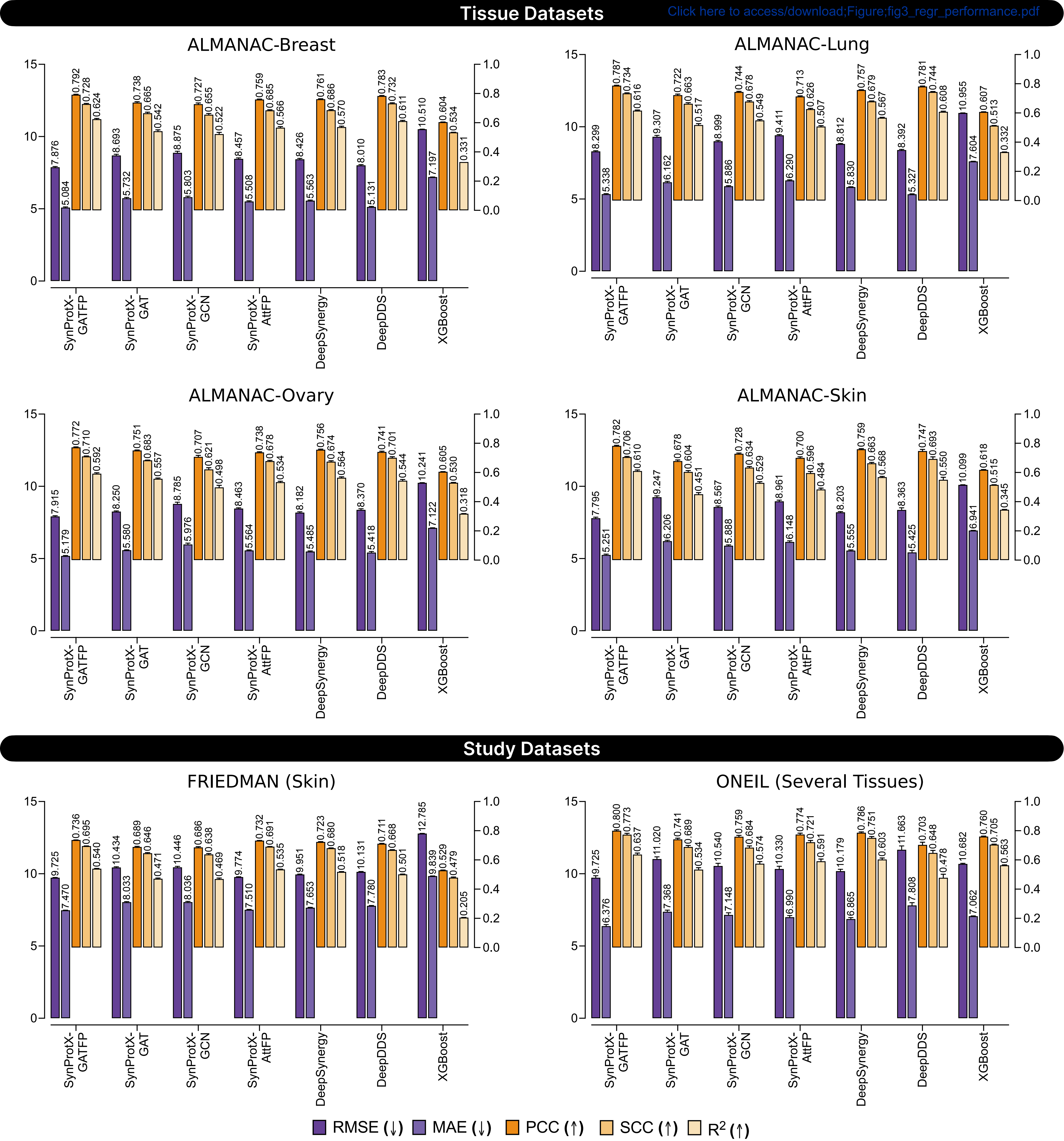

Click here to access/download;Figure;fig4_clas_performance.pdf

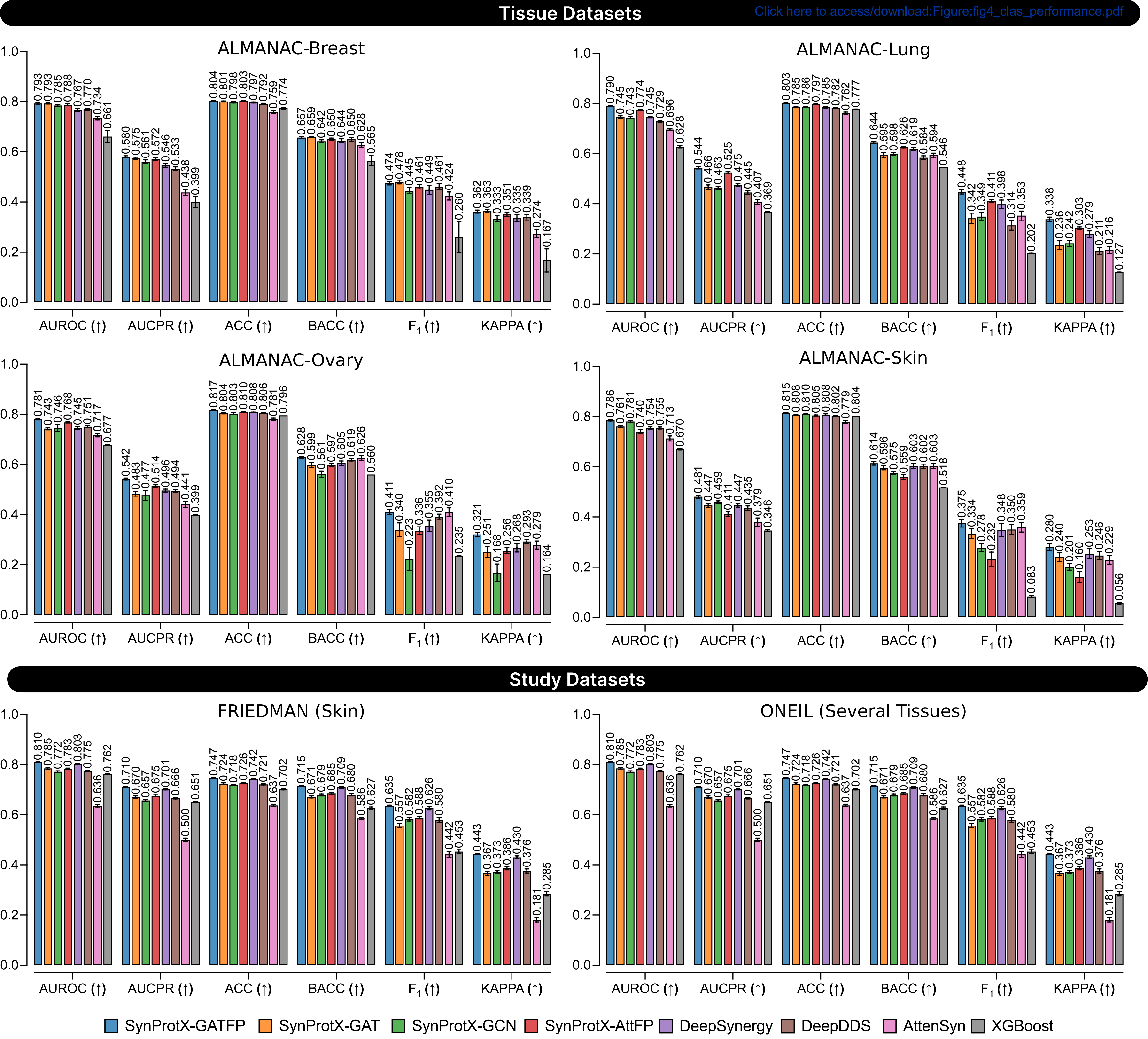

Figure
Click here to access/download;Figure;fig5_feature_learning.pdf

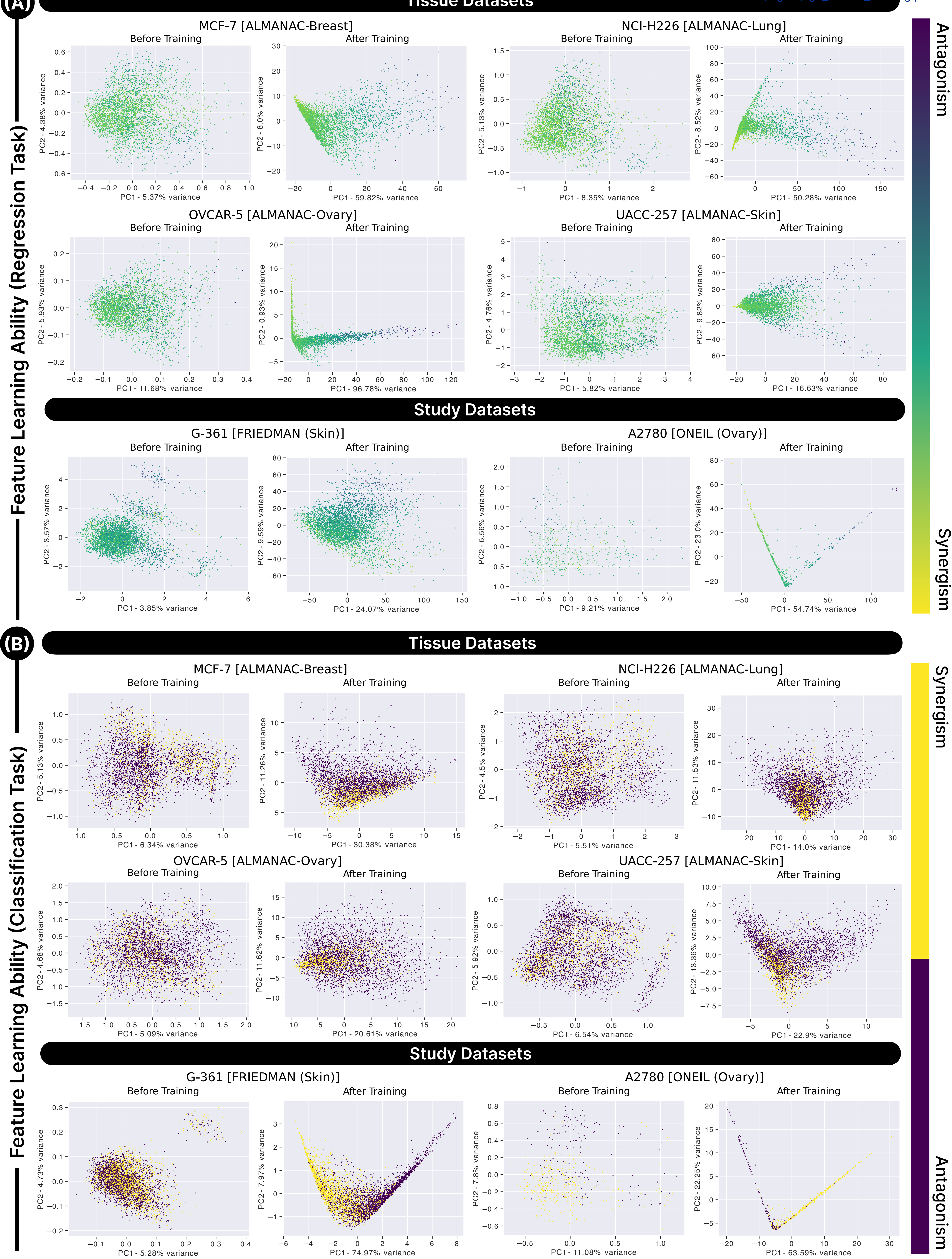

Click here to access/download;Figure;fig6_regr_cell_performance.pdf

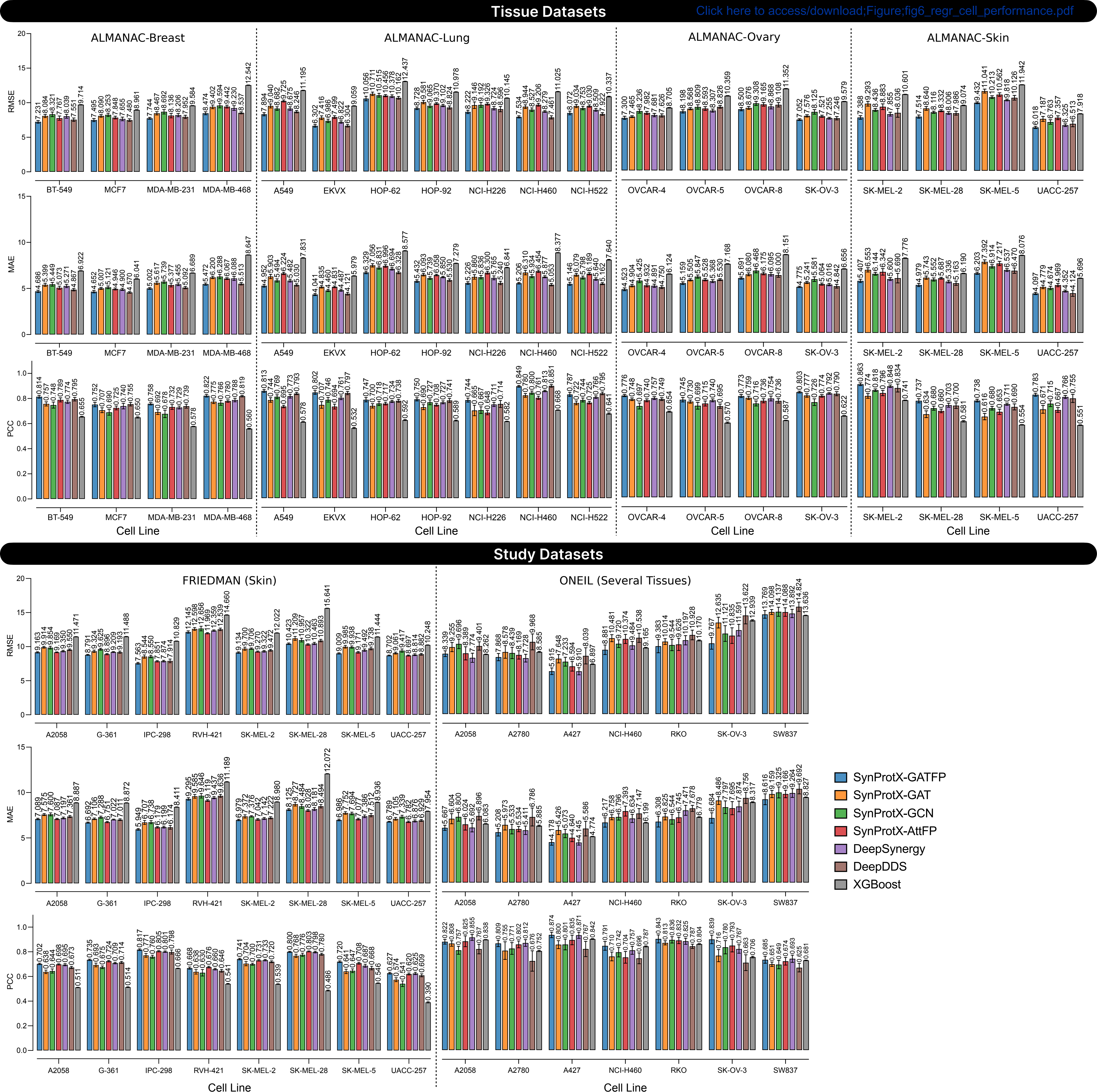

Click here to access/download;Figure;fig7_regr_cell_violinplot.pdf

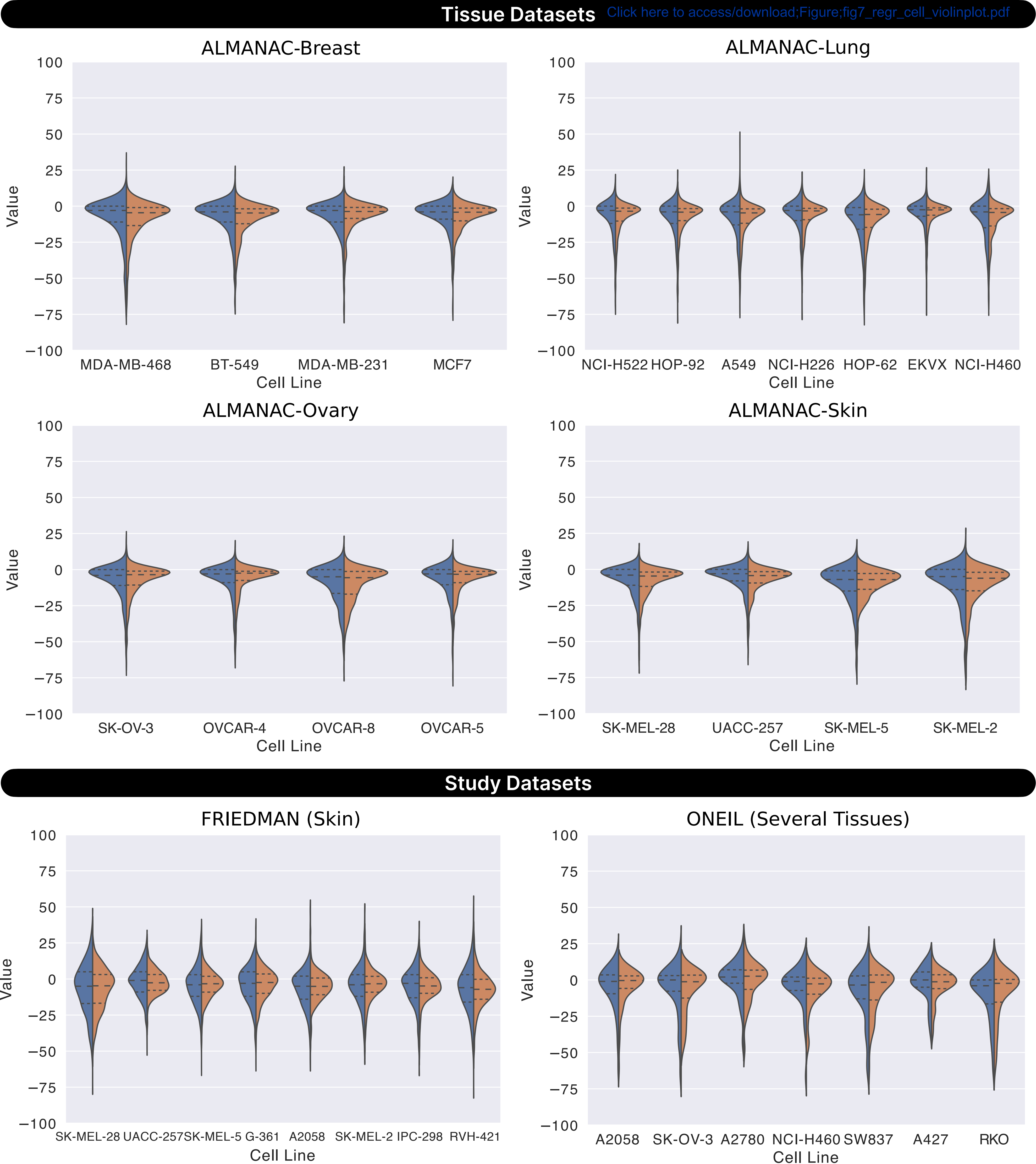

Click here to access/download;Figure;fig8_clas_cell_performance.pdf

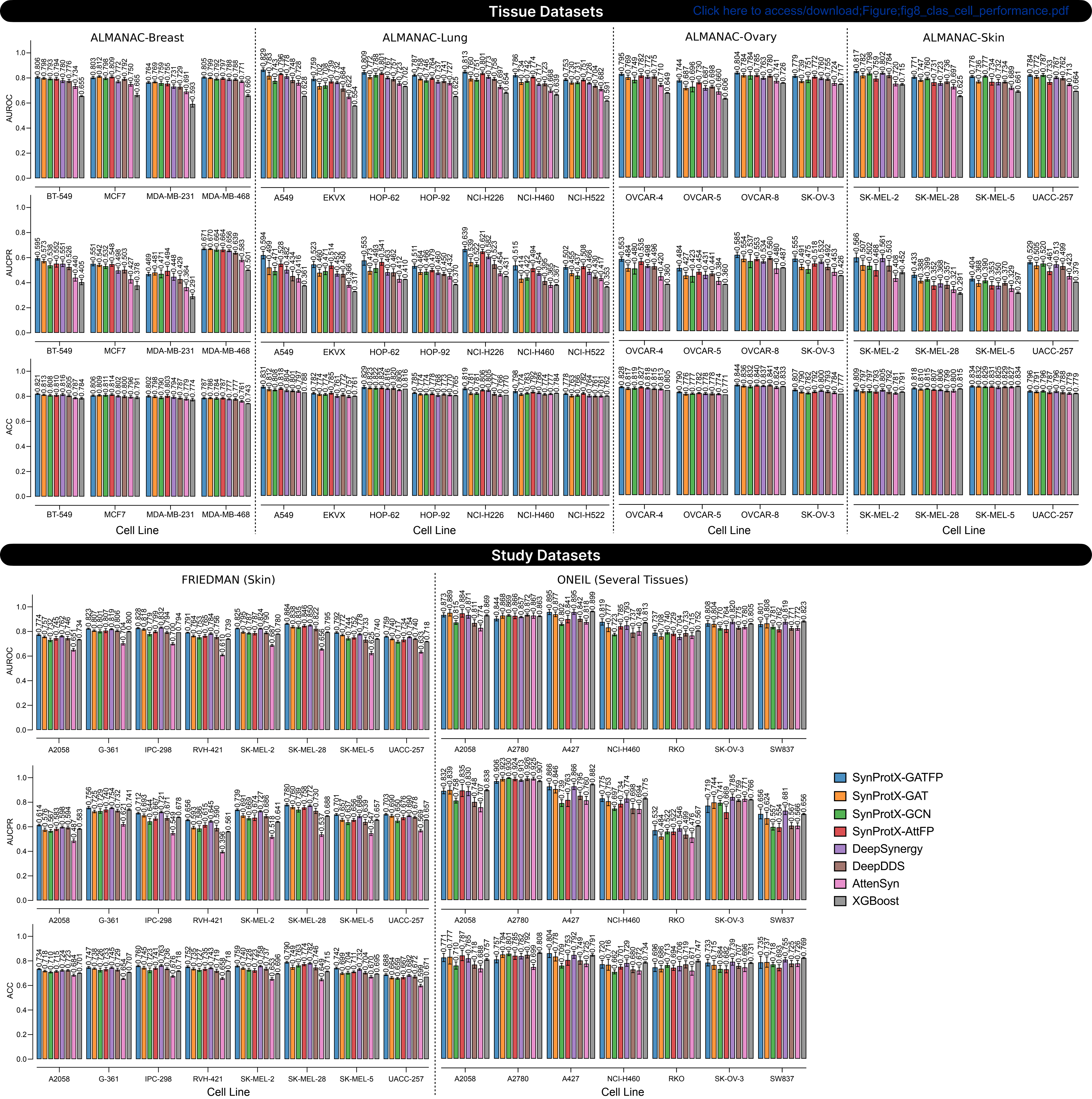

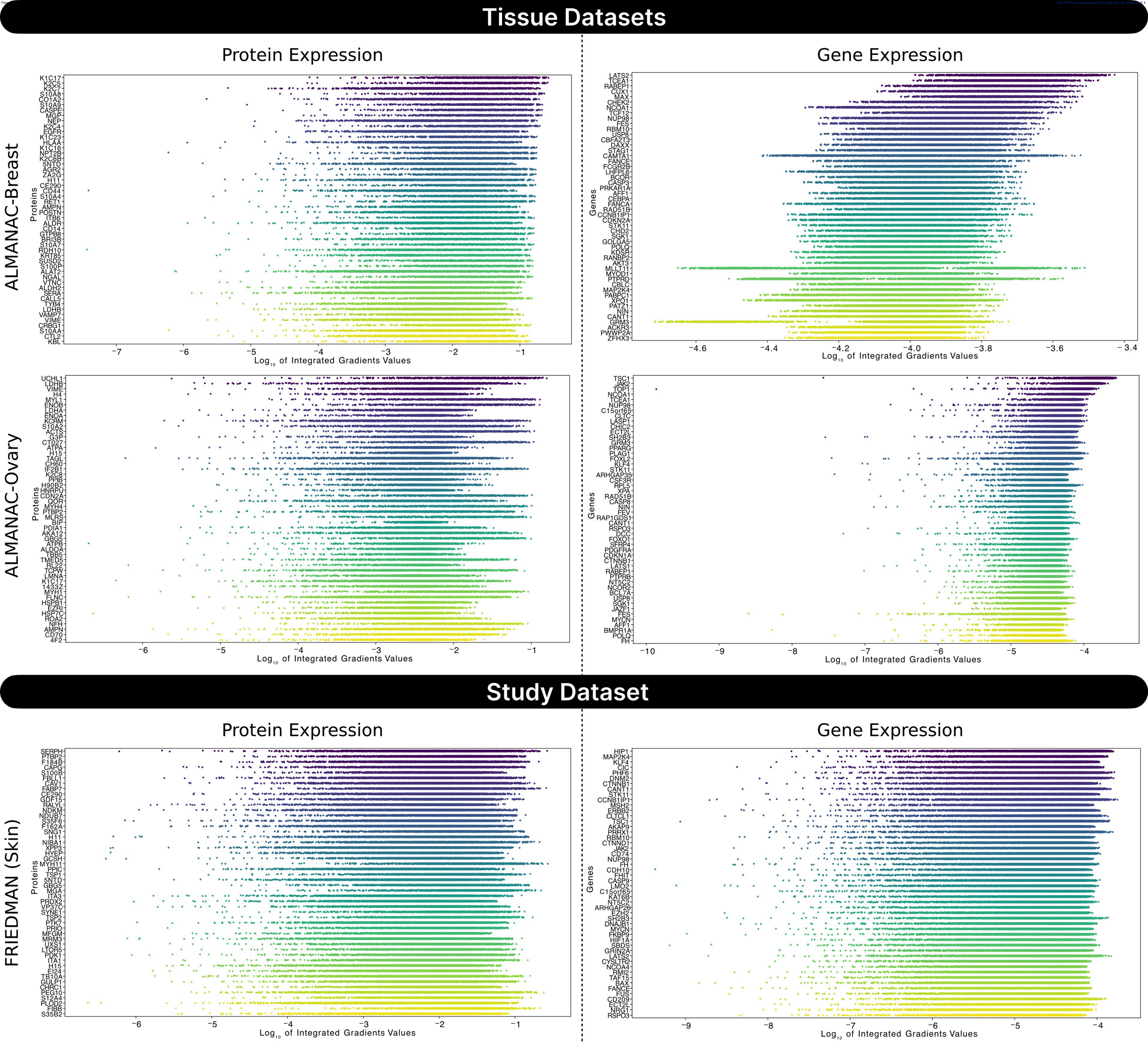
Tissue Datasets
Protein Expression
Gene Expression
ALMANAC-Breast
Proteins
K1C17
K2C5
K2C7
S10A8
CO1A2
S10A9
CASPE
MGP
NEP
K2C4
EGFR
K1C23
HLAA
K1C16
NPT2B
K2C6B
5NTD
AGR2
ZA2G
H11
CE290
CD44
S10A4
RET1
AMPN
POSTN
ITB6
ALDR
CD14
GTPB8
BRI3B
S10A7
RDH10
KRT85
SUSD2
S100P
ALAT2
NGAL
VTNC
ALDH2
SERA
CALL5
TYB4
LDHB
VAMP7
VIME
CRBG1
S10AA
CTL2
KBL
Log10 of Integrated Gradients Values
Genes
LATS2
TCEA1
RABEP1
CUX1
MAX
CHEK2
NCOA1
TCF12
NUP98
FES
RBM10
USP8
CBFA2T3
DAXX
STAG1
CAMTA1
FANCE
FCGR2B
LHFPL6
BCOR
CASP3
PRKAR1A
AFF1
CEBPA
FANCA
RAD51B
CCNB1IP1
CDKN2A
STK11
CHD2
SGK1
GOLGA5
POLQ
KDSR
RANBP2
AKT3
MLLT11
MYOD1
PTPRD
CBLC
MAP2K4
PABPC1
XPO1
PATZ1
NIN
CANT1
GRM3
ACKR3
PWWP2A
ZFHX3
ALMANAC-Ovary
UCHL1
LDHB
VIME
H4
MYL1
ENOB
LDHA
ENOA
KCRM
S10A2
ACTS
G3P
CT027
ATPA
H15
TAGL
CH60
IF2B1
K2C8
PPIB
H90B2
HNRPU
CDN2A
OOR
MYH4
PTBP2
MLRS
BIP
PDIA1
AKA12
GBG5
ATPB
ALDOA
TBB5
TMED5
RL22
TCPW
LMNA
K1C17
1433Z
MYH1
FLNC
HSPB1
EZRI
HSP7C
ROA2
NFH
AMPN
CD70
4F2
TSC1
JAK2
TOP1
NCOA1
TCEA1
NUP98
C15orf65
CLTC
LASP1
CHIC2
ECT2L
SH2B3
GRM3
PPARG
PLAG1
FOXL2
KLF4
STK11
ARHGAP35
CSF3R
RPL5
XPA
RAD51B
CASP8
NIN
FEV
RAP1GDS1
CANT1
RSPO3
DCC
FOXO1
SFRP4
PDGFRA
CDKN1A
CTNNB1
LATS1
RABEP1
PTPRB
NT5C2
NCOR2
BCL7A
USP8
SGK1
JAZF1
FES
MYCN
AFF1
BMPR1A
POLQ
FH
Study Dataset
FRIEDMAN (Skin)
SERPH
PTBP2
F184B
CAPG
S100B
FBLL1
CAV1
FABP7
CE290
GDF15
RALYL
NDKM
NDUB7
S35F6
F162A
SNG1
H11
NIBA1
XPP3
HYEP
GCSH
MYH11
PPIC
TSP1
5NTD
GBG5
MGA
ITA3
PRDX2
VP37C
SYNE1
TSP2
PTK7
PRIO
MFGM
MRM3
UXS1
LTOR5
PDK1
ITA1
H15
EI24
TB10A
GULP1
CHRC1
PEG10
S12A4
PLOD2
FIBB
S35B2
HIP1
MAP2K4
KLF4
CIC
PHF6
DNM2
CTNNB1
CANT1
STK11
CCNB1IP1
MSH2
ERBB2
CLTCL1
TSC1
AKAP9
PRRX1
RBM10
CTNND1
JAK2
CD74
NUP98
FH
CDH10
FHIT
CASP9
LMO2
C15orf65
KAT6B
NT5C2
ARHGAP26
EZH2
SH2B3
DNAJB1
MYCN
FKBP9
HIF1A
SBDS
GRIN2A
LATS2
CYSLTR2
NCOA4
RMI2
TAF15
BAX
FANCE
FUS
CD209
ECT2L
NRG1
RSPO3

Click here to access/download;Figure;fig10_clas_top_features.pdf

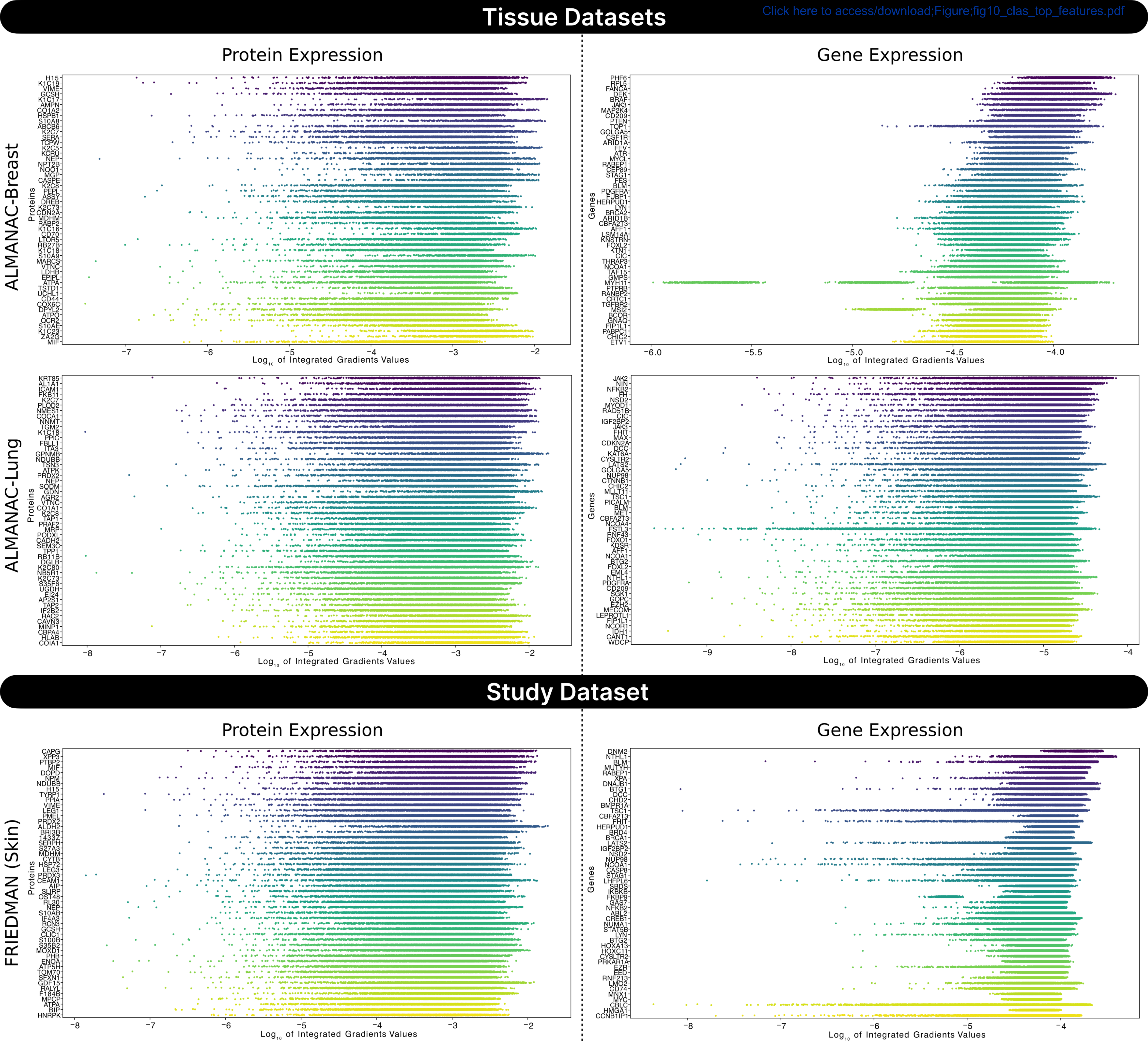

Click here to access/download;Figure;fig11_cold-start_drugcomb.pdf

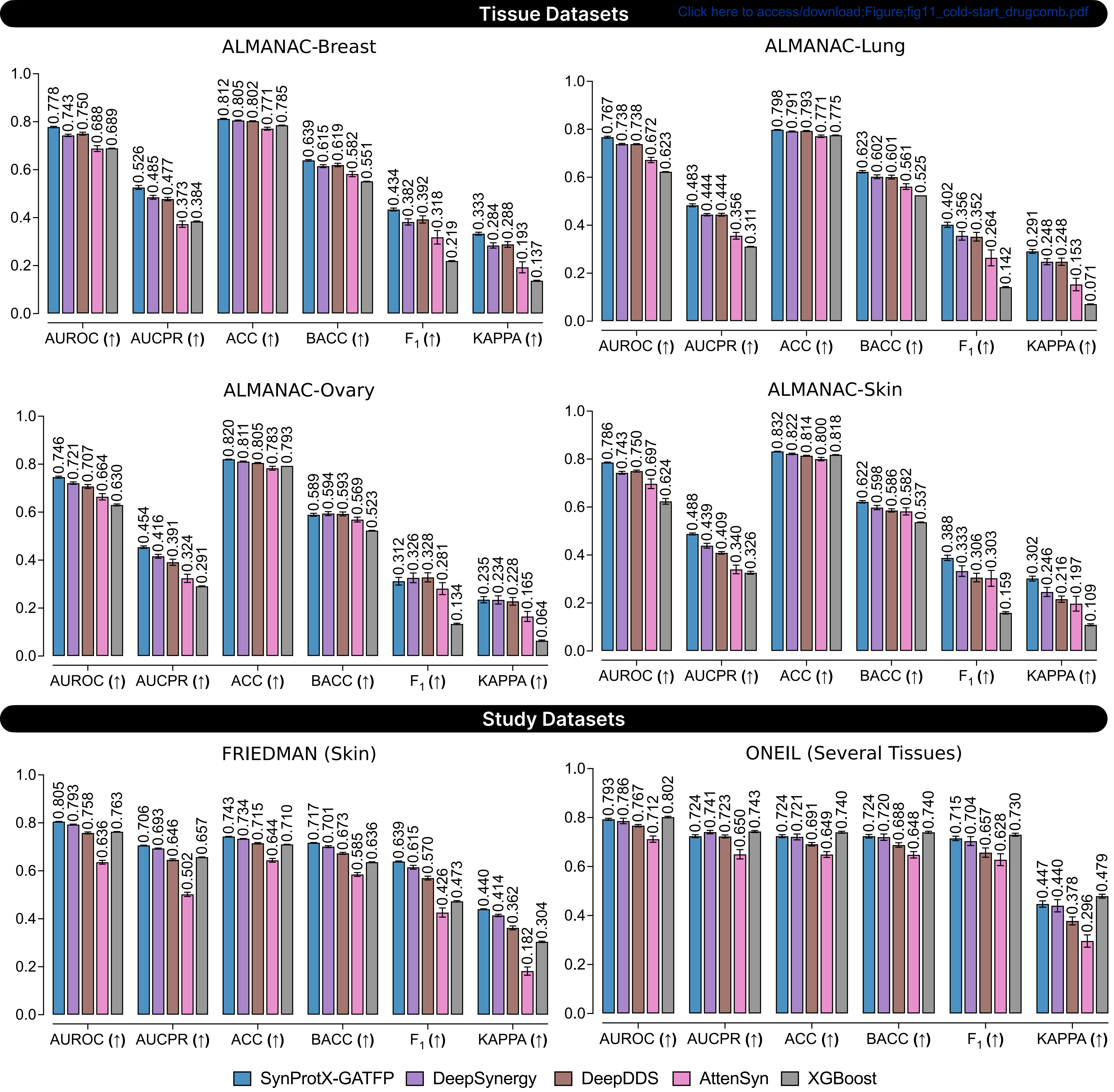

Click here to access/download;Figure;fig12_cold-start_drug.pdf

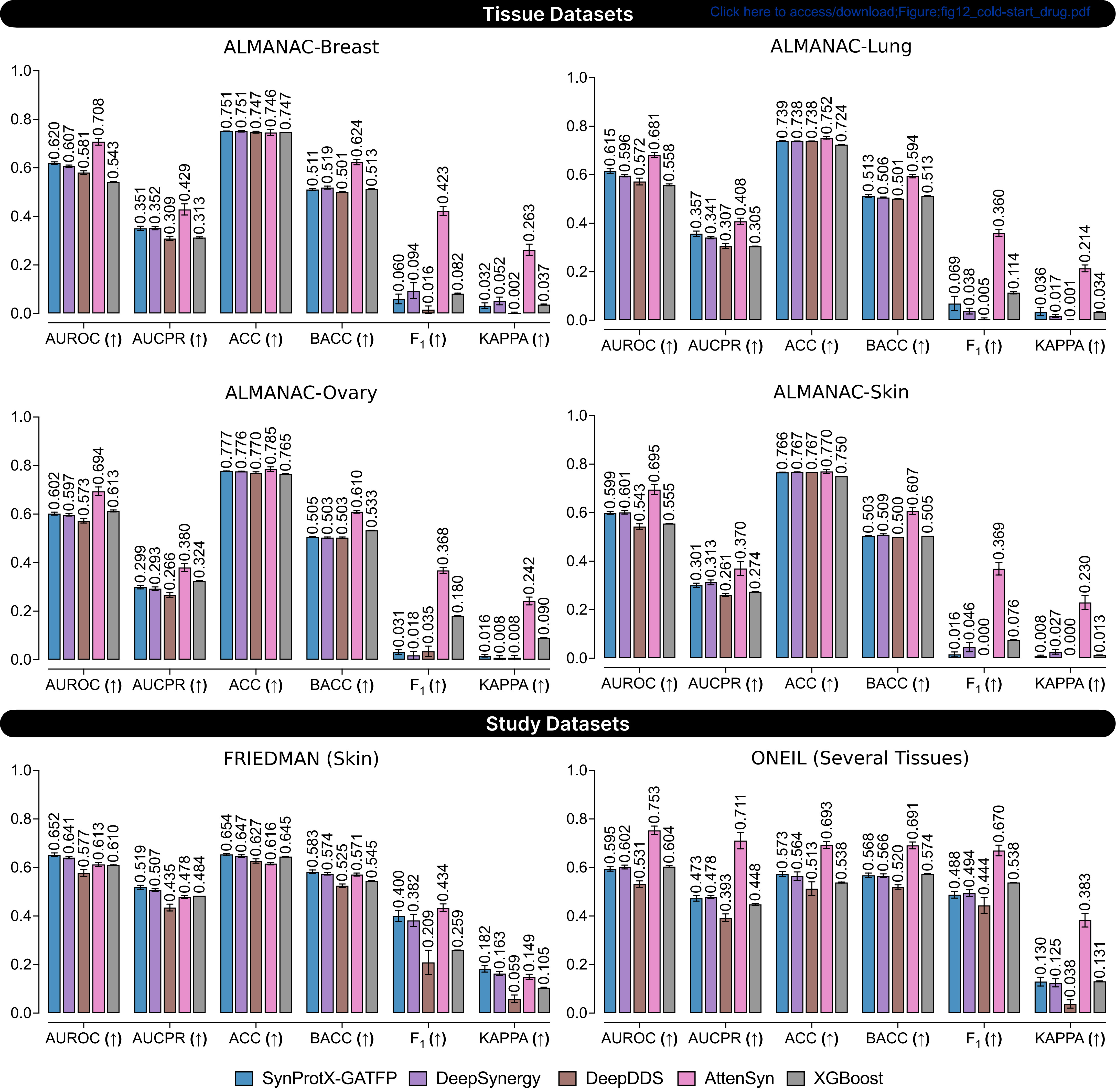

Click here to access/download;Figure;fig13_cold-start_cell.pdf

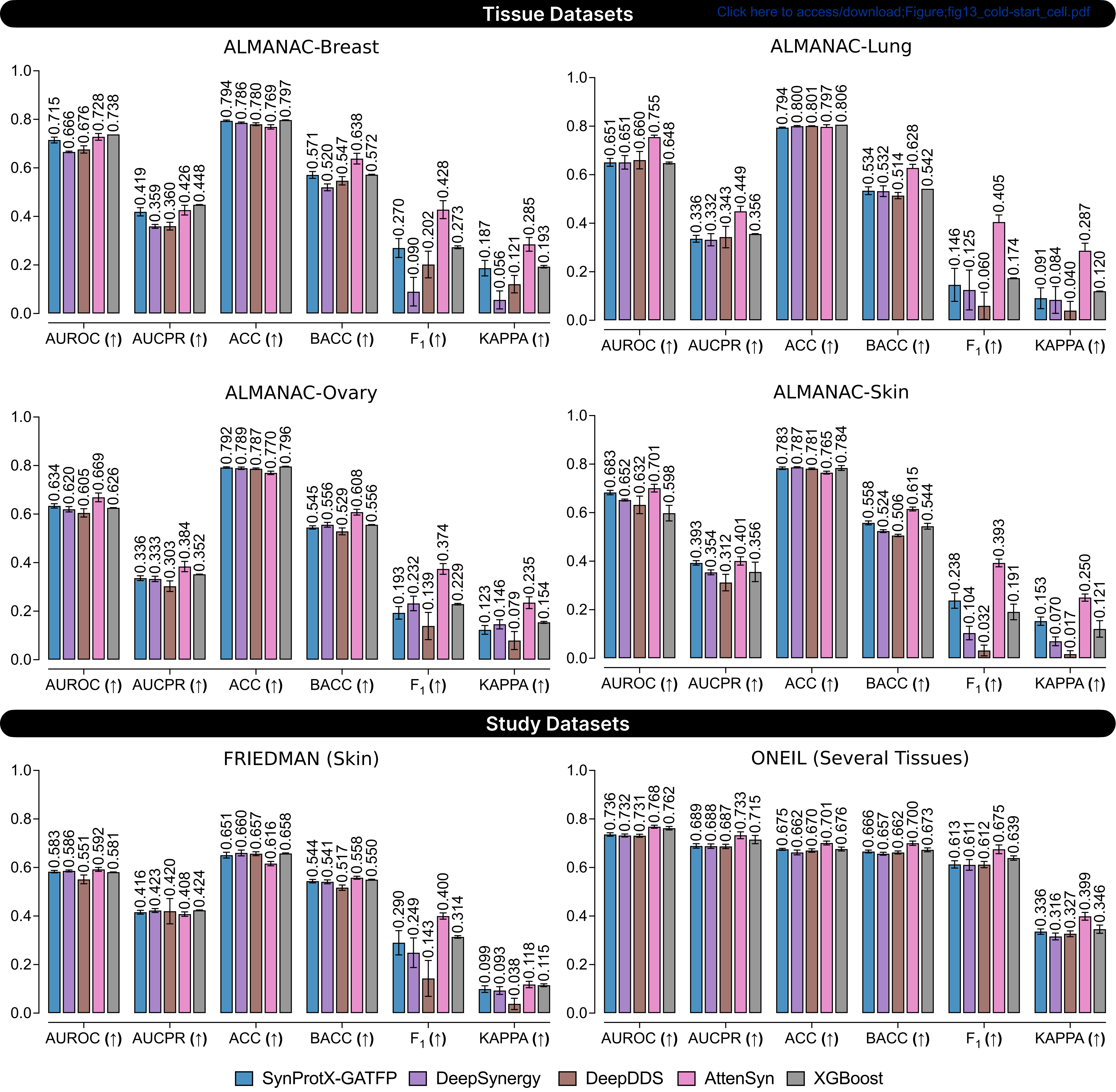

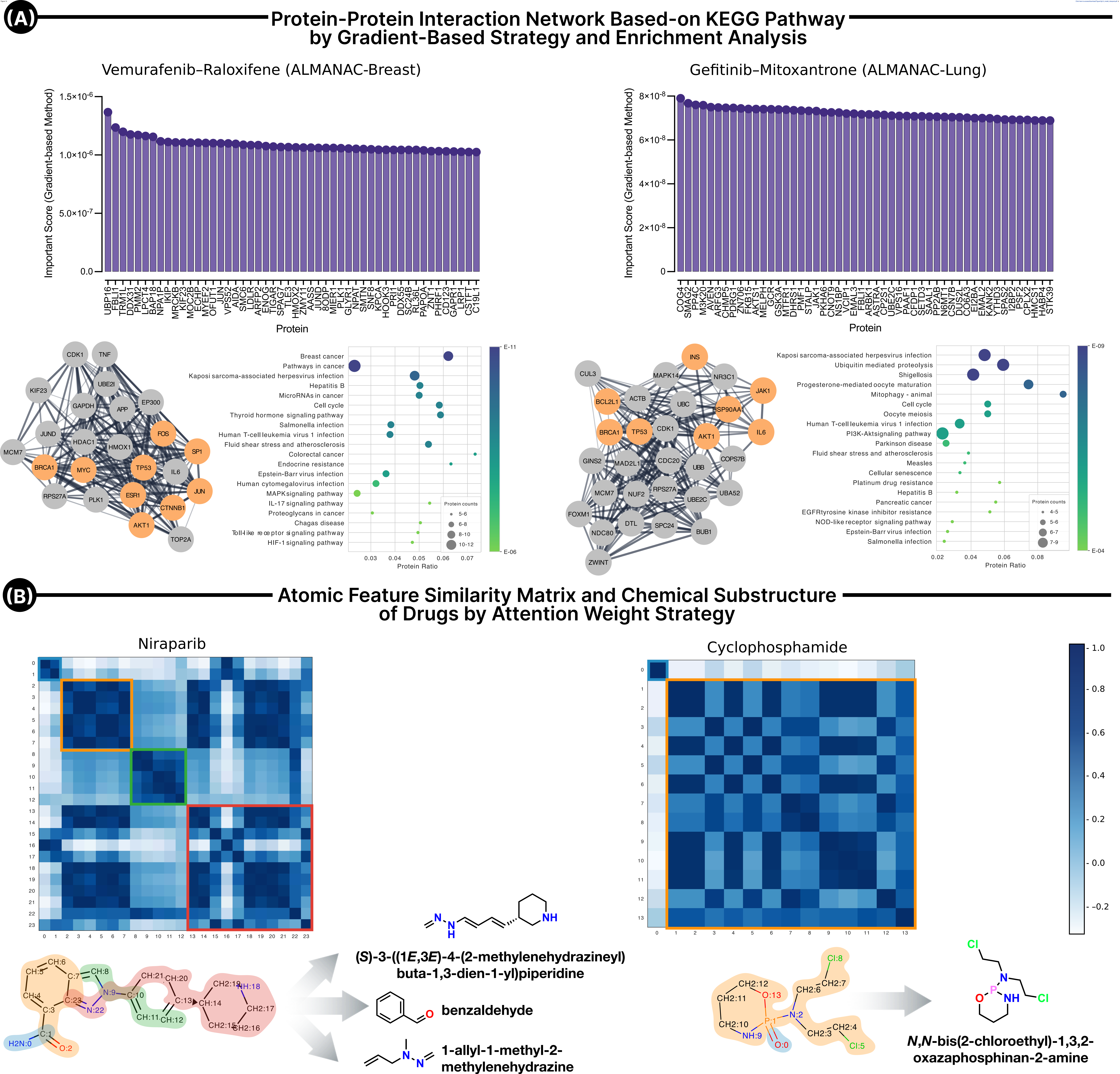
(A)
Protein-Protein Interaction Network Based-on KEGG Pathway by Gradient-Based Strategy and Enrichment Analysis
Vemurafenib–Raloxifene (ALMANAC-Breast)
Gefitinib–Mitoxantrone (ALMANAC-Lung)
Important Score (Gradient-based Method)
Protein
Breast cancer
Pathways in cancer
Kaposi sarcoma-associated herpesvirus infection
Hepatitis B
MicroRNAs in cancer
Cell cycle
Thyroid hormone signaling pathway
Salmonella infection
Human T-cell leukemia virus 1 infection
Fluid shear stress and atherosclerosis
Colorectal cancer
Endocrine resistance
Epstein-Barr virus infection
Human cytomegalovirus infection
MAPK signaling pathway
IL-17 signaling pathway
Proteoglycans in cancer
Chagas disease
Toll-like receptor signaling pathway
HIF-1 signaling pathway
Protein counts
Protein Ratio
Ubiquitin mediated proteolysis
Shigellosis
Progesterone-mediated oocyte maturation
Mitophagy - animal
Oocyte meiosis
PI3K-Akt signaling pathway
Parkinson disease
Measles
Cellular senescence
Platinum drug resistance
Pancreatic cancer
EGFR tyrosine kinase inhibitor resistance
NOD-like receptor signaling pathway
(B)
Atomic Feature Similarity Matrix and Chemical Substructure of Drugs by Attention Weight Strategy
Niraparib
Cyclophosphamide
(S)-3-((1E,3E)-4-(2-methylenehydrazineyl) buta-1,3-dien-1-yl)piperidine
benzaldehyde
1-allyl-1-methyl-2-methylenehydrazine
N,N-bis(2-chloroethyl)-1,3,2-oxazaphosphinan-2-amine

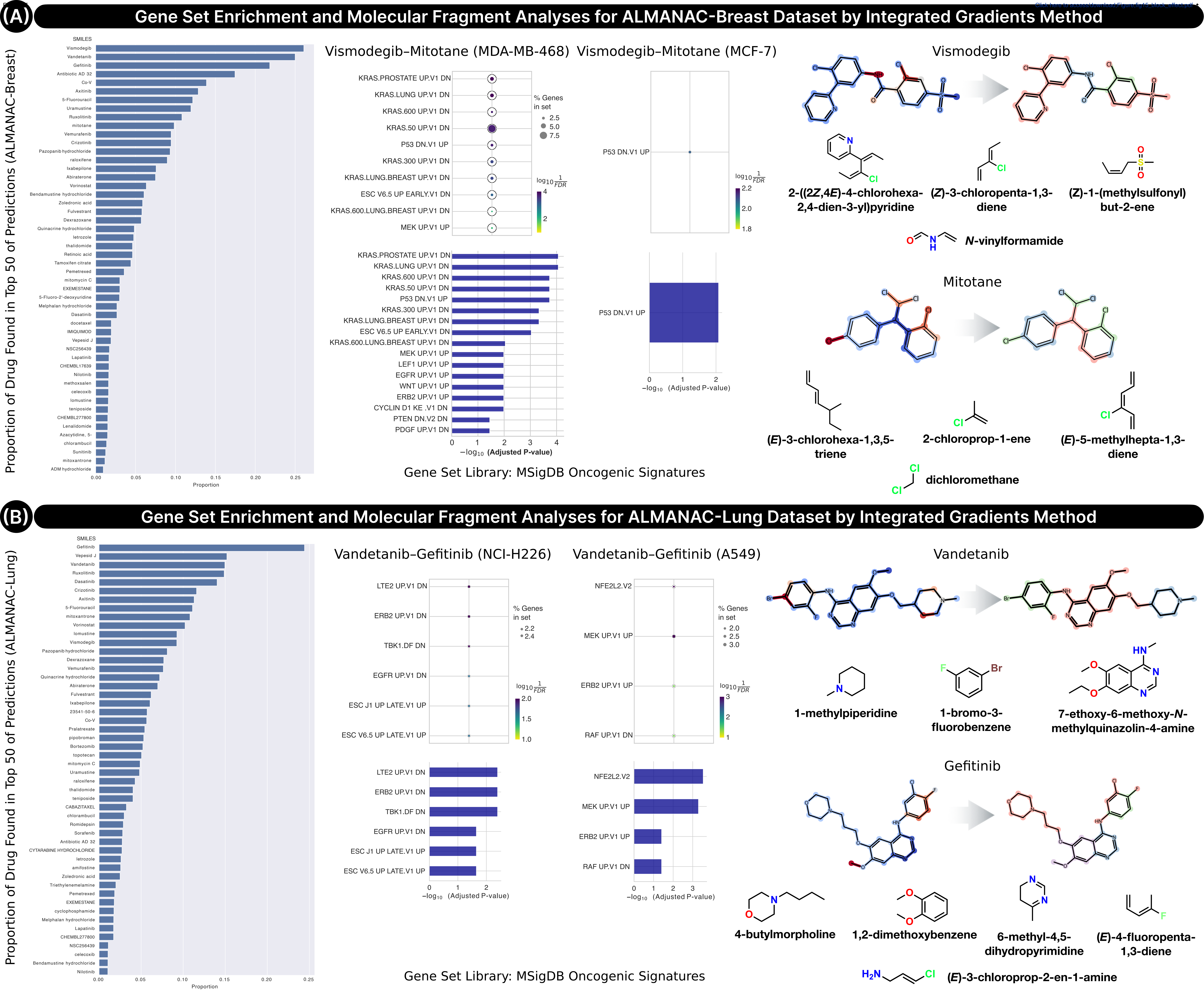
(A)
Gene Set Enrichment and Molecular Fragment Analyses for ALMANAC-Breast Dataset by Integrated Gradients Method
Proportion of Drug Found in Top 50 of Predictions (ALMANAC-Breast)
SMILES
Vismodegib
Vandetanib
Gefitinib
Antibiotic AD 32
Co-V
Axitinib
5-Fluorouracil
Uramustine
Ruxolitinib
mitotane
Vemurafenib
Crizotinib
Pazopanib hydrochloride
raloxifene
Ixabepilone
Abiraterone
Vorinostat
Bendamustine hydrochloride
Zoledronic acid
Fulvestrant
Dexrazoxane
Quinacrine hydrochloride
letrozole
thalidomide
Retinoic acid
Tamoxifen citrate
Pemetrexed
mitomycin C
EXEMESTANE
5-Fluoro-2'-deoxyuridine
Melphalan hydrochloride
Dasatinib
docetaxel
IMIQUIMOD
Vepesid J
NSC256439
Lapatinib
CHEMBL17639
Nilotinib
methoxsalen
celecoxib
lomustine
teniposide
CHEMBL277800
Lenalidomide
Azacytidine, 5-
chlorambucil
Sunitinib
mitoxantrone
ADM hydrochloride
Proportion
Vismodegib–Mitotane (MDA-MB-468)
Vismodegib–Mitotane (MCF-7)
KRAS.PROSTATE UP.V1 DN
KRAS.LUNG UP.V1 DN
KRAS.600 UP.V1 DN
KRAS.50 UP.V1 DN
P53 DN.V1 UP
KRAS.300 UP.V1 DN
KRAS.LUNG.BREAST UP.V1 DN
ESC V6.5 UP EARLY.V1 DN
KRAS.600.LUNG.BREAST UP.V1 DN
MEK UP.V1 UP
LEF1 UP.V1 UP
EGFR UP.V1 UP
WNT UP.V1 UP
ERB2 UP.V1 UP
CYCLIN D1 KE .V1 DN
PTEN DN.V2 DN
PDGF UP.V1 DN
% Genes in set
$\log_{10}\frac{1}{FDR}$
$-\log_{10}$ (Adjusted P-value)
Gene Set Library: MSigDB Oncogenic Signatures
Vismodegib
2-((2Z,4E)-4-chlorohexa-2,4-dien-3-yl)pyridine
(Z)-3-chloropenta-1,3-diene
(Z)-1-(methylsulfonyl)but-2-ene
N-vinylformamide
Mitotane
(E)-3-chlorohexa-1,3,5-triene
2-chloroprop-1-ene
(E)-5-methylhepta-1,3-diene
dichloromethane
(B)
Gene Set Enrichment and Molecular Fragment Analyses for ALMANAC-Lung Dataset by Integrated Gradients Method
Proportion of Drug Found in Top 50 of Predictions (ALMANAC-Lung)
SMILES
Gefitinib
Vepesid J
Vandetanib
Ruxolitinib
Dasatinib
Crizotinib
Axitinib
5-Fluorouracil
mitoxantrone
Vorinostat
lomustine
Vismodegib
Pazopanib hydrochloride
Dexrazoxane
Vemurafenib
Quinacrine hydrochloride
Abiraterone
Fulvestrant
Ixabepilone
23541-50-6
Co-V
Pralatrexate
pipobroman
Bortezomib
topotecan
mitomycin C
Uramustine
raloxifene
thalidomide
teniposide
CABAZITAXEL
chlorambucil
Romidepsin
Sorafenib
Antibiotic AD 32
CYTARABINE HYDROCHLORIDE
letrozole
amifostine
Zoledronic acid
Triethylenemelamine
Pemetrexed
EXEMESTANE
cyclophosphamide
Melphalan hydrochloride
Lapatinib
CHEMBL277800
NSC256439
celecoxib
Bendamustine hydrochloride
Nilotinib
Proportion
Vandetanib–Gefitinib (NCI-H226)
Vandetanib–Gefitinib (A549)
LTE2 UP.V1 DN
ERB2 UP.V1 DN
TBK1.DF DN
EGFR UP.V1 DN
ESC J1 UP LATE.V1 UP
ESC V6.5 UP LATE.V1 UP
NFE2L2.V2
MEK UP.V1 UP
ERB2 UP.V1 UP
RAF UP.V1 DN
% Genes in set
$\log_{10}\frac{1}{FDR}$
$-\log_{10}$ (Adjusted P-value)
Gene Set Library: MSigDB Oncogenic Signatures
Vandetanib
1-methylpiperidine
1-bromo-3-fluorobenzene
7-ethoxy-6-methoxy-N-methylquinazolin-4-amine
Gefitinib
4-butylmorpholine
1,2-dimethoxybenzene
6-methyl-4,5-dihydropyrimidine
(E)-4-fluoropenta-1,3-diene
(E)-3-chloroprop-2-en-1-amine

Click here to access/download
Supplementary Material
SynProtX_supp_V7.pdf

Personal Cover

Click here to access/download;Personal Cover;cover-rebuttal_letter_revised_ggs.pdf

School of Information Science and Technology (IST)
Vidyasirimedhi Institute of Science and Technology (VISTEC)
555 Moo 1, Payupnai, Wangchan, Rayong 21210, Thailand
Tel: +66 (0) 33-01 4444 | Fax: +66 (0) 33-01 4445
www.vistec.ac.th

May 12, 2025

To
Editor-in-Chief
GigaScience

Subject: Revised Manuscript Submission – Response to Reviewers' Comments on "SynProtX: A Large-Scale Proteomics-Based Deep Learning Model for Predicting Synergistic Anticancer Drug Combinations"

Dear Editor-in-Chief,

We sincerely appreciate the time and effort that you, GigaDB team, and the reviewers have dedicated to evaluating our manuscript and relevant datasets titled "**SynProtX: A Large-Scale Proteomics-Based Deep Learning Model for Predicting Synergistic Anticancer Drug Combinations**" We have carefully considered the constructive feedback provided, and we are grateful for the opportunity to revise and resubmit our work.

While we acknowledge that Reviewer #2 did not recommend acceptance in the current form, we believe that the substantial revisions we have undertaken—guided by both Reviewer #1's major comments and Reviewer #2's critical feedback—have significantly improved the quality, clarity, and scientific rigor of the manuscript.

Major Revisions and Rebuttals:

1. Novelty and Unique Contributions (Reviewer #1 and #2): We have thoroughly revised the Background section to explicitly highlight the unique contributions of SynProtX compared to existing models. Unlike prior studies that rely predominantly on transcriptomics, SynProtX integrates large-scale proteomics data (ProCan-DepMapSanger), offering a functional and mechanistic perspective on drug synergy. This aspect has been underexplored in previous works and represents a key innovation of our study. The revised sections now emphasize this contribution, supported by new references and comparative ablation studies.

2. Generalizability and Dataset Heterogeneity (Reviewer #2): To address concerns about generalizability, we expanded our validation to include leave-drug-combination-out, leave-drug-out, and leave-cell-line-out strategies, across tissue-specific and cross-study datasets. We clarified why utilizing the entire heterogeneous DrugComb dataset might lead to unreliable conclusions due to inconsistencies in dose-response matrix sizes and cell line redundancy. This addresses Reviewer #2's concerns about limited generalizability.

3. Feature Importance and Mechanistic Insights (Reviewer #1 and #2): We conducted a comprehensive integrated gradients analysis to systematically evaluate feature importance. Detailed results are now included in revised manuscript. Furthermore, we performed pathway enrichment (GSEA) and molecular fragment analyses to elucidate cell-specific mechanisms behind block effects. This directly responds to Reviewer #1's request for mechanistic interpretation and Reviewer #2's critique regarding insufficient novelty.

School of Information Science and Technology (IST)
Vidyasirimedhi Institute of Science and Technology (VISTEC)
555 Moo 1, Payupnai, Wangchan, Rayong 21210, Thailand
Tel: +66 (0) 33-01 4444 | Fax: +66 (0) 33-01 4445
www.vistec.ac.th

4. Block Effects Analysis (Reviewer #1): We expanded the analysis of block effects, where some drugs in combinations consistently dominate and drive the synergistic effect, leading to potential bias in synergy predictions. We performed integrated gradients to assess the contribution of individual protein features, focusing on the most influential features identified through ablation studies. Notably, we investigated combinations such as vismodegib–mitotane and vandetanib–gefitinib across various cancer cell lines (e.g., MDA-MB-468, MCF-7, NCI-H226, A549). The results highlight that these combinations exhibit significant block effects, driven by specific drugs consistently enhancing synergy across multiple cell lines.

We further elucidated the biological relevance of these effects by conducting pathway-enrichment analysis (GSEA) and molecular fragment analysis, revealing that key signaling pathways such as EGFR and MAPK were significantly enriched in the most influential proteins. This deeper mechanistic understanding goes beyond simple correlation and provides novel insights into the key biological mechanisms responsible for drug synergy.

5. Cold-Start Experiment (Reviewer #1 and #2): To assess the generalizability of SynProtX, we implemented cold-start experiments, including leave-drug-combination-out, leave-drug-out, and leave-cell-line-out strategies. These experiments help evaluate how the model performs when it encounters unseen data (such as new drugs, new cell lines, or novel combinations). The cold-start evaluations show that SynProtX maintains strong performance even when applied to unseen drug combinations, which is typical challenges in real-world clinical settings. This ability to generalize in cold-start scenarios is an important feature of the model's robustness, especially for predicting drug synergy in unknown clinical settings.

In the revised manuscript, we have added a dedicated section titled "SynProtX-GATFP Demonstrates Generalization Capability on Cold-Start Scenarios", where we emphasize that SynProtX can effectively handle novel, previously unseen data. This section also discusses the strengths and limitations of the cold-start methods employed.

6. Cell-Specific Response Evaluation (Reviewer #1 and #2): We have added a dedicated section, "SynProtX-GATFP Affirms the Performance on Cell-specific Responses", showcasing how SynProtX accurately predicts drug synergy across individual cancer cell lines. These results demonstrate the model's robustness in personalized medicine applications.

7. Addressing Model Performance Variability (Reviewer #2): We acknowledged the AUROC variability in the O'Neil dataset and provided a comparative analysis with ensemble methods like XGBoost. This nuanced discussion reflects a balanced interpretation of model performance across datasets, as suggested by Reviewer #2.

8. Limitations and Future Directions: We added a Limitations of the Study section discussing generalizability challenges in cold-start scenarios and dataset scale. Additionally, the Potential Implications section outlines how SynProtX could be extended to other drug discovery applications such as drug toxicity prediction and biomarker discovery.

VISTEC
VIDYASIRIMEDHI
INSTITUTE OF SCIENCE AND TECHNOLOGY

School of Information Science and Technology (IST)
Vidyasirimedhi Institute of Science and Technology (VISTEC)
555 Moo 1, Payupnai, Wangchan, Rayong 21210, Thailand
Tel: +66 (0) 33-01 4444 | Fax: +66 (0) 33-01 4445
www.vistec.ac.th

We are confident that these comprehensive revisions and additional analyses have significantly strengthened the manuscript, addressing the reviewers' concerns in depth. We remain committed to the principles of Open Science, ensuring full transparency and reproducibility through publicly available code and data.

We respectfully request your reconsideration of our manuscript for publication in ***GigaScience***.

Thank you once again for your thoughtful feedback and the opportunity to improve our work. We look forward to your favorable response.

Sincerely Yours,

Bundit Boonyarit
Ph.D. candidate
School of Information Science and Technology, VISTEC
Email: bundit.b_s18@vistec.ac.th

 Click here to access/download;Personal Cover;GGS_Authors_Responses.pdf

Author's Responses to Reviewers

(The sections that have been revised are highlighted in yellow in the main manuscript.)

Reviewer #1:

Synergistic drug combination prediction is important for disease treatment. However, it remains an open problem to identify the effective drug combinations in real clinical treatments. Computational models that can predict the drug combinations by integrating chemical and omic features can be helpful for the drug combination research. In this study, the authors proposed a deep learning model for drug combination prediction. Here are some comments might be helpful.

Author's response:

We sincerely thank ***Reviewer #1*** for their thoughtful and constructive feedback. Your comments have been invaluable in refining the manuscript and improving the clarity of our findings. We have carefully addressed each of your concerns, and below, we provide detailed responses to your points. We believe the revisions have significantly strengthened our work, and we appreciate your contribution to enhancing the manuscript.

1. The model design innovation is limited and also comparison results showed limited improvements of the proposed method than other existing models. So it is important to clearly indicate the unique contributions of the proposed model than existing models.

Author's response:

We understand the concern regarding the novelty of our model design. The primary innovation in our study lies in the explicit integration of large-scale proteomics data alongside molecular graphs and molecular fingerprints. This large-scale proteomics data with the slightly modified approach has not been extensively explored in the anticancer drug combination prediction. Our model, SynProtX, incorporates multi-omics data (proteomics, transcriptomics, and drug molecular structure) to predict drug synergy. This comprehensive methodology has demonstrated improvements in predictive performance compared to existing state-of-the-art models, especially

in the ablation study – w/o protein level data can significantly drop predictive performance when compared to w/o gene expression data. We have enhanced the ***Background*** section (***Lines 87–159***) to emphasize how current models primarily rely on genomic or transcriptomic data, which limits their ability to fully capture the functional states of cells. By explicitly integrating proteomics data, we improve the accuracy of synergy predictions by providing a more holistic view of the cellular environment. Furthermore, we stated the research question**: Can large-scale proteomics data, combined with molecular graphs and fingerprints, improve model performance compared to traditional gene expression data alone?** Additionally, we included the research evidence of "DeePathNet", a transformer-based deep learning model for cancer monotherapy drug response prediction. DeePathNet integrates protein expression data from the **ProCan-DepMapSanger** database, demonstrating the value of incorporating proteomics for improving predictive accuracy.

Additionally, we evaluated the **O'Neil (ONEIL) dataset** as a small-scale dataset to observe the model's ability to handle smaller, multi-tissue data scenarios. This evaluation is important for understanding how SynProtX performs on more limited datasets. We found that while SynProtX performed well across tissue-specific datasets, the ONEIL dataset revealed some interesting phenomena regarding the model's adaptability to smaller-scale data, which are discussed in the ***Results and Discussion*** section.

Furthermore, we have added a ***Limitations of the Study*** section (***Lines 759–791***) that discusses the constraints of SynProtX model, such as model's ability to generalize to unseen data, particularly in cold-start scenarios, and model overfitting in ONEIL dataset in small-scaled dataset and cell lines span multiple tissue types.

In the ***Potential Implications*** section (***Lines 793–820***), we discuss how the incorporation of proteomics data in SynProtX has important implications for personalized medicine to other diseases, drug toxicity prediction, and cancer biomarker discovery. The ability to predict drug synergy with a more comprehensive dataset can lead to more precise treatment strategies, offering new avenues for improving therapy outcomes.

---

2. From a large number of features, it remains challenging to identify the important features contributing to the model prediction. Without systematic evaluation, it is not sound to conclude the importance of the identified features. So it is important to systematically evaluate the importance/score distributions of the all the features/the top-ranked features.

Author's response:

We agree that a more systematic evaluation of feature importance is necessary. To address this, we have conducted a comprehensive feature importance analysis using integrated gradients (IG) (***Figures 9 and 10***). This allows us to rank and evaluate the contribution of each feature (including protein expression and gene expression) to the model's predictions. The revised manuscript now includes a detailed description of these results in "***Protein Level Improves Model Predictive Performance for SynProtX-GATFP***" section (***Lines 470–578***). Here, we provide insights into how individual features influence the model's performance across different datasets. Specifically, we found that protein expression plays a significant role in improving the model's predictive accuracy.

In addition, we have included research evidence supporting the top-ranked proteins based on their involvement in biochemical pathways specific to the tissue types in the datasets. This highlights how protein-level features can contribute to understanding the mechanisms driving drug synergy. The methodology of IG is elaborated further in the "***Model Interpretability***" section (***Lines 1021–1073***). Moreover, this section describes the attention weight strategy used to support the results presented in ***Figure 14***. This attention mechanism enhances the interpretability of the model, allowing us to identify the most influential chemical substructure features in drug synergy predictions.

---

3. There are some 'block effects' of some drugs, which means that some drugs always works better than other drugs. Are these features associated with these drugs? If yes, not much meaningful or informative as it is just a simple correlation. It is important to identify the key mechanisms of cell-specific response drugs.

Author's response:

We acknowledge that the "block effects" observed in some drug combinations need to be explored in greater depth. In response, we have expanded the analysis to include pathway-enrichment analyses (GSEA) and molecular fragment analysis in the section "***Integrated Gradients (IG) Method of SynProtX-GATFP Explains Block Effects***" (***Lines 697–758 and Supplementary Tables S17 and S18***). For the methodology, we applied IG to assess the contribution of individual protein features, focusing on the most influential features identified through ablation studies, to the drug combination synergy prediction. Specifically, we focused on combinations of top-proportion drugs found in the test set, including **vismodegib–mitotane** via the **MDA-MB-468** and **MCF-7** cell lines of the ALMANAC-Breast dataset, and **vandetanib–gefitinib** via the **NCI-H226** and **A549** cell lines of the ALMANAC-Lung dataset.

The results of these analyses show that vismodegib–mitotane and vandetanib–gefitinib exhibit consistent block effects across these cell lines, suggesting that certain drugs in the combinations play dominant roles in driving synergy. The pathway-enrichment analysis further revealed that specific signaling pathways, such as EGFR and MAPK pathways, were significantly enriched in the most influential proteins. This deeper mechanistic understanding goes beyond simple correlation, providing novel insights into the key biological mechanisms responsible for drug synergy.

---

4. It is important to evaluate the model performance on cell-specific.

Author's response:

We have now included an evaluation of the model's performance on cell-specific data, which was not adequately addressed in the initial version. In the revised manuscript, we have dedicated the section titled ***"SynProtX-GATFP Affirms the Performance on Cell-specific Responses"*** (***Lines 406-469***) to thoroughly describe the model's performance on individual cell lines within specific cancer types. The evaluation of cell-specific responses is critical for assessing how well SynProtX can predict drug synergy within different cancer cell lines, which may vary in their drug response profiles.

This evaluation is supported by ***Supplementary Tables S4–S15***, which provide detailed performance metrics across cell lines for different datasets such as ALMANAC-Breast, ALMANAC-Lung, ALMANAC-Ovary, ALMANAC-Skin, FRIEDMAN, and ONEIL for regression and classification tasks. The results demonstrate that SynProtX is capable of making highly accurate predictions for individual cell lines, accounting for their unique molecular and proteomic profiles. The ability to evaluate cell-specific data is a major strength of our model, as it enhances the precision and relevance of predictions for personalized cancer treatments.

We sincerly thank ***Reviwer #1*** for their invaluable suggestion to evaluate the model performance on cell-specific. We agree that this is an important aspect to consider, as different cell lines may exhibit distinct drug response profiles. By evaluating SynProtX on cell-specific data, we can better understand how the model performs across various biological contexts and ensure that it captures the unique molecular and proteomic characteristics of each cell type. This approach significantly enhances the model's ability to predict drug synergy in a more personalized manner

---

5. It is important to use 'cold-start' (remove all the information of given cells or given drugs from the training data to evaluate the model performance).

Author's response:

To address concerns about generalizability, we have implemented cold-start evaluation methods, including leave-drug-combination-out, leave-drug-out, and leave-cell-line-out strategies, to assess the generalizability of our model on unseen data (***Figures 11–13***). We would like to note that the leave-drug-combination-out result was included in the original manuscript.

The results of these cold-start validations are now presented in the section "***SynProtX-GATFP Demonstrates Generalization Capability on Cold-start Scenarios***" (***Lines 579–622***), demonstrating the model's ability to perform well even with previously unseen drug combinations and cell lines. This is discussed in the revised manuscript, where we emphasize the robustness and applicability of our model in clinical settings. However, we address the constraints of both leave-drug-out and leave-cell-line-out strategies.

Leave-Drug-Combination-Out: This strategy involves excluding drug combinations from the training data and evaluating the model's ability to predict drug synergy for these unseen combinations. The strength of this method is that it provides a more robust evaluation of the model's ability to generalize across different drug interactions, which is critical in drug synergy prediction. It ensures that the model can predict combinations it has never seen before, making it highly relevant for clinical scenarios where new drug combinations are constantly being explored. The strength of this method lies in its ability to simulate real-world applications where new drug combinations are tested.

Leave-Drug-Out: In this strategy, one drug is excluded from the training data, and the model's performance is evaluated based on the prediction of drug synergy for combinations involving these unseen drugs. The constraint of this method lies in the fact that the model may have limited exposure to the drug's specific interactions with other drugs, which may affect prediction accuracy for novel drug combinations. This strategy is essential for evaluating the model's ability to generalize to unseen drugs.

Leave-Cell-Line-Out: Here, the training data is split by excluding specific cell lines, and the model is tested on its ability to predict drug synergy in these cell lines. The constraint of this method is that it may reduce the representativeness of the training data, as the model is not exposed to the specific molecular and phenotypic characteristics of the excluded cell lines during training. This may result in reduced prediction accuracy for cell lines with distinct molecular profiles, especially if these cell lines significantly differ from those in the training set.

Reviewer #2:

In this paper, the authors propose a drug combination synergy prediction model using molecular graphs, molecular fingerprints, gene expression profiles, and proteomics information. The model is evaluated on five single cancer type sub-datasets of the DrugComb dataset instead of utilizing the full dataset, which limits generalizability. The use of DNN architectures and graph neural networks are not innovative, given that similar methods (e.g. DeepDDS) have already been widely explored. Although the proposed model outperforms the compared baselines, it is unclear whether baselines are strong enough.

Due to the poor performance (low AUROC), lack of generalizability, and insufficient novelty, I do not recommend acceptance of this manuscript in its current form.

Author's response:

We thank ***Reviewer #2*** for their comments. Your feedback has been incredibly valuable in helping us refine the manuscript, particularly with regard to the generalizability of our approach. We have carefully considered each of your points and have made several revisions to address the concerns raised. **In the following, we provide detailed responses to your comments, along with our rebuttal and clarifications.** We believe these changes not only address your concerns but also contribute to enhancing the clarity and impact of our findings.

Generalizability Across Datasets:
We understand your concern regarding the generalizability of our model, especially since our initial evaluation was focused on single cancer-type datasets. **To better evaluate the generalizability of SynProtX, we have employed leave-one-out validation (cold-start experiment)**, which includes **leave-drug-combination-out**, **leave-drug-out**, and **leave-cell-line-out** strategies across both tissue-specific (ALMANAC-Breast, ALMANAC-Lung, ALMANAC-Ovary, ALMANAC-Skin) and study datasets (ONEIL and FRIEDMAN). This expanded validation approach allows us to demonstrate that SynProtX generalizes effectively across different biological contexts, making it a more robust model for drug synergy prediction. This

highlight is presented in the section of "***SynProtX-GATFP Demonstrates Generalization Capability on Cold-start Scenarios***" (***Lines 579–622***).

Furthermore, we need to clarify that using the full DrugComb dataset could introduce challenges that may affect model reliability due to data quality and heterogeneity. Each sub-dataset in the **DrugComb collection comes from different experiments, and the dose-response matrix size** (***Supplementary Table S2***) varies between them, which can lead to **inconsistent data distribution** across the full dataset. This variability is an important factor to consider when evaluating model performance. Specifically, the subsets of the DrugComb dataset represent data collected from different experimental protocols and data collection methodologies, which can lead to significant variations in **data quality and completeness**. These discrepancies introduce **heterogeneity**, making it difficult to train a single model on the entire dataset **due to the inconsistency in experimental conditions**. Another important issue arises from **cell line redundancy within each sub-dataset**. Many sub-datasets, such as those from the ALMANAC-Skin or FRIDEMAN collections, may contain repeated use of the same cell lines in multiple drug combinations. **This redundancy can lead to overfitting and an unfair or biased evaluation**, as the model may inadvertently learn patterns from **overrepresented cell lines**. In such cases, the model becomes highly specialized in recognizing these familiar patterns, which limits its ability to generalize effectively to novel cell lines or drug combinations that were underrepresented or entirely absent in the training data.

Given these challenges, <u>we chose to evaluate SynProtX on tissue-specific and study sub-datasets rather than the full DrugComb dataset</u>. This allows us to focus on **more homogeneous data, thereby mitigating issues arising from cell line redundancy, experimental variability, and dose-response matrix size variability**. By using focused sub-datasets, we ensure that the evaluation is based on **consistent** and **uniform data**, leading to more **accurate and reliable predictions**.

Comparison with Existing Models & Novelty:

We appreciate your comments on the novelty of our approach, particularly given that models like DeepDDS and DeepSynergy have already been widely explored. While these models have

significantly contributed to the field, **we believe that our approach represents a substantial advancement due to its integration of large-scale proteomics data** alongside **molecular graphs** and **fingerprints**. The hypothesis guiding this study was: **"Can large-scale proteomics data, combined with molecular graphs and fingerprints, improve model performance compared to traditional gene expression data alone?"** (***Lines 100–125*****).** This multi-omics approach offers a more comprehensive understanding of drug synergy by providing a holistic view of the cellular environment, which ultimately improves predictive accuracy. Furthermore, we are pleased to submit this manuscript to ***GigaScience***, a prominent journal in the field of **big data-driven life sciences applications**, as we believe our work aligns with the journal's focus on computational approaches for solving complex biological problems.

To clearly demonstrate the performance of SynProtX, we have added a more detailed comparison with existing models in ***Figures 3 and 4*** and ***Supplementary Tables S2 and S3***. These comparisons highlight SynProtX's high performance in both regression and classification tasks. Additionally, the ***Results and Discussion*** sections (***Lines 309–365, 406–469, and 470–578***) further demonstrate the advantages of our approach in comparison to other baseline models.

We expanded the ***Background*** section (***Lines 87–125***) to address the limitations of current models, such as their reliance on genomic data alone, which often fails to capture the complexity of drug interactions. Our multi-omics approach, particularly the inclusion of proteomics data, overcomes these limitations, leading to a more accurate prediction of drug synergy.

Model Performance & Cell-Specific Response Evaluation:

We appreciate your comment on the AUROC score and agree that the performance across different datasets warrants further clarification. While SynProtX performs well on several tissue-specific datasets, we observed some variability in the O'Neil (ONEIL) dataset (expanded experiment in revised version), where it was marginally outperformed by XGBoost. We have now included a detailed discussion of these, where we suggest that ensemble-based methods like XGBoost may outperform individual deep learning models in certain scenarios, particularly with smaller or more heterogeneous datasets.

In addition, we expanded the experiment on **cell-specific responses** to provide a more thorough analysis of how SynProtX performs across different cancer cell lines. In the revised manuscript, we present a dedicated section titled **“*SynProtX-GATFP Affirms the Performance on Cell-Specific Responses*”** (***Lines 406–469***), where we evaluate the model’s performance on specific cancer cell lines within the ALMANAC-Breast, ALMANAC-Lung, ALMANAC-Ovary, ALMANAC-Skin, FRIEDMAN, and ONEIL datasets. The results demonstrate that SynProtX is capable of making highly accurate predictions for individual cell lines, accounting for their unique molecular and proteomic profiles. The ability to evaluate cell-specific data is a major strength of our model, as it enhances the precision and relevance of predictions for personalized cancer treatments.

We investigated block effects in drug combinations in section of “***Integrated Gradients (IG) Method of SynProtX-GATFP Explains Block Effects***” (***Lines: 697–758***). Block effects occur when one drug in a combination dominates the synergistic effect, causing the synergy to be primarily driven by this drug across multiple cell lines. To identify these block effects, we applied IG to assess the contribution of individual protein features, focusing on the most influential features, to the drug combination synergy prediction. The IG method is particularly useful for identifying the most influential proteins for predicting drug synergy, especially in cell-specific contexts. Our results show that SynProtX performs robustly across different cancer cell lines, accurately identifying drug synergy patterns that are unique to each cancer type. This was particularly evident in the ALMANAC-Breast and ALMANAC-Lung datasets, where we observed consistent predictive accuracy. These findings demonstrate that SynProtX is capable of capturing cell-specific drug synergy by accounting for the unique molecular and proteomic characteristics of each cell line. This ability to tailor predictions based on the specific features of individual cell lines underscores the model’s potential for personalized drug combination strategies, which is one of the key strengths of SynProtX.

Furthermore, we have conducted an ablation study titled **“*Protein Level Improves Model Predictive Performance for SynProtX-GATFP*”** (***Lines 470–578***). This ablation study demonstrates the significant impact of protein-level data on model performance. By isolating the influence of protein-level features, we show that including proteomics data substantially improves

the model's predictive accuracy compared to models relying solely on gene expression data. This study provides strong evidence supporting the importance of protein data for predicting drug synergy.

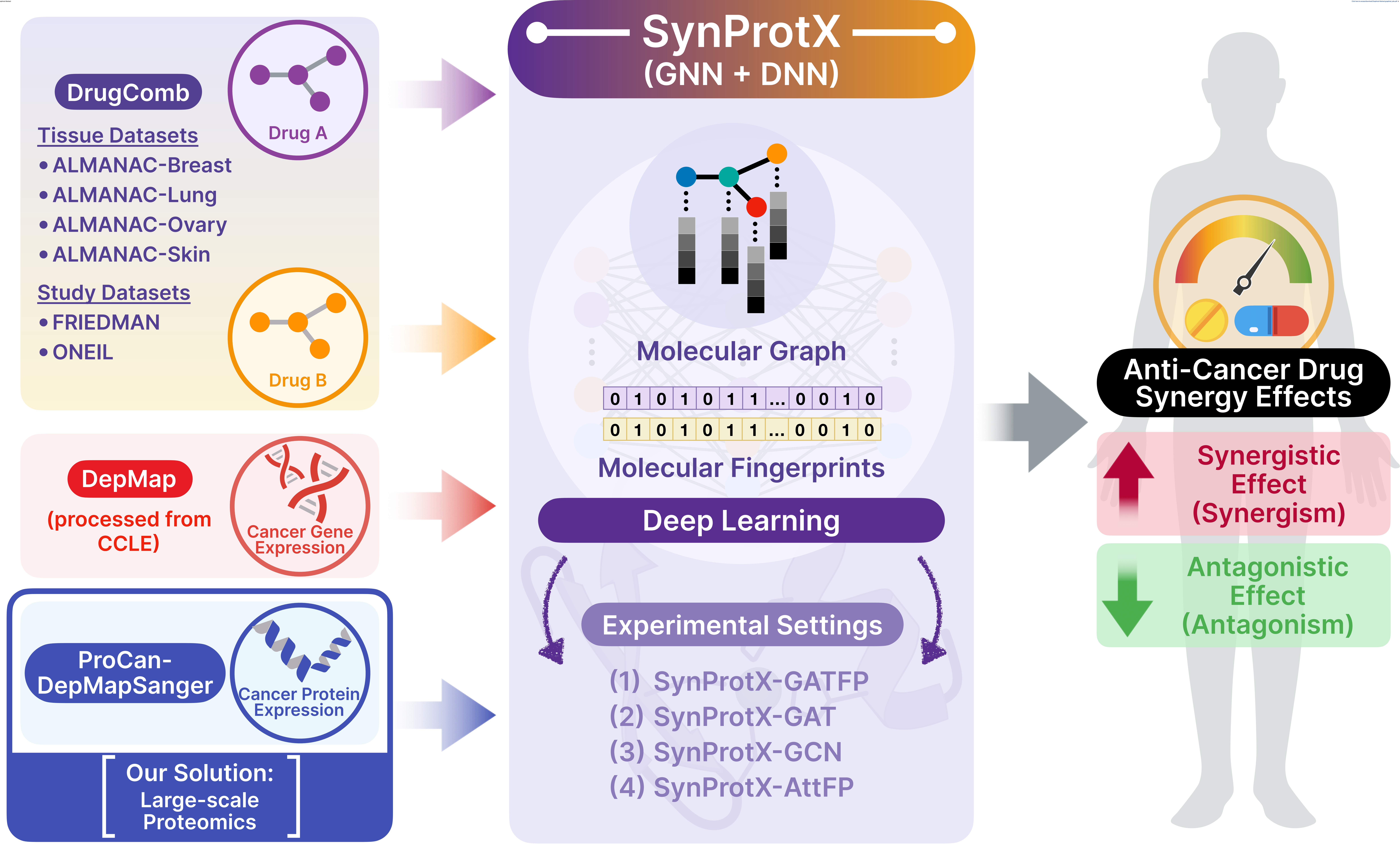
DrugComb
Drug A
Tissue Datasets
• ALMANAC-Breast
• ALMANAC-Lung
• ALMANAC-Ovary
• ALMANAC-Skin
Study Datasets
• FRIEDMAN
• ONEIL
Drug B
DepMap
(processed from CCLE)
Cancer Gene Expression
ProCan-DepMapSanger
Cancer Protein Expression
Our Solution: Large-scale Proteomics
SynProtX
(GNN + DNN)
Molecular Graph
0 1 0 1 0 1 1 ... 0 0 1 0
0 1 0 1 0 1 1 ... 0 0 1 0
Molecular Fingerprints
Deep Learning
Experimental Settings
(1) SynProtX-GATFP
(2) SynProtX-GAT
(3) SynProtX-GCN
(4) SynProtX-AttFP
Anti-Cancer Drug Synergy Effects
Synergistic Effect (Synergism)
Antagonistic Effect (Antagonism)
