# Supplementary material for: SynProtX: a large-scale proteomics-based deep learning model for predicting synergistic anticancer drug combinations
Source: Gigascience. 2025 Aug 12;14:giaf080. doi: 10.1093/gigascience/giaf080 (PMC12343095; doi:10.1093/gigascience/giaf080)
Supplement: giaf080_Authors_Response_To_Reviewer_Comments_Original_Submission [file giaf080_authors_response_to_reviewer_comments_original_submission.pdf]

Author's Response To Reviewer Comments

Note: The sections that have been revised are highlighted in yellow in the main manuscript.

Reviewer #1:

Synergistic drug combination prediction is important for disease treatment. However, it remains an open problem to identify the effective drug combinations in real clinical treatments. Computational models that can predict the drug combinations by integrating chemical and omic features can be helpful for the drug combination research. In this study, the authors proposed a deep learning model for drug combination prediction. Here are some comments might be helpful.

Author’s response:
We sincerely thank Reviewer #1 for their thoughtful and constructive feedback. Your comments have been invaluable in refining the manuscript and improving the clarity of our findings. We have carefully addressed each of your concerns, and below, we provide detailed responses to your points. We believe the revisions have significantly strengthened our work, and we appreciate your contribution to enhancing the manuscript.

1. The model design innovation is limited and also comparison results showed limited improvements of the proposed method than other existing models. So it is important to clearly indicate the unique contributions of the proposed model than existing models.

Author’s response:
We understand the concern regarding the novelty of our model design. The primary innovation in our study lies in the explicit integration of large-scale proteomics data alongside molecular graphs and molecular fingerprints. This large-scale proteomics data with the slightly modified approach has not been extensively explored in the anticancer drug combination prediction. Our model, SynProtX, incorporates multi-omics data (proteomics, transcriptomics, and drug molecular structure) to predict drug synergy. This comprehensive methodology has demonstrated improvements in predictive performance compared to existing state-of-the-art models, especially in the ablation study – w/o protein level data can significantly drop predictive performance when compared to w/o gene expression data. We have enhanced the Background section (Lines 87–159) to emphasize how current models primarily rely on genomic or transcriptomic data, which limits their ability to fully capture the functional states of cells. By explicitly integrating proteomics data, we improve the accuracy of synergy predictions by providing a more holistic view of the cellular environment. Furthermore, we stated the research question: Can large-scale proteomics data, combined with molecular graphs and fingerprints, improve model performance compared to traditional gene expression data alone? Additionally, we included the research evidence of “DeePathNet”, a transformer-based deep learning model for cancer monotherapy drug response prediction. DeePathNet integrates protein expression data from the ProCan-DepMapSanger database, demonstrating the value of incorporating proteomics for improving predictive accuracy.

Additionally, we evaluated the O’Neil (ONEIL) dataset as a small-scale dataset to observe the model’s ability to handle smaller, multi-tissue data scenarios. This evaluation is important for understanding how SynProtX performs on more limited datasets. We found that while SynProtX performed well across tissue-specific datasets, the ONEIL dataset revealed some interesting phenomena regarding the model’s adaptability to smaller-scale data, which are discussed in the Results and Discussion section.

Furthermore, we have added a Limitations of the Study section (Lines 759–791) that discusses the constraints of SynProtX model, such as model’s ability to generalize to unseen data, particularly in cold-start scenarios, and model overfitting in ONEIL dataset in small-scaled dataset and cell lines span multiple tissue types.

In the Potential Implications section (Lines 793–820), we discuss how the incorporation of proteomics data in SynProtX has important implications for personalized medicine to other diseases, drug toxicity

prediction, and cancer biomarker discovery. The ability to predict drug synergy with a more comprehensive dataset can lead to more precise treatment strategies, offering new avenues for improving therapy outcomes.

2. From a large number of features, it remains challenging to identify the important features contributing to the model prediction. Without systematic evaluation, it is not sound to conclude the importance of the identified features. So it is important to systematically evaluate the importance/score distributions of the all the features/the top-ranked features.

Author’s response:
We agree that a more systematic evaluation of feature importance is necessary. To address this, we have conducted a comprehensive feature importance analysis using integrated gradients (IG) (Figures 9 and 10). This allows us to rank and evaluate the contribution of each feature (including protein expression and gene expression) to the model’s predictions. The revised manuscript now includes a detailed description of these results in “Protein Level Improves Model Predictive Performance for SynProtX-GATFP” section (Lines 470–578). Here, we provide insights into how individual features influence the model’s performance across different datasets. Specifically, we found that protein expression plays a significant role in improving the model’s predictive accuracy.

In addition, we have included research evidence supporting the top-ranked proteins based on their involvement in biochemical pathways specific to the tissue types in the datasets. This highlights how protein-level features can contribute to understanding the mechanisms driving drug synergy.
The methodology of IG is elaborated further in the “Model Interpretability” section (Lines 1021–1073). Moreover, this section describes the attention weight strategy used to support the results presented in Figure 14. This attention mechanism enhances the interpretability of the model, allowing us to identify the most influential chemical substructure features in drug synergy predictions.

3. There are some ‘block effects’ of some drugs, which means that some drugs always works better than other drugs. Are these features associated with these drugs? If yes, not much meaningful or informative as it is just a simple correlation. It is important to identify the key mechanisms of cell-specific response drugs.

Author’s response:
We acknowledge that the “block effects” observed in some drug combinations need to be explored in greater depth. In response, we have expanded the analysis to include pathway-enrichment analyses (GSEA) and molecular fragment analysis in the section “Integrated Gradients (IG) Method of SynProtX-GATFP Explains Block Effects” (Lines 697–758 and Supplementary Tables S17 and S18). For the methodology, we applied IG to assess the contribution of individual protein features, focusing on the most influential features identified through ablation studies, to the drug combination synergy prediction. Specifically, we focused on combinations of top-proportion drugs found in the test set, including vismodegib–mitotane via the MDA-MB-468 and MCF-7 cell lines of the ALMANAC-Breast dataset, and vandetanib–gefitinib via the NCI-H226 and A549 cell lines of the ALMANAC-Lung dataset.

The results of these analyses show that vismodegib–mitotane and vandetanib–gefitinib exhibit consistent block effects across these cell lines, suggesting that certain drugs in the combinations play dominant roles in driving synergy. The pathway-enrichment analysis further revealed that specific signaling pathways, such as EGFR and MAPK pathways, were significantly enriched in the most influential proteins. This deeper mechanistic understanding goes beyond simple correlation, providing novel insights into the key biological mechanisms responsible for drug synergy.

4. It is important to evaluate the model performance on cell-specific.

Author’s response:
We have now included an evaluation of the model’s performance on cell-specific data, which was not adequately addressed in the initial version. In the revised manuscript, we have dedicated the section titled “SynProtX-GATFP Affirms the Performance on Cell-specific Responses” (Lines 406-469) to thoroughly describe the model's performance on individual cell lines within specific cancer types. The evaluation of cell-specific responses is critical for assessing how well SynProtX can predict drug synergy within different

cancer cell lines, which may vary in their drug response profiles.

This evaluation is supported by Supplementary Tables S4–S15, which provide detailed performance metrics across cell lines for different datasets such as ALMANAC-Breast, ALMANAC-Lung, ALMANAC-Ovary, ALMANAC-Skin, FRIEDMAN, and ONEIL for regression and classification tasks. The results demonstrate that SynProtX is capable of making highly accurate predictions for individual cell lines, accounting for their unique molecular and proteomic profiles. The ability to evaluate cell-specific data is a major strength of our model, as it enhances the precision and relevance of predictions for personalized cancer treatments.

We sincerly thank Reviwer #1 for their invaluable suggestion to evaluate the model performance on cell-specific. We agree that this is an important aspect to consider, as different cell lines may exhibit distinct drug response profiles. By evaluating SynProtX on cell-specific data, we can better understand how the model performs across various biological contexts and ensure that it captures the unique molecular and proteomic characteristics of each cell type. This approach significantly enhances the model's ability to predict drug synergy in a more personalized manner

5. It is important to use ‘cold-start’ (remove all the information of given cells or given drugs from the training data to evaluate the model performance).

Author’s response:
To address concerns about generalizability, we have implemented cold-start evaluation methods, including leave-drug-combination-out, leave-drug-out, and leave-cell-line-out strategies, to assess the generalizability of our model on unseen data (Figures 11–13). We would like to note that the leave-drug-combination-out result was included in the original manuscript.

The results of these cold-start validations are now presented in the section “SynProtX-GATFP Demonstrates Generalization Capability on Cold-start Scenarios” (Lines 579–622), demonstrating the model’s ability to perform well even with previously unseen drug combinations and cell lines. This is discussed in the revised manuscript, where we emphasize the robustness and applicability of our model in clinical settings. However, we address the constraints of both leave-drug-out and leave-cell-line-out strategies.

Leave-Drug-Combination-Out: This strategy involves excluding drug combinations from the training data and evaluating the model’s ability to predict drug synergy for these unseen combinations. The strength of this method is that it provides a more robust evaluation of the model’s ability to generalize across different drug interactions, which is critical in drug synergy prediction. It ensures that the model can predict combinations it has never seen before, making it highly relevant for clinical scenarios where new drug combinations are constantly being explored. The strength of this method lies in its ability to simulate real-world applications where new drug combinations are tested.

Leave-Drug-Out: In this strategy, one drug is excluded from the training data, and the model’s performance is evaluated based on the prediction of drug synergy for combinations involving these unseen drugs. The constraint of this method lies in the fact that the model may have limited exposure to the drug’s specific interactions with other drugs, which may affect prediction accuracy for novel drug combinations. This strategy is essential for evaluating the model’s ability to generalize to unseen drugs.

Leave-Cell-Line-Out: Here, the training data is split by excluding specific cell lines, and the model is tested on its ability to predict drug synergy in these cell lines. The constraint of this method is that it may reduce the representativeness of the training data, as the model is not exposed to the specific molecular and phenotypic characteristics of the excluded cell lines during training. This may result in reduced prediction accuracy for cell lines with distinct molecular profiles, especially if these cell lines significantly differ from those in the training set.

Reviewer #2:

In this paper, the authors propose a drug combination synergy prediction model using molecular graphs, molecular fingerprints, gene expression profiles, and proteomics information. The model is evaluated on

five single cancer type sub-datasets of the DrugComb dataset instead of utilizing the full dataset, which limits generalizability. The use of DNN architectures and graph neural networks are not innovative, given that similar methods (e.g. DeepDDS) have already been widely explored. Although the proposed model outperforms the compared baselines, it is unclear whether baselines are strong enough.

Due to the poor performance (low AUROC), lack of generalizability, and insufficient novelty, I do not recommend acceptance of this manuscript in its current form.

Author’s response:

We thank Reviewer #2 for their comments. Your feedback has been incredibly valuable in helping us refine the manuscript, particularly with regard to the generalizability of our approach. We have carefully considered each of your points and have made several revisions to address the concerns raised. In the following, we provide detailed responses to your comments, along with our rebuttal and clarifications. We believe these changes not only address your concerns but also contribute to enhancing the clarity and impact of our findings.

Generalizability Across Datasets:
We understand your concern regarding the generalizability of our model, especially since our initial evaluation was focused on single cancer-type datasets. To better evaluate the generalizability of SynProtX, we have employed leave-one-out validation (cold-start experiment), which includes leave-drug-combination-out, leave-drug-out, and leave-cell-line-out strategies across both tissue-specific (ALMANAC-Breast, ALMANAC-Lung, ALMANAC-Ovary, ALMANAC-Skin) and study datasets (ONEIL and FRIEDMAN). This expanded validation approach allows us to demonstrate that SynProtX generalizes effectively across different biological contexts, making it a more robust model for drug synergy prediction. This highlight is presented in the section of “SynProtX-GATFP Demonstrates Generalization Capability on Cold-start Scenarios” (Lines 579–622).

Furthermore, we need to clarify that using the full DrugComb dataset could introduce challenges that may affect model reliability due to data quality and heterogeneity. Each sub-dataset in the DrugComb collection comes from different experiments, and the dose-response matrix size (Supplementary Table S2) varies between them, which can lead to inconsistent data distribution across the full dataset. This variability is an important factor to consider when evaluating model performance. Specifically, the subsets of the DrugComb dataset represent data collected from different experimental protocols and data collection methodologies, which can lead to significant variations in data quality and completeness. These discrepancies introduce heterogeneity, making it difficult to train a single model on the entire dataset due to the inconsistency in experimental conditions. Another important issue arises from cell line redundancy within each sub-dataset. Many sub-datasets, such as those from the ALMANAC-Skin or FRIDEMAN collections, may contain repeated use of the same cell lines in multiple drug combinations. This redundancy can lead to overfitting and an unfair or biased evaluation, as the model may inadvertently learn patterns from overrepresented cell lines. In such cases, the model becomes highly specialized in recognizing these familiar patterns, which limits its ability to generalize effectively to novel cell lines or drug combinations that were underrepresented or entirely absent in the training data.

Given these challenges, we chose to evaluate SynProtX on tissue-specific and study sub-datasets rather than the full DrugComb dataset. This allows us to focus on more homogeneous data, thereby mitigating issues arising from cell line redundancy, experimental variability, and dose-response matrix size variability. By using focused sub-datasets, we ensure that the evaluation is based on consistent and uniform data, leading to more accurate and reliable predictions.

Comparison with Existing Models & Novelty:
We appreciate your comments on the novelty of our approach, particularly given that models like DeepDDS and DeepSynergy have already been widely explored. While these models have significantly contributed to the field, we believe that our approach represents a substantial advancement due to its integration of large-scale proteomics data alongside molecular graphs and fingerprints. The hypothesis guiding this study was: “Can large-scale proteomics data, combined with molecular graphs and fingerprints, improve model performance compared to traditional gene expression data alone?” (Lines 100–125). This multi-omics

approach offers a more comprehensive understanding of drug synergy by providing a holistic view of the cellular environment, which ultimately improves predictive accuracy. Furthermore, we are pleased to submit this manuscript to GigaScience, a prominent journal in the field of big data-driven life sciences applications, as we believe our work aligns with the journal’s focus on computational approaches for solving complex biological problems.

To clearly demonstrate the performance of SynProtX, we have added a more detailed comparison with existing models in Figures 3 and 4 and Supplementary Tables S2 and S3. These comparisons highlight SynProtX’s high performance in both regression and classification tasks. Additionally, the Results and Discussion sections (Lines 309–365, 406–469, and 470–578) further demonstrate the advantages of our approach in comparison to other baseline models.

We expanded the Background section (Lines 87–125) to address the limitations of current models, such as their reliance on genomic data alone, which often fails to capture the complexity of drug interactions. Our multi-omics approach, particularly the inclusion of proteomics data, overcomes these limitations, leading to a more accurate prediction of drug synergy.

Model Performance & Cell-Specific Response Evaluation:
We appreciate your comment on the AUROC score and agree that the performance across different datasets warrants further clarification. While SynProtX performs well on several tissue-specific datasets, we observed some variability in the O’Neil (ONEIL) dataset (expanded experiment in revised version), where it was marginally outperformed by XGBoost. We have now included a detailed discussion of these, where we suggest that ensemble-based methods like XGBoost may outperform individual deep learning models in certain scenarios, particularly with smaller or more heterogeneous datasets.

In addition, we expanded the experiment on cell-specific responses to provide a more thorough analysis of how SynProtX performs across different cancer cell lines. In the revised manuscript, we present a dedicated section titled “SynProtX-GATFP Affirms the Performance on Cell-Specific Responses” (Lines 406–469), where we evaluate the model’s performance on specific cancer cell lines within the ALMANAC-Breast, ALMANAC-Lung, ALMANAC-Ovary, ALMANAC-Skin, FRIEDMAN, and ONEIL datasets. The results demonstrate that SynProtX is capable of making highly accurate predictions for individual cell lines, accounting for their unique molecular and proteomic profiles. The ability to evaluate cell-specific data is a major strength of our model, as it enhances the precision and relevance of predictions for personalized cancer treatments.

We investigated block effects in drug combinations in section of “Integrated Gradients (IG) Method of SynProtX-GATFP Explains Block Effects” (Lines: 697–758). Block effects occur when one drug in a combination dominates the synergistic effect, causing the synergy to be primarily driven by this drug across multiple cell lines. To identify these block effects, we applied IG to assess the contribution of individual protein features, focusing on the most influential features, to the drug combination synergy prediction. The IG method is particularly useful for identifying the most influential proteins for predicting drug synergy, especially in cell-specific contexts. Our results show that SynProtX performs robustly across different cancer cell lines, accurately identifying drug synergy patterns that are unique to each cancer type. This was particularly evident in the ALMANAC-Breast and ALMANAC-Lung datasets, where we observed consistent predictive accuracy. These findings demonstrate that SynProtX is capable of capturing cell-specific drug synergy by accounting for the unique molecular and proteomic characteristics of each cell line. This ability to tailor predictions based on the specific features of individual cell lines underscores the model’s potential for personalized drug combination strategies, which is one of the key strengths of SynProtX.

Furthermore, we have conducted an ablation study titled “Protein Level Improves Model Predictive Performance for SynProtX-GATFP” (Lines 470–578). This ablation study demonstrates the significant impact of protein-level data on model performance. By isolating the influence of protein-level features, we show that including proteomics data substantially improves the model’s predictive accuracy compared to models relying solely on gene expression data. This study provides strong evidence supporting the importance of protein data for predicting drug synergy.

Cookie Preference Center

We use cookies which are necessary to make our site work. We may also use additional cookies to analyze, improve and personalize our content and your digital experience. For more information, see our Cookie Policy.

You may choose not to allow some types of cookies. However, blocking some types may impact your experience of our site and the services we are able to offer. See the different category headings below to find out more or change your settings.

Allow all

Manage Consent Preferences

Strictly Necessary Cookies

Always active

These cookies are necessary for the website to function and cannot be switched off in our systems. They are usually only set in response to actions made by you which amount to a request for services, such as setting your privacy preferences, logging in or filling in forms. You can set your browser to block or alert you about these cookies, but some parts of the site will not then work.

Functional Cookies

These cookies enable the website to provide enhanced functionality and personalisation. They may be set by us or by third party providers whose services we have added to our pages. If you do not allow these cookies then some or all of these services may not function properly.

Performance Cookies

These cookies allow us to count visits and traffic sources so we can measure and improve the performance of our site. They help us to know which pages are the most and least popular and see how visitors move around the site.

Targeting Cookies

These cookies may be set through our site by our advertising partners. They may be used by those companies to build a profile of your interests and show you relevant adverts on other sites. If you do not allow these cookies, you will experience less targeted advertising.

Cookie List

Clear

Apply Cancel
Consent Leg.Interest

Confirm my choices

Powered by onetrust
