# Supplementary material for: SynProtX: a large-scale proteomics-based deep learning model for predicting synergistic anticancer drug combinations
Source: Gigascience. 2025 Aug 12;14:giaf080. doi: 10.1093/gigascience/giaf080 (PMC12343095; doi:10.1093/gigascience/giaf080)
Supplement: giaf080_Authors_Response_To_Reviewer_Comments_Revision_1 [file giaf080_authors_response_to_reviewer_comments_revision_1.pdf]

Author's Response To Reviewer Comments

Note: The sections that have been revised for the first and second rounds are highlighted in yellow and cyan in the main manuscript, respectively.

Reviewer #1:

It might not appropriate to emphasize or claim 'Furthermore, SynProtX outperformed existing state-of-the-art models across diverse cancer tissue datasets ...' considering the limited improvement. Better to identify the unique contributions and edit the strong conclusions without solid evaluation support.

Author’s response:
We sincerely thank the Reviewer #1 for the continued constructive feedback and thoughtful critique. In response to the concern regarding the statement in the Abstract that SynProtX “Furthermore, SynProtX outperformed existing state-of-the-art models across diverse cancer tissue datasets from the ALMANAC study, including breast, lung, ovary, and skin tissues.” we have revised the language in the Abstract and Introduction sections to better reflect the nature of our findings without overstating the model’s comparative advantage.

In the revised Abstract (Lines 41–44) and Introduction (Lines 139–141 and Lines 159–161), we now emphasize the unique contribution of SynProtX, the integration of proteomic expression data which consistently improves predictive performance compared to models based solely on gene expression. We also highlight the cell-line-specific evaluation and ablation studies as central strengths that support the model’s functional relevance and biological interpretability

Revised Abstract sentence:
(Lines 41–44)
We further evaluated its cell-line-specific performance, which achieved accuracy across diverse tissue and study datasets. By incorporating protein expression data, the model consistently enhanced predictive performance over gene-expression-only models, reflecting the functional state of cancer cells.

Revised Introduction sentence:
(Lines 139–141)
Cell-line-specific evaluations further show that the model achieves accuracy across various tissue and study datasets. Ablation studies reveal that incorporating protein-level expression consistently improves predictive performance compared to models based solely on gene expression.

(Lines 159–161)
• We evaluated the model performance at the cell-line-specific level and conducted ablation studies to assess the impact of protein- and gene-level features on predictive accuracy.

We appreciate the reviewer’s guidance in helping us refine the positioning of our conclusions and enhance the scientific rigor of the manuscript.

Reviewer #2:

The authors have made substantial efforts to address the comments from the first round. The manuscript is clearer, and the methodology is more thoroughly explained. The additional validation results and improved discussion of the proteomics data strengthen the work.

I would support publication of the manuscript.

Author’s response:
We sincerely thank the Reviewer #2 for the positive feedback and support for the publication of our work.

We are pleased that the additional validations and improved discussion of the proteomics data strengthened the manuscript and clarified the methodology. We greatly appreciate your thoughtful review and encouragement.

Cookie Preference Center

We use cookies which are necessary to make our site work. We may also use additional cookies to analyze, improve and personalize our content and your digital experience. For more information, see our Cookie Policy.

You may choose not to allow some types of cookies. However, blocking some types may impact your experience of our site and the services we are able to offer. See the different category headings below to find out more or change your settings.

Allow all

Manage Consent Preferences

Strictly Necessary Cookies

Always active

These cookies are necessary for the website to function and cannot be switched off in our systems. They are usually only set in response to actions made by you which amount to a request for services, such as setting your privacy preferences, logging in or filling in forms. You can set your browser to block or alert you about these cookies, but some parts of the site will not then work.

Functional Cookies

These cookies enable the website to provide enhanced functionality and personalisation. They may be set by us or by third party providers whose services we have added to our pages. If you do not allow these cookies then some or all of these services may not function properly.

Performance Cookies

These cookies allow us to count visits and traffic sources so we can measure and improve the performance of our site. They help us to know which pages are the most and least popular and see how visitors move around the site.

Targeting Cookies

These cookies may be set through our site by our advertising partners. They may be used by those companies to build a profile of your interests and show you relevant adverts on other sites. If you do not allow these cookies, you will experience less targeted advertising.

Cookie List

Clear

Apply Cancel
Consent Leg.Interest

Confirm my choices

Powered by onetrust
