# Supplementary material for: SynProtX: a large-scale proteomics-based deep learning model for predicting synergistic anticancer drug combinations
Source: Gigascience. 2025 Aug 12;14:giaf080. doi: 10.1093/gigascience/giaf080 (PMC12343095; doi:10.1093/gigascience/giaf080)
Supplement: giaf080_Reviewer_1_Report_Original_Submission — Fuhai Li -- 2/10/2025 [file giaf080_reviewer_1_report_original_submission.pdf]

Reviewer Report

Title: SynProtX: A Large-Scale Proteomics-Based Deep Learning Model for Predicting Synergistic Anticancer Drug Combinations

Version: Original Submission **Date:** 2/10/2025

Reviewer name: Fuhai Li

Reviewer Comments to Author:

Synergistic drug combination prediction is important for disease treatment. However, it remains an open problem to identify the effective drug combinations in real clinical treatments. Computational models that can predict the drug combinations by integrating chemical and omic features can be helpful for the drug combination research. In this study, the authors proposed a deep learning model for drug combination prediction. Here are some comments might be helpful.
1) The model design innovation is limited and also comparison results showed limited improvements of the proposed method than other existing models. So it is important to clearly indicate the unique contributions of the proposed model than existing models.
2) From a large number of features, it remains challenging to identify the important features contributing to the model prediction. Without systematic evaluation, it is not sound to conclude the importance of the identified features. So it is important to systematically evaluate the importance/score distributions of the all the features/the top-ranked features.
3) There are some 'block effects' of some drugs, which means that some drugs always works better than other other drugs. Are these features associated with these drugs? If yes, not much meaningful or informative as it is just a simple correlation. It is important to identify the key mechanisms of cell-specific response drugs.
4) It is important to evaluate the model performance on cell-specific.
5) It is important to use 'cold-start' (remove all the information of given cells or given drugs from the training data to evaluate the model performance).

Methods

Are the methods appropriate to the aims of the study, are they well described, and are necessary controls included? Choose an item.

Conclusions

Are the conclusions adequately supported by the data shown? Choose an item.

Reporting Standards

Does the manuscript adhere to the journal's guidelines on [minimum standards of reporting?]() Choose an item.

Choose an item.

Statistics

Are you able to assess all statistics in the manuscript, including the appropriateness of statistical tests used? Choose an item.

Quality of Written English

Please indicate the quality of language in the manuscript: Choose an item.

Declaration of Competing Interests

Please complete a declaration of competing interests, considering the following questions:

- Have you in the past five years received reimbursements, fees, funding, or salary from an organisation that may in any way gain or lose financially from the publication of this manuscript, either now or in the future?
- Do you hold any stocks or shares in an organisation that may in any way gain or lose financially from the publication of this manuscript, either now or in the future?
- Do you hold or are you currently applying for any patents relating to the content of the manuscript?
- Have you received reimbursements, fees, funding, or salary from an organization that holds or has applied for patents relating to the content of the manuscript?
- Do you have any other financial competing interests?
- Do you have any non-financial competing interests in relation to this paper?

If you can answer no to all of the above, write 'I declare that I have no competing interests' below. If your reply is yes to any, please give details below.

I declare that I have no competing interests

I agree to the open peer review policy of the journal. I understand that my name will be included on my report to the authors and, if the manuscript is accepted for publication, my named report including any attachments I upload will be posted on the website along with the authors' responses. I agree for my report to be made available under an Open Access Creative Commons CC-BY license (http://creativecommons.org/licenses/by/4.0/). I understand that any comments which I do not wish to be included in my named report can be included as confidential comments to the editors, which will not be published.

Choose an item.

To further support our reviewers, we have joined with Publons, where you can gain additional credit to further highlight your hard work (see: https://publons.com/journal/530/gigascience). On publication of this paper, your review will be automatically added to Publons, you can then choose whether or not to claim your Publons credit. I understand this statement.

Yes Choose an item.
